# A novel food processing-based nutrition classification scheme for guiding policy actions applied to the Australian food supply

**DOI:** 10.3389/fnut.2023.1071356

**Published:** 2023-01-20

**Authors:** Sarah Dickie, Julie Woods, Priscila Machado, Mark Lawrence

**Affiliations:** School of Exercise and Nutrition Science, Institute for Physical Activity and Nutrition (IPAN), Deakin University, Geelong, VIC, Australia

**Keywords:** nutrition classification, food classification, NOVA, ultra-processed food, nutrient profiling, food policy, front-of-pack labelling (FOPL), food tax

## Abstract

Unhealthy diets are a leading risk factor for non-communicable diseases and negatively impact environmental sustainability. Policy actions recommended to address dietary risk factors, such as restrictions on marketing and front-of-pack labelling, are informed by nutrition classification schemes (NCSs). Ultra-processed foods are associated with adverse population and planetary health outcomes, yet the concept is rarely incorporated in nutrition classification schemes for policy actions. This study aims to develop a novel food processing-based nutrition classification scheme for guiding policy actions. A secondary aim is to validate the scheme by classifying food and beverage items in the Australian food supply (face validity) and comparing them to the classifications of existing NCSs (convergent validity). Two versions of a model were developed, classifying foods and beverages in two steps, first using the NOVA classification system and secondly by applying upper thresholds for added free sugars and sodium, producing a binary output of either healthy or unhealthy. All food and beverage items (*n* = 7,322) in a dataset combining the Australian Food Composition Database (AUSNUT 2011–2013) and Mintel’s Global New Product Database (2014–2019) were classified using the two models. The same dataset was also classified by the Health Star Rating system (HSR), The Australian Dietary Guidelines (ADGs), The Pan American Health Organization’s Nutrient Profile Model (PAHO NPM), and the NOVA classification scheme, and pairwise agreement between all NCSs and the two models was determined (using Cohen’s Kappa coefficient). A higher proportion of food categories consistent with dietary patterns that are associated with positive health outcomes, such and fruits, vegetables, and eggs were classified as healthy. And the clear majority of food categories consistent with dietary patterns associated with adverse health outcomes, such as confectionery, snack foods, and convenience foods were classified as unhealthy. The two versions of the model showed substantial agreement with NOVA and the PAHO NPM, fair agreement with the ADGs and slight to moderate agreement with the HSR system. A model NCS combining level of processing and nutrient criteria presents a valid alternative to existing methods to classify the health potential of individual foods for policy purposes.

## 1. Introduction

Unhealthy diets are a leading risk factor for non-communicable diseases and negatively impact environmental sustainability (1, 2). Policy actions such as restrictions on marketing, taxes and subsidies, front-of-pack labelling, and regulation of health claims have been recommended to tackle dietary risk factors (3–5). However, growing evidence indicates nutrient-based nutrition classification schemes, such as the Health Star Rating (HSR) system and Nutri-score, do not align with nutrition science principles as they cannot differentiate degree of processing (6–8). Nutrient-based schemes only assess a handful of isolated nutrients or components, ignoring the more holistic concept of processing. The NOVA classification system categorises foods into four groups based on the nature, extent and purpose of processing, group 1–unprocessed or minimally processed foods, group 2–processed culinary ingredients, group 3–processed foods, and group 4–ultra-processed foods, defined as “*formulations of ingredients, mostly of exclusive industrial use, that result from a series of industrial processes*” (9). Ultra-processed foods are often hyper-palatable, convenient and heavily marketed, and can displace nutritious unprocessed and minimally processed foods in the diet (10). Diets that include higher proportions of ultra-processed foods are linked to several chronic diseases, including obesity, metabolic syndrome, cardiovascular disease, depression, and all-cause mortality (11, 12). The scale of their production, the intense industrial processes involved, extensive packaging, and unnecessary overconsumption also contribute considerably to greenhouse gas emissions, biodiversity loss, land degradation and increased water footprints (13, 14).

In a previous study we compared seven nutrient-based, food-based and dietary-based nutrition classification schemes (NCSs) and identified key characteristics that could be used to holistically identify foods to be encouraged (“healthy”) or discouraged (“unhealthy”) for guiding the design of policy actions (15). A holistic approach in the context of NCSs considers the food matrix (extent of degradation or preservation) and the complex synergies that exist between the nutrient and non-nutrient components [more than 26,000 bioactive components are present in foods (16)]. A reductionist approach, on the other hand, assesses foods for their content of specific nutrients with established health associations (e.g., excess sodium and hypertension). A reductionist approach used in isolation assesses a food’s health potential only as the sum of its parts, ignoring the complex structure in which nutrients are contained (17). We found that NCS’s that used a “top-down” orientation, i.e., first assessing the holistic characteristics of a food’s health potential before assessing reductionist characteristics (17) identified more foods as being unhealthy [when unhealthy was defined as “discretionary” by the Australian Dietary Guidelines (ADGs) or ultra-processed].

Food-based NCSs following a top-down approach, such as the Pan American Health Organization’s Nutrient Profiling Model (PAHO NPM) and the World Health Organization European Region’s Nutrient Profile Model (WHO-Euro NPM), can more often identify unhealthy foods (when unhealthy is defined as ultra-processed) (15). However, limitations were identified in these schemes, including ambiguous food category classification and the assessment of certain nutrients, such as total fat, which resulted in the penalisation of some whole foods (overlooking holistic concepts). Thus, there is potential to reform existing NCSs to be more fit-for-purpose in informing policy actions, a particularly timely and important task considering current plans to harmonise NCSs internationally (18, 19).

Effective nutrition classification for policy actions should help shift the food supply away from ultra-processed foods toward dietary patterns consisting mostly of unprocessed and minimally processed foods. It is proposed that such nutrition classification can be improved by applying the key characteristics of NCSs identified in our previous research. This would involve a top-down holistic approach with assessment of degree of processing as the first step and application of upper thresholds for risk nutrients as the second step. Therefore, this study aims to develop a novel food processing-based nutrition classification scheme for guiding policy actions. A secondary aim is to validate the scheme by classifying food and beverage items in the Australian food supply (face validity) and comparing them to the classifications of existing NCSs (convergent validity).

## 2. Materials and methods

### 2.1. Model development

Model development was guided by 7 technical design characteristics of NCSs identified in our previous research (15). The characteristics explained the key technical differences between different NCSs that resulted in better ability to identify unhealthy foods (when unhealthy is defined as discretionary or ultra-processed). Each characteristic has been addressed as a requirement in the current model. Nutrition policy actions usually require a method to unambiguously identify unhealthy foods (e.g., taxing unhealthy foods or front-of-pack warning labels) (20). Therefore, a fit-for-purpose approach also informed model development, whereby the researchers intended the model to be used for policy actions in which an accurate classification of unhealthy is the primary purpose. For this reason, the resulting classification is binary, with one of two outcomes - either healthy or unhealthy (permitted or not permitted for a specific policy purpose). It is acknowledged that not all individual foods can be classified as “healthy” or “unhealthy” in absolute terms as the health potential of foods depends on the context of an individual’s overall dietary pattern. The terms “healthy” and “unhealthy” have been chosen as they clearly convey the intended meaning of the classifications, and the terms are not intended to be used in the application to policy. Through development it was found that the binary approach may lead to a small number of food items that are not identified as “unhealthy” despite subjectively appearing to be, and similarly for some classified as “healthy.” Adjustment may therefore be required to ensure the model is fit-for-purpose for policy actions in which the accurate identification of healthy foods is the priority (e.g., healthy food subsidies). Ease of use is also an important requirement for an NCS to successfully inform policy by reducing burden on manufacturers and regulators, therefore this was a consideration in all decisions made.

Characteristic 1: Consideration of level of processing.

• To consider level of processing, the NOVA classification system was chosen. NOVA is considered the most objective, comprehensive, and clear scheme to incorporate food processing into classification of diets (21). NOVA has been incorporated into the dietary guidelines of Brazil, Uruguay, Ecuador, Peru, Israel, and Malaysia (10). When used for dietary advice, the recommendation is to mostly consume foods or ingredients that fall into NOVA groups 1–3 (with priority for NOVA group 1 foods), and reduce or avoid the consumption of NOVA group 4, ultra-processed foods (22). NOVA group 3 processed foods can be included as part of a healthy diet depending on their nutritional composition. NOVA has previously informed classification schemes at the food-level for regulatory purposes, such as the PAHO NPM (23).

Characteristic 2: Identification of specific food categories as always healthy or unhealthy.

• Characteristic 2 was also addressed by use of the NOVA categories. NOVA identifies group 1, unprocessed and minimally processed foods, as the least processed (9). These foods have undergone no or minimal processing methods, such as chopping, grinding, boiling and fermentation, and do not contain any added processed culinary ingredients (NOVA group 2), such as salt, sugars, and oils. Examples include fresh fruits and vegetables, natural unflavoured yoghurt, pasteurised milk, flours, and dried pasta. NOVA group 1 foods were automatically identified as healthy without any additional criteria. Similarly, NOVA group 2 foods are used in home culinary preparations and are rarely consumed by themselves but added to NOVA group 1 foods for additional flavour, to enhance the cooking process or for preservation, and therefore it is inappropriate to assess their nutritional attributes. NOVA group 2 ingredients were included in the “healthy” category as it is unnecessary for them to be identified as “unhealthy” for regulatory purposes. If salt or sugar is added in excessive quantities in the manufacturing process, this will be identified in the assessment of NOVA group 3 foods (processed foods). As foods that should be limited or avoided, ultra-processed foods were automatically classified as unhealthy. Ultra-processed foods are identified based on the presence of a marker of ultra-processing, i.e., a substance extracted from foods (e.g., modified starches, protein isolates) or a cosmetic additive (e.g., flavours, colours, thickeners) (9).

Characteristic 3: Category-specific nutrient criteria.

• Characteristic 3 is addressed by only assessing the nutrient content of NOVA group 3 foods. These foods are manufactured products that result from the addition of group 2 culinary ingredients to group 1 foods, examples include cheeses, fresh bakery-style breads, canned vegetables or legumes in brine, salted nuts, and cured or smoked meats and fish. The recommendation in dietary guidelines to date is not to avoid group 3 foods completely, but to limit their consumption. Our previous research found some can contain high levels of added salt or sugars, such as beef jerky, fruits in syrup, kombuchas, and sweet bakery products. Consuming high quantities of added sugars and salt has established adverse health outcomes (24–26). These foods can be seen as an unnecessary addition to a traditional diet mostly consisting of minimally processed foods. Therefore, the “unhealthier” versions of NOVA group 3 foods need to be identified for the model to be useful for policy and eliminate potential loopholes for manufacturers.

Characteristic 4: No allowance for the substitution of processed ingredients.

• Characteristic 4 is addressed as foods with substituted forms of sugar (non-nutritive sweeteners), or salt (flavour enhancers) are identified as ultra-processed.

Characteristic 5: Assessment of nutrients only when added as ingredients.

• Characteristic 5 can be addressed by only assessing the content of sugars and sodium added as ingredients in food products. When the content of sugars or sodium is high in NOVA group 3 foods these nutrients have typically been added *via* processed culinary ingredients (NOVA group 2). Because manufacturers also use ingredients that consist of concentrated or processed fruit to sweeten foods, the term “free sugars” as defined by the World Health Organization (WHO) has been used for this model, which includes fruit juices, fruit purees and fruit concentrates in addition to pure or refined sugars (e.g., honey or cane sugar). The use of free sugars prevents potential loopholes for manufacturers wherein concentrated or processed fruit products can be added as substitutions for traditional sugars. The justification for the inclusion of free sugars and sodium as “risk” nutrients of importance in this model is outlined below.

#### 2.1.1. Sodium

High sodium intake can increase blood pressure, a major risk factor for cardiovascular and renal diseases (26, 27). To reduce blood pressure, the WHO recommends reducing sodium intake to less than 2 g per day (or 5 g of salt) for adults (28). However globally, sodium intake averages 3.95 g per day, with sodium intake exceeding recommendations in almost all countries with available data in 2010 (29). In Australia, modelling has indicated that a reduction from the current average sodium intake of 3.6 to 2 g could reduce average population blood pressure (30). Although some sodium is naturally occurring in foods, most sodium is consumed in processed or ultra-processed foods, or through the addition of salt in food preparation (31).

#### 2.1.2. Free sugars

High intake of free sugars is associated with obesity, risk of NCDs, and dental caries (24, 32, 33). The WHO recommends reducing free sugars to under 10% of total energy intake (34). However, free sugar intake likely exceeds the recommendation in most countries (35). Intake of total sugar in countries with data available from nationally representative dietary surveys (18 countries) ranged from an average of 18% for infants to 20% for adults (35). In 2011–2012, Australians age 2 years and over on average consumed 11.7% of energy from free sugars, with just over half exceeding the WHO recommendation (36). The majority of free sugars are consumed in processed and ultra-processed foods, especially beverages (including fruit juice) (36).

Characteristic 6: Appropriate selection of risk nutrients that do not penalise whole foods.

• Existing nutrient-based classification schemes often include additional risk nutrients or components, including total fat, saturated fat, trans fat, non-nutritive sweeteners, and energy. The justifications for not including these components relates to characteristic 6 and are outlined below.
• Foods containing non-nutritive sweeteners and industrial trans fats are classified as NOVA group 4 ultra-processed foods, therefore these will be identified without setting specific criteria. Trans fats can also occur intrinsically in unprocessed dairy and meat (biohydrogenation), but at a lower level and potentially not as harmful as industrially generated trans fats (37).
• Total fats and saturated fats are often present intrinsically in whole foods, therefore assessing fat content can potentially penalise healthy whole foods. The assessment of total fats could be particularly problematic, as recommended foods such as nuts, seeds, fish, and olive oil contain high amounts of “healthy” monounsaturated and polyunsaturated fats. Furthermore, foods that consist largely of fats are composed of varying proportions of different types of monounsaturated, polyunsaturated and saturated fatty acids, all with differential effects on health (38). The association between consumption of saturated fats and adverse health outcomes is complex and controversial (38). For example, the food source of the saturated fat has been shown to attenuate any association, with dairy foods (whether low or full fat) having a neutral or positive effect on health, but processed meats having a negative effect (39–41). Assessment only of added saturated fats is also not a straightforward solution, as the source of fats in a food cannot be easily distinguished. Moreover, added fats such as butter, olive oil, and coconut milk exist as complex food matrices containing different types of fatty acids and non-fat components. Therefore, the assessment of fats in individual foods to explain a food’s health potential could be problematic.
• The justification for excluding energy follows a similar logic to fats. Whole foods and culinary ingredients that contribute positively to healthy diets, such as nuts, seeds, fish, and olive oil could be potentially penalised due to high energy content if restrictive criteria were included. Energy is more appropriate to consider when assessing consumption over whole dietary patterns.

Characteristic 7: Appropriate upper thresholds for risk nutrients that do not penalise whole foods.

• In line with characteristic 7, an upper threshold approach was chosen for the assessment of sodium and free sugars (*via* added ingredients).
• The first consideration to define upper thresholds of risk nutrients was whether to base the reference amount on weight, energy or serving size (per 100 g, per 100 kj/kcal, or per serving size). Serving size can be highly variable across different types of products and difficult to standardise across the food supply and as such was ruled out. The PAHO NPM uses an energy approach for free sugars, assessing content of risk nutrients based on the percentage energy they contribute to the total energy of the food. Our previous research found that the energy approach in the PAHO NPM could lead to products of low energy density being penalised despite low content of risk nutrients (by weight), for example cottage cheese, chickpea patties, milk, yoghurt, and salads. An energy approach could risk foods of low energy but high satiety, such as those containing fruits, vegetables, and legumes being penalised despite relatively low content of risk nutrients, and therefore would not align with the fit-for-purpose approach in which accurate classification of “unhealthy” foods is prioritised. Thus, weight (per 100 g) was considered the approach that could be applied with the fewest outliers and could be applied consistently for free sugars and sodium. A weight approach however can be confounded by water content, for example soups containing salt or sugar-sweetened beverages can appear low in sodium and sugar as their energy to weight ratio is low (low energy density). To avoid this issue, separate upper thresholds are specified for solid and liquid products (including soups and beverages). This solution is not perfect as it does not account for solid foods with high water content, for example yoghurts, and preserved vegetables and fruits (semi-solid foods or foods where water content is contained within the food matrix). However higher water content in these cases can contribute favourably to a food’s energy density and satiety, and can indicate a less degraded food matrix (42), and such misclassifications as “healthy” would not be of great concern. Potentially there will be exceptions to the outlined logic, and adjustments to the reference amount and nutrient thresholds may be needed post-analysis.
  ∘ A variety of sources were consulted to determine cut-offs to test in this study, as presented in Table 1. In addition, the range of sodium and free sugar values were examined in the dataset, and different values were modelled for relevant NOVA group 3 categories (e.g., soup, cheese, and bread for sodium). The upper thresholds decided on for each category are presented in Table 2.

**TABLE 1:** Policy examples in Australia and internationally of sodium and sugar thresholds that were consulted to decide on pilot thresholds for model.

	Sodium	Sugars
**Australian examples**		
Australian dietary guidelines educators guide	Recommends choosing foods under 400 mg/100 g	Recommends choosing foods under 15 g/100 g of total sugar
The Australian healthy food partnership’s reformulation targets	Ready meals–250 mg/100 g Soups–280 mg/100 ml Leavened bread–380 mg/100 g Pizza–450 mg/100 g Cheddar cheese–710 mg/100 g	Breakfast cereals–20 g/100 g total sugar Sweetened yoghurt–12.5 g/00 g total sugar Flavoured milk–9 g/100 g total sugar Flavoured waters–5 g/100 ml total sugar
Australian food standards nutrient claims	To make “low in salt” claim 120 mg/100 g or 120 mg/100 ml	To make “low in sugar” claim Foods–5 g/100 g total sugar Beverages–2.5 g/100 g total sugar
**International examples**		
The World Health Organization European region’s nutrient profiling model	Bread–480 mg/100 g Cheese–520 mg/100 g Processed meat–680 mg/100 g	For all food types except breakfast cereals–10 g/100 g total sugar Breakfast cereals–15 g/100 g total sugar No added sugar in beverages
Chilean nutrient profiling model (for the Chilean food act)	Solids–400 mg/100 g Liquids–100 mg/100 g	Solids–10 g/100 g added sugar Liquids–5 g/100 g added sugar
The United Kingdom’s multiple traffic light front-of-pack label	Red light for foods–600 mg/100 g Red light for beverages–300 mg/100 g	Red light for foods–22.5/100 g total sugar Red light for beverages–11.5 g/100 ml total sugar
Pan American Health Organization’s regional reformulation targets	Cheese–559 mg/100 g Bread–600 mg/100 g Snacks–900 mg/100 g	N/A

**TABLE 2:** Sodium and free sugar thresholds for the developed model.

	Sodium	Free sugars
Foods	450 mg/100 g	10 g/100 g
Cheese	650 mg/100 g	10 g/100 g
Beverages or liquids	250 mg/100 ml	5 g/100 ml

#### 2.1.3. Sodium thresholds

Foods exceeding 450 mg/100 g in sodium are classified as “high in sodium” or “unhealthy.”

Cheese is the only exception. Cheese products exceeding 650 mg/100 g of sodium are classified as “high in sodium.” Basic cheese products on average contain a higher amount of sodium compared to other nutritious foods recommended by the Australian Dietary Guidelines, but they are eaten in smaller portions compared to other categories such as bread or ready meals and are a valuable source of nutrition. Therefore, the threshold has been set higher to allow basic NOVA group 3 cheese products to pass the criteria.

Beverages or liquids exceeding 250 mg/100 ml in sodium are classified as “high in sodium” or “unhealthy.”

#### 2.1.4. Free sugar thresholds

Foods exceeding 10 g/100 g of free sugars are classified as “high in sugar” or “unhealthy.”

Beverages or liquids exceeding 5 g/100 ml of free sugars are classified as “high in sugar” or “unhealthy.”

#### 2.1.5. Model 2

**FIGURE 1:**
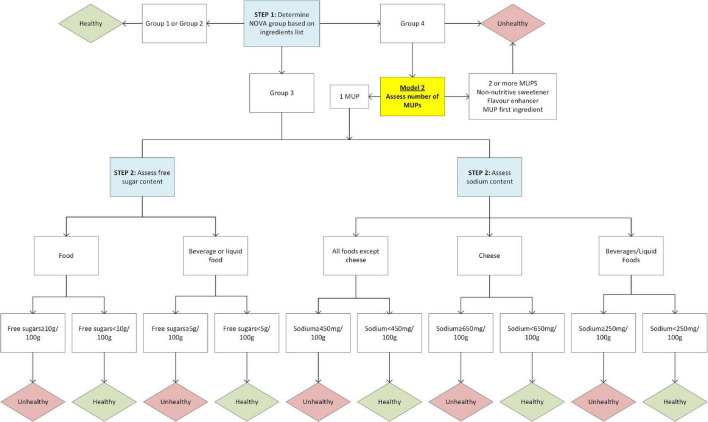
Flow diagram for classification of Model 1 and Model 2 (variation for Model 2 highlighted yellow).

The use of markers of ultra-processing (MUPs) (i.e., processed food substances and cosmetic additives) to identify ultra-processed foods is a simple and effective way to capture the concept (43, 44). The MUPs term was first coined by Davidou et al. when developing the SIGA classification scheme (43, 44). Ultra-processed foods are defined as “*formulations of ingredients, mostly of exclusive industrial use, that result from a series of industrial processes*” (9), and MUPs have been used as proxies to identify food ultra-processing. Another common characteristic of ultra-processed foods is the low presence or absence of intact whole foods. This is a difficult dimension to metricise considering the limited information about ultra-processing techniques available on food labelling. For the purpose of using the NOVA system to inform the development of this scheme, we assumed that when only one MUP is used, most of the food matrix of wholefoods is preserved or the product might not be a “*formulation of ingredients.*” In our previous research we found a small number of examples of these food and beverage products, such as cheeses, yoghurts, and breads. Therefore, a second version of the model (Model 2) was developed and tested. Model 2 follows the same criteria as Model 1, except the ultra-processed group is divided into sub-groups (group 4.1: foods contain only one MUP, group 4.2: foods contain more than one MUP). The division of the ultra-processed group by number of MUPs is a technical approach first applied in the Siga classification scheme (43). Food items falling into sub-group 4.1 will undergo further assessment of free sugars and sodium as for NOVA group 3 foods before being classified as healthy or unhealthy. Sub-group 4.2 will automatically be classified as unhealthy. To ensure no substitutions can occur for free sugars or sodium, aligning with requirement 4, foods with any non-nutritive sweeteners or flavour enhancers, regardless of number of MUPs, will automatically be classified as sub-group 4.2 and unhealthy. It is noted here that other ingredients can contain sodium, such as sodium stearoyl-2-lactylate which is used as an emulsifier, but these have not been considered as salt substitutes for this analysis. Furthermore, if the MUP is listed as the first ingredient (or second ingredient after water) and therefore making up the highest proportion of the food, the product is also automatically classified as sub-group 4.2. Model 2 does not consider the type of MUP, apart from the exceptions above, although type of MUP may be an important consideration in any further iterations. Figure 1 presents a diagram outlining the steps involved in models 1 and 2.

### 2.2. Validity measures

The literature on validation of NCSs focuses specifically on nutrient profiling models (45–48). Many different types of validation have been recommended and applied, including content, face, criterion, construct, convergent, discriminant, and predictive validity. The definitions and applications of the measures of validity are inconsistent throughout the research (47), therefore there is no clear recommendation on the type of validation that should be conducted on NCSs. For this research the authors have chosen to test two types of validity that are appropriate for a new model and have been applied in similar analyses (47, 49), face validity and convergent validity. Face validity is how accurately a test or model measures what it intends to measure “on the face of it,” usually from the point of view of the end user (45, 50). Although a simple validation method, a lack of face validity could mean a lack of confidence in the effectiveness of the model by end users (e.g., consumers at the point of purchase for FOPLs) (50). Convergent validity is the extent to which a measure aligns with a closely related one and is recognised to be an important step in the validation of NCSs (45–47).

### 2.3. Data collection

A dataset was assembled combining food and nutrient data from the Australian Food Composition Database AUSNUT 2011–2013 (51), and Mintel’s Global New Product Database (2014–2019) (Mintel) (52). The combination of databases ensured a wide range of products in the Australian food supply would be represented, from fresh unpackaged vegetables to novel processed packaged products. The dataset provides examples of food items typically available or newly launched in the Australian food supply between 2011 and 2019. Details of the assembly of the dataset have been described previously (15).

### 2.4. Data analysis

Face validity was assessed by applying the models to the Australian dataset and judging the resulting classifications over different categories and individual products. Items in the dataset were classified by models 1 and 2. First, all items were classified by NOVA: group 1 unprocessed or minimally processed foods, group 2 processed culinary ingredients, group 3 processed foods, and group 4 ultra-processed foods. Mintel data was classified by examining ingredients lists, guided by the most recent published descriptions of the NOVA groups (9). Ultra-processed foods were classified based on the presence of processed food substances or cosmetic additives (MUPs). The MUP status of novel or ambiguous ingredients was discussed amongst all authors until a consensus was reached. A list of ingredients considered MUPs in this research is provided in Supplementary Tables 1, 2. Items in AUSNUT have been previously classified by NOVA by Machado et al. (53).

All items classified as ultra-processed were then categorised into sub-groups: group 4.1 (containing only one MUP), or group 4.2 (containing more than one MUP, a non-nutritive sweetener or flavour enhancer, or a MUP in the first position of the ingredients list). Number of MUPs had to be estimated for items in AUSNUT as manufacturer’s ingredients lists are not provided for commercial items in the database. A comprehensive and robust methodology was developed to estimate MUPs for AUSNUT and is provided in Supplementary material 1. Sodium content was available for all items in both Mintel and AUSNUT, however free sugar content was only provided in AUSNUT. Free sugar content was estimated for the Mintel data using a method proposed in the guide to the PAHO NPM (23). Free sugars were defined using the WHO definition (54). All items in the combined dataset were categorised as either solid foods, liquids (beverages or soups), or cheeses. For analysis purposes, all products were classified into a food grouping system adapted from the Global Food Monitoring Group food categorisation system (55). Finally, a syntax was created in Stata version 17 to categorise all items as either healthy or unhealthy by Model 1 and Model 2 following the logic in Figure 1. A complete list of classifications for models 1 and 2 for every food and beverage item in the dataset was produced, and items considered potential anomalies (from the opinion of the researchers/public health nutritionists) were identified.

To assess convergent validity the model NCSs were compared to other similar measures of healthiness. Thus, the combined dataset was also classified by the PAHO NPM (23), the HSR (56), and a binary version of the ADGs, wherein foods are classified as recommended five food group (FFG) foods or discretionary foods (57). The PAHO NPM was chosen as it operates from a similar logic as this NCS proposal, classifying foods by NOVA and overlaying nutrient criteria, albeit with some key differences. The PAHO NPM was included to ascertain if limitations identified in previous research had been resolved with these models. The HSR informs currently implemented policy actions in Australia, and the ADGs are a key tool informing nutrition policy in Australia. All items in the combined dataset have been classified by the PAHO NPM, the HSR, and the ADGs in previous research, and details on classification methodology are described elsewhere (15).

### 2.5. Statistical analysis

All statistical analyses were conducted using Stata version 17 (58). The frequency of food items classified as healthy and unhealthy for the total sample, by NOVA category, and by food category were produced for both models. The classification differences between Model 1 and Model 2 were examined by producing the frequency of food items that moved from the unhealthy category to the healthy category for Model 2, by food category. The frequency of food items exceeding sodium and free sugar thresholds for NOVA group 3 and sub-group 4.1 were produced by food category and sub-category, and as a proportion of the total number of food items in each food category and sub-category.

The proportion of food items classified as unhealthy by both models was compared to that of NOVA, the PAHO NPM, the HSR, and the ADGs. The scaled ratings of the HSR were grouped into a binary output for comparison purposes. As there is no agreed cut-off in which food items are defined as “healthy” on the HSR scale, both 2.5 stars and 3.5 stars were used as cut-off points for healthy foods. Only ultra-processed foods were classified as unhealthy for NOVA, and discretionary foods as unhealthy for the ADGs. The PAHO NPM binary output clearly identifies a category as “unhealthy.” One of the major differences between the PAHO NPM and the developed models was the exclusion of total fat and saturated fat as nutrient criteria. To examine the result of this difference, the frequency of NOVA group 3 food items exceeding total fat and saturated fat for the PAHO NPM (and thus classified as unhealthy) but classified as healthy for models 1 and 2 (as fats are not assessed) were produced, by subcategory.

The Cohen’s Kappa agreement coefficient and percentage agreement was calculated for each pairing between both models and the comparison NCSs. The Kappa coefficient was interpreted using Landis and Koch (59): below 0.0–poor, 0.00–0.20–slight, 0.21–0.40–fair, 0.41–0.60–moderate, 0.61–0.80–substantial, and 0.81–1.00–almost perfect.

## 3. Results

A total of 7,322 food items were assessed in the combined dataset, 3,002 from AUSNUT and 4,320 from Mintel (Table 3). Models 1 and 2 classified the majority of items as unhealthy, 73.3 and 68.2%, respectively. Model 1 classified 1,958 items as healthy, of which 1,447 were NOVA group 1 or 2 foods and ingredients, and 511 were NOVA group 3 foods; and 5,364 as unhealthy, of which 570 were NOVA group 3 foods, and 4,794 NOVA group 4 foods (total number of ultra-processed items in the dataset). Model 2 differed by the number of MUPs used to identify healthy/unhealthy foods. For Model 2, 790 (16.5%) of NOVA group 4 foods were classified into sub-group 4.1 (contained only one MUP), and of these, 367 (46.5%) moved to the healthy category (representing only 7.7% of all NOVA group 4 foods).

**TABLE 3:** Frequency and proportion of food items classified as healthy and unhealthy for each model by groups.

		Model 1		Model 2	
	**Total**	**Healthy**	**Unhealthy**	**Healthy**	**Unhealthy**
All groups	7,322	1,958 (26.7%)	5,364 (73.3%)	2,325 (31.8%)	4,997 (68.2%)
NOVA groups 1 and 2	1,447	1447 (100%)	0	1447 (100%)	0
NOVA group 3	1,081	511 (47.3%)	570 (52.7%)	511 (47.3%)	570 (52.7%)
NOVA group 4	4,794	0	4,794 (100%)	367 (7.7%)	4,427 (91.3%)
Sub-group 4.1	790	-	790 (100%)	367 (47.1%)	418 (52.9%)
Sub-group 4.2	4,009	-	4,009 (100%)	0	4,009 (100%)

**FIGURE 2:**
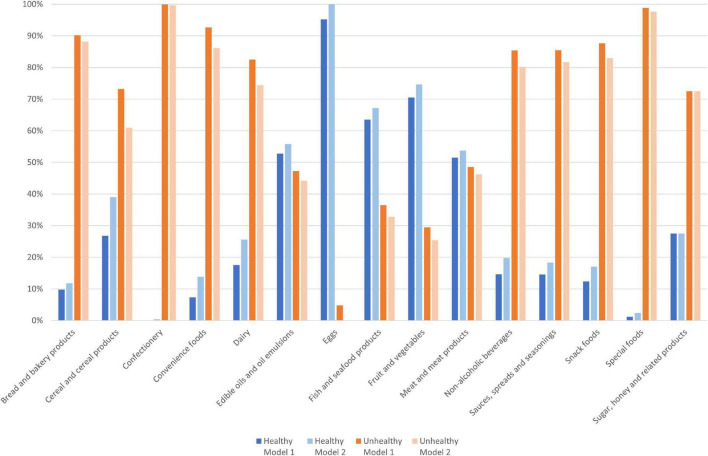
Proportion of food items (*n* = 7,322) classified as healthy and unhealthy by Model 1 and Model 2, by category.

Face validity was assessed by evaluating the resulting healthy/unhealthy classifications within different food categories and over individual products. The majority of items in the categories of eggs, fish and seafood products, fruit and vegetables, and meat and meat products, were classified as healthy for both models (Figure 2). Over 50% of food items were classified as unhealthy in the food categories of: bread and bakery products; cereal and cereal products; confectionery; convenience foods; dairy; non-alcoholic beverages; sauces, seasonings, and spreads; snack foods; special foods; and sugar, honey, and related products. Of the 367 sub-group 4.1 food items that moved to the healthy category for Model 2, the highest number were in the cereal and cereal products category (*n* = 78), the dairy category (*n* = 75), and the convenience food category (*n* = 51) (Supplementary Figure 1).

A complete list of the classifications for the 7,322 food and beverage items for models 1 and 2 is presented in Supplementary material 2, along with a list of the sub-group 4.1 items classified as healthy, and 99 “healthy” classifications that were considered anomalies. Examples include frozen puff pastry sheets; high fat meals (e.g., “Mac and cheese croquettes” and “Fettucine with chicken and cream sauce”); meals with processed meats as ingredients (e.g., “Ham and cheese quiche” and “One-pan brekky with beef chipolata sausages”); dairy- or coconut-based desserts (e.g., “Chocolate flavoured coconut milk mousse” and “dairy dessert, flavours other than chocolate”); pâtés (e.g., “Chicken and Madeira Pâté” and “Duck and Shiraz Pâté”); frozen potato products (e.g., “potato wedges, purchased frozen, deep fried or fried” and “potato gem, purchased frozen, baked or roasted”); mayonnaises (e.g., “Garlic Aioli Mayonnaise” and “Tartare Sauce”); and 2 alternative meat products composed largely of starches (“Lightly battered prawns” and “Lightly crumbed scallops”).

For NOVA group 3 items, a high percentage of pizzas (100%), processed meats (90.9%), breads (75%), soups (64.7%), crisps and snacks (49.2%), and cheeses (48.1%) exceeded sodium thresholds (Table 4). And a high percentage of NOVA group 3 spreads (64.8%), ice cream and ice edibles (80%), and fruit and vegetable juices (42.9%) exceeded free sugar thresholds. All NOVA group 3 cakes, muffins and pastries, confectionery, pizza, beverage mixes, cordials, and sugar products were classified as unhealthy. For sub-group 4.1 items, a high percentage of sauces (68.9%), processed meats (61.9%), cheeses (60.7%), and crisps and snacks (51.4%) exceeded sodium thresholds, and a high percentage of fruit and vegetable juices (79.2%), and nuts and seeds (63.6%), cakes, muffins and pastries (51.9%), and sauces (42.2%) exceeded free sugar thresholds (Table 5). Less than 15% of sub-group 4.1 noodles, pasta, pre-prepared salads and sandwiches, milk, and vegetables were classified as unhealthy by Model 2.

**TABLE 4:** Frequency of group 3 food items exceeding sodium and free sugar thresholds, and frequency and proportion classified as unhealthy, by category and subcategory.

Category	Subcategory	Exceeds sodium	Exceeds free sugars	Total unhealthy	Total G3	% G3 unhealthy
Bread and bakery products		125	11	132	174	75.9
	Biscuits	17	6	20	26	76.9
	Bread	102	0	102	136	75.0
	Cakes, muffins, and pastries	1	4	5	5	100.0
	Flours, breadcrumbs, and bread mixes	5	1	5	7	71.4
Cereal and cereal products		11	10	21	55	38.2
	Breakfast cereals	0	10	10	15	66.7
	Noodles	3	0	3	13	23.1
	Pasta	7	0	7	17	41.2
	Rice	1	0	1	7	14.3
Confectionery		0	5	5	5	100.0
Convenience foods		36	0	36	92	39.1
	Canapes	4	0	4	10	44.4
	Meal kits	4	0	4	10	40.0
	Pizza	14	0	14	14	100.0
	Ready meals	3	0	3	22	14.3
	Soup	11	0	11	17	64.7
Dairy		52	12	64	148	43.2
	Cheese	52	0	52	108	48.1
	Ice cream and edible ices	0	4	4	5	80.0
	Milk	0	8	8	22	36.4
Fish and seafood products		19	0	19	44	43.2
	Fresh or frozen fish and seafood	1	0	1	1	100.0
	Processed fish and seafood	18	0	18	43	41.9
Fruit and vegetables		53	48	95	212	44.8
	Fruits	14	32	46	97	47.4
	Herbs and spices	7	2	7	9	77.8
	Nuts and seeds	14	12	23	44	52.3
	Vegetables	18	2	19	62	30.6
Meat and meat products		44	0	44	65	67.7
	Fresh or frozen meat	1	0	1	2	50.0
	Meat alternatives	3	0	3	19	15.8
	Processed meat	40	0	40	44	90.9
Non-alcoholic beverages		1	14	14	35	40.0
	Beverage mixes	1	7	7	7	100.0
	Cordials	0	2	2	2	100.0
	Fruit and vegetable juices	0	3	3	7	42.9
	Soft drinks, iced teas, and kombuchas	0	2	2	13	15.4
Sauces, spreads, and seasonings		44	54	88	152	57.9
	Dips	9	1	10	29	34.5
	Mayonnaise and salad dressings	3	1	4	8	50.0
	Sauces	24	16	33	55	60.0
	Seasonings, recipe bases, and stocks	3	1	3	6	50.0
	Spreads	5	35	40	54	74.1
Snack foods		40	15	50	96	52.1
	Crisps and snacks	31	6	34	63	54.0
	Popcorn	5	2	5	6	83.3
	Snack mixes and bars	4	7	11	27	40.7
Sugar, honey, and related products		0	2	2	2	100.0
	Dessert additions	0	1	1	1	100.0
	Other sugar-based products	0	1	1	1	100.0

G3, NOVA group 3; G4.1, NOVA group 4 containing 1 marker of ultra-processing. Categories and subcategories with no products classified as unhealthy are not shown.

**TABLE 5:** Frequency of sub-group 4.1 products exceeding sodium and free sugar thresholds, and frequency and proportion classified as unhealthy, by category and subcategory.

Category	Subcategory	Exceeds sodium	Exceeds free sugars	Total unhealthy	Total G4.1	% G4.1 unhealthy
Bread and bakery products		36	20	53	67	79.1
	Savoury biscuits	14	0	14	21	66.7
	Bread	9	0	9	12	75.0
	Cakes, muffins, and pastries	11	14	24	27	88.9
	Sweet biscuits	2	6	6	6	100.0
Cereal and cereal products		14	22	35	113	31.0
	Breakfast cereals	9	20	28	61	45.9
	Cereal bars	0	2	2	2	100.0
	Noodles	1	0	1	9	11.1
	Pasta	3	0	3	24	12.5
	Quinoa and other cereals	1	0	1	3	33.3
Confectionery		1	9	9	10	90.00
Convenience foods		29	2	29	80	36.3
	Canapes	5	1	6	9	66.7
	Meal kits	7	1	8	14	57.1
	Pizza	10	0	10	11	90.9
	Pre-prepared salads and sandwiches	1	0	1	15	6.7
	Soup	6	0	6	7	85.7
Dairy		17	9	26	101	25.7
	Cheese	17	0	17	28	60.7
	Dairy desserts	0	2	2	8	25.0
	Ice cream and edible ices	0	5	5	6	83.3
	Milk	0	2	2	30	6.7
Fish and seafood products		8	1	8	15	53.3
	Processed fish and seafood	8	1	8	15	53.3
Fruit and vegetables		9	18	23	59	39.00
	Fruits	1	7	8	22	25.8
	Herbs and spices	6	3	6	6	100.0
	Nuts and seeds	0	7	7	11	63.6
	Vegetables	2	1	2	20	10.0
Meat and meat products		16	0	16	31	55.6
	Meat alternatives	3	0	3	10	30.00
	Processed meat	13	0	13	21	61.9
Non-alcoholic beverages		1	102	102	141	72.3
	Beverage mixes	1	10	10	14	71.4
	Coffee and tea	0	1	1	4	25.0
	Cordials	0	1	1	1	100.0
	Energy drinks	0	1	1	1	100.0
	Fruit and vegetable juices	0	80	80	101	79.2
	Soft drinks, iced teas, and kombuchas	0	9	9	13	69.2
Sauces, spreads, and seasonings		50	42	75	98	76.5
	Dips	7	0	7	16	43.8
	Mayonnaise and salad dressings	9	6	11	13	84.6
	Sauces	31	19	37	45	82.2
	Seasonings, recipe bases, and stocks	1	0	1	4	25.00
	Spreads	2	17	19	20	95.00
Snack foods		28	15	37	58	65.0
	Crisps and snacks	18	7	22	35	62.9
	Popcorn	4	0	4	5	80.0
	Snack mixes and bars	4	8	11	18	61.1
Sugar, honey, and related products		1	5	5	5	100.0
	Dessert additions	1	4	4	4	100.0
	Other sugar-based products	0	1	1	1	100.0

G3, NOVA group 3; G4.1, NOVA group 4 containing 1 marker of ultra-processing. Categories and subcategories with no products classified as unhealthy are not shown.

**FIGURE 3:**
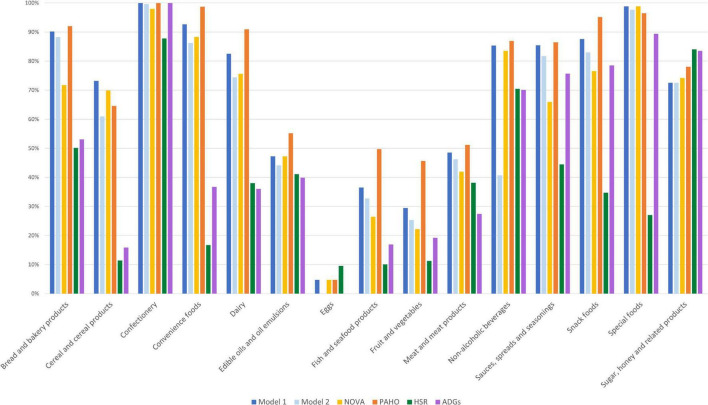
Proportion of products classified as unhealthy by category and nutrition classification schemes [Model 1, Model 2, NOVA, the Pan American Health Organization’s Nutrient Profile Model (PAHO), the Health Star Rating (HSR), and the Australian Dietary Guidelines (ADGs)].

For the assessment of convergent validity, most food categories in both Model 1 and Model 2 classified food items in closest alignment to NOVA (Figure 3). In all food categories, except for cereal products and special foods, the PAHO NPM classified more items as unhealthy compared to both models. This difference is explained by a high number of NOVA group 3 items in the subcategories of cheese, crisps and snacks, dips, nuts and seeds, and processed fish and seafood being classified as unhealthy by the PAHO NPM due to total fat and saturated fat criteria Supplementary Figure 2. Both models were fair to substantially aligned (although only at the 3.5 cut-off for the HSR) to all other NCS’s when pairwise agreement using Cohen’s Kappa was assessed (Table 6). Model 1 had highest agreement with NOVA, with almost perfect agreement. Model 2 had the highest pairwise agreement with the PAHO NPM, with substantial agreement. The HSR and ADGs had lower agreement with both models, but slightly higher agreement with Model 2 compared to Model 1.

**TABLE 6:** Pairwise agreement between Model 1 and Model 2, and NOVA, PAHO NPM, the ADGs, and the HSR using Cohen’s Kappa co-efficient and percentage agreement.

		Model 1 (73.3% unhealthy)		Model 2 (68.3% unhealthy)	
	**Unhealthy**	***K* (95% CI)**	**Agreement**	***K* (95% CI)**	**Agreement**
NOVA	65.6%	0.816 (0.801–0.829)	92.1%	0.709 (0.691–0.726)	87.1%
PAHO NPM	77.9%	0.773 (0.756–0.790)	91.6%	0.707 (0.689–0.725)	88.3%
ADGs	46.1%	0.358 (0.341–0.376)	67.8%	0.402 (0.383–0.420)	69.2%
HSR 2.5	37%	0.204 (0.187–0.220)	55.4%	0.252 (0.234–0.269)	59.0%
HSR 3.5	54%	0.372 (0.352–0.392)	69.8%	0.426 (0.406–0.447)	72.2%

K, Cohen’s Kappa coefficient; PAHO NPM, Pan American Health Organization’s nutrient profile model; ADGs, Australian dietary guidelines; HSR, health star rating. Landis and Koch interpretation of kappa (59): below 0.0–poor, 0.00–0.20–slight, 0.21–0.40–fair, 0.41–0.60–moderate, 0.61–0.80–substantial, and 0.81–1.00–almost perfect.

## 4. Discussion

This study aimed to develop a model NCS combining level of processing and nutrient thresholds, and to test the validity against items in the Australian food supply and against existing NCSs. The resulting two versions of the model combine the NOVA classification system with criteria for added sodium and free sugars, producing a binary output of either healthy or unhealthy. The intended application of the model NCSs is specific to policy purposes where a binary judgement of individual food items is required, for example front-of-package warning labels, restrictions to marketing of unhealthy food to children, taxes, or regulation of nutrition and health claims.

A higher proportion of food categories consistent with dietary patterns that are associated with positive health outcomes and included as recommended in most national dietary guidelines (60, 61), such as fruits, vegetables, and eggs were classified as healthy. And the clear majority of food categories consistent with dietary patterns associated with adverse health outcomes and not recommended in national dietary guidelines, such as confectionery, snack foods, and convenience foods were classified as unhealthy. Therefore, the models produced health assessments of food items reflective of current nutrition evidence when applied to the Australian food supply, suggesting good face validity.

The added free sugar and sodium thresholds set for the models reliably identified NOVA group 3 food items that are typically high in added sugars and salt, for example, processed meats are not usually classified as ultra-processed as the additives used have a preservation purpose. However, 91% (40/44) of the NOVA group 3 processed meats in the dataset exceeded the sodium threshold and were therefore classified as unhealthy. Only a modest number of products in the muffins, cakes and pastries, confectionery, pizza, cordials, and dessert addition sub-categories were classified as NOVA group 3 (most were NOVA group 4 ultra-processed), however 100% exceeded the added sodium or free sugar thresholds. Furthermore, over 75% of biscuits, ice creams and edible ices, and popcorn exceeded nutrient thresholds. A high percentage of NOVA group 3 breads, cheeses, and soups exceeded the sodium threshold. Although usually encouraged in dietary guidelines, there is wide variability in the sodium content of these foods, and those in the higher range can contribute significantly to dietary intake. In Australia, 15% of average daily sodium intake is derived from bread products alone (31). NOVA group 1 fruit juices are automatically classified as healthy, despite containing a significant amount of free sugars, thus juice products might need to be considered as an exemption to this criterion in the model’s application to policy.

Two versions of the model were tested in this study, differing in how they treat NOVA group 4 (ultra-processed) foods. Model 1 automatically classifies all ultra-processed foods as unhealthy, whereas Model 2 considers the presence of only 1 MUP to be healthy unless the added sodium and free sugar content exceeds thresholds. The difference this made to the types of products classified as healthy and unhealthy was explored, with particular interest in those ultra-processed foods moving to the healthy classification in Model 2. Of the 4,794 ultra-processed foods in the dataset, 16.5% contained only one MUP (sub-group 4.1), and of these 47.1% were under the set thresholds for sodium and free sugars. This resulted in only 7.7% (*n* = 367) of items in the dataset moving to the healthy classification for Model 2. Over half of these food items were cereals, dairy, or convenience foods. Common examples of items include gluten-free pastas, grated cheeses containing anti-caking agents, or ready-to-eat or ready-to-heat packaged meals, consisting of mostly a combination of whole foods such as meats and vegetables.

The NOVA system was developed to assess diet quality at the population level, and the recommendation is to limit ultra-processed foods as a group to achieve diets with low content of these foods. Therefore, the application of the NOVA system at the individual food-level for regulatory purposes presents some challenges. Although there is no evidence on how the number of markers of ultra-processed foods affect health, we assumed that most of the food matrix of wholefoods are preserved when only one marker is used. In fact, only a small number of products were assessed as such, and our findings indicate that the approach of Model 2 could be a suitable solution to this issue when classifying foods as unhealthy for these particular policy purposes. However, the importance of the number and type of MUP to the classification of unhealthy needs to be further investigated. For example, the presence of ingredients such as colours and flavours (purely cosmetic and unnecessary) and emulsifiers [associated with changes in microbiota (62)] could be considered to also automatically classify a food as unhealthy. Furthermore, the accurate classification of ultra-processed foods is currently limited by the lack of information provided by manufacturers or regulated on labelling regarding the specific purpose/function of an industrial food substance in a food, and the industrial processing techniques or processing aids used.

Convergent validity, the extent to which a measure aligns with a closely related one, is recognised to be an important step in the validation of NCSs (45, 46). Agreement with NOVA was almost perfect (according to the Kappa coefficient) for Model 1 and substantial for Model 2, indicating only modest modification to and departure from the original holistic concept on which they are based. Substantial agreement of both models with the PAHO NPM is also a predictable outcome considering they both combine level of processing and nutrient criteria. Much lower agreement was observed for the ADGs and the HSR, particularly for the HSR when a 2.5-star rating was applied as the cut off for identifying a healthy food. The current ADGs do not incorporate the concept of level of processing into the description of discretionary foods, hence the low agreement observed was expected. However, moderate agreement with the ADGs still indicates broad alignment with the evidence base underlying this key Australian policy tool. The fair to moderate agreement (depending on what cut-off is applied) with the HSR is further evidence of the low alignment of nutrient-profiling-only approaches and the NOVA concept (6).

A key difference between the tested models and the PAHO NPM is exclusion of the fat criteria. This difference was most apparent in the cheese category, with the PAHO NPM classifying 106 of the 108 NOVA group 3 cheese items as unhealthy due to excess total or saturated fat (Supplementary Figure 2). Over half of these items were classified as healthy by Models 1 and 2, examples of which included cottage cheese, reduced fat cheddar, and buffalo mozzarella. Other food examples with misalignment were firm tofu, soy milk, seedy crackers, roasted cashews, and unflavoured salmon canned in water, all classified as unhealthy by the PAHO NPM on the basis of total fat/saturated fat, but healthy by Models 1 and 2 (Supplementary Table 3). These examples are all recommended nutritious five food group foods according to the ADGs, and not foods that would need to be targeted as “unhealthy” for policy actions. However, other examples of NOVA group 3 foods with a higher fat content classified as healthy by the tested models were not always clearly healthier. For example, tartare sauce, beetroot chips, cashew cream cheese, coconut yoghurt with raw cacao, black truffle duck paté, and fettucine with chicken and cream sauce (Supplementary material 2). On balance however the exclusion of fats criteria in Models 1 and 2 appears to overcome the PAHO NPM’s limitation of penalising whole foods, and more accurately reflects the evidence base on the food-specific effects of fats (39).

This is not the first time the NOVA concept has been incorporated into a NCS in combination with nutrient profiling. As discussed, the PAHO NPM presents a strong example of this approach and is already applied for policy purposes (e.g., informs labelling schemes in Mexico and Argentina). Although in contrast to Models 1 and 2, it assesses the nutrient content of all NOVA group 3 and group 4 foods, meaning manufacturers of ultra-processed foods are still able to manipulate nutrient content to avoid an unhealthy classification (63). The inclusion of % energy and fat criteria in the PAHO NPM also leads to a limited interpretation of what can be classified as a healthy food. The Siga scheme, developed and applied for commercial purposes, also employs a top-down approach, applying NOVA categories before overlaying nutrient criteria (43). However, the complexity of the system, which considers risk assessment of additives and the application of industrial processing techniques, may not be practical for policy purposes, potentially being overly burdensome on manufacturers, enforcement agencies, policy makers, and regulators. The Food Compass scheme, developed to guide consumer behaviour, food policy, scientific research, and industry reformulations (49), includes the NOVA classification as a minor criterion, yet also involves an impractical level of complexity, incorporating a range of vitamins, minerals, and phytonutrients that are rarely provided in the nutrition information panel.

The binary classification of the developed models results in less flexibility regarding foods and beverages in the “grey zone” of healthiness. For example, Food Compass and the HSR are ranking scales that enable foods to sit at a mid-point for healthiness. However, nutrition policy actions require a method to unambiguously identify unhealthy foods, the regulation of which will likely result in the greatest improvement to food environments (20, 64). The application of the models to policies such as restrictions to marketing is relatively straightforward, as foods classified as unhealthy will not be eligible for the specific marketing activity. The models could be applied to a warning style FOPL by labelling unhealthy products with one or more of three statements, based on the criteria on which the product failed, i.e., “Ultra-processed food,” “High in free sugars,” and/or “High in sodium” (65). Given the diversity of foods in the marketplace, no NCS will always correctly identify healthy and unhealthy foods in absolute terms. However, an underlying principle in the development of the models is that it is preferable, in policy terms, to favour the most accurate classification of unhealthy foods so that healthy foods are not incorrectly identified for regulation. Any anomalies that may occur are most likely to represent potentially unhealthy foods as healthy (e.g., some dairy desserts, high-fat meals, crumbed fish, and puff pastry). These anomalies are minimal, and the actual foods would not be labelled as healthy but would just not be subject to taxation, warning labels or restrictions to marketing.

Potential difficulties that could be encountered in the model’s use are determining MUPs and added free sugar content. A standard list of ingredients considered MUPs would need to be developed and updated regularly for any new or novel products, specific to the national food supply. The reporting of added free sugars is not a requirement on the nutrition information panel in Australia and many other countries, and thus the free sugar content needs to be estimated in most jurisdictions. A standard estimation approach was used in this study, however, for greater accuracy food regulations would need to be modified to include free sugars. The simplicity of the model however enables the upper nutrient thresholds and the categories they apply to (cheeses, foods, and liquids) to be adjusted based on the specific policy application and national context.

The development of the model NCS was based on a strong foundation of principles evolved through rigorous analysis of existing schemes (15). The models were applied to a large dataset comprising a diverse range of food and beverage products available in the Australian food supply and potentially subject to regulation. Convergent validity was also rigorously assessed against four existing NCSs with differing conceptual bases. Nevertheless, some limitations in the analysis should be acknowledged. Some variables were estimated, including free sugars, and the number of MUPs (for AUSNUT data) for the model NCS, and fibre and fruit/vegetable/nut/legume content for existing NCSs. However, robust procedures were developed and recorded for each estimation. Our analysis only assesses the model NCS against the Australian food supply, product types available in other marketplaces may produce different results, thus assessment against national food supplies would be recommended and any adjustments made before application to policy. The validation of a NCS should also involve predictive validity, that is, the ability of the model to predict health outcomes, thus this should be prioritised as the next step.

This study found the incorporation of a top-down holistic approach to nutrition classification, considering degree of processing as a first step, produces a NCS that may prevent ultra-processed foods from being promoted or evading regulation. The novel NCS represents an improvement on existing models by avoiding an overly strict interpretation of what is considered an “unhealthy” food. This analysis also indicates strong face and convergent validity against existing NCSs. Thus, a model NCS combining level of processing and nutrient criteria presents a valid alternative to existing methods to classify the health potential of individual foods for policy purposes.

## Data availability statement

The data analyzed in this study is subject to the following licenses/restrictions: Restrictions apply to the availability of the data described in this manuscript, which were used under license from Mintel, and so are not publicly available. Data are, however, available from the authors upon reasonable request and with permission of Mintel. Requests to access these datasets should be directed to SD, s.dickie@deakin.edu.au.

## Author contributions

SD: conducted the research, analysed the data, and wrote the manuscript. ML, JW, and PM: provided supervision and reviewed and edited the manuscript. All authors contributed to the conception and design of the research and read and approved the final manuscript.

## References

[1] Afshin A Sur P Fay K Cornaby L Ferrara G Salama J Health effects of dietary risks in 195 countries, 1990–2017: a systematic analysis for the Global Burden of Disease Study 2017. *Lancet.* (2019) 393:1958–72. 30954305 10.1016/S0140-6736(19)30041-8 PMC6899507

[2] Food and Agricultural Organization of the United Nations, World Health Organization. *Sustainable Healthy Diets - Guiding Principles.* Rome: FAO (2019).

[3] World Health Organization. *Global Action Plan for the Prevention and Control of NCDs 2013-2020.* Geneva: WHO (2013).

[4] The World Health Organization. *Report of the the Commission on Ending Childhood Obesity.* Geneva: WHO (2016).

[5] World Cancer Research Fund International. *Nourishing framework: WCRF International.* London: World Cancer Research Fund International (2017).

[6] Dickie S Woods J Baker P Elizabeth L Lawrence M. Evaluating nutrient-based indices against food- and diet-based indices to assess the health potential of foods: how does the Australian health star rating system peform after five years. *Nutrients.* (2020) 12:1463. 10.3390/nu12051463 32443570 PMC7284529

[7] Romero Ferreiro C Lora Pablos D Gómez De La Cámara A. Two dimensions of nutritional value: nutri-score and NOVA. *Nutrients.* (2021) 13:2783. 10.3390/nu13082783 PMC8399905 34444941

[8] Monteiro C. All the harmful effects of ultra-processed foods are not captured by nutrient profiling. *Public Health Nutr.* (2009) 12:1968–9.

[9] Monteiro C Cannon G Levy R Moubarac J Louzada M Rauber F Ultra-processed foods: what they are and how to identify them. *Public Health Nutr.* (2019) 22:936–41. 30744710 10.1017/S1368980018003762 PMC10260459

[10] Monteiro C Cannon C Lawrence M Costa Louzada M Pereira Machado P. *Ultra-processed Foods, Diet Quality, and Health Using the NOVA Classification System.* Rome: FAO (2019).

[11] Lane M Davis J Beattie S Gomez-Donoso C Loughman A O’Neil A Ultraprocessed food and chronic noncommunicable diseases: a systematic review and meta-analysis of 43 observational studies. *Obes Rev.* (2021) 22:e13146. 10.1111/obr.13146 33167080

[12] Pagliai G Dinu M Madarena M Bonaccio M Iacoviello L Sofi F. Consumption of ultra-processed foods and health status: a systematic review and meta-analysis. *Br J Nutr.* (2021) 125:308–18. 32792031 10.1017/S0007114520002688 PMC7844609

[13] Anastasiou K Baker P Hadjikakou M Hendrie G Lawrence MA. conceptual framework for understanding the environmental impacts of ultra-processed foods and implications for sustainable food systems. *J Clean Product.* (2022) 368:133155.

[14] Garzillo J Poli V Leite F Steele E Machado P Louzada M Ultra-processed food intake and diet carbon and water footprints: a national study in Brazil. *Rev Saúde Pública.* (2022) 56:6. 10.11606/s1518-8787.2022056004551 35239844 PMC8859933

[15] Dickie S Woods J Machado P Lawrence M. Nutrition classification schemes for informing nutrition policy in Australia: nutrient-based, food-based, or dietary-based? *Curr Dev Nutr.* (2022) 6:nzac112. 10.1093/cdn/nzac112 PMC9429971 36060220

[16] Barabási A Menichetti G Loscalzo J. The unmapped chemical complexity of our diet. *Nat Food.* (2020) 1:33–7.

[17] Fardet A Rock E. Toward a new philosophy of preventive nutrition: from a reductionist to a holistic paradigm to improve nutritional recommendations. *Adv Nutr.* (2014) 5:430–46. 10.3945/an.114.006122 25022992 PMC4085191

[18] Codex Alimentarius Commission. *Joint FAO/WHO food Standards Programme Codex Committee Meeting on Food Labelling, 46th Session, 27 September-1 October and 7 October 2021: Proposed Draft Guidelines on Front-of-pack Nutrition Labelling.* Rome: Food and Agriculture Organization of the United Nations and World Health Organization (2021).

[19] European Commission. *Report from the Commission to the European Parliament and the Council Regarding the use of Additional Forms of Expression and Presentation of the Nutrition Declaration.* Brussels: European Commission (2020).

[20] Khandpur N Monteiro C Swinburn B. Nutrient-Based warning labels may help in the pursuit of healthy diets. *Obesity.* (2018) 26:1670–1. 10.1002/oby.22318 30358147

[21] Moubarac J Parra D Cannon G Monteiro C. Food classification systems based on food processing: significance and implications for policies and actions: a systematic literature review and assessment. *Curr Obes Rep.* (2014) 3:256–72. 10.1007/s13679-014-0092-0 26626606

[22] Ministry of Health of Brazil. *Dietary Guidelines for the Brazilian Population.* Brazil: Ministry of Health of Brazil (2014).

[23] PAN American Health Organization. *PAHO Nutrient Profiling Model.* Washington, DC: PAHO (2017).

[24] Te Morenga L Mallard S Mann J. Dietary sugars and body weight: systematic review and meta-analyses of randomised controlled trials and cohort studies. *BMJ.* (2012) 346:e7492. 10.1136/bmj.e7492 23321486

[25] Anderson C Curzon M Van Loveren C Tatsi C Duggal M. Sucrose and dental caries: a review of the evidence. *Obes Rev.* (2009) 10:41–54. 19207535 10.1111/j.1467-789X.2008.00564.x

[26] Aburto N Ziolkovska A Hooper L Elliott P Cappuccio F Meerpohl J. Effect of lower sodium intake on health: systematic review and meta-analyses. *BMJ.* (2013) 346:f1326. 10.1136/bmj.f1326 PMC4816261 23558163

[27] Mozaffarian D Fahimi S Singh G Micha R Khatibzadeh S Engell R Global sodium consumption and death from cardiovascular causes. *New Engl J Med.* (2014) 371:624–34. 25119608 10.1056/NEJMoa1304127

[28] The World Health Organization. *Guideline: Sodium intake for Adults and Children.* Geneva: WHO (2012). 23658998

[29] Powles J Fahimi S Micha R Khatibzadeh S Shi P Ezzati M Global, regional and national sodium intakes in 1990 and 2010: a systematic analysis of 24 h urinary sodium excretion and dietary surveys worldwide. *BMJ Open.* (2013) 3:e003733. 10.1136/bmjopen-2013-003733 24366578 PMC3884590

[30] Council NHaMR. *Nutrient Reference Values for Australia and New Zealand: Sodium Canberra.* Canberra, ACT: NHMRC (2017).

[31] Australian Bureau of Statistics. *4364.0.55.007 - Australian Health Survey: Nutrition First Results - Foods and Nutrients, 2011-2012.* Canberra, ACT: Australian Bureau of Statistics (2014).

[32] Yang Q Zhang Z Gregg E Flanders W Merritt R Hu F. Added sugar intake and cardiovascular diseases mortality among US adults. *JAMA Int Med.* (2014) 174:516. 10.1001/jamainternmed.2013.13563 PMC10910551 24493081

[33] Moynihan P Kelly S. Effect on caries of restricting sugars intake. *J Dental Res.* (2014) 93:8–18. 10.1177/0022034513508954 PMC3872848 24323509

[34] The World Health Organization. *Guideline: Sugars Intake for Adults and Children.* Geneva: WHO (2015). 25905159

[35] Newens K Walton JA. review of sugar consumption from nationally representative dietary surveys across the world. *J Hum Nutr Dietet.* (2016) 29:225–40. 10.1111/jhn.12338 26453428 PMC5057348

[36] The Australian Bureau of Statistics. *4364.0.55.011 - Australian Health Survey: Consumption of Added Sugars, 2011-2012.* Canberra, ACT: The Australian Bureau of Statistics (2016).

[37] Teegala S Willett W Mozaffarian D. Consumption and health effects of trans fatty acids: a review. *J Aoac Int.* (2009) 92:1250–7. 19916363

[38] Astrup A Magkos F Bier Dennis M Brenna J de Oliveira Otto Marcia C Hill James O Saturated fats and health: a reassessment and proposal for food-based recommendations. *J Am College Cardiol.* (2020) 76:844–57. 10.1016/j.jacc.2020.05.077 32562735

[39] De Oliveira Otto M Mozaffarian D Kromhout D Bertoni A Sibley C Jacobs D Dietary intake of saturated fat by food source and incident cardiovascular disease: the multi-ethnic study of atherosclerosis. *Am J Clin Nutr.* (2012) 96:397–404. 22760560 10.3945/ajcn.112.037770 PMC3396447

[40] Micha R Mozaffarian D. Saturated fat and cardiometabolic risk factors, coronary heart disease, stroke, and diabetes: a fresh look at the evidence. *Lipids.* (2010) 45:893–5. 10.1007/s11745-010-3393-4 20354806 PMC2950931

[41] Mozaffarian D. Dairy foods, obesity, and metabolic health: the role of the food matrix compared with single nutrients. *Adv Nutr.* (2019) 10:917S–23S. 10.1093/advances/nmz053 31518410 PMC6743828

[42] Rolls B. Dietary energy density: applying behavioural science to weight management. *Nutr Bull.* (2017) 42:246–53. 29151813 10.1111/nbu.12280 PMC5687574

[43] Davidou S Christodoulou A Fardet A Frank K. The holistico-reductionist Siga classification according to the degree of food processing: an evaluation of ultra-processed foods in French supermarkets. *Food Funct.* (2020) 11:2026–39. 10.1039/c9fo02271f 32083627

[44] Davidou S Christodoulou A Frank K Fardet A. A study of ultra-processing marker profiles in 22,028 packaged ultra-processed foods using the Siga classification. *J Food Comp Anal.* (2021) 99:103848. 10.1016/j.jfca.2021.103848

[45] Townsend M. Where is the science? what will it take to show that nutrient profiling systems work? *Am J Clin Nutr.* (2010) 91:1109S–15S. 10.3945/ajcn.2010.28450F 20164310

[46] Arambepola C Scarborough P Rayner M. Validating a nutrient profile model. *Public Health Nutr.* (2008) 11:371–8. 17605841 10.1017/S1368980007000377

[47] Poon T Labonté M Mulligan C Ahmed M Dickinson K L’Abbé M. Comparison of nutrient profiling models for assessing the nutritional quality of foods: a validation study. *Br J Nutr.* (2018) 120:567–82. 10.1017/S0007114518001575 30015603 PMC6137431

[48] Cooper S Pelly F Lowe J. Construct and criterion-related validation of nutrient profiling models: a systematic review of the literature. *Appetite.* (2016) 100:26–40. 10.1016/j.appet.2016.02.001 26850312

[49] Mozaffarian D El-Abbadi N O’Hearn M Erndt-Marino J Masters W Jacques P Food Compass is a nutrient profiling system using expanded characteristics for assessing healthfulness of foods. *Nat Food.* (2021) 2:809–18. 37117986 10.1038/s43016-021-00381-y

[50] Cohen R Swerdlik M. *Psychologocial Testing and Assessment: an Introduction to Test and Measurement.* Ninth ed. New York, NY: Mcgraw-Hill Education (2018).

[51] Food Standards Australia New Zealand. *AUSNUT 2011-2013 - Food Composition Database.* New Zealand: Food Standards Australia New Zealand (2014).

[52] Mintel. *GNPD - Global New Products Database.* London: Mintel Group Ltd. (2017).

[53] Machado P Steele E Levy R Sui Z Rangan A Woods J Ultra-processed foods and recommended intake levels of nutrients linked to non-communicable diseases in Australia: evidence from a nationally representative cross-sectional study. *BMJ Open.* (2019) 9:12. 10.1136/bmjopen-2019-029544 31462476 PMC6720475

[54] World Health Organization. *Reducing free Sugars Intake in Children to Reduce the Risk of Noncommunicable Diseases.* Geneva: WHO (2019).

[55] Dunford E Webster J Blanco Metzler A Czernichow S Ni Mhurchu C Wolmarans P International collaborative project to compare and monitor the nutritional composition of processed foods. *Eur J Preventative Cardiol.* (2012) 19:1326–32. 10.1177/1741826711425777 21971487

[56] Department of Health. *Health Star Rating System: Calculator and Style Guide.* Canberra, ACT: Department of Health (2020).

[57] National Health and Medical Research Council. *Australian Dietary Guidelines.* Canberra, ACT: National Health and Medical Research Council (2013).

[58] StataCorp. *Stata Statistical Software: Release 16.* College Station, TX: StataCorp LLC (2019).

[59] Landis J Koch G. The measurement of observer agreement for categorical data. *Biometrics.* (1977) 33:159–74. 843571

[60] Mozaffarian D. Dietary and policy priorities for cardiovascular disease, diabetes, and obesity. *Circulation.* (2016) 133:187–225. 26746178 10.1161/CIRCULATIONAHA.115.018585 PMC4814348

[61] Herforth A Arimond M Álvarez-Sánchez C Coates J Christianson K Muehlhoff EA. Global review of food-based dietary guidelines. *Adv Nutr.* (2019) 10:590–605. 31041447 10.1093/advances/nmy130 PMC6628851

[62] Chassaing B Van de Wiele T De Bodt J Marzorati M Gewirtz A. Dietary emulsifiers directly alter human microbiota composition and gene expression ex vivo potentiating intestinal inflammation. *Gut.* (2017) 66:1414. 10.1136/gutjnl-2016-313099 PMC5940336 28325746

[63] Dickie S Woods J Lawrence M. Analysing the use of the Australian health star rating system by level of food processing. *Int J Behav Nutr Phys Act.* (2018) 15:128. 10.1186/s12966-018-0760-7 PMC6293654 30545373

[64] Popkin B Barquera S Corvalan C Hofman K Monteiro C Ng S Towards unified and impactful policies to reduce ultra-processed food consumption and promote healthier eating. *Lancet Diab Endocrinol.* (2021) 9:462–70. 10.1016/S2213-8587(21)00078-4 33865500 PMC8217149

[65] Cotter T Kotov A Wang S Murukutla N. ‘Warning: ultra-processed’ — a call for warnings on foods that aren’t really foods. *BMJ Global Health.* (2021) 6:e007240. 10.1136/bmjgh-2021-007240 34933866 PMC8666852

