# Corrigendum: A novel food processing-based nutrition classification scheme for guiding policy actions applied to the Australian food supply

**DOI:** 10.3389/fnut.2023.1223316

**Published:** 2023-07-04

**Authors:** Sarah Dickie, Julie Woods, Priscila Machado, Mark Lawrence

**Affiliations:** School of Exercise and Nutrition Science, Institute for Physical Activity and Nutrition (IPAN), Deakin University, Geelong, VIC, Australia

**Keywords:** nutrition classification, food classification, NOVA, ultra-processed food, nutrient profiling, food policy, front-of-pack labelling (FOPL), food tax

In the published article, the references “Davidou S, Christodoulou A, Fardet A, Frank K. The holistico-reductionist Siga classification according to the degree of food processing: an evaluation of ultra-processed foods in French supermarkets. *Food Funct*. (2020) 11:2026–39. doi: 10.1039/c9fo02271f” and “Davidou S, Christodoulou A, Frank K, Fardet A. A study of ultra-processing marker profiles in 22,028 packaged ultra-processed foods using the Siga classification. *J Food Comp Anal*. (2021) 99:103848. doi: 10.1016/j.jfca.2021.103848” were not cited in the article. The citations have now been inserted as reference (43) and (44), in Section “*2.1. Model development*,” Sub-section “*2.1.5. Model 2*,” paragraph 1 and should read:

“The use of markers of ultra-processing (MUPs) (i.e., processed food substances and cosmetic additives) to identify ultra-processed foods is a simple and effective way to capture the concept (43, 44).”

In the published article, there was an error. Text was missing from the methods section.

A correction has been made to Section “*2.1. Model development*,” Sub-section “*2.1.5. Model 2*,” paragraph 1. This paragraph previously stated:

“The use of markers of ultra-processing (MUPs) (i.e., processed food substances and cosmetic additives) to identify ultra-processed foods is a simple and effective way to capture the concept. Ultra-processed foods are defined as “formulations of ingredients, mostly of exclusive industrial use, that result from a series of industrial processes” (9), and MUPs have been used as proxies to identify food ultra-processing. Another common characteristic of ultra-processed foods is the low presence or absence of intact whole foods. This is a difficult dimension to metricise considering the limited information about ultra-processing techniques available on food labelling. For the purpose of using the NOVA system to inform the development of this scheme, we assumed that when only one MUP is used, most of the food matrix of wholefoods is preserved or the product might not be a “formulation of ingredients.” In our previous research we found a small number of examples of these food and beverage products, such as cheeses, yoghurts, and breads. Therefore, a second version of the model (Model 2) was developed and tested. Model 2 follows the same criteria as Model 1, except the ultra-processed group is divided into sub-groups (group 4.1: foods contain only one MUP, group 4.2: foods contain more than one MUP).”

The corrected paragraph appears below:

“The use of markers of ultra-processing (MUPs) (i.e., processed food substances and cosmetic additives) to identify ultra-processed foods is a simple and effective way to capture the concept (43, 44). The MUPs term was first coined by Davidou et al. when developing the SIGA classification scheme (43, 44). Ultra-processed foods are defined as “formulations of ingredients, mostly of exclusive industrial use, that result from a series of industrial processes” (9), and MUPs have been used as proxies to identify food ultra-processing. Another common characteristic of ultra-processed foods is the low presence or absence of intact whole foods. This is a difficult dimension to metricise considering the limited information about ultra-processing techniques available on food labelling. For the purpose of using the NOVA system to inform the development of this scheme, we assumed that when only one MUP is used, most of the food matrix of wholefoods is preserved or the product might not be a “formulation of ingredients.” In our previous research we found a small number of examples of these food and beverage products, such as cheeses, yoghurts, and breads. Therefore, a second version of the model (Model 2) was developed and tested. Model 2 follows the same criteria as Model 1, except the ultra-processed group is divided into sub-groups (group 4.1: foods contain only one MUP, group 4.2: foods contain more than one MUP). The division of the ultra-processed group by number of MUPs is a technical approach first applied in the Siga classification scheme (43).”

The authors apologize for this error and state that this does not change the scientific conclusions of the article in any way. The original article has been updated.

