# Supplementary material for: A novel food processing-based nutrition classification scheme for guiding policy actions applied to the Australian food supply
Source: Front Nutr. 2023 Jan 20;10:1071356. doi: 10.3389/fnut.2023.1071356 (PMC9895835; doi:10.3389/fnut.2023.1071356)
Supplement: Supplementary file 1 [file Data_Sheet_1.docx]

**Supplementary Materials: File 1**

1. Supplementary Table 1. *List of ingredients considered MUPs*
2. Supplementary Table 2. *List of ingredients not considered MUPs*
3. Procedure for estimating number of MUPs in AUSNUT 2011-2013
4. Supplementary Figure 1. *Frequency of sub-group 4.1 items classified as healthy by category*
5. Supplementary Figure 2. *Frequency of NOVA group 3 items classified as unhealthy by PAHO due to total fat and saturated fat criteria by sub-category*
6. Supplementary Table 3. *Examples of food and beverage items for different classification scenarios, displaying product name and ingredient list*

**Supplementary Table 1. Ingredients identified as markers of ultra-processing (MUPs) in the Australian food supply for this research*.**

| **Markers of Ultra-processing** | **Purpose/reason** | **Exceptions** |
| --- | --- | --- |
| Maltodextrin | Bulking agent/thickener/sweetener/industrially derived substance/cosmetic additive |  |
| Dextrin | Emulsifier/thickener | Unless used as stabiliser |
| Wheat gluten | Bread improver |  |
| Bread improver | Changes texture or speed up process |  |
| Collagen | Industrial food substance |  |
| Humectant | Cosmetic additive – texture and moisture control | Unless used as a stabiliser or preservative |
| Glycerine/glycerin | Humectant, thickener |  |
| Glycerol | Humectant, thickener |  |
| Gelatin/gelatine | Gelling agent/emulsifier/thickener | Unless used as a stabiliser |
|  |  |  |
|  |  |  |
| Industrially derived sweeteners | Industrially derived sweetener |  |
| *Can be listed as:* | | |
| Glucose | Industrially derived sweetener |  |
| Glucose syrup | Industrially derived sweetener |  |
| Wheat glucose syrup | Industrially derived sweetener |  |
| Dried glucose syrup | Industrially derived sweetener |  |
| Fructose | Industrially derived sweetener |  |
| High fructose corn syrup | Industrially derived sweetener |  |
| Dextrose | Industrially derived sweetener |  |
| Golden syrup | Industrially derived sweetener |  |
| Invert sugar | Industrially derived sweetener |  |
| Agave syrup | Industrially derived sweetener |  |
| Rice malt syrup | Industrially derived sweetener |  |
| Corn syrup | Industrially derived sweetener |  |
|  |  |  |
| Non-nutritive sweeteners | Cosmetic additive |  |
| *Can be listed as:* | | |
| Natural sweetener | Non-nutritive sweetener |  |
| Sweetener | Non-nutritive sweetener |  |
| Malitol | Non-nutritive sweetener |  |
| Stevia | Non-nutritive sweetener |  |
| Stevia leaf extract | Non-nutritive sweetener |  |
| Steviol glycosides | Non-nutritive sweetener |  |
| Steviol | Non-nutritive sweetener |  |
| Erythritol | Non-nutritive sweetener |  |
| Acesulphame potassium | Non-nutritive sweetener |  |
| Aspartame | Non-nutritive sweetener |  |
| Sucralose | Non-nutritive sweetener |  |
| Thaumatin | Non-nutritive sweetener |  |
| Xylitol | Non-nutritive sweetener |  |
| Neotame | Non-nutritive sweetener |  |
| Mannitol | Non-nutritive sweetener/humectant |  |
|  |  |  |
| Gums | Gelling or thickening agent | Unless listed as a stabiliser |
| *Can be listed as:* | | |
| Vegetable gum | Gelling or thickening agent | Unless listed as a stabiliser |
| Xanthan gum | Gelling or thickening agent | Unless listed as a stabiliser |
| Locust bean gum | Gelling or thickening agent | Unless listed as a stabiliser |
| Natural gum | Gelling or thickening agent | Unless listed as a stabiliser |
| Guar gum | Gelling or thickening agent | Unless listed as a stabiliser |
| Pectin | Gelling agent | Unless listed as a stabiliser |
| Agar | Gelling agent | Unless listed as a stabiliser |
|  |  |  |
| Extracts | Cosmetic additives |  |
| *Can be listed as:* | | |
| Mustard extract | Flavour/cosmetic additive |  |
| Spice extract | Flavour/cosmetic additive |  |
| Herb extract | Flavour/cosmetic additive |  |
| Yeast extract | Flavour enhancer/cosmetic additive |  |
| Rosemary extract | Flavour/cosmetic additive |  |
| Aloe vera extract | Flavour/cosmetic additive |  |
| Tamarind extract | Flavour/cosmetic additive |  |
|  |  |  |
| Protein isolates |  |  |
| *Can be listed as:* | | |
| Whey | Protein isolate |  |
| Whey powder | Protein isolate |  |
| Whey protein | Protein isolate |  |
| Protein | Protein isolate |  |
| Milk protein | Protein isolate |  |
| Soy protein | Protein isolate |  |
| Hydrolyzed maize protein | Protein isolate |  |
| Hydrolyzed vegetable protein (hvp) | Protein isolate |  |
| Hydrolyzed soy protein | Protein isolate |  |
| Hydrolyzed plant protein | Protein isolate |  |
| Textured vegetable protein | Protein isolate |  |
| Pork protein | Protein isolate |  |
|  |  |  |
| Emulsifiers | Cosmetic additive/industrially derived food substance |  |
| *Can be listed as:* | | |
| Diphosphates | Emulsifier and bread improver |  |
| Distilled monoglyceride | Emulsifier |  |
| Mono and diglycerides | Emulsifier |  |
| Mono and diglycerides of fatty acids | Emulsifier |  |
| Polyglycerol polyricinoleate | Emulsifier |  |
| Soy lecithin | Emulsifier |  |
| Sunflower lecithin | Emulsifier |  |
|  |  |  |
| Thickeners | Cosmetic additive |  |
| *Can be listed as:* | | |
| Carrageenan | Thickener |  |
| Vegetable derived mono-glyceride | Thickener |  |
|  |  |  |
| Concentrates |  |  |
| *Can be listed as:* | | |
| Concentrated juice | Sweetener/industrial food substance |  |
| Juice concentrate | Sweetener/industrial food substance |  |
|  |  |  |
| Modified starches | Thickener/industrial derived food substance |  |
| *Can be listed as:* | | |
| Modified corn starch | Thickener/industrial derived food substance |  |
| Modified tapioca starch | Thickener/industrial derived food substance |  |
|  |  |  |
| Colours | Cosmetic additive |  |
| *Can be listed as:* | | |
| Natural colour | Cosmetic additive |  |
| Natural colour (carmine) | Colour/cosmetic additive |  |
| Natural colour (turmeric) | Colour/cosmetic additive |  |
| Natural colour (paprika) | Colour/cosmetic additive |  |
| Caramel (colour) | Colour/cosmetic additive |  |
| Caramel I | Colour/cosmetic additive |  |
| Tartarzine | Colour/cosmetic additive |  |
| Ferrous gluconate | Colour/cosmetic additive | Unless used as a stabiliser |
| Carmine | Colour/cosmetic additive |  |
| Annatto extracts | Colour/cosmetic additive |  |
| Beta-carotene | Colour/cosmetic additive | Unless used for vitamin fortification |
| Carotene | Colour/cosmetic additive |  |
| Paprika oleosin | Colour/cosmetic additive |  |
| Paprika extract | Colour/cosmetic additive |  |
| Turmeric oleoresin | Colour/cosmetic additive |  |
| Chlorophyll | Colour/cosmetic additive |  |
|  |  |  |
| Flavours | Cosmetic additive |  |
| *Can be listed as:* | | |
| Natural flavour | Cosmetic additive |  |
| Vanilla flavour | Cosmetic additive |  |
| Vanilla extract | Flavour/cosmetic additive |  |
| Barbeque flavour | Flavour/cosmetic additive |  |
| Vegetable extract | Flavour/cosmetic additive |  |
| Smoke flavour | Flavour/cosmetic additive |  |
| Natural flavour | Flavour/cosmetic additive |  |
| Natural chicken flavour | Flavour/cosmetic additive |  |
| Natural banana flavour | Flavour/cosmetic additive |  |
|  |  |  |
| Flavour enhancers |  |  |
| *Can be listed as:* | | |
| Msg | Flavour enhancer |  |
| Monosodium glutamate | Flavour enhancer |  |
| Monosodium L-glutamate | Flavour enhancer |  |
| Magnesium glutamate | Flavour enhancer |  |
| Monopotassium L-glutamate | Flavour enhancer |  |
| Maltol |  |  |
|  |  |  |
| Isolated fibres | Industrial food substance |  |
| *Can be listed as:* | | |
| Dietary fibre | Isolated fibre/industrial food substance |  |
| Fibre | Isolated fibre/industrial food substance |  |
| Wheat fibre | Isolated fibre/industrial food substance |  |
| Oat fibre | Isolated fibre/industrial food substance |  |
| Citrus fibre | Isolated fibre/industrial food substance |  |
| Inulin | Isolated fibre/industrial food substance |  |
| Polydextrose | Isolated fibre/industrial food substance |  |
| Potato fibre | Isolated fibre/industrial food substance |  |
| Barley beta glucans | Isolated fibre/industrial food substance |  |
| Chicory | Isolated fibre/industrial food substance |  |
|  |  |  |
| Anti-caking agents | Cosmetic additive |  |
| *Can be listed as:* | | |
| Cellulose | Anti-caking agent |  |
|  |  |  |
| Hydrogenated oils | Hydrogenation is a process characteristic of ultra-processing |  |
| *Can be listed as:* |  |  |
| Hydrogenated palm oil | Hydrogenated oil |  |
| Shortening | Hydrogenated oil |  |
|  |  |  |
| Margarine | Ultra-processed food -produced through multiple industrial processes |  |
| Bran straws | Industrial food substance |  |
| Alcoholic liqueurs | Distilled alcohol |  |
| Alcoholic spirits | Distilled alcohol |  |
| Sherry | Fortified wine (uses a spirit for fermentation) |  |
| Brandy | Fortified wine (uses a spirit for fermentation) |  |
| Aromas | Cosmetic additive |  |
| Aroms of truffle | Cosmetic additive |  |
| Fish sauce | Usually contains mups (e.g extracts) | Unless ingredients listed and no mups present |
| Oyster sauce | Usually contains mups (e.g extracts) | Unless ingredients listed and no mups present |
| Soy sauce | Usually contains mups (e.g extracts) | Unless ingredients listed and no mups present |
| Tamari sauce | Usually contains mups (e.g extracts) | Unless ingredients listed and no mups present |
| Seasonings | Usually contains mupss (e.g. Maltodextrin) | Unless ingredients listed and no mups present |
| Stocks | Usually contains mups (e.g. Flavours) | Unless ingredients listed and no mups present |

***This is not an extensive list of all potential MUPs identified in this research but does include the most commonly encountered.**

**Supplementary Table 2. Ingredients not considered markers of ultra-processing (MUPs) in the Australian food supply for this research*.**

| **Not considered mups** | **Purpose/reason** |
| --- | --- |
| Mineral salts | Processed culinary ingredient (NOVA group 2) |
| Reconstituted juices | Processing techniques not always clear |
| Starches | A processed culinary ingredient (NOVA group 2) |
| Stabilisers | Preservation purpose |
| Preservatives | Preservation purpose |
| Sodium Benzoate | Preservative |
| Potassium Sorbate | Preservative |
| Sodium nitrite | Preservative in meat products |
| Acidity regulators | Preservation purpose |
| Food acids | Preservatives |
| Citric acid | Preservative |
| Antioxidants | Preservatives |
| Fruit or vegetable powders | Dehydration/grinding a minimal process |
| Dehydrated fruits or vegetables | Dehydration a minimal process |
| Garlic powder | Dehydration/grinding a minimal process |
| Dried herbs | Dehydration a minimal process |
| Starches | Processed culinary ingredients (NOVA group 2) |
| Potato starch | Processed culinary ingredient (NOVA group 2) |
| Tapioca starch | Processed culinary ingredient (NOVA group 2) |
| Wheat starch | Processed culinary ingredient (NOVA group 2) |
| Tomato paste | Concentrated tomatoes not derived from an industrial process |
| Tomato concentrate | Same as tomato paste |
| Sugar | Processed culinary ingredient (NOVA group 2) |
| Brown sugar | Processed culinary ingredient (NOVA group 2) |
| Molasses | Processed culinary ingredient (NOVA group 2) |
| Honey | Processed culinary ingredient (NOVA group 2) |
| Cane sugar syrup | Processed culinary ingredient (NOVA group 2) |
| Salt | Processed culinary ingredient (NOVA group 2) |
| Idodised salt | Fortified salt, processed culinary ingredient |
| Enzymes | Usually have a functional purpose (e.g. Lactose free milk) |
| Bacteria cultures | Used for fermentation and nutrient fortification purposes |
| Vegetable oils | Processed culinary ingredient |
| Vegetable fats | Processed culinary ingredient |
| Baking powder | Raising agent |
| Raising agents | Processed culinary ingredient (NOVA group 2) |
| Yeast | Raising agent - a processed culinary ingredient (NOVA group 2) |
| Milk solids | Dehydrated/powdered milk |
| Wine | Alcohol produced through fermentation – NOVA group 3 |
| Beer | Alcohol produced through fermentation – NOVA group 3 |
| Cocoa powder | Minimally processed food |
| Cocoa mass | Minimally processed food |
| Cocoa butter | Processed culinary ingredient |
| Vinegar | Processed culinary ingredient |
| Vitamins/minerals | Fortification purposes (remain NOVA group 1) |
| Wheat bran | Minimal processing involved |

***This is not an extensive list of all ingredients *not* considered MUPs in this research but rather the most commonly questioned/misidentified ingredients.**

**Procedure for estimating number of MUPs for ultra-processed items in AUSNUT 2011-2013**

1. The product foodcode was searched in AUSNUT *food details file (8a)* spreadsheet. Relevant details were recorded: product or brands that the specific item number referred to; how nutrient data was estimated (e.g., sampling or recipe); or what types of products were included in the sampling.
2. If the foodcode is based on a **specific brand name product** or multiple commercial brands were sampled for analysis, either Mintel GNPD or supermarket websites (Woolworths or Coles) were referred to for ingredients lists. Where possible, the version of the product released closest to the AUSNUT 2011-2013 timeframe was chosen (when using Mintel GNPD). If the *food details file* did not refer to one specific brand product (e.g., Kellogg’s Coco pops), 2 or 3 examples were copied from Mintel or supermarket websites and the average number of MUPs was taken as the final value.
3. If the nutrient details were estimated from a **recipe approach**, the foodcode was searched in the AUSNUT *recipe file (8c)* spreadsheet. MUPs were then counted from the recipe file. If recipe the was based on basic ingredients (no commercial products) when the item was known to be commercial product other methods of estimation were used. If the recipe was produced by averaging items also made from recipes/multiple ingredients, the item making up the largest proportion was used as the new reference.
4. If MUPs could not be estimated from the previous steps, then MUPs were based on a similar item in the AUSNUT database. If the item had only minor differences to another product (e.g., items with or without added salt for example will have the same number of MUPs), number of MUPs could be replicated.

**
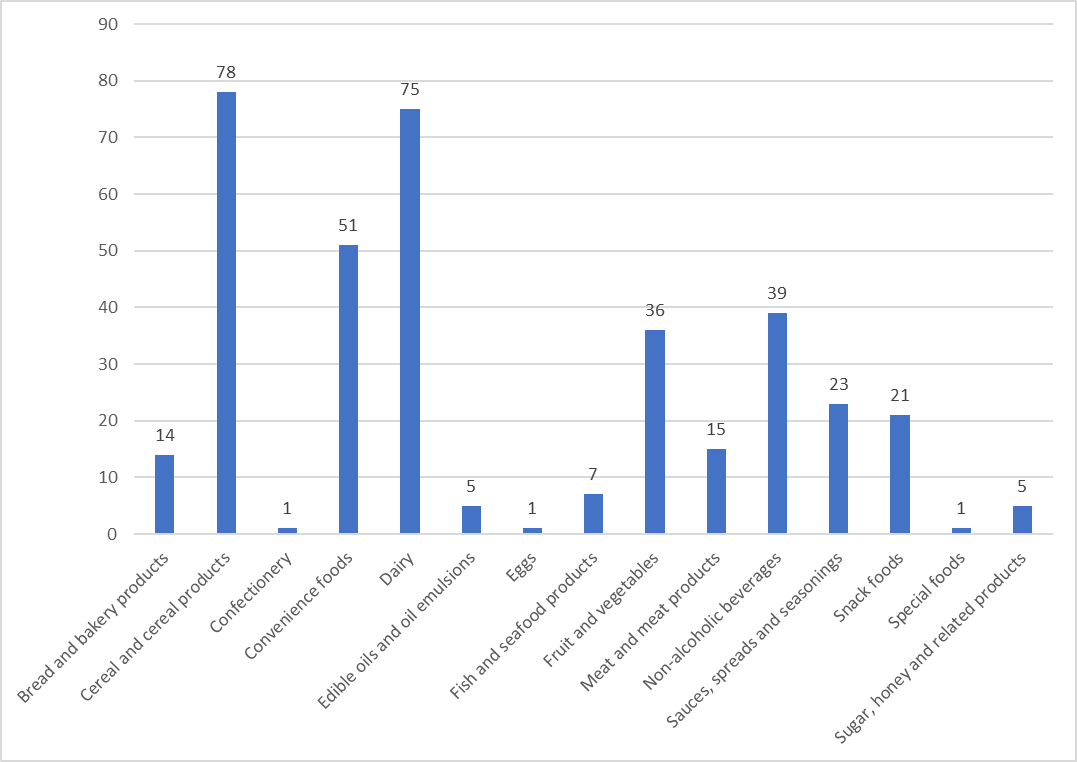
**

**Supplementary Figure 1. Number of ultra-processed products in sub-group 4.1 (1 marker of ultra-processing) classified as healthy by Model 2 (n=367), by category.**


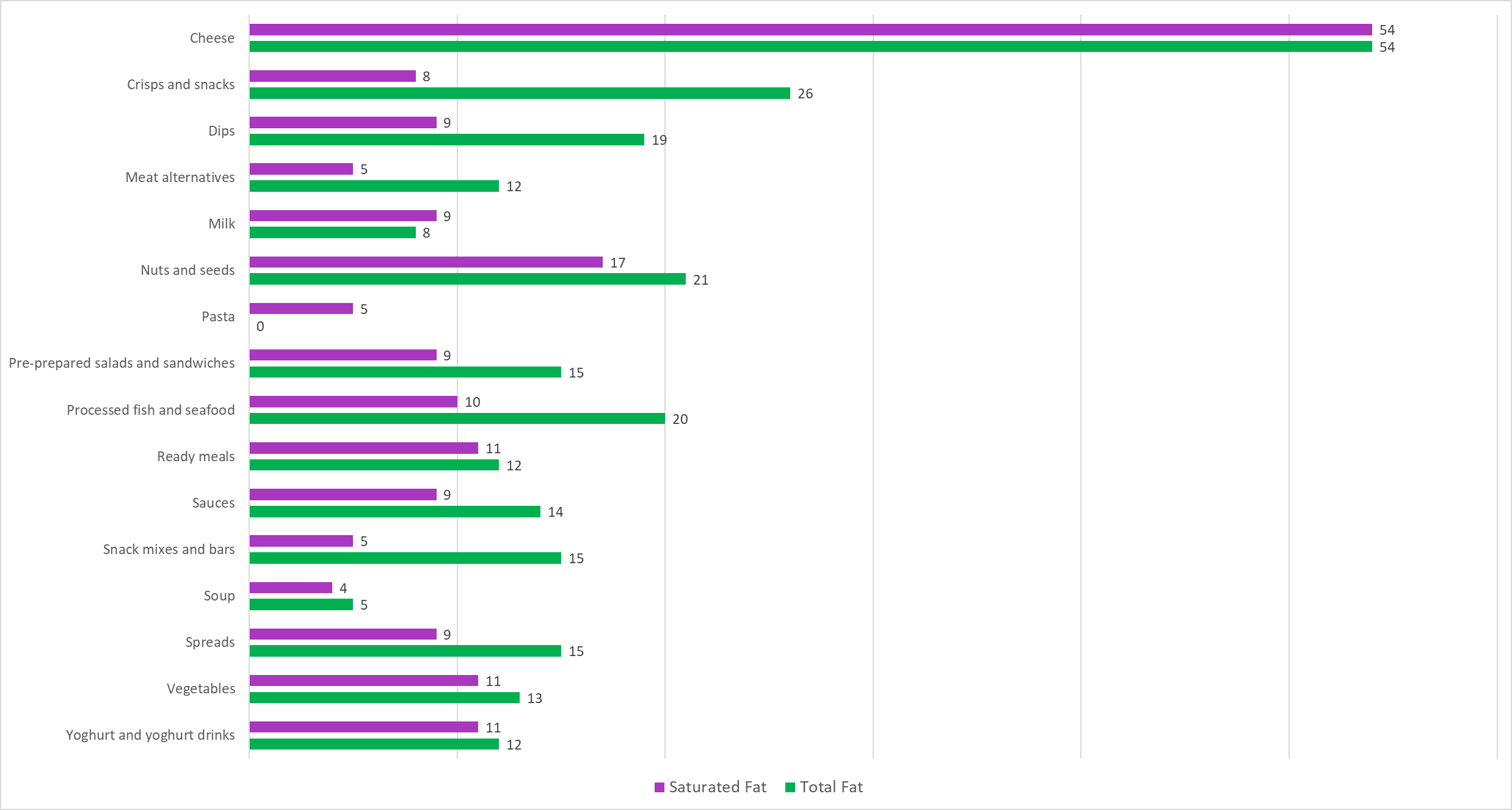


**Supplementary Figure 2. Number of NOVA group 3 food items classified as healthy by model 1 and 2 but unhealthy by the Pan American Health Organization's Nutrient Profiling Model because they exceed the saturated fat or total fat thresholds, by subcategory. Only categories with n>5 presented.**

**Supplementary Table 3. Examples of food and beverage items for different classification scenarios, displaying product name and ingredient list. Markers of ultra-processing and content of free sugars and sodium when exceeding thresholds are highlighted in red.**

| **Sub-group 4.1 and healthy by Model 2 (not exceeding sodium or free sugar thresholds)** | | | | | |
| --- | --- | --- | --- | --- | --- |
| **Item** | Gluten & Wheat Free Rice Macaroni | Vegetable Fried Rice | Lemon Juice | Nut Mix | Three Cheese Blend |
| **Ingredients** | Rice flour, rice bran, beetroot powder, spinach powder, vegetable gum (405), added water | cooked jasmine rice (45%) (water, jasmine rice), baked egg (13%) (pasteurised free range liquid egg, milk, canola oil), brown mushroom (8%), Chinese cabbage (8%), carrot, onion (5%), spring onion, canola oil, soy sauce (contains wheat, soybeans), ginger, coriander, sesame oil, salt | reconstituted lemon juice (99.9%), food acid (ascorbic acid), natural flavour, preservative (223) | Nuts (56%) (peanuts, almonds, cashews), noodles (yellow peas, chickpeas, sunflower oil and/or canola oil, rice, potato, tapioca, sesame, salt), green peas, chickpeas, sunflower oil, canola oil, cane sugar, salt, spices (chilli, paprika, turmeric, cumin), maltodextrin, vegetable powder | mozzarella (40%) (milk, salt, starter culture, enzyme (non-animal rennet)), colby (40%) (milk, salt, starter culture, enzyme (non-animal rennet)), parmesan (20%) (milk, salt, starter culture, enzyme), anti-caking (460) |
| **Item** | Hommus | Raspberry Greek Yoghurt | Beetroot Lentil Bites | Chilli & Olive Sauce | Chickpea & Lentil Burger |
| **Ingredients** | cooked chickpeas (72%) (chickpeas, water), tahini (10%) (sesame paste), canola oil, vinegar, salt, food acid (330), garlic (0.5%), preservatives (202, 211), vegetable gum (415) | whole milk, cream, milk solids, seedless raspberry pulp (4%), rice starch, natural flavour, live yoghurt culture | lentil flour (26%), sunflower oil, maize flour, chickpea flour (15%), rice flour, potato starch, beetroot powder (5%), chickpea fibre, salt | tomato (74%) (tomato puree, reconstituted tomato), water, green olives (11%) (olives, salt, acidity regulator (270)), olive oil, canola oil, sugar, herbs and spices (chilli), salt, lemon juice, spice extract (160c) | vegetables, chickpeas (30%), lentils (13%), wheat flour (wheat flour, raising agents (450, 500)), breadcrumb (wheat flour, salt, emulsifier (471)), herbs and spices, vegetable oil, sugar, salt, garlic |
| **NOVA Group 3 and unhealthy by Model 1 and 2 (exceeds sodium or free sugar thresholds)** | | | | | |
| **Item** | Mixed Berry Oats + Chia | The Original Biscuits | White Wraps | Vegetable and Bean Soup | Parmesan Cheese |
| **Ingredients** | Whole grain oats (58%), chia seed (13%), coconut sugar, dried mixed berries (strawberries, raspberries, blackberries, blueberries) (10%), virgin coconut oil.  13g/100g free sugars | Wheat flour, sugar, butter (cream, salt), vegetable oil (contains soy), condensed milk, salt, eggs, baking powder.  18g/100g frees sugars | wheat flour, water, soybean oil, sugar, iodised salt, baking powder (contains mineral salts (500, 450, 341)), acidity regulator (297), preservatives (282, 200), mineral salt (500), yeast, vitamins (thiamin, folate). 700mg/100g sodium | Vegetable broth (41.7%) (water, onion, cauliflower, carrot), vegetables (43%) (potato, cabbage, carrot, onion, zucchini), legumes (6%) (borlotti beans, lentils), chicken stock (water, chicken), tomato paste, salt, pepper.  429mg/100g sodium | Pasteurised milk, sodium chloride, starter culture, non-animal enzyme.  1040mg/100g sodium |
| **Item** | Teriyaki Beef Biltong | Sesame Delicate Rice Wafers | Sea Salt Flavoured Sweet Potato Chips | Rich Sweetened Condensed Milk | Happy Pig Pizza |
| **Ingredients** | lean Australian beef, salt, spices, brown vinegar, sugar, soy sauce (water, wheat, soybeans, salt)  1700mg/100g sodium | rice (65%), corn flour, potato starch, milk powder, sesame seeds (4%), cheese powder, salt, soy antioxidant (E307B)  733mg/100g sodium | sweet potatoes, sunflower oil, sea salt  576mg/100g sodium | milk, sugar (43%), lactose (from milk)  42g/100g free sugars | wheat flour (contains gluten), mozzarella (pasteurised milk, salt, starter cultures, enzyme (non-animal coagulant)), water, mushrooms, bacon, tomato paste, tomato puree, olives, extra virgin olive oil, canola oil, garlic, salt, capers, sugar, chili flakes, black pepper, yeast, dried oregano  628mg/100g sodium |
| **NOVA Group 3 healthy by Model 1 and 2 but unhealthy by PAHO due to total fat** | | | | | |
| **Item** | Smooth Tofu | Yellow Fish Curry | Quinoa, Baby Broccoli & Fetta Salad | Crunchy Natural Peanut Butter | Double Cream Camembert |
| **Ingredients** | Australian soy beans (100%), filter water, glucono delta lactone, calcium sulphate, magnesium chloride | fish (hoki) (33%), yellow curry sauce (17%) (water, coconut powder (3%), onion, milk powder, tomato paste, garlic, cornflour, lemongrass, salt, turmeric, chilli, lemon juice), brown lentils (17%) (lentils, water, salt, antioxidant (ascorbic acid)), pumpkin (13%), green beans (10%), zucchini (10%) | Broccoli (19%), pumpkin (16%), quinoa (16%), carrot (12%), zesty lemon dressing (12%) (water, canola oil, lemon juice, vinegar, maple syrup, sugar, lemon, salt, stabiliser (415), black pepper), rocket (11%), vegetarian feta cheese (11%) (milk, salt, lactic starter culture, non-animal rennet), hemp seeds (3%) | roasted peanuts (99.5%), salt | pasteurised milk, pasteurised cream, starter culture, salt, non-animal rennet |
| **NOVA Group 3 – Healthy by Model 1 and 2 but unhealthy by PAHO due to saturated fat** | | | | | |
| **Item** | Salted Cashews | Vegetable Mash | Original Deliciously Seedy Crackers | Natural Coconut Yogurt | Bocconcini |
| **Ingredients** | cashews (96%), sunflower oil, sea salt (1%) | vegetables (96%) (pumpkin (34%), potato (34%), sweet potato (17%), carrot (11%)), butter (2%) (milk), milk (1.5%) (milk), salt, pepper (0.02%), nutmeg (0.01%), garlic (0.01%) | organic sunflower seeds, organic golden linseeds, organic sesame seeds, organic psyllium husk, organic coconut oil, sea salt | Coconut yogurt (coconut, water, cornflour, cultures) | milk, salt, food acid (330), rennet |

**PAHO: Pan American Health Organization's Nutrient Profiling Model**
