# Supplementary material for: A novel food processing-based nutrition classification scheme for guiding policy actions applied to the Australian food supply
Source: Front Nutr. 2023 Jan 20;10:1071356. doi: 10.3389/fnut.2023.1071356 (PMC9895835; doi:10.3389/fnut.2023.1071356)
Supplement: Supplementary file 2 [file Table_1.docx]

**Potential anomalies for food and beverage items classified as healthy (Model 1 and 2)**

| **Product** | **Model 1** | **Model 2** |
| --- | --- | --- |
| Cheez 'n Chive Flavour Mac n Cheez | Healthy | Healthy |
| Chilli Cheez Flavour Mac n Cheez | Healthy | Healthy |
| Original Cheez Flavour Mac N Cheez Pasta Meal | Healthy | Healthy |
| Porcini and Cheez Flavour Mac N Cheez Pasta Meal | Healthy | Healthy |
| Pastry, puff, with butter, commercial, raw | Unhealthy | Healthy |
| Pastry, puff, with butter, commercial, baked | Unhealthy | Healthy |
| Strawberries, Apple and Chamomile Wellness Jelly | Unhealthy | Healthy |
| Mac & Cheese Croquettes | Unhealthy | Healthy |
| Mac & Cheese Balls | Unhealthy | Healthy |
| Dumpling or wonton, savoury, meat & vegetable filled, takeaway style, cooked | Unhealthy | Healthy |
| Samosa, vegetable, deep fried | Unhealthy | Healthy |
| One-Pan Brekky with Beef Chipolata Sausages | Healthy | Healthy |
| Potato & Chorizo | Healthy | Healthy |
| Butter Chicken Naanwich | Unhealthy | Healthy |
| Chilli Dynamite | Unhealthy | Healthy |
| Chilli Wedges | Unhealthy | Healthy |
| Rice Mac & Cheese | Unhealthy | Healthy |
| Pork Belly Buns with Shallot & Hoisin | Unhealthy | Healthy |
| Chunky Pepper Steak Pie | Unhealthy | Healthy |
| Mild Butter Chicken & Basmati Rice | Unhealthy | Healthy |
| Bangers & Sweet Potato Mash | Healthy | Healthy |
| Pancetta Macaroni & Cheese | Unhealthy | Healthy |
| Fettucine with Chicken and Cream Sauce | Healthy | Healthy |
| Cheese & Leek Plaits | Unhealthy | Healthy |
| Ham & Cheese Quiche | Healthy | Healthy |
| Spring Onion Cream Cheese | Healthy | Healthy |
| Spreadable Light Cream Cheese | Healthy | Healthy |
| Barossa Triple Cream Cheese | Healthy | Healthy |
| Lactose Free Original All Natural Cream Cheese Block | Unhealthy | Healthy |
| Cheese, cream, herb or spice flavoured | Unhealthy | Healthy |
| Cheese spread, cream cheese, regular fat | Unhealthy | Healthy |
| Espresso Cream | Unhealthy | Healthy |
| Vanilla Cream | Unhealthy | Healthy |
| Pure Double Cream | Healthy | Healthy |
| Chocolate Heaven Whipped Cream | Unhealthy | Healthy |
| Creamy Rice Snack with Vanilla | Unhealthy | Healthy |
| Creamy Rice Snack with Coconut Milk | Healthy | Healthy |
| Chocolate Custard | Unhealthy | Healthy |
| Strawberry Flavoured Coconut Milk Mousse | Unhealthy | Healthy |
| Chocolate Flavoured Coconut Milk Mousse | Unhealthy | Healthy |
| Mango Flavoured Coconut Milk Mousse | Unhealthy | Healthy |
| Dairy dessert, flavours other than chocolate, regular fat | Unhealthy | Healthy |
| Sweetened Organic Coconut Yogurt Alternative | Unhealthy | Healthy |
| Chocolate Mousse Organic Coconut Yogurt Alternative | Unhealthy | Healthy |
| Fish finger, crumbed, packaged frozen, raw | Unhealthy | Healthy |
| Fish finger, crumbed, packaged frozen, baked, roasted, fried or grilled, no added fat | Unhealthy | Healthy |
| Fish finger, crumbed, packaged frozen, baked, roasted, fried, grilled or BBQ'd, fat not further defined | Unhealthy | Healthy |
| Blueberries in Syrup | Healthy | Healthy |
| South African Peach Slices in Syrup | Healthy | Healthy |
| Apple Fruit Puree | Unhealthy | Healthy |
| Peach Halves in Syrup | Unhealthy | Healthy |
| Sliced Pineapple in Light Syrup | Healthy | Healthy |
| Pear, canned in light syrup | Unhealthy | Healthy |
| Pear, canned in light syrup, drained | Unhealthy | Healthy |
| Potato, wedges, regular, purchased frozen, par-fried in canola oil, raw | Unhealthy | Healthy |
| Potato, wedges, regular, purchased frozen, baked or roasted, no added fat | Unhealthy | Healthy |
| Potato, wedges, regular, purchased frozen, deep fried or fried, fat not further defined | Unhealthy | Healthy |
| Lightly Battered Prawns | Unhealthy | Healthy |
| Lightly Crumbed Scallops | Unhealthy | Healthy |
| Black Truffle Duck Paté | Healthy | Healthy |
| Chicken & Madeira Pâté | Unhealthy | Healthy |
| Duck & Shiraz Pâté | Healthy | Healthy |
| Sausage, beef, plain or flavoured, reduced fat, fried, grilled, BBQ'd or baked | Unhealthy | Healthy |
| Sausage, beef, plain or flavoured, reduced fat, boiled, casseroled, microwaved, poached, steamed or stewed | Unhealthy | Healthy |
| Roasted Duck Fat Potatoes | Healthy | Healthy |
| Crinkle Cut Potato Chips | Unhealthy | Healthy |
| Potato, gem, nugget or royal, regular, purchased frozen, par-fried in canola oil, raw | Unhealthy | Healthy |
| Potato, gem, nugget or royal, regular, purchased frozen, baked or roasted, with or without added fat | Unhealthy | Healthy |
| Potato, gem, nugget or royal, regular, purchased frozen, deep fried or fried, fat not further defined | Unhealthy | Healthy |
| Potato, hash brown, purchased frozen, par-fried in canola oil, raw | Unhealthy | Healthy |
| Potato, hash brown, purchased frozen, baked, roasted, grilled or BBQ'd, no added fat | Unhealthy | Healthy |
| Chicken & Madeira Pâté | Unhealthy | Healthy |
| Duck & Shiraz Pâté | Healthy | Healthy |
| Gourmet Aioli Garlic Mayonnaise | Healthy | Healthy |
| Chilli & Lime Mayonnaise | Healthy | Healthy |
| Ginger & Sesame Mayonnaise | Healthy | Healthy |
| Real Whole Egg Creamy Mayonnaise | Healthy | Healthy |
| Garlic Aioli Mayonnaise | Healthy | Healthy |
| Tartare Sauce | Healthy | Healthy |
| Slow Cooked Potato Chips | Healthy | Healthy |
| Pizza Flavoured Roasted Fav-va Beans | Healthy | Healthy |
| Parsnip Crisps | Healthy | Healthy |
| Andean Potato Crisps Mix | Healthy | Healthy |
| Sea Salt Flavoured Protein Chips | Unhealthy | Healthy |
| Avocado Oil Classic Sea Salt Potato Chips | Healthy | Healthy |
| BBQ Blast Flavour Air Puffed Fava Beans | Unhealthy | Healthy |
| Cheese Puffed Corn Chips | Unhealthy | Healthy |
| Potato crisps or chips, plain, unsalted | Unhealthy | Healthy |
| Mac' Lemon Paleo Bar | Healthy | Healthy |
| Choc Mud Bars | Healthy | Healthy |
| Choc Sea Salt Real Food Protein Bar | Healthy | Healthy |
| Creamy Coconut Mango & Plant Protein Organic Snack Bar | Unhealthy | Healthy |
| Salted Caramel Sauce | Healthy | Healthy |

**Food and beverage items in sub-group 4.1 (Model 2) classified as healthy when sodium and/or free sugars assessed**

| **Product** | **Model 1** | **Model 2** |
| --- | --- | --- |
| Original Rice Cakes | Unhealthy | Healthy |
| Sea Salt Flavoured Protein Crackers | Unhealthy | Healthy |
| Cheese & Onion Rice Crackers | Unhealthy | Healthy |
| Biscuit, savoury, from wholemeal wheat flour, crispbread | Unhealthy | Healthy |
| Biscuit, savoury, from wholemeal wheat flour & rye flour, crispbread, puffed | Unhealthy | Healthy |
| Biscuit, savoury, rice cake, from brown rice, plain | Unhealthy | Healthy |
| Biscuit, savoury, corn cake, multigrain, salted | Unhealthy | Healthy |
| Biscuit, savoury, corn cake, multigrain, salted | Unhealthy | Healthy |
| Multi Fruit Sourdough Loaf | Unhealthy | Healthy |
| Sliced White Toast | Unhealthy | Healthy |
| Pane Di Casa Sliced Bread | Unhealthy | Healthy |
| Coconut & Vanilla Flavour Muesli Bar Mix | Unhealthy | Healthy |
| Pastry, puff, with butter, commercial, raw | Unhealthy | Healthy |
| Pastry, puff, with butter, commercial, baked | Unhealthy | Healthy |
| Sourdough Bread Base | Unhealthy | Healthy |
| Ancient Grains and Superfruits Natural Muesli | Unhealthy | Healthy |
| Roasted Almond Muesli | Unhealthy | Healthy |
| Almond, Coconut & Chia Gourmet Porridge Sachets | Unhealthy | Healthy |
| Black Rice, Honey & Cinnamon Pudding Brekky Rice | Unhealthy | Healthy |
| Honey Roasted Nut Gourmet Porridge Sachets | Unhealthy | Healthy |
| Cranberry & Fig Baked Muesli | Unhealthy | Healthy |
| Breakfast cereal, flakes of corn, unfortified | Unhealthy | Healthy |
| Breakfast cereal, flakes of corn, no added sugar or salt, unfortified | Unhealthy | Healthy |
| Breakfast cereal, puffed or popped corn, no added sugar or salt, unfortified | Unhealthy | Healthy |
| Breakfast cereal, puffed or popped rice, no added sugar or salt, unfortified | Unhealthy | Healthy |
| Breakfast cereal, whole wheat, biscuit, no added sugar or salt, unfortified | Unhealthy | Healthy |
| Breakfast cereal, whole wheat, biscuit, no added sugar, unfortified | Unhealthy | Healthy |
| Breakfast cereal, whole wheat, biscuit, added sugar & salt, unfortified | Unhealthy | Healthy |
| Breakfast cereal, whole wheat, flakes, no added sugar, unfortified | Unhealthy | Healthy |
| Breakfast cereal, whole wheat, puffed, no added sugar or salt, unfortified | Unhealthy | Healthy |
| Breakfast cereal, wheat bran, flakes, added vitamins B1, B2 B3, B6, & folate, Fe & Zn | Unhealthy | Healthy |
| Breakfast cereal, whole wheat, biscuit, added vitamins B1, B2 & B3 | Unhealthy | Healthy |
| Breakfast cereal, whole wheat, biscuit, added vitamins B1, B2 & B3, Ca, Fe & Zn | Unhealthy | Healthy |
| Breakfast cereal, whole wheat, biscuit, added vitamins B1, B2, B3 & folate, Fe & Zn | Unhealthy | Healthy |
| Breakfast cereal, whole wheat, biscuit, bran, added vitamins B1, B2, B3 & folate & Fe | Unhealthy | Healthy |
| Breakfast cereal, whole wheat, biscuit, not further defined | Unhealthy | Healthy |
| Breakfast cereal, whole wheat, flakes, added vitamins B1, B2, B3 & folate | Unhealthy | Healthy |
| Breakfast cereal, whole wheat, small biscuit, added vitamins B1, B2, B3 & folate | Unhealthy | Healthy |
| Breakfast cereal, wheat based, commercial, not further defined | Unhealthy | Healthy |
| Breakfast cereal, wheat bran, flakes, sultanas, added vitamins B1, B2, B3, B6 & folate, Fe & Zn | Unhealthy | Healthy |
| Breakfast cereal, oat & whole wheat, biscuit, added salt, unfortified | Unhealthy | Healthy |
| Breakfast cereal, whole wheat, biscuit, multigrain, added vitamins B1, B2, B3 & E & Fe | Unhealthy | Healthy |
| Muesli, commercial, gluten free | Unhealthy | Healthy |
| Muesli, commercial, toasted, added dried fruit, unfortified | Unhealthy | Healthy |
| Muesli, commercial, toasted, added dried fruit & nuts, unfortified | Unhealthy | Healthy |
| Muesli, commercial, toasted, added nuts, unfortified | Unhealthy | Healthy |
| Breakfast cereal, not further defined | Unhealthy | Healthy |
| Israeli Pearl Couscous | Unhealthy | Healthy |
| Instant Brown Rice Ramen | Unhealthy | Healthy |
| Instant Buckwheat Ramen | Unhealthy | Healthy |
| Wok Ready Flat Noodles | Unhealthy | Healthy |
| Chow Mein Fresh Noodles | Unhealthy | Healthy |
| Zen Garden Vegetables Flavoured Noodles | Unhealthy | Healthy |
| Medium Egg Noodles | Unhealthy | Healthy |
| Original Kelp Noodles | Unhealthy | Healthy |
| Kona Berry Kelp Noodles | Unhealthy | Healthy |
| Quinoa Pasta Elbows | Unhealthy | Healthy |
| Saffron Twists | Unhealthy | Healthy |
| Organic Brown Rice Twists | Unhealthy | Healthy |
| Gluten Free Fettuccine Pasta | Unhealthy | Healthy |
| Spaghetti | Unhealthy | Healthy |
| Chicken Parmigiana Ravioli | Unhealthy | Healthy |
| Gluten Free Spinach Fettucini | Unhealthy | Healthy |
| Ravioli Grandi with Italian Sausage & Fennel | Unhealthy | Healthy |
| Roast Pumpkin & Sage Ravioli Fresh Pasta | Unhealthy | Healthy |
| Sweet Potato & Parmesan Ravioli | Unhealthy | Healthy |
| Lemon & Parsley Linguine | Unhealthy | Healthy |
| Noodle, wheat, instant, unflavoured, boiled, drained | Unhealthy | Healthy |
| Noodle, wheat, instant, low fat, unflavoured, boiled, drained | Unhealthy | Healthy |
| Noodle, wheat, instant, low fat, flavoured, boiled, drained | Unhealthy | Healthy |
| Quinoa Cups | Unhealthy | Healthy |
| Persian Quinoa with Dates and Pistachio | Unhealthy | Healthy |
| Steamed Brown Rice Quick Cup | Unhealthy | Healthy |
| Long Grain Steamed Rice | Unhealthy | Healthy |
| Brown Rice & Chia | Unhealthy | Healthy |
| Vegetable Fried Rice | Unhealthy | Healthy |
| Brown Rice & Chia Seed | Unhealthy | Healthy |
| Carnaroli Rice with Vegetables | Unhealthy | Healthy |
| Edamame, Spring Onion & Wasabi with Wholegrain Basmati Rice | Unhealthy | Healthy |
| Black Bean, Jerk & Coconut with Wholegrain Basmati Rice | Unhealthy | Healthy |
| Split Pea, Green Chilli & Coriander with Wholegrain Basmati Rice | Unhealthy | Healthy |
| Tasmanian Mushroom and Black Truffle Risotto Rice | Unhealthy | Healthy |
| Brown Rice | Unhealthy | Healthy |
| Basmati Rice | Unhealthy | Healthy |
| Coconut, Chilli & Lemongrass Rice | Unhealthy | Healthy |
| Strawberries, Apple and Chamomile Wellness Jelly | Unhealthy | Healthy |
| Cabbage & Pork Dumplings | Unhealthy | Healthy |
| Hong Kong Style Pork Dumplings | Unhealthy | Healthy |
| Soup Dumplings | Unhealthy | Healthy |
| Mac & Cheese Croquettes | Unhealthy | Healthy |
| Mac & Cheese Balls | Unhealthy | Healthy |
| Dumpling or wonton, savoury, meat & vegetable filled, takeaway style, cooked | Unhealthy | Healthy |
| Samosa, vegetable, deep fried | Unhealthy | Healthy |
| Ranch Slaw Kit | Unhealthy | Healthy |
| Chicken with Green Peppercorn & Pearl Barley Recipe Bag | Unhealthy | Healthy |
| Falafel Protein Bistro Box | Unhealthy | Healthy |
| Japanese Ramen Kit | Unhealthy | Healthy |
| Thai Massaman Curry | Unhealthy | Healthy |
| Thai Red Curry | Unhealthy | Healthy |
| Kaleslaw Salad Kit | Unhealthy | Healthy |
| Ultimate Tomato & Mozzarella Pizza | Unhealthy | Healthy |
| Chicken Basil Penne Salad | Unhealthy | Healthy |
| Vietnamese Chicken Salad | Unhealthy | Healthy |
| Teriyaki Chicken Salad | Unhealthy | Healthy |
| Lentil Salad | Unhealthy | Healthy |
| Butter Chicken Naanwich | Unhealthy | Healthy |
| Italian Penne Pasta Salad with Sundried Tomato & Kalamata Olive | Unhealthy | Healthy |
| Tandoori Chicken Flatbread Wrap | Unhealthy | Healthy |
| Hot Smoked Salmon Poke Bowl | Unhealthy | Healthy |
| Teriyaki Chicken Flavoured Noodle Salad Bowl | Unhealthy | Healthy |
| Asian Style Salad Bowl | Unhealthy | Healthy |
| Kaleslaw Kit | Unhealthy | Healthy |
| Mexican Style Salad Bowl | Unhealthy | Healthy |
| Hot Smoked Salmon & Rainbow Salad | Unhealthy | Healthy |
| Rainbow Whole Bowl | Unhealthy | Healthy |
| Green Whole Bowl | Unhealthy | Healthy |
| Chilli Dynamite | Unhealthy | Healthy |
| Chilli Wedges | Unhealthy | Healthy |
| Lasagne Bolognese | Unhealthy | Healthy |
| Rice Mac & Cheese | Unhealthy | Healthy |
| Pork Belly Buns with Shallot & Hoisin | Unhealthy | Healthy |
| Mild Chicken & Rice Curry | Unhealthy | Healthy |
| Chunky Pepper Steak Pie | Unhealthy | Healthy |
| Mild Butter Chicken & Basmati Rice | Unhealthy | Healthy |
| Massaman Chicken with Rice | Unhealthy | Healthy |
| Pancetta Macaroni & Cheese | Unhealthy | Healthy |
| Vegetable Dahl Curry | Unhealthy | Healthy |
| Turmeric Omelette with Chipotle Jam | Unhealthy | Healthy |
| Lamb Rogan Josh with Rice | Unhealthy | Healthy |
| Kangaroo Chilli Con Carne with Green Penne | Unhealthy | Healthy |
| Jamaican Jerk Chicken & Black-Eyed Beans | Unhealthy | Healthy |
| Egg White Omelette & Potato Smash | Unhealthy | Healthy |
| Chicken Tikka Masala | Unhealthy | Healthy |
| Satay Cashew Free Range Chicken on Rice with Broccoli and Cauliflower | Unhealthy | Healthy |
| Beef Madras | Unhealthy | Healthy |
| Sauce, pasta, beef bolognese, commercial | Unhealthy | Healthy |
| Frozen meal, vegetables & pasta or noodles, energy controlled portion | Unhealthy | Healthy |
| Curry, commercial, legume (dhal) | Unhealthy | Healthy |
| Cheese & Leek Plaits | Unhealthy | Healthy |
| Beef Bone Broth | Unhealthy | Healthy |
| 5 Cheese Italian Style Cooking Blend | Unhealthy | Healthy |
| Black Truffle Australian Fetta | Unhealthy | Healthy |
| Three Cheese Blend | Unhealthy | Healthy |
| Chef Style Shredded Cheddar | Unhealthy | Healthy |
| Rubicon Red Cheese | Unhealthy | Healthy |
| Natural Australian Mozzarella Shredded Cheese | Unhealthy | Healthy |
| Bocconcini Dairy Free Cheese | Unhealthy | Healthy |
| Yorkshire Wensleydale & Apricots Cheese | Unhealthy | Healthy |
| Lactose Free Original All Natural Cream Cheese Block | Unhealthy | Healthy |
|  |  |  |
| Cheese, cream, herb or spice flavoured | Unhealthy | Healthy |
| Cheese spread, cream cheese, regular fat | Unhealthy | Healthy |
| Espresso Cream | Unhealthy | Healthy |
| Vanilla Cream | Unhealthy | Healthy |
| Chocolate Heaven Whipped Cream | Unhealthy | Healthy |
| Lactose Free Light Sour Cream | Unhealthy | Healthy |
| Thickened Cream | Unhealthy | Healthy |
| Chocolate Heaven Whipped Cream | Unhealthy | Healthy |
| Creamy Rice Snack with Vanilla | Unhealthy | Healthy |
| Chocolate Custard | Unhealthy | Healthy |
| Strawberry Flavoured Coconut Milk Mousse | Unhealthy | Healthy |
| Chocolate Flavoured Coconut Milk Mousse | Unhealthy | Healthy |
| Mango Flavoured Coconut Milk Mousse | Unhealthy | Healthy |
| Dairy dessert, flavours other than chocolate, regular fat | Unhealthy | Healthy |
| Strawberry Cream and Real Fruit Ice Pops | Unhealthy | Healthy |
| Espresso Shot Flavoured Milk | Unhealthy | Healthy |
| Almond Milk | Unhealthy | Healthy |
| Unsweetened Soy Milk | Unhealthy | Healthy |
| Affogato Flavoured Milk | Unhealthy | Healthy |
| Double Shot Iced Coffee | Unhealthy | Healthy |
| Coffee | Unhealthy | Healthy |
| Beverage with Real Chia, Real Vanilla, Real Milk and Real Oats | Unhealthy | Healthy |
| Ice Coffee Breakfast Smoothie | Unhealthy | Healthy |
| Easy to Digest Chocolate Milk | Unhealthy | Healthy |
| Unsweetened Soy Milk | Unhealthy | Healthy |
| Vanilla Coconut Milk Yoghurt | Unhealthy | Healthy |
| Banana and Mango Coconut Milk Yoghurt | Unhealthy | Healthy |
| Activated Almond Milk | Unhealthy | Healthy |
| Almond & Coconut Milk | Unhealthy | Healthy |
| Vanilla Malt Flavoured Milk | Unhealthy | Healthy |
| Skim Milk | Unhealthy | Healthy |
| Iced Mocha Cold Brew Coffee | Unhealthy | Healthy |
| Buttermilk, cultured, 2% fat | Unhealthy | Healthy |
| Soy beverage, regular fat (~3%), added Ca | Unhealthy | Healthy |
| Soy beverage, regular fat (~3%), added Ca & vitamins A, B1, B2, B6, B12, & D | Unhealthy | Healthy |
| Soy beverage, reduced fat (~1.5% fat), added Ca | Unhealthy | Healthy |
| Soy beverage, reduced fat (~1.5% fat), added fibre, Ca & vitamins A, B1, B2, B6 & B12 | Unhealthy | Healthy |
| Soy beverage, reduced fat (~1% fat), added Ca & vitamins A, B2 & B12 | Unhealthy | Healthy |
| Milk, almond, fluid | Unhealthy | Healthy |
| Milk, oat, fluid, unfortified | Unhealthy | Healthy |
| Milk, oat, fluid, added calcium | Unhealthy | Healthy |
| Milk, rice, fluid, added calcium | Unhealthy | Healthy |
| Milk, rice, fluid, protein enriched, added calcium | Unhealthy | Healthy |
| Dahi Indian Style Yoghurt | Unhealthy | Healthy |
| Strawberry Greek Yogurt | Unhealthy | Healthy |
| Tropical Greek Yoghurt | Unhealthy | Healthy |
| Kesar Mango Yogurt Smoothie | Unhealthy | Healthy |
| Pleasantly Tart Blueberry Flavoured Kefir Probiotic Fermented Yogurt Drink | Unhealthy | Healthy |
| Mixed Berry Flavoured Coconut Milk Yoghurt | Unhealthy | Healthy |
| Original Organic Probiotic Kefir | Unhealthy | Healthy |
| Passionfruit Greek Yogurt with Fruit on the Bottom | Unhealthy | Healthy |
| Vanilla Probiotic Coconut Yogurt | Unhealthy | Healthy |
| Mixed Berry Flavoured Coconut Milk Yogurt | Unhealthy | Healthy |
| Greek Style Yogurt with a Hint of Real Honey | Unhealthy | Healthy |
| Natural Coconut Yogurt | Unhealthy | Healthy |
| Sweetened Organic Coconut Yogurt Alternative | Unhealthy | Healthy |
| Chocolate Mousse Organic Coconut Yogurt Alternative | Unhealthy | Healthy |
| Sweet Plain Greek Yogurt | Unhealthy | Healthy |
| Extra Creamy Vanilla Flavoured Yoghurt | Unhealthy | Healthy |
| Natural Probiotic Yogurt | Unhealthy | Healthy |
| Vanilla Bean Yogurt | Unhealthy | Healthy |
| Roasted Almond Natural Yogurt | Unhealthy | Healthy |
| Raspberry Greek Yoghurt | Unhealthy | Healthy |
| Yoghurt, honey flavoured, high fat (~5%) | Unhealthy | Healthy |
| Yoghurt, passionfruit flavoured, regular fat (~3%) | Unhealthy | Healthy |
| Yoghurt, flavoured, reduced fat (~1.5%), homemade from basic ingredients | Unhealthy | Healthy |
| Yoghurt, soy based, berry flavoured, regular fat (approx. 3%) | Unhealthy | Healthy |
| Unsalted Cultured Style Butter | Unhealthy | Healthy |
| Pesto Infusion Extra Virgin Olive Oil | Unhealthy | Healthy |
| Non-Stick Australian Canola Spray | Unhealthy | Healthy |
| Canola Oil Spray | Unhealthy | Healthy |
| Chilli Extra Virgin Olive Oil | Unhealthy | Healthy |
| Omelette, prepared from yolk-free egg mix, fried, butter | Unhealthy | Healthy |
| Classic Tuna Bruschetta | Unhealthy | Healthy |
| Tuna Bruschetta with Mayonnaise & Herbs | Unhealthy | Healthy |
| Smoked Tuna | Unhealthy | Healthy |
| Roe Off Scallops | Unhealthy | Healthy |
| Fish finger, crumbed, packaged frozen, raw | Unhealthy | Healthy |
| Fish finger, crumbed, packaged frozen, baked, roasted, fried or grilled, no added fat | Unhealthy | Healthy |
| Fish finger, crumbed, packaged frozen, baked, roasted, fried, grilled or BBQ'd, fat not further defined | Unhealthy | Healthy |
| Apple Fruit Puree | Unhealthy | Healthy |
| Peach Halves in Syrup | Unhealthy | Healthy |
| Cinnamon Apple Chips | Unhealthy | Healthy |
| Peaches in Juice | Unhealthy | Healthy |
| Mandarin Whole Segments | Unhealthy | Healthy |
| Coconut Chia with Mango & Passionfruit | Unhealthy | Healthy |
| Fruit & Oats with Blueberry and Quinoa Breakfast Pouch | Unhealthy | Healthy |
| Pear, canned in light syrup | Unhealthy | Healthy |
| Pear, canned in light syrup, drained | Unhealthy | Healthy |
| Pineapple, fresh, cooked in light syrup, drained | Unhealthy | Healthy |
| Peach, canned in light syrup | Unhealthy | Healthy |
| Peach, canned in light syrup, drained | Unhealthy | Healthy |
| Apricot, canned in light syrup | Unhealthy | Healthy |
| Apricot, canned in light syrup, drained | Unhealthy | Healthy |
| Chia & Berry Bar | Unhealthy | Healthy |
| Chia & Cacao Bar | Unhealthy | Healthy |
| Pumpkin Seed & Cranberry Salad Topper | Unhealthy | Healthy |
| Sesame & Almond Salad Topper | Unhealthy | Healthy |
| Crinkle Cut Potato Chips | Unhealthy | Healthy |
| Scalloped Potatoes | Unhealthy | Healthy |
| Baby Potatoes with Broccoli, Carrots, Peas & Herbs | Unhealthy | Healthy |
| Roasted Vegetables Delights | Unhealthy | Healthy |
| Bubble 'N Squeak | Unhealthy | Healthy |
| Sweet Potato & Herbs Bites | Unhealthy | Healthy |
| Beetroot Slices | Unhealthy | Healthy |
| Stir Fry Vegetables with Mushrooms Mix | Unhealthy | Healthy |
| Carrot, Cucumber, Capsicum & Tzatziki | Unhealthy | Healthy |
| Refried Beans | Unhealthy | Healthy |
| Potato, gem, nugget or royal, regular, purchased frozen, par-fried in canola oil, raw | Unhealthy | Healthy |
| Potato, gem, nugget or royal, regular, purchased frozen, baked or roasted, with or without added fat | Unhealthy | Healthy |
| Potato, gem, nugget or royal, regular, purchased frozen, deep fried or fried, fat not further defined | Unhealthy | Healthy |
| Potato, hash brown, purchased frozen, par-fried in canola oil, raw | Unhealthy | Healthy |
| Potato, hash brown, purchased frozen, baked, roasted, grilled or BBQ'd, no added fat | Unhealthy | Healthy |
| Potato, wedges, regular, purchased frozen, par-fried in canola oil, raw | Unhealthy | Healthy |
| Potato, wedges, regular, purchased frozen, baked or roasted, no added fat | Unhealthy | Healthy |
| Potato, wedges, regular, purchased frozen, deep fried or fried, fat not further defined | Unhealthy | Healthy |
| Cheese & Veggie Burger | Unhealthy | Healthy |
| Chickpea & Lentil Burger | Unhealthy | Healthy |
| Mixed Vegetable Veggie Burgers | Unhealthy | Healthy |
| Japanese Tofu | Unhealthy | Healthy |
| Lightly Battered Prawns | Unhealthy | Healthy |
| Lightly Crumbed Scallops | Unhealthy | Healthy |
| Soy bean curd skins, dried, rehydrated in boiling water | Unhealthy | Healthy |
| Chicken & Madeira Pâté | Unhealthy | Healthy |
| Australian Angus Beef Burgers with Ale & Shallot | Unhealthy | Healthy |
| Lemon Pepper Smashin' Chicken Fillets | Unhealthy | Healthy |
| Frozen Whole Turkey | Unhealthy | Healthy |
| Sausage, beef, plain or flavoured, reduced fat, fried, grilled, BBQ'd or baked | Unhealthy | Healthy |
| Sausage, beef, plain or flavoured, reduced fat, boiled, casseroled, microwaved, poached, steamed or stewed | Unhealthy | Healthy |
| Chicken, breast, flesh, canned in water, drained | Unhealthy | Healthy |
| Casserole or curry, meat & vegetables, canned, heated | Unhealthy | Healthy |
| Turmeric Matcha Flakes | Unhealthy | Healthy |
| Berry Digest Smoothie Cubes | Unhealthy | Healthy |
| Vegetable &/or fruit blend, prepared from dry powder with water | Unhealthy | Healthy |
| Cocoa powder | Unhealthy | Healthy |
| Cappuccino | Unhealthy | Healthy |
| Original Sparkling Coffee | Unhealthy | Healthy |
| Coffee substitute, cereal beverage, dry powder or granules | Unhealthy | Healthy |
| Raspberry and Pomegranate Iced Cooler with Coconut Water | Unhealthy | Healthy |
| Organic King Coconut Water with Guava | Unhealthy | Healthy |
| Apple Carrot Mango Acerola and Forest Anise Juice | Unhealthy | Healthy |
| Limited Edition Orchard Fruits Juice | Unhealthy | Healthy |
| Water Apple + Cucumber Lemon Drink | Unhealthy | Healthy |
| SuperGreens + Reds with Superfoods Coconut Water | Unhealthy | Healthy |
| Pineapple, Passionfruit, Sweet Potato Juice | Unhealthy | Healthy |
| Easy Tiger Pressed Juice | Unhealthy | Healthy |
| Low Sodium Vegetable Juice | Unhealthy | Healthy |
| Pulp Free Orange Juice + Calcium | Unhealthy | Healthy |
| Orange Mango Juice | Unhealthy | Healthy |
| Apple, Peach + Passionfruit Juice | Unhealthy | Healthy |
| Gimme Green Pressed Juice | Unhealthy | Healthy |
| Juice, lemon, commercial | Unhealthy | Healthy |
| Juice, tomato, commercial | Unhealthy | Healthy |
| Juice, tomato, commercial, added salt | Unhealthy | Healthy |
| Juice, tomato, commercial, sweetened, added salt | Unhealthy | Healthy |
| Juice, vegetable blend, tomato, carrot, celery & parsley, commercial | Unhealthy | Healthy |
| Juice, vegetable, commercial, not further defined | Unhealthy | Healthy |
| Juice, wheatgrass, commercial | Unhealthy | Healthy |
| Berry Black Unsweetened Iced Tea | Unhealthy | Healthy |
| Cucumber and Lavender Beverage | Unhealthy | Healthy |
| Low Sugar Pineapple and Coconut Sparkling Probiotic Tonic | Unhealthy | Healthy |
| Berry Good Kombucha | Unhealthy | Healthy |
| Sparkling Cucumber Mineral Water | Unhealthy | Healthy |
| Sparkling Water with a Hint of Raspberry & Blackcurrant | Unhealthy | Healthy |
| Raspberry & Orange Flavoured Water | Unhealthy | Healthy |
| Lightly Sparkling Spring Water with a Hint of Mango | Unhealthy | Healthy |
| Lemon Infused Spring Water | Unhealthy | Healthy |
| Lightly Sparkling Lime Flavoured Spring Water | Unhealthy | Healthy |
| Lightly Sparkling Spring Water with a Hint of Natural Lemon | Unhealthy | Healthy |
| Romesco | Unhealthy | Healthy |
| Beautiful Beetroot, Tahini & Pomegranate Dip | Unhealthy | Healthy |
| Beetroot, Walnut & Pomegranate Dip | Unhealthy | Healthy |
| Persian Style Fetta with Herbs & Pepper Dip | Unhealthy | Healthy |
| Hommus | Unhealthy | Healthy |
| Cheese & Chive Dip | Unhealthy | Healthy |
| Beetroot & Roasted Almond Dip | Unhealthy | Healthy |
| Tuna Spread | Unhealthy | Healthy |
| Dip, beetroot, commercial | Unhealthy | Healthy |
| Lemon Juice | Unhealthy | Healthy |
| Lime Juice | Unhealthy | Healthy |
| Pumpkin and Tomato Pasta Sauce | Unhealthy | Healthy |
| Extra Bolognese Pasta Sauce | Unhealthy | Healthy |
| Raphael's Chilli & Olive Sauce | Unhealthy | Healthy |
| Sensational Pasta Sauce with Porcini Mushrooms | Unhealthy | Healthy |
| Bolognese Chunky Pasta Sauce | Unhealthy | Healthy |
| Sauce, pasta, tomato-based, commercial | Unhealthy | Healthy |
| Sauce, taco style, commercial | Unhealthy | Healthy |
| Sauce, pasta or simmer, not further defined, commercial, low fat | Unhealthy | Healthy |
| Natural Chicken Stock | Unhealthy | Healthy |
| Natural Beef Stock | Unhealthy | Healthy |
| Australian Vegetable Stock | Unhealthy | Healthy |
| Organic Choco Peanut Butter | Unhealthy | Healthy |
| Carrot & Flax Seed Corn Chips | Unhealthy | Healthy |
| Sea Salt Flavoured Protein Chips | Unhealthy | Healthy |
| Buzzin Beetroot Lentil Bites | Unhealthy | Healthy |
| BBQ Blast Flavour Air Puffed Fava Beans | Unhealthy | Healthy |
| Italian Herbs Grain Bites | Unhealthy | Healthy |
| Cheese Puffed Corn Chips | Unhealthy | Healthy |
| Chick Peas with Zesty Vinaigrette | Unhealthy | Healthy |
| Potato crisps or chips, plain, unsalted | Unhealthy | Healthy |
| Corn chips, plain, toasted, no added salt | Unhealthy | Healthy |
| Corn chips, plain toasted, salted | Unhealthy | Healthy |
| Taco shell, from corn flour, plain | Unhealthy | Healthy |
| Grain waves wheat snack, plain, original | Unhealthy | Healthy |
| Cheese Flavoured Air Popped Corn | Unhealthy | Healthy |
| Nut Mix | Unhealthy | Healthy |
| Almond & Cacao Nibs Bar | Unhealthy | Healthy |
| Strawberry & Apple Snack Bars | Unhealthy | Healthy |
| Creamy Coconut Mango & Plant Protein Organic Snack Bar | Unhealthy | Healthy |

**Classifications for all items in the dataset (n=7,322)**

| **Product** | **Model 1** | **Model 2** |
| --- | --- | --- |
| **Bread and bakery products** | | |
| **Biscuits** | | |
| Original Crackers | Unhealthy | Unhealthy |
| Thin Rice & Corn Cakes | Healthy | Healthy |
| Tomato & Basil Wholegrain Crackers | Unhealthy | Unhealthy |
| Cheese Flavoured French Twists | Unhealthy | Unhealthy |
| Custard Cream Sandwich Biscuits | Unhealthy | Unhealthy |
| Chilli Charge Crackers | Unhealthy | Unhealthy |
| BBQ Ribs Blast Crackers | Unhealthy | Unhealthy |
| Coconut Cream Biscuits | Unhealthy | Unhealthy |
| The Original Biscuits | Unhealthy | Unhealthy |
| Mint Supremes | Unhealthy | Unhealthy |
| Mini Melting Moments Cookies | Unhealthy | Unhealthy |
| Macadamia Nut Brownies Biscuits | Unhealthy | Unhealthy |
| Apricot Coconut Flavour Fusion Breakfast Biscuits | Unhealthy | Unhealthy |
| Strawberry Baked Crispy Biscuits | Unhealthy | Unhealthy |
| Forest Fruit Flavour Crispy Biscuits | Unhealthy | Unhealthy |
| Black Rice Crackers with Sesame Flavour | Healthy | Healthy |
| Cheese Palmiers | Unhealthy | Unhealthy |
| Jamdrops Biscuits | Unhealthy | Unhealthy |
| Classic Christmas Trees Biscuits | Unhealthy | Unhealthy |
| Build Your Own Gingerbread Tree Kit | Unhealthy | Unhealthy |
| Dark Chocolate Half Coated Chunk & Hazelnut Biscuits | Unhealthy | Unhealthy |
| Cheese Flavoured Want Want Rice Crackers | Unhealthy | Unhealthy |
| Cookies & Cream Flavoured Cream Wafers | Unhealthy | Unhealthy |
| Rice Crackers | Unhealthy | Unhealthy |
| Australian Water Cracker Biscuits | Unhealthy | Unhealthy |
| Cornflake Biscuits | Unhealthy | Unhealthy |
| Sesame Delicate Rice Wafers | Unhealthy | Unhealthy |
| Rice Fine Wafer Crackers | Unhealthy | Unhealthy |
| Little Cookies | Unhealthy | Unhealthy |
| Chocolate Mint Flavoured Cream Wafers | Unhealthy | Unhealthy |
| Rosemary & Sea Salt Italian Bread Sticks | Unhealthy | Unhealthy |
| Cashew & Rosemary Specialty Crackers | Unhealthy | Unhealthy |
| Crispy Oat with a Hint of Honey Crackers | Unhealthy | Unhealthy |
| Lavosh Charcoal Crackers | Unhealthy | Unhealthy |
| Crisp Oat | Unhealthy | Unhealthy |
| Original Rice Cakes | Unhealthy | Healthy |
| Wafer and Chocolate Hazelnut Flavoured Cream | Unhealthy | Unhealthy |
| Kasha Toasted Buckwheat Light and Crunchy Crispbread | Healthy | Healthy |
| Rice Crackers with Sweet Carrot | Unhealthy | Unhealthy |
| Organic Original Black Rice Crackers | Healthy | Healthy |
| Fig & Pecan Speciality Crackers | Unhealthy | Unhealthy |
| Chocolate Café Style Cream Wafers | Unhealthy | Unhealthy |
| Cheese and Herbs Festive Puff Pastry Savoury Biscuits | Unhealthy | Unhealthy |
| Wholegrain Oat Biscuits with Almond Butter Filling | Unhealthy | Unhealthy |
| Mosaic Biscuit Filled with Cocoa Cream | Unhealthy | Unhealthy |
| Hundreds & Thousands White Choc Minis | Unhealthy | Unhealthy |
| Choc Chip Minis | Unhealthy | Unhealthy |
| Choc Chip Bickies | Unhealthy | Unhealthy |
| Cocktail Cheese Roughs | Unhealthy | Unhealthy |
| No Gluten Turmeric Wafer Crackers | Unhealthy | Unhealthy |
| Sea Salt & Apple Cider Vinegar Wholegrain Brown Rice Crackers | Unhealthy | Unhealthy |
| Sweet Chilli & Sour Cream Rice Crackers | Unhealthy | Unhealthy |
| Smokey BBQ Rice Crackers | Unhealthy | Unhealthy |
| Moroccan Spice Grain Free Crackers with Turmeric and Mozuku Seaweed | Healthy | Healthy |
| Organic Corn Cakes | Healthy | Healthy |
| Mini Cheese Flavour Thin Rice Crackers | Unhealthy | Unhealthy |
| No Gluten Cranberry & Pumpkin Seed Artisan Crackers | Unhealthy | Unhealthy |
| Salted Caramel Wafers | Unhealthy | Unhealthy |
| Rice Crackers with Seasoned Pumpkin | Unhealthy | Unhealthy |
| Double Choc Biscuits | Unhealthy | Unhealthy |
| Sea Salt Flavoured Protein Crackers | Unhealthy | Healthy |
| Paprika Brown Rice Crackers | Unhealthy | Unhealthy |
| Chicken Snack Blasts | Unhealthy | Unhealthy |
| Cadbury Coated Mint Cookies | Unhealthy | Unhealthy |
| Black Sesame Rice Crackers | Unhealthy | Unhealthy |
| Pure Butter Shortbread Highland Cows | Unhealthy | Unhealthy |
| Original Rice Crackers | Unhealthy | Unhealthy |
| Milk Rusk | Unhealthy | Unhealthy |
| Original Deliciously Seedy Crackers | Healthy | Healthy |
| Caramelised Onion Lavosh Crackers | Unhealthy | Unhealthy |
| Spelt & Pumpkin Seeds Norwegian Crispbread | Healthy | Healthy |
| Christmas Merrilossus Biscuits | Unhealthy | Unhealthy |
| Breadsticks from Piedmont with Olive Oil | Unhealthy | Unhealthy |
| Breadsticks from Piedmont with Rosemary | Unhealthy | Unhealthy |
| Hemp Seed & Black Pepper Crackers | Unhealthy | Unhealthy |
| Sour Cherry Cheesecake Cookie | Unhealthy | Unhealthy |
|  |  |  |
| Chocolate Chip Protein Cookie | Unhealthy | Unhealthy |
| Cookies & Cream Flavoured Thermogenic Low Carb Cookie for Women | Unhealthy | Unhealthy |
| Espresso Bite Size Wafer Cookies | Unhealthy | Unhealthy |
| Taralli with Fennel Seeds & Olive Oil | Unhealthy | Unhealthy |
| Wafers Layered with Cocoa Filling Covered in Milk Chocolate | Unhealthy | Unhealthy |
| Caraway 4 Seed Oat Crackers | Unhealthy | Unhealthy |
| Caramelised Onion & Balsamic Vinegar Premium Flavoured Rice Crackers | Unhealthy | Unhealthy |
| Aged Cheddar & Chives Premium Flavoured Rice Crackers | Unhealthy | Unhealthy |
| Organic 3 Grains Rice Cakes | Healthy | Healthy |
| Honey Bears | Unhealthy | Unhealthy |
| Vanilla & Chewy Choc Flavour Biscuits | Unhealthy | Unhealthy |
| Pizza Flavoured Biscuits | Unhealthy | Unhealthy |
| Gingerbread Babies | Unhealthy | Unhealthy |
| Original Rice Crackers | Unhealthy | Unhealthy |
| Cheese & Onion Rice Crackers | Unhealthy | Healthy |
| Sea Salt Linguette | Unhealthy | Unhealthy |
| Table Water Crackers | Unhealthy | Unhealthy |
| Soy & Ginger Flavour Rice Crackers | Unhealthy | Unhealthy |
| Chocolate Biscuits | Unhealthy | Unhealthy |
| Tasty Cheese Flavour Rice Crackers | Unhealthy | Unhealthy |
| Sour Cream & Chives Flavoured Rice Crackers | Unhealthy | Unhealthy |
| Sundried Tomato and Black Olive Mediterranean Savoury Crisps | Unhealthy | Unhealthy |
| Spiced Brandy Butter Cookies | Unhealthy | Unhealthy |
| Kale Artisan Crackers | Unhealthy | Unhealthy |
| Biscuit, savoury, from wheat flour, crispbread, puffed & toasted | Unhealthy | Unhealthy |
| Biscuit, savoury, from wheat flour, crispbread, puffed & toasted, reduced fat | Unhealthy | Unhealthy |
| Biscuit, savoury, from white wheat flour, plain snack cracker style, reduced fat | Unhealthy | Unhealthy |
| Biscuit, savoury, from white wheat flour, Salada style | Unhealthy | Unhealthy |
| Biscuit, savoury, from white wheat flour, Salada style, reduced fat | Unhealthy | Unhealthy |
| Biscuit, savoury, from white wheat flour, wafer style | Unhealthy | Unhealthy |
| Biscuit, savoury, from white wheat flour, water cracker style | Unhealthy | Unhealthy |
| Biscuit, savoury, from wholemeal wheat flour | Unhealthy | Unhealthy |
| Biscuit, savoury, from wholemeal wheat flour, added grains | Unhealthy | Unhealthy |
| Biscuit, savoury, from wholemeal wheat flour, crispbread | Unhealthy | Healthy |
| Biscuit, savoury, from wholemeal wheat flour, Salada style, high fibre | Unhealthy | Unhealthy |
| Biscuit, savoury, from wholemeal wheat flour & rye flour, crispbread, puffed | Unhealthy | Healthy |
| Biscuit, savoury, not further defined | Unhealthy | Unhealthy |
| Biscuit, savoury, from white wheat flour, cheese-flavoured | Unhealthy | Unhealthy |
| Biscuit, savoury, from white wheat flour, other flavours | Unhealthy | Unhealthy |
| Biscuit, savoury, from white wheat flour, flaky cracker style | Unhealthy | Unhealthy |
| Biscuit, savoury, from white wheat flour, flatbread style | Unhealthy | Unhealthy |
| Biscuit, savoury, from white wheat flour, plain snack cracker style | Unhealthy | Unhealthy |
| Biscuit, savoury, from white wheat flour, stick, salted | Unhealthy | Unhealthy |
| Biscuit, savoury, from white wheat flour, plain, not further defined | Unhealthy | Unhealthy |
| Biscuit, savoury, from rye flour, crispbread | Unhealthy | Unhealthy |
| Biscuit, savoury, from rye flour, crispbread, with added grains (buckwheat, linseed, soy, sesame) | Unhealthy | Unhealthy |
| Biscuit, savoury, rice cake, from brown rice, plain | Unhealthy | Healthy |
| Biscuit, savoury, rice cake, from brown rice, flavoured | Unhealthy | Unhealthy |
| Biscuit, savoury, rice cake, from brown rice, carob coated | Unhealthy | Unhealthy |
| Biscuit, savoury, rice cracker, plain | Unhealthy | Unhealthy |
| Biscuit, savoury, rice cracker, cheese based flavours | Unhealthy | Unhealthy |
| Biscuit, savoury, rice cracker, seaweed flavoured | Unhealthy | Unhealthy |
| Biscuit, savoury, rice cracker, sour cream based flavours | Unhealthy | Unhealthy |
| Biscuit, savoury, rice cracker, other flavours | Unhealthy | Unhealthy |
| Biscuit, savoury, rice cracker, from brown rice, all flavours | Unhealthy | Unhealthy |
| Biscuit, savoury, rice cracker, not further defined | Unhealthy | Unhealthy |
| Biscuit, savoury, rice flour, peanut cracker | Unhealthy | Unhealthy |
| Biscuit, savoury, corn cake, plain, salted | Unhealthy | Unhealthy |
| Biscuit, savoury, corn cake, multigrain, salted | Unhealthy | Healthy |
| Biscuit, savoury, corn, crispbread, puffed & toasted | Unhealthy | Unhealthy |
| Biscuit, savoury, rice cake, from brown rice, carob coated | Unhealthy | Unhealthy |
| Biscuit, savoury, rice cracker, plain | Unhealthy | Unhealthy |
| Biscuit, savoury, rice cracker, cheese based flavours | Unhealthy | Unhealthy |
| Biscuit, savoury, rice cracker, seaweed flavoured | Unhealthy | Unhealthy |
| Biscuit, savoury, rice cracker, sour cream based flavours | Unhealthy | Unhealthy |
| Biscuit, savoury, rice cracker, other flavours | Unhealthy | Unhealthy |
| Biscuit, savoury, rice cracker, from brown rice, all flavours | Unhealthy | Unhealthy |
| Biscuit, savoury, rice cracker, not further defined | Unhealthy | Unhealthy |
| Biscuit, savoury, rice flour, peanut cracker | Unhealthy | Unhealthy |
| Biscuit, savoury, corn cake, plain, salted | Unhealthy | Unhealthy |
| Biscuit, savoury, corn cake, multigrain, salted | Unhealthy | Healthy |
| Biscuit, savoury, corn, crispbread, puffed & toasted | Unhealthy | Unhealthy |
| **Bread** | | |
| Croutons with Garlic & Herbs | Unhealthy | Unhealthy |
| Lite White Soft Wraps | Unhealthy | Unhealthy |
| Seven Grain Sourdough Bread | Unhealthy | Unhealthy |
| White Wraps | Unhealthy | Unhealthy |
| Chickpeas Quinoa & Beetroot Organic Wraps | Unhealthy | Unhealthy |
| Semi-Dried Tomato & Mozzarella Bread | Unhealthy | Unhealthy |
| Multi Fruit Sourdough Loaf | Unhealthy | Healthy |
| De Luxe Brioche | Unhealthy | Unhealthy |
| Turkish Rolls | Healthy | Healthy |
| Stone Baked White Batard | Healthy | Healthy |
| Mixed Seeds Gluten Free Bread | Unhealthy | Unhealthy |
| 6 Grain & Rustic Country Wholegrain Sliced Bread | Unhealthy | Unhealthy |
| Low GI Wraps | Unhealthy | Unhealthy |
| Pane Di Casa Sliced Bread | Unhealthy | Healthy |
| Bread Bites | Unhealthy | Unhealthy |
| Matcha Buns | Unhealthy | Unhealthy |
| Country Grains | Unhealthy | Unhealthy |
| Traditional White Lebanese Bread | Healthy | Healthy |
| Wholemeal & Seeds Bread | Unhealthy | Unhealthy |
| Soy-Lin Bread | Unhealthy | Unhealthy |
| Plain Puppodums | Unhealthy | Unhealthy |
| Original Organic Sourdough Bread | Unhealthy | Unhealthy |
| Olive Oil, Herb & Garlic Sliced Bread | Unhealthy | Unhealthy |
| Chandni Chowk Plain Naan | Healthy | Healthy |
| High Fibre White Sandwich Loaf | Unhealthy | Unhealthy |
| Olive Oil & Herb Bread | Unhealthy | Unhealthy |
| Crumpets | Unhealthy | Unhealthy |
| Original Turkish Bread | Unhealthy | Unhealthy |
| High Fibre Sandwich Bread | Unhealthy | Unhealthy |
| Sliced Brioche | Unhealthy | Unhealthy |
| Garlic Bread | Unhealthy | Unhealthy |
| White Sandwich Bread | Unhealthy | Unhealthy |
| Tasty Cheese & Bacon Flatbread | Unhealthy | Unhealthy |
| Sliced White Toast | Unhealthy | Healthy |
| Indian Style Roti Paratha | Unhealthy | Unhealthy |
| Traditional Sweet Loaf | Unhealthy | Unhealthy |
| Original Burger Thins | Unhealthy | Unhealthy |
| Bagel, from white flour, commercial | Unhealthy | Unhealthy |
| Bagel, from white flour, commercial, toasted | Unhealthy | Unhealthy |
| Breadcrumbs, white | Unhealthy | Unhealthy |
| Bread, damper, from white flour, commercial | Unhealthy | Unhealthy |
| Bread, damper, from white flour, commercial, toasted | Unhealthy | Unhealthy |
| Bread, focaccia, plain, commercial | Unhealthy | Unhealthy |
| Bread, focaccia, plain, commercial, toasted | Unhealthy | Unhealthy |
| Bread, from spelt flour, commercial | Unhealthy | Unhealthy |
| Bread, from spelt flour, commercial, toasted | Unhealthy | Unhealthy |
| Bread, from white flour, commercial | Unhealthy | Unhealthy |
| Bread, from white flour, commercial, toasted | Unhealthy | Unhealthy |
| Bread, from white flour, commercial, low GI | Unhealthy | Unhealthy |
| Bread, from white flour, commercial, low GI, toasted | Unhealthy | Unhealthy |
| Bread, from white flour, chia seeds, commercial | Healthy | Healthy |
| Bread, from white flour, chia seeds, commercial, toasted | Unhealthy | Unhealthy |
| Bread, from white flour, ciabatta, commercial | Unhealthy | Unhealthy |
| Bread, from white flour, ciabatta, commercial, toasted | Unhealthy | Unhealthy |
| Bread, from white flour, French stick or baguette, commercial | Unhealthy | Unhealthy |
| Bread, from white flour, French stick or baguette, commercial, toasted | Unhealthy | Unhealthy |
| Bread, from white flour, pane di casa, commercial | Unhealthy | Unhealthy |
| Bread, from white flour, pane di casa, commercial, toasted | Unhealthy | Unhealthy |
| Bread, from white flour, sour dough, commercial | Unhealthy | Unhealthy |
| Bread, from white flour, sour dough, commercial, toasted | Unhealthy | Unhealthy |
| Bread roll, from white flour, commercial | Unhealthy | Unhealthy |
| Bread roll, from white flour, commercial, toasted | Unhealthy | Unhealthy |
| Bread roll, from white flour, fast food style | Unhealthy | Unhealthy |
| Bread or bread roll, Turkish, plain, commercial | Unhealthy | Unhealthy |
| Bread or bread roll, Turkish, plain, commercial, toasted | Unhealthy | Unhealthy |
| Bread, from white flour, commercial, added calcium | Unhealthy | Unhealthy |
| Bread, from white flour, commercial, added calcium, toasted | Unhealthy | Unhealthy |
| Bread, from white flour, commercial, added fibre | Unhealthy | Unhealthy |
| Bread, from white flour, commercial, added fibre, toasted | Unhealthy | Unhealthy |
| Bread, from white flour, commercial, added fibre & vitamins B1, B3, B6 & E, Fe & Zn | Unhealthy | Unhealthy |
| Bread, from white flour, commercial, added fibre & vitamins B1, B3, B6 & E, Fe & Zn, toasted | Unhealthy | Unhealthy |
| Bread, from white flour, commercial, added iron | Unhealthy | Unhealthy |
| Bread, from white flour, commercial, added iron, toasted | Unhealthy | Unhealthy |
| Bread, from white flour, commercial, added omega-3 polyunsaturates | Unhealthy | Unhealthy |
| Bread, from white flour, commercial, added omega-3 polyunsaturates, toasted | Unhealthy | Unhealthy |
| Bread, from white Jackaroo flour, commercial, added vitamins B1 & folate & Fe | Unhealthy | Unhealthy |
| Bread, from white Jackaroo flour, commercial, added vitamins B1 & folate & Fe, toasted | Unhealthy | Unhealthy |
| Bread, flat, injera | Healthy | Healthy |
| Bread, from white flour, organic, commercial | Unhealthy | Unhealthy |
| Bread, from white flour, organic, commercial, toasted | Unhealthy | Unhealthy |
| Bread, from white flour, commercial, fresh, not further defined | Unhealthy | Unhealthy |
| Bread, from white flour, commercial, toasted, not further defined | Unhealthy | Unhealthy |
| Bread, from white flour, for homemade sandwiches | Unhealthy | Unhealthy |
| Bread, from white flour, for homemade sandwiches, toasted | Unhealthy | Unhealthy |
| Bread, from white or wholemeal flour, organic, commercial | Unhealthy | Unhealthy |
| Bread, from white or wholemeal flour, organic, commercial, toasted | Unhealthy | Unhealthy |
| Bread, Naan, commercial | Unhealthy | Unhealthy |
| Bread, paratha | Healthy | Healthy |
| Bread, pizza base, commercial | Unhealthy | Unhealthy |
| Bread, pizza base, thick base, fast food-style | Unhealthy | Unhealthy |
| Bread, pizza base, thin base, fast food-style | Unhealthy | Unhealthy |
| Bread, pumpkin | Healthy | Healthy |
| Bread, pumpkin, toasted | Healthy | Healthy |
| Bread, from white flour, for homemade sandwiches | Unhealthy | Unhealthy |
| Bread, from white flour, for homemade sandwiches, toasted | Unhealthy | Unhealthy |
| Bread, mixed grain, commercial | Unhealthy | Unhealthy |
| Bread, mixed grain, commercial, toasted | Unhealthy | Unhealthy |
| Bread, mixed grain, added seeds, commercial | Healthy | Healthy |
| Bread, mixed grain, added seeds, commercial, toasted | Unhealthy | Unhealthy |
| Bread, mixed grain, extra grainy & seeds, added pumpkin seeds, commercial | Healthy | Healthy |
| Bread, mixed grain, extra grainy & seeds, added pumpkin seeds, commercial, toasted | Healthy | Healthy |
| Bread, mixed grain, French stick or baguette, commercial | Unhealthy | Unhealthy |
| Bread, mixed grain, French stick or baguette, commercial, toasted | Unhealthy | Unhealthy |
| Bread, mixed grain, commercial, fresh, not further defined | Healthy | Healthy |
| Bread, mixed grain, commercial, toasted, not further defined | Unhealthy | Unhealthy |
| Bread, mixed grain, for sandwiches purchased from a cafe, takeaway shop or canteen | Healthy | Healthy |
| Bread, mixed grain, for sandwiches purchased from a cafe, takeaway shop or canteen, toasted | Unhealthy | Unhealthy |
| Bread, from white flour, extra grainy & seeds, commercial | Healthy | Healthy |
| Bread, from white flour, extra grainy & seeds, commercial, toasted | Unhealthy | Unhealthy |
| Bread, soy & linseed, commercial | Healthy | Healthy |
| Bread, soy & linseed, commercial, toasted | Unhealthy | Unhealthy |
| Bread roll, mixed grain, commercial | Unhealthy | Unhealthy |
| Bread roll, mixed grain, commercial, toasted | Unhealthy | Unhealthy |
| Bread, from white or wholemeal flour, organic, added grains, commercial | Healthy | Healthy |
| Bread, from white or wholemeal flour, organic, added grains, commercial, toasted | Unhealthy | Unhealthy |
| Bread, mixed grain, for homemade sandwiches | Healthy | Healthy |
| Bread, mixed grain, for homemade sandwiches, toasted | Unhealthy | Unhealthy |
| Bread, damper, from wholemeal flour, commercial | Unhealthy | Unhealthy |
| Bread, damper, from wholemeal flour, commercial, toasted | Unhealthy | Unhealthy |
| Bread, from wholemeal flour, commercial | Unhealthy | Unhealthy |
| Bread, from wholemeal flour, commercial, toasted | Unhealthy | Unhealthy |
| Bread, from wholemeal flour, added seeds, commercial | Healthy | Healthy |
| Bread, from wholemeal flour, added seeds, commercial, toasted | Unhealthy | Unhealthy |
| Bread, from wholemeal flour, mixed grain & seeds, commercial | Healthy | Healthy |
| Bread, from wholemeal flour, mixed grain & seeds, commercial, toasted | Unhealthy | Unhealthy |
| Bread, from wholemeal flour, mixed grain, with quinoa & flaxseeds, commercial | Healthy | Healthy |
| Bread, from wholemeal flour, mixed grain, with quinoa & flaxseeds, commercial, toasted | Unhealthy | Unhealthy |
| Bread, from wholemeal flour, extra grainy, four seeds, commercial | Healthy | Healthy |
| Bread, from wholemeal flour, extra grainy, four seeds, commercial, toasted | Healthy | Healthy |
| Bread, from wholemeal flour, extra grainy & seeds, commercial | Healthy | Healthy |
| Bread, from wholemeal flour, extra grainy & seeds, commercial, toasted | Unhealthy | Unhealthy |
| Bread, from wholemeal flour, extra grainy & seeds, added oats, commercial | Healthy | Healthy |
| Bread, from wholemeal flour, extra grainy & seeds, added oats, commercial, toasted | Healthy | Healthy |
| Bread, from wholemeal flour, sour dough, commercial | Unhealthy | Unhealthy |
| Bread, from wholemeal flour, sour dough, commercial, toasted | Unhealthy | Unhealthy |
| Bread, from white & wholemeal flour, commercial | Healthy | Healthy |
| Bread, from white & wholemeal flour, commercial, toasted | Unhealthy | Unhealthy |
| Bread roll, from wholemeal flour, commercial | Unhealthy | Unhealthy |
| Bread roll, from wholemeal flour, commercial, toasted | Unhealthy | Unhealthy |
| Bread, from wholemeal flour, commercial, added fibre | Unhealthy | Unhealthy |
| Bread, from wholemeal flour, commercial, added fibre, toasted | Unhealthy | Unhealthy |
| Bread, from wholemeal flour, commercial, added fibre & vitamins B1, B3, B6 & E, Fe & Zn | Unhealthy | Unhealthy |
| Bread, from wholemeal flour, commercial, added fibre & vitamins B1, B3, B6 & E, Fe & Zn, toasted | Unhealthy | Unhealthy |
| Bread, from wholemeal flour, commercial, added iron | Unhealthy | Unhealthy |
| Bread, from wholemeal flour, commercial, added iron, toasted | Unhealthy | Unhealthy |
| Bread, from wholemeal flour, commercial, added omega-3 polyunsaturates | Unhealthy | Unhealthy |
| Bread, from wholemeal flour, commercial, added omega-3 polyunsaturates, toasted | Unhealthy | Unhealthy |
| Bread, from wholemeal flour, organic, commercial | Healthy | Healthy |
| Bread, from wholemeal flour, organic, commercial, toasted | Unhealthy | Unhealthy |
| Bread, from wholemeal flour, commercial, fresh, not further defined | Unhealthy | Unhealthy |
| Bread, from wholemeal flour, commercial, toasted, not further defined | Unhealthy | Unhealthy |
| Bread, from wholemeal flour, for homemade sandwiches | Unhealthy | Unhealthy |
| Bread, from wholemeal flour, for homemade sandwiches, toasted | Unhealthy | Unhealthy |
| Bread, from rye flour, dark, commercial | Unhealthy | Unhealthy |
| Bread, from rye flour, dark, commercial, toasted | Unhealthy | Unhealthy |
| Bread, from rye flour, light, commercial | Unhealthy | Unhealthy |
| Bread, from rye flour, light, commercial, toasted | Unhealthy | Unhealthy |
| Bread, from rye flour, sour dough, commercial | Unhealthy | Unhealthy |
| Bread, from rye flour, sour dough, commercial, toasted | Unhealthy | Unhealthy |
| Bread, from rye flour, added grains, commercial | Healthy | Healthy |
| Bread, from rye flour, added grains, commercial, toasted | Unhealthy | Unhealthy |
| Bread, pumpernickel, commercial | Unhealthy | Unhealthy |
| Bread, pumpernickel, commercial, toasted | Unhealthy | Unhealthy |
| Bread, from rye flour, organic, commercial | Healthy | Healthy |
| Bread, from rye flour, organic, commercial, toasted | Unhealthy | Unhealthy |
| Bread, gluten free, commercial | Unhealthy | Unhealthy |
| Bread, gluten free, commercial, toasted | Unhealthy | Unhealthy |
| Bread, gluten free, with added grains, commercial | Unhealthy | Unhealthy |
| Bread, gluten free, with added grains, commercial, toasted | Unhealthy | Unhealthy |
| Bread, corn | Healthy | Healthy |
| Bread, commercial, fresh, not further defined | Unhealthy | Unhealthy |
| Bread, commercial, toasted, not further defined | Unhealthy | Unhealthy |
| Bread roll, commercial, fresh, not further defined | Unhealthy | Unhealthy |
| Bread roll, for use in hamburger or hot dog recipes | Unhealthy | Unhealthy |
| Bread roll, for use in subway sandwich recipes | Unhealthy | Unhealthy |
| Bread, tortilla, for use in Mexican recipes | Unhealthy | Unhealthy |
| Bread, fresh, not further defined | Unhealthy | Unhealthy |
| Bread, toasted, not further defined | Unhealthy | Unhealthy |
| Muffin, English style, from white flour, commercial, fresh/untoasted | Unhealthy | Unhealthy |
| Muffin, English style, from white flour, commercial, toasted | Unhealthy | Unhealthy |
| Muffin, English style, from white flour, commercial, added dried fruit, toasted | Unhealthy | Unhealthy |
| Muffin, English style, from wholemeal flour, commercial, toasted | Unhealthy | Unhealthy |
| Muffin, English style, mixed grain, commercial, toasted | Unhealthy | Unhealthy |
| Bread, chapatti, commercial | Healthy | Healthy |
| Bread, flat (pita or Lebanese), white, commercial | Unhealthy | Unhealthy |
| Bread, flat (pita or Lebanese), white, commercial, toasted | Unhealthy | Unhealthy |
| Bread, flat (pita or Lebanese), wholemeal, commercial | Unhealthy | Unhealthy |
| Bread, flat (pita or Lebanese), wholemeal, commercial, toasted | Unhealthy | Unhealthy |
| Bread, flat wrap, white, commercial | Unhealthy | Unhealthy |
| Bread, flat wrap, white, commercial, toasted | Unhealthy | Unhealthy |
| Bread, flat wrap or tortilla, mixed grain | Unhealthy | Unhealthy |
| Bread, flat wrap or tortilla, mixed grain, toasted | Unhealthy | Unhealthy |
| Bread, flat wrap or tortilla, wholemeal, commercial | Unhealthy | Unhealthy |
| Bread, flat wrap or tortilla, wholemeal, commercial, toasted | Unhealthy | Unhealthy |
| Bread, Roti, commercial | Healthy | Healthy |
| Bread, tortilla, white, commercial | Unhealthy | Unhealthy |
| Bread, tortilla, white, commercial, toasted | Unhealthy | Unhealthy |
| Bread, flat, not further defined, commercial | Unhealthy | Unhealthy |
| Bread, flat, not further defined, commercial, toasted | Unhealthy | Unhealthy |
| Bread, flat wrap, corn, commercial | Healthy | Healthy |
| Bread, flat wrap, corn, commercial, toasted | Unhealthy | Unhealthy |
| Bread, flat wrap or tortilla, rice, commercial | Unhealthy | Unhealthy |
| Bread, flat wrap or tortilla, rye, commercial | Unhealthy | Unhealthy |
| Bread, flat wrap or tortilla, rye, commercial, toasted | Unhealthy | Unhealthy |
| Bread, tortilla, corn, commercial | Unhealthy | Unhealthy |
| Bread, tortilla, corn, commercial, toasted | Unhealthy | Unhealthy |
| Bagel, fruit, commercial | Unhealthy | Unhealthy |
| Bagel, fruit, commercial, toasted | Unhealthy | Unhealthy |
| Bread, from wheat flour, commercial, added dried fruit | Unhealthy | Unhealthy |
| Bread, from wheat flour, commercial, added dried fruit, toasted | Unhealthy | Unhealthy |
| Bread, garlic or herb, commercial, cooked | Unhealthy | Unhealthy |
| Crumpet, from white flour, commercial | Unhealthy | Unhealthy |
| Crumpet, from white flour, commercial, toasted | Unhealthy | Unhealthy |
| Crumpet, from wholemeal flour, commercial, toasted | Unhealthy | Unhealthy |
| Butter, plain, salted | Healthy | Healthy |
| Pappadam, raw | Unhealthy | Unhealthy |
| Pappadam, deep fried | Unhealthy | Unhealthy |
| Pappadam, microwaved without oil or salt | Unhealthy | Unhealthy |
| **Cakes, muffins and pastries** | | |
| Chocolate & Hazelnut Cake | Unhealthy | Unhealthy |
| Toffee Apple Pudding | Unhealthy | Unhealthy |
| Pancake Shake Chocolate Mix | Unhealthy | Unhealthy |
| Banana Coconut Muffin Mix | Unhealthy | Unhealthy |
| Raspberry and Chestnut Muffin Mix | Unhealthy | Unhealthy |
| Lemon Coconut Crumble Cake | Unhealthy | Unhealthy |
| Reduced Fat Chocolate Fudge Brownie Mix | Unhealthy | Unhealthy |
| Steamy Puds Chocolate | Unhealthy | Unhealthy |
| Profiteroles Mix | Unhealthy | Unhealthy |
| Princess Carriage Cup Cake Kit | Unhealthy | Unhealthy |
| Strawberry & Cream Cupcake Mix with Icing | Unhealthy | Unhealthy |
| Banana & Cream Cheese Cupcake Mix with Icing | Unhealthy | Unhealthy |
| Chocolate Donut | Unhealthy | Unhealthy |
| Jam Ball | Unhealthy | Unhealthy |
| Blueberry Danish | Unhealthy | Unhealthy |
| Iced Donuts | Unhealthy | Unhealthy |
| Cinnamon Crumble Muffin Mix | Unhealthy | Unhealthy |
| Toblerone Chocolate Cake | Unhealthy | Unhealthy |
| Premium Apple Crunch | Unhealthy | Unhealthy |
| Sponge Cakes with Fat-Reduced Cocoa Filling and Coating | Unhealthy | Unhealthy |
| Profiterole Dessert | Unhealthy | Unhealthy |
| Raspberry Tartlets | Unhealthy | Unhealthy |
| Shortbread Mix | Unhealthy | Unhealthy |
| Triple Berry Delights | Unhealthy | Unhealthy |
| Brandy Snap Birthday Puds with Toffee Sauce | Unhealthy | Unhealthy |
| M&M's Minis Chewy Cookie Dough | Unhealthy | Unhealthy |
| Pecan & Walnut Tart | Unhealthy | Unhealthy |
| Rich Chocolate Mug Cake Mix | Unhealthy | Unhealthy |
| Lemon Meringues | Unhealthy | Unhealthy |
| Chocolate Flavoured Cake Mix | Unhealthy | Unhealthy |
| Pikelets Mix | Unhealthy | Unhealthy |
| Sticky Date Flavoured Cake Mix | Unhealthy | Unhealthy |
| 25% Reduced Fat Puff Pastry | Unhealthy | Unhealthy |
| Dessert Pudding Log with Chocolate | Unhealthy | Unhealthy |
| Christmas Tree Brownie Mix | Unhealthy | Unhealthy |
| Butter Cake Mix | Unhealthy | Unhealthy |
| Choc Rainbow Space Balls Mix | Unhealthy | Unhealthy |
| Limited Edition Morello Cherry Brownie Mix | Unhealthy | Unhealthy |
| Vanilla Cake Mix | Unhealthy | Unhealthy |
| Baklava | Unhealthy | Unhealthy |
| Chocolate Puddings with Chocolate Sauce | Unhealthy | Unhealthy |
| Dutch Crispbakes Toast | Unhealthy | Unhealthy |
| Apple Rounds | Unhealthy | Unhealthy |
| Rich Chocolate & Raspberry Sauced Pudding | Unhealthy | Unhealthy |
| Milano Panettone Cake with Raisins and Candied Fruits | Unhealthy | Unhealthy |
| Sticky Fig & Ginger Rice Pudding Mix | Unhealthy | Unhealthy |
| Gluten Free Waffle Cones | Unhealthy | Unhealthy |
| Ultimate 40% Choc Brownie | Unhealthy | Unhealthy |
| Choc Salted Caramel Brownie Mix | Unhealthy | Unhealthy |
| Banana Bread Baking Pre-Mix | Unhealthy | Unhealthy |
| Mocha Cake | Unhealthy | Unhealthy |
| Apple & Spice Pancake Mix | Unhealthy | Unhealthy |
| Angel Slices | Unhealthy | Unhealthy |
| Tiramisu | Unhealthy | Unhealthy |
| Apple & Butterscotch Crumbles | Unhealthy | Unhealthy |
| Caramel Crowns Inspired Brownies | Unhealthy | Unhealthy |
| Vanilla & Coconut Flavoured Bliss Balls Mix | Unhealthy | Unhealthy |
| Jam Sponge Roll | Unhealthy | Unhealthy |
| Father’s Day Chocolate Cup Cake Kit with Icing and Decorations | Unhealthy | Unhealthy |
| Buttermilk Pancake Shake Mix | Unhealthy | Unhealthy |
| Onion & Chives Puff Pastry Twists | Unhealthy | Unhealthy |
| Chocolate Chip Cookies Mix | Unhealthy | Unhealthy |
| Banana Muffin Bars | Unhealthy | Unhealthy |
| Bite Sized Choc Mint Cake Balls | Unhealthy | Unhealthy |
| Soan Cake | Unhealthy | Unhealthy |
| Traditional Christmas Pudding | Unhealthy | Unhealthy |
| Baked Wafer Cannoli | Unhealthy | Unhealthy |
| Cheese & Chive Scone Mix | Unhealthy | Unhealthy |
| Deep Dish Saucy Double Chocolate Pudding | Unhealthy | Unhealthy |
| Panettone Classico | Unhealthy | Unhealthy |
| Raspberry Roulade | Unhealthy | Unhealthy |
| Coconut & Vanilla Flavour Muesli Bar Mix | Unhealthy | Healthy |
| I'm a Guilt Free Caramel Spice Donut | Unhealthy | Unhealthy |
| Glutinous Peanut Rice Balls | Unhealthy | Unhealthy |
| Gluten Free Muffin Mix with Teff | Unhealthy | Unhealthy |
| Baked Choc Donut Balls Mix | Unhealthy | Unhealthy |
| Australian Flour Vanilla Cake Mix | Unhealthy | Unhealthy |
| Peppa Pig Choc Muddy Puddles Pancake Shake | Unhealthy | Unhealthy |
| Cookies & Cream Cupcakes Mix | Unhealthy | Unhealthy |
| Molten Puddings | Unhealthy | Unhealthy |
| Low Sugar Rich Chocolate Cake Mix | Unhealthy | Unhealthy |
| Tartelettes Apéro | Unhealthy | Unhealthy |
| Mini Blueberry Pies | Unhealthy | Unhealthy |
| Strawberries & Cream Protein Ball Mix | Unhealthy | Unhealthy |
| Honey Cake Bars | Unhealthy | Unhealthy |
| Chocolate Mud Cake | Unhealthy | Unhealthy |
| Original Buckwheat Pancake Mix | Unhealthy | Unhealthy |
| Vanilla Cake Mix | Unhealthy | Unhealthy |
| Decadent Chocolate Cake Mix | Unhealthy | Unhealthy |
| Vanilla Flavoured Cake Mix | Unhealthy | Unhealthy |
| Panettoncino Classico | Unhealthy | Unhealthy |
| Sliced Chocolate Chip Brioche Loaf | Unhealthy | Unhealthy |
| Banana Caramel Tart | Unhealthy | Unhealthy |
| Fruit Pudding | Unhealthy | Unhealthy |
| Vampire Fancies | Unhealthy | Unhealthy |
| Ginger & Apricot Cake Slices | Unhealthy | Unhealthy |
| Stollen Bites | Unhealthy | Unhealthy |
| Pandoro | Unhealthy | Unhealthy |
| Traditional Pandoro | Unhealthy | Unhealthy |
| Tiramisu Premium Cake Mix | Unhealthy | Unhealthy |
| Bun, sweet, chocolate or with chocolate chips, uniced | Unhealthy | Unhealthy |
| Bun, sweet, with sultanas, uniced | Unhealthy | Unhealthy |
| Bun, sweet, with fruit (other than sultanas), uniced | Unhealthy | Unhealthy |
| Panettone | Unhealthy | Unhealthy |
| Bun, sweet, steamed bun, filled with bean paste | Unhealthy | Unhealthy |
| Bun, sweet, sticky cinnamon, with icing | Unhealthy | Unhealthy |
| Bun, sweet, with custard, iced | Unhealthy | Unhealthy |
| Bun, sweet, with sultanas, iced | Unhealthy | Unhealthy |
| Bun, sweet, with fruit (other than sultanas), iced | Unhealthy | Unhealthy |
| Bun, sweet, with fruit (other than sultanas) & nuts, iced | Unhealthy | Unhealthy |
| Bun, sweet, with mock cream & jam, uniced | Unhealthy | Unhealthy |
| Bun, sweet, with taro | Unhealthy | Unhealthy |
| Bun, sweet, not further defined | Unhealthy | Unhealthy |
| Breakfast pastry, sweet, fruit-paste filled, commercial | Unhealthy | Unhealthy |
| Cake mix, chocolate flavour, dry powder | Unhealthy | Unhealthy |
| Cake or cupcake, black forest (chocolate-cherry), layered, cream-filled, commercial | Unhealthy | Unhealthy |
| Cake or cupcake, chocolate, commercial, uniced | Unhealthy | Unhealthy |
| Cake or cupcake, chocolate, commercial, uniced, filled with cream | Unhealthy | Unhealthy |
| Cake or cupcake, chocolate, commercial, sugar based icing | Unhealthy | Unhealthy |
| Cake or cupcake, chocolate, commercial, sugar based icing, filled with cream | Unhealthy | Unhealthy |
| Cake or cupcake, mud, caramel or white chocolate, uniced | Unhealthy | Unhealthy |
| Cake or cupcake, mud, caramel or white chocolate, sugar based icing | Unhealthy | Unhealthy |
| Cake or cupcake, mud, dark chocolate, commercial, chocolate ganache icing | Unhealthy | Unhealthy |
| Cake mix, sponge, dry powder | Unhealthy | Unhealthy |
| Cake or cupcake, sponge, plain, commercial, uniced, unfilled | Unhealthy | Unhealthy |
| Cake or cupcake, sponge, plain, commercial, uniced, filled with cream | Unhealthy | Unhealthy |
| Cake or cupcake, sponge, plain, commercial, uniced, filled with jam | Unhealthy | Unhealthy |
| Cake or cupcake, sponge, plain, commercial, uniced, filled with jam & cream | Unhealthy | Unhealthy |
| Cake or cupcake, sponge, plain, commercial, iced, unfilled | Unhealthy | Unhealthy |
| Cake or cupcake, sponge, plain, commercial, iced, filled with cream | Unhealthy | Unhealthy |
| Cake or cupcake, sponge, plain, uniced, filled with fruit & cream | Unhealthy | Unhealthy |
| Cake or cupcake, sponge, chocolate flavoured, commercial, uniced, filled with jam & cream | Unhealthy | Unhealthy |
| Cake or cupcake, sponge, chocolate flavoured, commercial, iced, filled with cream | Unhealthy | Unhealthy |
| Cake, lamington, unfilled | Unhealthy | Unhealthy |
| Cake, lamington, filled with jam &/or cream | Unhealthy | Unhealthy |
| Bread, banana, commercial | Unhealthy | Unhealthy |
| Bread, banana, commercial, toasted | Unhealthy | Unhealthy |
| Cake mix, plain, dry powder | Unhealthy | Unhealthy |
| Cake mix, orange & poppy seed, dry powder | Unhealthy | Unhealthy |
| Cake or cupcake, banana, commercial, uniced | Unhealthy | Unhealthy |
| Cake or cupcake, banana, commercial, iced | Unhealthy | Unhealthy |
| Cake or cupcake, berry, undefined fat, uniced | Unhealthy | Unhealthy |
| Cake or cupcake, berry, reduced fat, commercial, uniced | Unhealthy | Unhealthy |
| Cake or cupcake, caramel, uniced | Unhealthy | Unhealthy |
| Cake or cupcake, caramel, iced | Unhealthy | Unhealthy |
| Cake or cupcake, carrot, commercial, uniced | Unhealthy | Unhealthy |
| Cake or cupcake, carrot, commercial, iced | Unhealthy | Unhealthy |
| Cake or cupcake, carrot, commercial, iced, filled with cream | Unhealthy | Unhealthy |
| Cake or cupcake, citrus, commercial, uniced | Unhealthy | Unhealthy |
| Cake or cupcake, citrus, commercial, iced | Unhealthy | Unhealthy |
| Cake or cupcake, coconut, commercial or homemade, uniced | Unhealthy | Unhealthy |
| Cake or cupcake, date loaf, commercial, uniced | Unhealthy | Unhealthy |
| Cake or cupcake, date loaf, commercial or homemade, undefined fat, iced | Unhealthy | Unhealthy |
| Cake, friand, commercial, uniced | Unhealthy | Unhealthy |
| Cake, fruit, dark in colour, commercial, uniced | Unhealthy | Unhealthy |
| Cake, fruit, light in colour, commercial, uniced | Unhealthy | Unhealthy |
| Cake, fruit, commercial, uniced | Unhealthy | Unhealthy |
| Cake, fruit, commercial, reduced fat, uniced | Unhealthy | Unhealthy |
| Cake, fruit, commercial, iced | Unhealthy | Unhealthy |
| Cake or cupcake, orange & almond, commercial, uniced | Unhealthy | Unhealthy |
| Cake or cupcake, plain butter cake, commercial, uniced | Unhealthy | Unhealthy |
| Cake or cupcake, plain butter cake, commercial, iced | Unhealthy | Unhealthy |
| Cake, tea cake, commercial, uniced | Unhealthy | Unhealthy |
| Cake, custard filled, with & without icing | Unhealthy | Unhealthy |
| Cake, gluten free, with & without icing | Unhealthy | Unhealthy |
| Muffin bar, fruit flavoured | Unhealthy | Unhealthy |
| Muffin, cake-style, apple, commercial, uniced | Unhealthy | Unhealthy |
| Muffin, cake-style, banana, commercial, uniced | Unhealthy | Unhealthy |
| Muffin, cake-style, berry, commercial, uniced | Unhealthy | Unhealthy |
| Muffin, cake-style, bran, with or without dried fruit, commercial, uniced | Unhealthy | Unhealthy |
| Muffin, cake-style, chocolate chip, commercial, uniced | Unhealthy | Unhealthy |
| Muffin, cake-style, chocolate, with or without chocolate chip, commercial, uniced | Unhealthy | Unhealthy |
| Muffin, cake-style, orange & poppy seed, commercial, uniced | Unhealthy | Unhealthy |
| Muffin, cake-style, plain, commercial, uniced | Unhealthy | Unhealthy |
| Muffin, cake-style, raspberry & white chocolate, commercial, uniced | Unhealthy | Unhealthy |
| Muffin, cake-style, commercial, not further defined | Unhealthy | Unhealthy |
| Pudding, plum, canned, commercial | Unhealthy | Unhealthy |
| Pudding, self-saucing, chocolate flavoured, prepared from dry mix | Unhealthy | Unhealthy |
| Pudding, self-saucing, other flavours, prepared from dry mix | Unhealthy | Unhealthy |
| Pudding, sticky date, commercial | Unhealthy | Unhealthy |
| Dumpling, sweet, soaked in syrup | Unhealthy | Unhealthy |
| Cake or cupcake, berry shortcake, undefined fat | Unhealthy | Unhealthy |
| Confectionery, rum ball (rumball) | Unhealthy | Unhealthy |
| Slice, brownie, chocolate, with nuts, commercial | Unhealthy | Unhealthy |
| Slice, caramel, commercial | Unhealthy | Unhealthy |
| Slice, chocolate, commercial, chocolate icing | Unhealthy | Unhealthy |
| Slice, coconut, with jam, commercial | Unhealthy | Unhealthy |
| Slice, fruit mince, commercial, with icing | Unhealthy | Unhealthy |
| Slice, hedgehog, commercial, chocolate icing | Unhealthy | Unhealthy |
| Slice, jelly, commercial | Unhealthy | Unhealthy |
| Slice, passionfruit | Unhealthy | Unhealthy |
| Slice, vanilla, commercial, with icing (except chocolate flavoured) | Unhealthy | Unhealthy |
| Slice, not further defined | Unhealthy | Unhealthy |
| Scone, date, commercial | Unhealthy | Unhealthy |
| Scone, plain, commercial | Unhealthy | Unhealthy |
| Scone, pumpkin, commercial | Unhealthy | Unhealthy |
| Scone, sultana, commercial | Unhealthy | Unhealthy |
| Scone, wholemeal, commercial | Unhealthy | Unhealthy |
| Scone, cheese, commercial | Unhealthy | Unhealthy |
| Scone, chocolate, with chocolate chips, commercial | Unhealthy | Unhealthy |
| Croissant, commercial, plain | Unhealthy | Unhealthy |
| Pastry, choux, commercial, baked, unfilled | Unhealthy | Unhealthy |
| Pastry, filo (fillo), commercial, raw | Unhealthy | Unhealthy |
| Pastry, filo (fillo), commercial, baked | Unhealthy | Unhealthy |
| Pastry, puff, with butter, commercial, raw | Unhealthy | Healthy |
| Pastry, puff, with butter, commercial, baked | Unhealthy | Healthy |
| Pastry, puff, vegetable oil, commercial, raw | Unhealthy | Unhealthy |
| Pastry, puff, vegetable oil, commercial, baked | Unhealthy | Unhealthy |
| Pastry, puff, commercial, raw, not further defined | Unhealthy | Unhealthy |
| Pastry, shortcrust style, commercial, raw | Unhealthy | Unhealthy |
| Pastry, shortcrust style, commercial, baked | Unhealthy | Unhealthy |
| Pastry, shortcrust style, reduced fat, commercial, raw | Unhealthy | Unhealthy |
| Pastry, shortcrust, wholemeal, commercial, raw | Unhealthy | Unhealthy |
| Pastry, shortcrust, wholemeal, commercial, baked | Unhealthy | Unhealthy |
| Pie, sweet, apple, commercial | Unhealthy | Unhealthy |
| Pie, sweet, apple, fast food outlet | Unhealthy | Unhealthy |
| Pie, sweet, apple, from frozen, baked | Unhealthy | Unhealthy |
| Pie, sweet, apple, from frozen, reduced fat, baked | Unhealthy | Unhealthy |
| Pie, sweet, apple & berry, from frozen, baked | Unhealthy | Unhealthy |
| Pie, sweet, apple & rhubarb | Unhealthy | Unhealthy |
| Pie, sweet, fruit (apple or apricot), commercial | Unhealthy | Unhealthy |
| Pie, sweet, fruit mince, commercial | Unhealthy | Unhealthy |
| Pie, sweet, pecan, commercial | Unhealthy | Unhealthy |
| Strudel, apple, commercial | Unhealthy | Unhealthy |
| Tart, jam, commercial | Unhealthy | Unhealthy |
| Cake, moon, commercial | Unhealthy | Unhealthy |
| Croissant, chocolate filled, commercial | Unhealthy | Unhealthy |
| Danish, custard & fruit filled | Unhealthy | Unhealthy |
| Danish, custard & pecan filled, commercial | Unhealthy | Unhealthy |
| Eclair or profiterole, cream filled, commercial, chocolate icing | Unhealthy | Unhealthy |
| Eclair or profiterole, custard filled, commercial, chocolate icing | Unhealthy | Unhealthy |
| Eclair or profiterole, cream & custard filled, commercial, chocolate icing | Unhealthy | Unhealthy |
| Eclair or profiterole, mock cream filled, reduced fat, commercial, chocolate icing | Unhealthy | Unhealthy |
| Pie, sweet, lemon meringue, commercial | Unhealthy | Unhealthy |
| Tart, custard, commercial | Unhealthy | Unhealthy |
| Tart, lemon or lime, commercial | Unhealthy | Unhealthy |
| Crepe or pancake, plain, commercial | Unhealthy | Unhealthy |
| Crepe or pancake, plain, dry mix | Unhealthy | Unhealthy |
| Crepe or pancake, with butter & syrup, fast food chain | Unhealthy | Unhealthy |
| Pikelet, plain, commercial | Unhealthy | Unhealthy |
| Waffle, plain, frozen, commercial | Unhealthy | Unhealthy |
| Doughnut, cream filled | Unhealthy | Unhealthy |
| Doughnut, cream filled, with chocolate icing | Unhealthy | Unhealthy |
| Doughnut, custard filled | Unhealthy | Unhealthy |
| Doughnut, custard filled, with chocolate icing | Unhealthy | Unhealthy |
| Doughnut, custard & cream filled, with chocolate icing | Unhealthy | Unhealthy |
| Doughnut, jam filled, sugar coated | Unhealthy | Unhealthy |
| Doughnut, jam filled, with chocolate icing | Unhealthy | Unhealthy |
| Doughnut, unfilled, with cinnamon & sugar dusting | Unhealthy | Unhealthy |
| Doughnut, unfilled, glazed | Unhealthy | Unhealthy |
| Doughnut, unfilled, with chocolate flavoured icing | Unhealthy | Unhealthy |
| Doughnut, unfilled, with icing (except chocolate flavoured) | Unhealthy | Unhealthy |
| **Flours, breadcrumbs and bread mixes** | | |
| Sourdough Bread Base | Unhealthy | Healthy |
| Rosemary, Thyme & Garlic Bread Mix | Unhealthy | Unhealthy |
| Brown Rice Flour | Healthy | Healthy |
| Gluten Free Quinoa Breadcrumbs | Unhealthy | Unhealthy |
| All Purpose Rice Breadcrumbs | Healthy | Healthy |
| Golden Wholemeal Bread Mix with Bio-Fort Selenium | Unhealthy | Unhealthy |
| Cranberry, Pecan & Rosemary Stuffing | Unhealthy | Unhealthy |
| Organic Unbleached Self Raising Flour | Healthy | Healthy |
| Date Loaf Mix | Unhealthy | Unhealthy |
| White Rice Crumbs | Unhealthy | Unhealthy |
| Japanese Panko Bread Crumbs | Unhealthy | Unhealthy |
| Australian Hemp Wholemeal Flour | Healthy | Healthy |
| Fibre Enriched Plain Flour | Healthy | Healthy |
| Fibre Enriched Self Raising Flour | Unhealthy | Unhealthy |
| Bread Crumbs | Unhealthy | Unhealthy |
| Gluten Free Corn Breadcrumbs | Healthy | Healthy |
| Low Fodmap Plain Flour | Unhealthy | Unhealthy |
| Super-Fine Almond Flour | Healthy | Healthy |
| Wholemeal Spelt Flour | Healthy | Healthy |
| Chickpea Flour | Healthy | Healthy |
| Flour, arrowroot | Healthy | Healthy |
| Flour, cornflour, from maize starch | Healthy | Healthy |
| Flour, cornflour, from maize & wheat starch | Healthy | Healthy |
| Flour, rice | Healthy | Healthy |
| Flour, rye, wholemeal | Healthy | Healthy |
| Flour, spelt | Healthy | Healthy |
| Flour, wheat, white, plain | Healthy | Healthy |
| Flour, wheat, white, self-raising | Healthy | Healthy |
| Flour, wheat, wholemeal, plain | Healthy | Healthy |
| Flour, wheat, wholemeal, self-raising | Healthy | Healthy |
| Gluten, from wheat (vital wheat gluten) | Healthy | Healthy |
| Flour, wheat, white, high protein or bread making flour | Healthy | Healthy |
| Flour, wheat, white, self-raising, added calcium & folate | Healthy | Healthy |
| Flour, wheat, white, plain, added vitamins B1, B2, B3, B6, E & folate & Fe, Mg & Zn (Jackaroo) | Healthy | Healthy |
| Flour, wheat, white, self-raising, added vitamins B1, B2, B3, B6, E & folate & Fe, Mg & Zn (Jackaroo) | Healthy | Healthy |
| Breadcrumbs for coating food, commercial, uncooked | Unhealthy | Unhealthy |
| Batter for coating food, commercial, uncooked | Unhealthy | Unhealthy |
| Coating, commercial, for fish & seafood, uncooked | Unhealthy | Unhealthy |
| Tempura for coating food, commercial, uncooked | Unhealthy | Unhealthy |
| Stuffing, bread-based, commercial | Unhealthy | Unhealthy |
| Flour, chick pea (besan) | Healthy | Healthy |
| Flour, soya | Healthy | Healthy |
| **Sweet biscuits** | | |
| Biscuit, sweet, Anzac style, commercial | Unhealthy | Unhealthy |
| Biscuit, sweet, Butternut Snap style, commercial | Unhealthy | Unhealthy |
| Biscuit, sweet, chocolate flavoured, commercial | Unhealthy | Unhealthy |
| Biscuit, sweet, chocolate flavoured, bite-size shell, unfilled for pods recipes, commercial | Unhealthy | Unhealthy |
| Biscuit, sweet, fortune cookie, commercial | Unhealthy | Unhealthy |
| Biscuit, sweet, ginger flavoured, commercial | Unhealthy | Unhealthy |
| Biscuit, sweet, oatmeal, commercial | Unhealthy | Unhealthy |
| Biscuit, sweet, plain, commercial | Unhealthy | Unhealthy |
| Biscuit, sweet, plain, sponge finger, commercial | Unhealthy | Unhealthy |
| Biscuit, sweet, shortbread style, commercial | Unhealthy | Unhealthy |
| Biscuit, sweet, shortbread style, with custard powder, commercial | Unhealthy | Unhealthy |
| Biscuit, sweet, sugar-glazed pastry, commercial | Unhealthy | Unhealthy |
| Biscuit, sweet, wafer style | Unhealthy | Unhealthy |
| Biscuit, sweet, wheatmeal, commercial | Unhealthy | Unhealthy |
| Biscuit, sweet, plain assorted, commercial, not further defined | Unhealthy | Unhealthy |
| Biscuit, sweet, not further defined | Unhealthy | Unhealthy |
| Biscuit mix, sweet, dry mix | Unhealthy | Unhealthy |
| Cone, wafer style, for ice cream | Unhealthy | Unhealthy |
| Cone, waffle style, for ice cream | Unhealthy | Unhealthy |
| Cone, for ice cream, not further defined | Unhealthy | Unhealthy |
| Biscuit, sweet, almond spekulatius (butter almond cookie), commercial | Unhealthy | Unhealthy |
| Biscuit, sweet, biscotti (almond bread), commercial | Unhealthy | Unhealthy |
| Biscuit, sweet, fruit-filled, commercial | Unhealthy | Unhealthy |
| Biscuit, sweet, fruit-filled, commercial, reduced fat | Unhealthy | Unhealthy |
| Biscuit, sweet, gluten free, non-chocolate flavour | Unhealthy | Unhealthy |
| Biscuit, sweet, macaroon (coconut based), commercial | Unhealthy | Unhealthy |
| Biscuit, sweet, with coconut, commercial | Unhealthy | Unhealthy |
| Biscuit, sweet, with dried fruit, commercial | Unhealthy | Unhealthy |
| Biscuit, sweet, with dried fruit & icing, commercial | Unhealthy | Unhealthy |
| Biscuit, sweet, with dried fruit & nuts, commercial | Unhealthy | Unhealthy |
| Biscuit, sweet, with nuts, commercial | Unhealthy | Unhealthy |
| Biscuit, sweet, with nuts, reduced fat, commercial | Unhealthy | Unhealthy |
| Biscuit, sweet, jam-filled, commercial | Unhealthy | Unhealthy |
| Biscuit, sweet, jam-filled, reduced fat, commercial | Unhealthy | Unhealthy |
| Biscuit, sweet, marshmallow filling, commercial | Unhealthy | Unhealthy |
| Biscuit, sweet, plain, with icing, commercial | Unhealthy | Unhealthy |
| Biscuit, sweet, sandwich, syrup wafer, commercial | Unhealthy | Unhealthy |
| Biscuit, sweet, macaron (almond based), filled | Unhealthy | Unhealthy |
| Biscuit, sweet, sandwich, butternut snap biscuit, chocolate cream filling, commercial | Unhealthy | Unhealthy |
| Biscuit, sweet, sandwich, chocolate flavour biscuit, cream filling, commercial | Unhealthy | Unhealthy |
| Biscuit, sweet, sandwich, chocolate flavoured shortbread style biscuit, chocolate icing filling, commercial | Unhealthy | Unhealthy |
| Biscuit, sweet, sandwich, cream & jam filling, commercial | Unhealthy | Unhealthy |
| Biscuit, sweet, sandwich, shortbread style biscuit, icing filling, commercial | Unhealthy | Unhealthy |
| Biscuit, sweet, sandwich, vanilla flavour biscuit, cream filling, commercial | Unhealthy | Unhealthy |
| Biscuit, sweet, sandwich, vanilla flavour biscuit, cream filling, white chocolate coated, commercial | Unhealthy | Unhealthy |
| Biscuit, sweet, sandwich, wafer layers, cream filling, chocolate flavoured, commercial | Unhealthy | Unhealthy |
| Biscuit, sweet, sandwich, wafer layers, cream filling, non-chocolate flavours, commercial | Unhealthy | Unhealthy |
| Biscuit, sweet, sandwich, wafer layers, cream filling, commercial, not further defined | Unhealthy | Unhealthy |
| Biscuit, sweet, cream assorted, commercial, not further defined | Unhealthy | Unhealthy |
| Biscuit, sweet, chocolate chip, commercial | Unhealthy | Unhealthy |
| Biscuit, sweet, chocolate chip, reduced fat, commercial | Unhealthy | Unhealthy |
| Biscuit, sweet, chocolate chip & caramel, commercial | Unhealthy | Unhealthy |
| Biscuit, sweet, chocolate chip & nut, commercial | Unhealthy | Unhealthy |
| Biscuit, sweet, gluten free, chocolate flavoured with chocolate chips | Unhealthy | Unhealthy |
| Biscuit, sweet, marshmallow filling, chocolate-coated, commercial | Unhealthy | Unhealthy |
| Biscuit, sweet, plain, chocolate-coated, commercial | Unhealthy | Unhealthy |
| Biscuit, sweet, with coconut, chocolate base, commercial | Unhealthy | Unhealthy |
| Biscuit, sweet, wheatmeal, chocolate-coated, commercial | Unhealthy | Unhealthy |
| Biscuit, sweet, biscuit base, caramel filling, chocolate-coated, commercial | Unhealthy | Unhealthy |
| Biscuit, sweet, biscuit base, mint filling, chocolate-coated, commercial | Unhealthy | Unhealthy |
| Biscuit, sweet, chocolate flavoured, bite-size shell, filled with layers of caramel & chocolate, commercial | Unhealthy | Unhealthy |
| Biscuit, sweet, sandwich, cream filling, chocolate-coated, commercial | Unhealthy | Unhealthy |
| Biscuit, sweet, sandwich, cream & caramel filling, chocolate-coated, commercial | Unhealthy | Unhealthy |
| Biscuit, sweet, sandwich, wafer layers, cream filling, chocolate flavoured, chocolate coated, commercial | Unhealthy | Unhealthy |
| Biscuit, sweet, chocolate, commercial, not further defined | Unhealthy | Unhealthy |
| Biscuit, sweet, plain, carob-coated, commercial | Unhealthy | Unhealthy |
| **Cereal and cereal products** | | |
| **Breakfast cereals** | | |
| Strawberries & Cream Instant Porridge Snack | Unhealthy | Unhealthy |
| Oats + Chia Apple Spice | Unhealthy | Unhealthy |
| Mixed Berry Oats + Chia | Unhealthy | Unhealthy |
| Banana & Mango Flavoured Oats with Chia | Unhealthy | Unhealthy |
| Banana Flavour Porridge Bowl | Unhealthy | Unhealthy |
| Original Wholegrain Corn & Soy Instant Porridge | Unhealthy | Unhealthy |
| Chocolate Flavoured Instant Porridge | Unhealthy | Unhealthy |
| Maple & Pecan Muesli Clusters | Unhealthy | Unhealthy |
| Honey & Banana Flavoured Oats | Unhealthy | Unhealthy |
| 5 Grains & Hazelnut Natural Muesli | Unhealthy | Unhealthy |
| Ancient Grains and Superfruits Natural Muesli | Unhealthy | Healthy |
| Chocolate Taste Crispy Breakfast Cereal | Unhealthy | Unhealthy |
| Choco Corn Flakes | Unhealthy | Unhealthy |
| Wheat Bran Cereal | Unhealthy | Unhealthy |
| Gluten Free Mixed Berry Porridge with Flax Flakes | Unhealthy | Unhealthy |
| Roasted Almond Muesli | Unhealthy | Healthy |
| Crunchy 3 Grain Granola with Flame Raisin & Red Apple | Unhealthy | Unhealthy |
| Corn, Oats & Wheat Original Protein Cereal | Unhealthy | Unhealthy |
| Ancient Grains | Healthy | Healthy |
| Cherry Chia Super Premium Gourmet Muesli | Unhealthy | Unhealthy |
| Mango & Coconut Quinoa Porridge | Unhealthy | Unhealthy |
| Berry Cereal | Unhealthy | Unhealthy |
| Lime & Coconut Muesli | Unhealthy | Unhealthy |
| Cacao & Wild Berry Granola | Unhealthy | Unhealthy |
| Berries & Toasted Coconut Cereals | Unhealthy | Unhealthy |
| Nicely Nutty Muesli | Unhealthy | Unhealthy |
| Crunchy Granola Blueberry, Coconut & Quinoa | Unhealthy | Unhealthy |
| Cacao Coconut Crunch Paleo Granola | Unhealthy | Unhealthy |
| Golden Maple Gourmet Porridge Sachets | Unhealthy | Unhealthy |
| Almond, Coconut & Chia Gourmet Porridge Sachets | Unhealthy | Healthy |
| Super Berry & Coconut Gourmet Porridge Sachets | Unhealthy | Unhealthy |
| Apricot, Sultanas, Apples + Coconut Muesli Flakes | Unhealthy | Unhealthy |
| Crunchy Granola with Cashew & Almond | Unhealthy | Unhealthy |
| Quick 2 Minutes Smooth & Creamy Oats | Healthy | Healthy |
| Untoasted Apricot, Peach & Coconut Muesli | Unhealthy | Unhealthy |
| Black Rice, Honey & Cinnamon Pudding Brekky Rice | Unhealthy | Healthy |
| Black Rice & Coconut Pudding Brekky Rice | Unhealthy | Unhealthy |
| Cacao & Maca Breakfast Bowl with Superfoods | Unhealthy | Unhealthy |
| Wholegrain Oats with Chia | Healthy | Healthy |
| Porridge Oats | Healthy | Healthy |
| Protein Oats | Healthy | Healthy |
| Roasted Almond & Cinnamon Oats | Unhealthy | Unhealthy |
| Creamy Vanilla Flavour Oats | Unhealthy | Unhealthy |
| Honey Roasted Nut Gourmet Porridge Sachets | Unhealthy | Healthy |
| Very Berry Toasted Muesli | Unhealthy | Unhealthy |
| Original Gluten Free Porridge | Healthy | Healthy |
| Gluten Free Cacao Porridge | Healthy | Healthy |
| Oats | Healthy | Healthy |
| Puffed Sorghum, Nut & Cinnamon Muesli | Unhealthy | Unhealthy |
| Organic Creamy Style Instant Oats | Healthy | Healthy |
| Crunchy Honey Cereal | Unhealthy | Unhealthy |
| Natural Protein Instant Oats | Healthy | Healthy |
| Berry Bliss Granola | Unhealthy | Unhealthy |
| Cranberry & Fig Baked Muesli | Unhealthy | Healthy |
| Gluten Free Fruity Premium Muesli | Unhealthy | Unhealthy |
| Honey Flavoured Quick Oats Sachets | Unhealthy | Unhealthy |
| Honey & Vanilla Porridge Sachets | Unhealthy | Unhealthy |
| Berry-Licious Porridge Sachets | Unhealthy | Healthy |
| Corn Flakes | Unhealthy | Unhealthy |
| Dark Chocolate & Toasted Coconut Granola | Unhealthy | Unhealthy |
| Balanced Right Cereal | Unhealthy | Unhealthy |
| Original Cereal | Unhealthy | Unhealthy |
| Creamy Style Australian Oats Protein Porridge | Unhealthy | Unhealthy |
| Multigrain Porridge | Healthy | Healthy |
| Chocolate Granola | Unhealthy | Unhealthy |
| Honey Granola | Unhealthy | Unhealthy |
| Apple & Brown Sugar Porridge Sachets | Unhealthy | Unhealthy |
| Oats, Rye & Spelt with Almonds and a Hint of Cinnamon | Healthy | Healthy |
| Full o' Fruit Toasted Muesli with a Blend of Tropical Fruits | Unhealthy | Unhealthy |
| Quick Oats | Healthy | Healthy |
| Macadamia with Oat Clusters Cereal | Unhealthy | Unhealthy |
| Apple & Sultana Muesli | Unhealthy | Unhealthy |
| Cranberry, Almond & Cinnamon Muesli | Unhealthy | Unhealthy |
| Choc Boulders | Unhealthy | Unhealthy |
| Almond & Manuka Honey Toasted Muesli | Unhealthy | Unhealthy |
| Three Grain Raspberry Porridge | Unhealthy | Unhealthy |
| Chai Spiced Apricot & Buckwheat Porridge | Unhealthy | Unhealthy |
| Chocolate Porridge | Unhealthy | Unhealthy |
| Vanilla Dream Flavoured Instant Probiotic Protein Porridge | Unhealthy | Unhealthy |
| Puffed Rice Grains with Cocoa | Unhealthy | Unhealthy |
| Oat bran, unprocessed, uncooked | Healthy | Healthy |
| Oats, rolled, uncooked | Healthy | Healthy |
| Oats, rolled, uncooked, added fibre & Ca | Healthy | Healthy |
| Breakfast cereal, flakes of corn, unfortified | Unhealthy | Healthy |
| Breakfast cereal, flakes of corn, no added sugar or salt, unfortified | Unhealthy | Healthy |
| Breakfast cereal, puffed or popped corn, no added sugar or salt, unfortified | Unhealthy | Healthy |
| Breakfast cereal, crispy corn pillows, honey, added vitamins B1, B2, B3 & folate | Unhealthy | Unhealthy |
| Breakfast cereal, flakes of corn, added vitamins B1, B2, B3 & folate | Unhealthy | Unhealthy |
| Breakfast cereal, flakes of corn, added vitamins B1, B2, B3, C & folate, Fe & Zn | Unhealthy | Unhealthy |
| Breakfast cereal, flakes of corn, added vitamins B1, B2, B3, B6, E & folate & Fe | Unhealthy | Unhealthy |
| Breakfast cereal, flakes of corn, added vitamins B1, B2, B3 & folate & Fe | Unhealthy | Unhealthy |
| Breakfast cereal, flakes of corn, with psyllium, added vitamins B1, B2, B3 & folate & Fe | Unhealthy | Unhealthy |
| Breakfast cereal, flakes of corn, not further defined | Unhealthy | Unhealthy |
| Breakfast cereal, flakes of corn, added nuts, added vitamins B1, B2, B3, C & folate, Fe & Zn | Unhealthy | Unhealthy |
| Breakfast cereal, flakes of corn, added nuts, added vitamins B1, B2, B3, B6, folate & E | Unhealthy | Unhealthy |
| Breakfast cereal, flakes of corn, added nuts, added vitamins B1, B2, B3 & folate & Fe | Unhealthy | Unhealthy |
| Breakfast cereal, frosted flakes of corn, added vitamins B1, B2, B3, C, E & folate, Fe & Zn | Unhealthy | Unhealthy |
| Breakfast cereal, corn based, not further defined | Unhealthy | Unhealthy |
| Breakfast cereal, puffed or popped rice, no added sugar or salt, unfortified | Unhealthy | Healthy |
| Breakfast cereal, puffed or popped rice, cocoa coating, no added sugar or salt, unfortified | Unhealthy | Unhealthy |
| Breakfast cereal, puffed or popped rice, cocoa coating, unfortified | Unhealthy | Unhealthy |
| Breakfast cereal, flakes of rice, added vitamins B1, B2, B3 & folate & Fe | Unhealthy | Unhealthy |
| Breakfast cereal, puffed or popped rice, added vitamins B1, B2 & B3 & Fe | Unhealthy | Unhealthy |
| Breakfast cereal, puffed or popped rice, added vitamins B1, B3 & folate & Fe | Unhealthy | Unhealthy |
| Breakfast cereal, puffed or popped rice, added vitamins B1, B2, B3 & folate & Fe | Unhealthy | Unhealthy |
| Breakfast cereal, puffed or popped rice, added vitamins B1, B2, B3, C & folate, Fe & Zn | Unhealthy | Unhealthy |
| Breakfast cereal, puffed or popped rice, not further defined | Unhealthy | Unhealthy |
| Breakfast cereal, puffed or popped rice, cocoa coating, added vitamins B1, B2, B3 & folate & Fe | Unhealthy | Unhealthy |
| Breakfast cereal, puffed or popped rice, cocoa coating, added vitamins B1, B2, B3, C & folate, Ca, Fe & Zn | Unhealthy | Unhealthy |
| Breakfast cereal, wheat bran, flakes, unfortified | Unhealthy | Unhealthy |
| Breakfast cereal, whole wheat, biscuit, no added sugar or salt, unfortified | Unhealthy | Healthy |
| Breakfast cereal, whole wheat, biscuit, no added sugar, unfortified | Unhealthy | Healthy |
| Breakfast cereal, whole wheat, biscuit, added sugar & salt, unfortified | Unhealthy | Healthy |
| Breakfast cereal, whole wheat, flakes, no added sugar, unfortified | Unhealthy | Healthy |
| Breakfast cereal, whole wheat, puffed, no added sugar or salt, unfortified | Unhealthy | Healthy |
| Breakfast cereal, whole wheat, puffed, honey, unfortified | Unhealthy | Unhealthy |
| Breakfast cereal, wheat bran, flakes, added vitamins B1, B2 B3, B6, & folate, Fe & Zn | Unhealthy | Healthy |
| Breakfast cereal, wheat bran, pellets, added vitamins B1, B2 & folate | Unhealthy | Unhealthy |
| Breakfast cereal, wheat bran, pellets, added vitamins B1, B2, B3 & folate & Fe | Unhealthy | Unhealthy |
| Breakfast cereal, wheat bran, pellets, added vitamins E & folate, Ca & Zn | Unhealthy | Unhealthy |
| Breakfast cereal, wheat bran, pellets, added vitamins A, B1, B2, B3, E & folate, Ca & Fe | Unhealthy | Unhealthy |
| Breakfast cereal, wheat bran, pellets, not further defined | Unhealthy | Unhealthy |
| Breakfast cereal, whole wheat, biscuit, added vitamins B1, B2 & B3 | Unhealthy | Healthy |
| Breakfast cereal, whole wheat, biscuit, added vitamins B1, B2 & B3, Ca, Fe & Zn | Unhealthy | Healthy |
| Breakfast cereal, whole wheat, biscuit, added vitamins B1, B2, B3 & folate, Fe & Zn | Unhealthy | Healthy |
| Breakfast cereal, whole wheat, biscuit, bran, added vitamins B1, B2, B3 & folate & Fe | Unhealthy | Healthy |
| Breakfast cereal, whole wheat, biscuit, not further defined | Unhealthy | Healthy |
| Breakfast cereal, whole wheat, flakes, added vitamins B1, B2, B3 & folate | Unhealthy | Healthy |
| Breakfast cereal, whole wheat, flakes, added vitamins B1, B2, B3, C & folate & Fe | Unhealthy | Unhealthy |
| Breakfast cereal, whole wheat, small biscuit, added vitamins B1, B2, B3 & folate | Unhealthy | Healthy |
| Breakfast cereal, wheat based, commercial, not further defined | Unhealthy | Healthy |
| Breakfast cereal, whole wheat, puffed, honey, added vitamins B1, B2, B3, B6, E & folate | Unhealthy | Unhealthy |
| Breakfast cereal, whole wheat, small biscuit, with honey, added vitamins B1, B2, B3 & folate, Fe & Zn | Unhealthy | Unhealthy |
| Breakfast cereal, whole wheat, flakes, added dried fruit &/or nuts, no added sugar, unfortified | Unhealthy | Unhealthy |
| Breakfast cereal, wheat bran, flakes, sultanas, added vitamins B1, B2, B3, B6 & folate, Fe & Zn | Unhealthy | Healthy |
| Breakfast cereal, whole wheat, small biscuit, with apricot, added vitamins B1, B2, B3 & folate, Ca & Fe | Unhealthy | Unhealthy |
| Breakfast cereal, whole wheat, small biscuit, with berries, added vitamins B1, B2, B3 & folate, Ca & Fe | Unhealthy | Unhealthy |
| Breakfast cereal, whole wheat, small biscuit, with blackcurrant paste, added vitamins B1, B2, B3 & folate | Unhealthy | Unhealthy |
| Breakfast cereal, whole wheat, small biscuit, with fruit paste, added vitamins B1, B2, B3 & C & Fe | Unhealthy | Unhealthy |
| Breakfast cereal, whole wheat, flakes, dried fruit, added vitamins B1, B2, B3, C & folate | Unhealthy | Unhealthy |
| Breakfast cereal, whole wheat, flakes, dried fruit & nuts, added fibre, vitamins B1, B2, B3 & folate, Ca & Fe | Unhealthy | Unhealthy |
| Breakfast cereal, wheat bran, flakes, honey & almond, added vitamins B1, B2 B3, B6 & folate, Fe & Zn | Unhealthy | Unhealthy |
| Breakfast cereal, wheat bran, flakes, sultanas, added vitamins B1, B2, B3 & folate & Fe | Unhealthy | Unhealthy |
| Breakfast cereal, wheat bran, small biscuit, with sultana paste, added vitamins B1, B2, B3, B6, E & folate, Fe & Zn | Unhealthy | Unhealthy |
| Breakfast cereal, mixed grain (barley & soy), flakes, honey, unfortified | Unhealthy | Unhealthy |
| Breakfast cereal, mixed grain (wheat & corn), flakes or extruded, no added salt, unfortified | Unhealthy | Unhealthy |
| Breakfast cereal, mixed grain (wheat, oat & corn), flakes or extruded, no added salt, unfortified | Unhealthy | Unhealthy |
| Breakfast cereal, mixed grain (wheat, rice & oat), flakes, honey, unfortified | Unhealthy | Unhealthy |
| Breakfast cereal, oat & whole wheat, biscuit, added salt, unfortified | Unhealthy | Healthy |
| Breakfast cereal, mixed grain (rice & wheat), flakes, added vitamins B1, B2, B3, B6 & folate, Ca, Fe & Zn | Unhealthy | Unhealthy |
| Breakfast cereal, mixed grain (wheat, oat, rice & corn), clusters, added vitamins B1, B2, B3, C & folate, Fe & Zn | Unhealthy | Unhealthy |
| Breakfast cereal, mixed grain (wheat, corn, rice & oat), extruded, added vitamins B1, B2, B3, B6, C, E & folate, Ca, Fe & Zn | Unhealthy | Unhealthy |
| Breakfast cereal, mixed grain (wheat, corn, rice & oat), flakes, added vitamins B1, B2, B3, B6, E & folate, Ca & Fe | Unhealthy | Unhealthy |
| Breakfast cereal, mixed grain (wheat, rice & oat), flakes, honey, added vitamins B1, B2, B3 & folate & Fe | Unhealthy | Unhealthy |
| Breakfast cereal, whole wheat, biscuit, multigrain, added vitamins B1, B2, B3 & E & Fe | Unhealthy | Healthy |
| Breakfast cereal, mixed grain (wheat & corn), extruded, chocolate malt coating, added vitamins B1, B2, B3, B6, C, E & folate, Ca, Fe & Zn | Unhealthy | Unhealthy |
| Breakfast cereal, mixed grain (wheat, oat & corn), extruded, added vitamins B1, B2, B3, B6 & C, Ca & Fe | Unhealthy | Unhealthy |
| Breakfast cereal, mixed grain (wheat, oat & corn), extruded, added vitamins B1, B2, B3, B6, C & folate, Fe & Zn | Unhealthy | Unhealthy |
| Breakfast cereal, mixed grain (wheat, oat & corn), extruded, added vitamins B1, B2, B3, B6, C & folate, Ca, Fe & Zn | Unhealthy | Unhealthy |
| Breakfast cereal, mixed grain (wheat, oat & corn), extruded, fruit flavours, added vitamins B1, B2, B3, C & folate, Fe & Zn | Unhealthy | Unhealthy |
| Breakfast cereal, mixed grain (wheat, corn, rice & oat), extruded, honey, added vitamins B1, B2, B3, B6, C, E & folate, Ca, Fe & Zn | Unhealthy | Unhealthy |
| Breakfast cereal, mixed grain (wheat, oat & corn), extruded, cocoa coating, added B1, B2, B3, B6, C & folate, Ca, Fe & Zn | Unhealthy | Unhealthy |
| Breakfast cereal, mixed grain (wheat, corn & rice), extruded, cocoa coating, added vitamins B1, B2, B3, B6, C, E & folate, Ca, Fe, Mg & Zn | Unhealthy | Unhealthy |
| Breakfast cereal, mixed grain (wheat, corn, rice & oat), crispy pillows, cocoa coating, added vitamins B1, B2, B3 & folate | Unhealthy | Unhealthy |
| Breakfast cereal, mixed grain (rice & wheat), flakes, almond & honey, vitamins B1, B2, B3, B6 & folate, Ca, Fe & Zn | Unhealthy | Unhealthy |
| Breakfast cereal, mixed grain (barley & oat), flakes, honey, added fruit, nuts & seeds, unfortified | Unhealthy | Unhealthy |
| Breakfast cereal, mixed grain (wheat & oat), flakes, dried fruit, no added salt, unfortified | Unhealthy | Unhealthy |
| Breakfast cereal, mixed grain (wheat, rice & oat), flakes, dried fruit, no added salt, unfortified | Unhealthy | Unhealthy |
| Breakfast cereal, mixed grain (wheat, rice & oat), flakes, cashew, almond, hazelnut & coconut, unfortified | Unhealthy | Unhealthy |
| Breakfast cereal, mixed grain (wheat, rice & oat), flakes & clusters, apple & raisins, unfortified | Unhealthy | Unhealthy |
| Breakfast cereal, mixed grain (wheat, corn, rice & oat), flakes, added fruit &/or nuts, no added salt, unfortified | Unhealthy | Unhealthy |
| Muesli, commercial, gluten free | Unhealthy | Healthy |
| Muesli, commercial, toasted, added dried fruit, unfortified | Unhealthy | Healthy |
| Muesli, commercial, toasted, added dried fruit & nuts, unfortified | Unhealthy | Healthy |
| Muesli, commercial, toasted, added nuts, unfortified | Unhealthy | Healthy |
| Muesli, commercial, untoasted or natural style, added dried fruit, unfortified | Healthy | Healthy |
| Muesli, commercial, untoasted or natural style, added dried fruit, low fat, unfortified | Unhealthy | Unhealthy |
| Muesli, commercial, untoasted or natural style, added dried fruit & nuts, unfortified | Healthy | Healthy |
| Muesli, commercial, untoasted or natural style, added nuts & seeds | Healthy | Healthy |
| Breakfast cereal, mixed grain (rice & wheat), flakes, berries, added vitamins B1, B2, B3, B6 & folate, Ca, Fe & Zn | Unhealthy | Unhealthy |
| Breakfast cereal, mixed grain (rice & wheat), flakes, fruit & nut, added vitamins B1, B2, B3, B6, E & folate, Ca, Fe & Zn | Unhealthy | Unhealthy |
| Breakfast cereal, mixed grain (rice & wheat), flakes, nuts, added vitamins B1, B2 & B3, Ca & Fe | Unhealthy | Unhealthy |
| Breakfast cereal, mixed grain (wheat & corn), flakes, berries & sultanas, added vitamins A, B1, B2, B3, B6, E & folate, Ca, Fe & Zn | Unhealthy | Unhealthy |
| Breakfast cereal, mixed grain (wheat & corn), flakes, sultana, apple & currant, added vitamins B1, B2, B3, B6, E & folate, Ca, Fe & Zn | Unhealthy | Unhealthy |
| Breakfast cereal, mixed grain (wheat & oat), clusters, almond, added vitamins B1, B2, B3, B6, C, E & folate, Ca, Fe & Zn | Unhealthy | Unhealthy |
| Breakfast cereal, mixed grain (wheat & oat), flakes, apple, sultana & cranberry, added vitamins B1, B2, B3, B6, C, E & folate | Unhealthy | Unhealthy |
| Breakfast cereal, mixed grain (wheat & oat), flakes, apricot & sultana, added vitamins B1, B2, B3 & folate & Fe | Unhealthy | Unhealthy |
| Breakfast cereal, mixed grain (wheat & oat), flakes, berries, added vitamins B1, B2, B3 & folate & Fe | Unhealthy | Unhealthy |
| Breakfast cereal, mixed grain (wheat, oat & corn), clusters, honey, nuts, added vitamins B1, B2, B3, E, C & folate, Fe & Zn | Unhealthy | Unhealthy |
| Breakfast cereal, mixed grain (wheat, oat & corn), flakes, fruit, added vitamins B1, B2, B3, B6, E & folate, Ca, Fe & Zn | Unhealthy | Unhealthy |
| Breakfast cereal, mixed grain (wheat, oat & corn), flakes, fruit & nuts, added vitamins B1, B2, B3, C & folate & Fe | Unhealthy | Unhealthy |
| Breakfast cereal, mixed grain (wheat, oat & corn), flakes, fruit & nuts, added vitamins B1, B2, B3, B6, E & folate, Ca, Fe & Zn | Unhealthy | Unhealthy |
| Breakfast cereal, mixed grain (wheat, rice & oat), flakes & clusters, apple, added vitamins B1, B2, B3, B6, E & folate, Fe & Zn | Unhealthy | Unhealthy |
| Breakfast cereal, mixed grain (wheat, oat & corn), flakes & clusters, pomegranate & berries, added vitamins A, B1, B2, B3, B6, E & folate, Ca, Fe & Zn | Unhealthy | Unhealthy |
| Breakfast cereal, mixed grain (wheat, rice & oat), flakes & clusters, sultanas, added vitamins B1, B2, B3, B6, C & folate, Fe & Zn | Unhealthy | Unhealthy |
| Breakfast cereal, mixed grain (wheat, rice & oat), flakes, dried fruit, added vitamins B1, B2, B3 & E & Fe | Unhealthy | Unhealthy |
| Breakfast cereal, mixed grain (wheat, corn, rice & oat), clusters, apple & almonds, added vitamins B1, B2, B3 & folate & Fe | Unhealthy | Unhealthy |
| Breakfast cereal, mixed grain (wheat, corn, rice & oat), clusters, sultana & berries, added vitamins B1, B2, B3, E & folate, Ca & Fe | Unhealthy | Unhealthy |
| Breakfast cereal, mixed grain (wheat, corn, rice & oat), flakes & clusters, honey & macadamias, added vitamins B1, B2, B3, E & folate, Ca & Fe | Unhealthy | Unhealthy |
| Breakfast cereal, mixed grain (wheat, corn, rice & oat), flakes, apricot & coconut, added vitamins B1, B2, B3, E & folate, Ca & Fe | Unhealthy | Unhealthy |
| Breakfast cereal, mixed grain (wheat, corn, rice & oat), flakes, berries, added vitamins B1, B2, B3, E & folate, Ca & Fe | Unhealthy | Unhealthy |
| Breakfast cereal, mixed grain (wheat, corn, rice & oat), flakes, fruit, added vitamins B1, B2, B3 & folate & Fe | Unhealthy | Unhealthy |
| Breakfast cereal, mixed grain (wheat, corn, rice & oat), flakes, fruit, added vitamins B1, B2, B3, & folate, Ca & Fe | Unhealthy | Unhealthy |
| Breakfast cereal, mixed grain (wheat, corn, rice & oat), flakes, fruit & nuts, added vitamins B1, B2, B3, C & folate & Fe | Unhealthy | Unhealthy |
| Breakfast cereal, mixed grain, commercial, not further defined | Unhealthy | Unhealthy |
| Muesli, commercial, toasted, added dried fruit & nuts, added vitamins B2, B3, E & folate & Ca | Unhealthy | Unhealthy |
| Breakfast cereal, barley, flakes, honey, added sultanas, unfortified | Unhealthy | Unhealthy |
| Breakfast cereal, cocoa coating, commercial, not further defined | Unhealthy | Unhealthy |
| Breakfast cereal, not further defined | Unhealthy | Healthy |
| Oats, rolled, mixed with sugar or honey & other flavours, uncooked | Unhealthy | Unhealthy |
| Oats, rolled, mixed with sugar, flavours & dried fruit, uncooked | Unhealthy | Unhealthy |
| **Cereal bars** | | |
| Apricot & Almond with Toasted Muesli Bites | Unhealthy | Unhealthy |
| Vanilla Crunch Rice Bars | Unhealthy | Unhealthy |
| Cocoa Crunch Bars | Unhealthy | Unhealthy |
| Nut Free Ancient Grains Super Bar | Unhealthy | Unhealthy |
| Yoghurt, Peach & Mango Oat Slice | Unhealthy | Unhealthy |
| Mixed Berry Yogurt Flavour Snack Bars | Unhealthy | Unhealthy |
| Golden Oats Carob Dipped Bars | Unhealthy | Unhealthy |
| Raspberry & White Choc Chip Flavour Muesli Bars | Unhealthy | Unhealthy |
| Choc Honeycomb Muesli Bars | Unhealthy | Unhealthy |
| Cranberry & Yogurt Bars with Almonds & Pepitas | Unhealthy | Unhealthy |
| Ginger Dark Choc Coated Bars | Unhealthy | Unhealthy |
| Cranberry & Almond Muesli Bars | Unhealthy | Unhealthy |
| White Choc Chip Muesli Bars | Unhealthy | Unhealthy |
| Vanilla Blueberry Bars | Unhealthy | Unhealthy |
| Strawberry & Blackcurrant Flavour Muesli Bar | Unhealthy | Unhealthy |
| Strawberry Yogurt Mini Muesli Bars | Unhealthy | Unhealthy |
| Raspberry Fruity Tops with Yogurt Drizzle Bars | Unhealthy | Unhealthy |
| Choc Chip Chia Crunchy Granola & Seed Bars | Unhealthy | Unhealthy |
| Yogurt & Strawberry Muesli Bars | Unhealthy | Unhealthy |
| Nut Deluxe Bars | Unhealthy | Unhealthy |
| Peanut Butter Baked Oaty Slices | Unhealthy | Unhealthy |
| French Vanilla with Sour Cherry Bar | Unhealthy | Unhealthy |
| Raspberry Flavoured Muesli Bars | Unhealthy | Unhealthy |
| Peanut Butter & Honey Energy Bar | Unhealthy | Unhealthy |
| Nutty Choc Chip Energy Bar | Unhealthy | Unhealthy |
| Cranberry & Almond Bars | Unhealthy | Unhealthy |
| Chewy Oh My Choc Wholegrain Bar | Unhealthy | Unhealthy |
| Banana & Nut Breakfast Bar | Unhealthy | Unhealthy |
| Lemon Coconut Baked Oat Slice | Unhealthy | Unhealthy |
| Chocolate Chip Energy Bar | Unhealthy | Unhealthy |
| Fruit Free Bars with Coffee & Cacao | Unhealthy | Unhealthy |
| Vanilla Shake Crunch Bar | Unhealthy | Unhealthy |
| Raspberry Flavoured Yoghurt Muesli Bars | Unhealthy | Unhealthy |
| Choc Rainbow Rice Crispy Bars | Unhealthy | Unhealthy |
| Almond Berry with Creamy Yoghurt All Day Brekky Bar | Unhealthy | Unhealthy |
| Apple & Cinnamon Flavoured Fruity Filled Bars | Unhealthy | Unhealthy |
| Chocolate Puffed Rice Bars | Unhealthy | Unhealthy |
| Golden Honey Linseed Crunchy Granola Bars | Unhealthy | Unhealthy |
| Yoghurty Strawberry Flavoured Muesli Slice | Unhealthy | Unhealthy |
| Triple Berry Muesli Bars with Oats, Seeds, Almonds, Currants, Berries and Cinnamon | Unhealthy | Unhealthy |
| Blueberry Crisp Energy Bar | Unhealthy | Unhealthy |
| Almond Apricot Milk Choc High Energy Bar | Unhealthy | Unhealthy |
| Banana Split Snack Bars | Unhealthy | Unhealthy |
| Choc Peanut Butter Snack Bars | Unhealthy | Unhealthy |
| Organic Choc Almond Chia Chewy Muesli Bars | Unhealthy | Unhealthy |
| Blueberry & Ancient Grains Bars | Unhealthy | Unhealthy |
| Honey Hazelnut Prebiotic Bars | Unhealthy | Unhealthy |
| Bar, muesli or snack, with caramel & sesame seeds | Unhealthy | Unhealthy |
| Bar, muesli or snack, made from breakfast cereal | Unhealthy | Unhealthy |
| Bar, muesli or snack, made from breakfast cereal with milk solids | Unhealthy | Unhealthy |
| Bar, muesli or snack, made from breakfast cereal, with chocolate coating, added vitamins B1, B2, B3, B6 & folate, Ca, Fe & Zn | Unhealthy | Unhealthy |
| Bar, muesli or snack, made from puffed rice, added vitamins B1, B2, B3, C & folate, Fe, & Zn | Unhealthy | Unhealthy |
| Bar, muesli or snack, made from puffed rice, with chocolate flavour, chips or coating | Unhealthy | Unhealthy |
| Bar, snack style, chocolate fortified cereal, milk solids | Unhealthy | Unhealthy |
| Bar, muesli or snack, plain or with 10% dried fruit | Unhealthy | Unhealthy |
| Bar, muesli or snack, plain or with 10% dried fruit, added vitamins B1, B2, B3, folate & Fe | Unhealthy | Unhealthy |
| Bar, muesli or snack, plain or with 10% dried fruit, high fibre, added vitamins B1, B2, B3, B6, E, & folate, Fe & Zn | Unhealthy | Unhealthy |
| Bar, muesli or snack, with 10% dried fruit & 5% nuts | Unhealthy | Unhealthy |
| Bar, muesli or snack, with 10% dried fruit & 10% nuts | Unhealthy | Unhealthy |
| Bar, muesli or snack, with 10% dried fruit & 45% nuts, chocolate-coated | Unhealthy | Unhealthy |
| Bar, muesli or snack, with 10% dried fruit & 60% nuts | Unhealthy | Unhealthy |
| Bar, muesli or snack, with 10% dried fruit & 60% nuts, yoghurt-coated | Unhealthy | Unhealthy |
| Bar, muesli or snack, with 15% dried fruit & 25% nuts, added vitamins B1, B2, B3, C & folate, Fe, & Zn | Unhealthy | Unhealthy |
| Bar, muesli or snack, with 20% dried fruit & 5% nuts | Unhealthy | Unhealthy |
| Bar, muesli or snack, with 20% dried fruit & 20% nuts, chocolate base | Unhealthy | Unhealthy |
| Bar, muesli or snack, with 30% dried fruit & 30% nuts | Unhealthy | Unhealthy |
| Bar, muesli or snack, with 10% nuts | Unhealthy | Unhealthy |
| Bar, muesli or snack, with 10% nuts, added flaxseeds | Unhealthy | Unhealthy |
| Bar, muesli or snack, with 70% nuts | Unhealthy | Unhealthy |
| Bar, muesli or snack, with 70% nuts, added vitamins B1, B2, B3, C & folate, Fe, & Zn | Unhealthy | Unhealthy |
| Bar, muesli or snack, gluten free, with 20% dried fruit & 20% seeds | Unhealthy | Unhealthy |
| Bar, muesli or snack, made from breakfast cereal with dried fruit | Unhealthy | Unhealthy |
| Bar, muesli or snack, made from breakfast cereal with nuts | Unhealthy | Unhealthy |
| Bar, muesli or snack, made from breakfast cereal, with chocolate & nuts, added vitamins B1, B2, B3, C & folate, Fe & Zn | Unhealthy | Unhealthy |
| Bar, muesli or snack, not further defined | Unhealthy | Unhealthy |
| Bar, nutrition, energy or snack | Unhealthy | Unhealthy |
| Bar, muesli or snack, plain or with 10% dried fruit, chocolate-coated | Unhealthy | Unhealthy |
| Bar, muesli or snack, plain or with 10% dried fruit, yoghurt-coated | Unhealthy | Unhealthy |
| Bar, muesli or snack, plain or with 10% dried fruit, yoghurt-coated, added vitamins B1, B2, B3, folate & Fe | Unhealthy | Unhealthy |
| Bar, muesli or snack, with 15% dried fruit & 15% nuts, yoghurt-coated | Unhealthy | Unhealthy |
| Bar, muesli or snack, with 30% dried fruit, yoghurt-coated, added vitamins B1, B2, B3, folate & Fe | Unhealthy | Unhealthy |
| Bar, muesli or snack, with 10% nuts, chocolate-coated | Unhealthy | Unhealthy |
| Bar, muesli or snack, with chocolate chips or coating | Unhealthy | Unhealthy |
| Bar, muesli or snack, made from puffed rice, with chocolate flavour, chips or coating, added vitamins B1, B2, B3, C & folate, Fe, & Zn | Unhealthy | Unhealthy |
| Bar, muesli or snack, made from puffed rice, with yoghurt flavour coating, added vitamins B1, B2, B3, C & folate, Fe, & Zn | Unhealthy | Unhealthy |
| Bar, muesli or snack, fruit filled, baked | Unhealthy | Unhealthy |
| Bar, muesli or snack, fruit filled, high fibre, baked | Unhealthy | Unhealthy |
| **Couscous** | | |
| Moroccan Flavoured Cous Cous | Unhealthy | Unhealthy |
| Mediterranean Flavoured Cous Cous | Unhealthy | Unhealthy |
| Mediterranean Couscous | Healthy | Healthy |
| Roasted Vegetable Flavoured Cous Cous | Unhealthy | Unhealthy |
| Israeli Pearl Couscous | Unhealthy | Healthy |
| French Organic Whole Wheat Couscous | Healthy | Healthy |
| Wholemeal Couscous | Healthy | Healthy |
| Lebanese Couscous | Healthy | Healthy |
| Mexican Cous Cous, Rice, Bulgur Wheat & Lentil Snack Pot | Unhealthy | Unhealthy |
| Moroccan Cous Cous, Rice & Bulgur Wheat Snack Pot | Unhealthy | Unhealthy |
| Cauliflower Cous Cous with Hemp, Kale & Pepitas | Unhealthy | Unhealthy |
| Couscous, uncooked | Healthy | Healthy |
| **Noodles** | | |
| Spicy Kung Pao Udon Noodle Bowl | Unhealthy | Unhealthy |
| Pad Thai Noodles | Unhealthy | Unhealthy |
| Instant Brown Rice Ramen | Unhealthy | Healthy |
| Instant Buckwheat Ramen | Unhealthy | Healthy |
| Oriental Style Instant Noodles | Unhealthy | Unhealthy |
| Ramen Noodles | Unhealthy | Unhealthy |
| Singapore Wok Ready Noodles | Unhealthy | Unhealthy |
| Thin Rice Wok Ready Noodles | Healthy | Healthy |
| Udon Wok Ready Noodles | Unhealthy | Unhealthy |
| Soba Wok Ready Noodles | Unhealthy | Unhealthy |
| Curry Laksa Flavour Instant Pulled Noodles with Dry Tofu and Vegetable | Unhealthy | Unhealthy |
| Spicy Chicken Shiitake Flavour Instant Pulled Noodles with Shiitake Mushrooms and Vegetables | Unhealthy | Unhealthy |
| Mi Goreng Instant Cup Noodles | Unhealthy | Unhealthy |
| Sweet Potato Noodles | Healthy | Healthy |
| Organic Broad Noodles | Healthy | Healthy |
| Gluten Free Rice Hokkien Fresh Noodles | Healthy | Healthy |
| Hokkien Thin Fresh Noodles | Unhealthy | Unhealthy |
| Fresh Fried Chow Mein Noodles | Unhealthy | Unhealthy |
| Wok Ready Flat Noodles | Unhealthy | Healthy |
| Instant Noodles with Real Australian Roast Beef | Unhealthy | Unhealthy |
| Steamed Singapore Noodles | Unhealthy | Unhealthy |
| Chow Mein Fresh Noodles | Unhealthy | Healthy |
| Instant Noodle Vegetable Soup | Unhealthy | Unhealthy |
| Gomtang Beef Flavour with Vegetable Noodles | Unhealthy | Unhealthy |
| Chicken Flavour Oriental Style Instant Noodles | Unhealthy | Unhealthy |
| Chicken Flavour Brown Rice Noodle Cup | Unhealthy | Unhealthy |
| Egg Noodles | Healthy | Healthy |
| Mi Goreng BBQ Chicken Flavour Instant Cup Noodles | Unhealthy | Unhealthy |
| Brown Rice Vermicelli | Healthy | Healthy |
| Noodles with BBQ Teriyaki Flavour | Unhealthy | Unhealthy |
| Hokkien Noodles with Sweet Chilli Chicken Flavoured Sauce | Unhealthy | Unhealthy |
| Chow Mein Noodles | Unhealthy | Unhealthy |
| Mi Goreng Flavoured Instant Noodles | Unhealthy | Unhealthy |
| Original Fried Noodles | Unhealthy | Unhealthy |
| Abalone Noodle with Black Truffle Oil | Unhealthy | Unhealthy |
| XO Sauce Abalone Noodles | Unhealthy | Unhealthy |
| Organic Turmeric Rice Noodle | Healthy | Healthy |
| Organic Black Rice Noodle | Healthy | Healthy |
| White Organic Rice Noodle | Healthy | Healthy |
| Chicken & Sweetcorn Noodle Snack | Unhealthy | Unhealthy |
| Mi Goreng Satay Flavour Stir Fry Instant Noodles Multipack | Unhealthy | Unhealthy |
| Lower Carb Konjac Noodles | Healthy | Healthy |
| Instant Tom Yum Flavoured Noodles Bowl | Unhealthy | Unhealthy |
| Tai Chi Chicken Flavoured Noodles | Unhealthy | Unhealthy |
| Hong Kong Street Beef Flavoured Noodles | Unhealthy | Unhealthy |
| Zen Garden Vegetables Flavoured Noodles | Unhealthy | Healthy |
| Fresh Udon Noodles | Healthy | Healthy |
| Fresh Rice Noodles | Healthy | Healthy |
| Spicy Flavoured Mini Instant Noodles | Unhealthy | Unhealthy |
| Carbo Hot Chicken Flavour Ramen | Unhealthy | Unhealthy |
| An Sung Tang Myun Spicy Miso Noodle Soup | Unhealthy | Unhealthy |
| Medium Egg Noodles | Unhealthy | Healthy |
| Original Kelp Noodles | Unhealthy | Healthy |
| Kona Berry Kelp Noodles | Unhealthy | Healthy |
| Chicken Flavoured Noodles | Unhealthy | Unhealthy |
| Rice Vermicelli | Healthy | Healthy |
| Mi Goreng Hot & Spicy Noodles | Unhealthy | Unhealthy |
| Organic Handmade Spinach Noodle | Healthy | Healthy |
| Organic Red Rice Noodles with Chia | Healthy | Healthy |
| Egg Noodles | Unhealthy | Unhealthy |
| Wok-Ready Rice Noodles | Healthy | Healthy |
| Edamame Organic Asian Noodles | Healthy | Healthy |
| Roasted Vermicelli | Healthy | Healthy |
| Organic Brown Rice Stir Fry Noodles | Healthy | Healthy |
| Chicken Flavoured Noodles | Unhealthy | Unhealthy |
| Beef Flavoured Noodles | Unhealthy | Unhealthy |
| Mi Goreng Soy & Mild Spice Noodles | Unhealthy | Unhealthy |
| Fresh Rice Roll Noodle | Healthy | Healthy |
| Pho Bo Beef Flavour Vietnamese Style Instant Rice Noodles | Unhealthy | Unhealthy |
| Fresh Egg Noodles | Unhealthy | Unhealthy |
| Wok-Ready Thin Hokkien Noodles | Unhealthy | Unhealthy |
| Organic Purple Sweet Potato Noodle | Healthy | Healthy |
| Vegetable Laksa Noodles | Unhealthy | Unhealthy |
| Beef Flavoured Noodle Cup | Unhealthy | Unhealthy |
| Asian Hokkien Noodles with Black Sesame & Kale | Unhealthy | Unhealthy |
| Beef Flavour Brown Rice Noodles | Unhealthy | Unhealthy |
| Chicken Flavour Brown Rice Noodles | Unhealthy | Unhealthy |
| Fresh Pho Noodles | Healthy | Healthy |
| Curry Flavoured Instant Noodles | Unhealthy | Unhealthy |
| Seafood & Spicy Ramyun | Unhealthy | Unhealthy |
| Noodle, wheat, Asian style, cooked | Healthy | Healthy |
| Noodle, wheat with egg, plain, dry | Healthy | Healthy |
| Noodle, wheat with egg, plain, boiled, no added fat | Healthy | Healthy |
| **Pasta** | | |
| Creamy Cheese Pasta | Unhealthy | Unhealthy |
| Pasta with Truffle | Healthy | Healthy |
| Quinoa Pasta Elbows | Unhealthy | Healthy |
| Saffron Twists | Unhealthy | Healthy |
| Organic Brown Rice Twists | Unhealthy | Healthy |
| Four Cheeses Pasta & Sauce | Unhealthy | Unhealthy |
| Mushroom, Garlic & Black Pepper Pasta & Sauce | Unhealthy | Unhealthy |
| Pasta Shells | Healthy | Healthy |
| Rigatoni Corn Pasta | Healthy | Healthy |
| Gluten Free Buckwheat Fettuccine Pasta | Healthy | Healthy |
| Gluten Free Fettuccine Pasta | Unhealthy | Healthy |
| Organic Spelt Fusilli | Healthy | Healthy |
| Gluten & Wheat Free Rice Macaroni | Unhealthy | Healthy |
| Gluten & Wheat Free Conchiglie Pasta | Unhealthy | Healthy |
| Creamy Tomato, Parmesan and Basil Pasta | Unhealthy | Unhealthy |
| Primavera with Vine-Ripened Tomato Pasta | Unhealthy | Unhealthy |
| Creamy Chicken Pesto and Spring Onion Pasta | Unhealthy | Unhealthy |
| Creamy Spinach, Parmesan and Bacon Pasta | Unhealthy | Unhealthy |
| Tomato & Onion Pasta | Unhealthy | Unhealthy |
| Mushroom Risoni | Unhealthy | Unhealthy |
| Classic Cheese Pasta & Sauce Microwaveable Meal | Unhealthy | Unhealthy |
| Liscio Piccolo 4Healthy Pasta | Healthy | Healthy |
| Bavarian Style Spaetzle Pasta | Healthy | Healthy |
| Aged Cheddar Parmesan & Chive Pasta | Unhealthy | Unhealthy |
| Spaghetti | Unhealthy | Healthy |
| Wholemeal Lasagna Sheets | Healthy | Healthy |
| Lasagne Sheets | Unhealthy | Unhealthy |
| Organic Kamut Conchieglie Pasta | Healthy | Healthy |
| Fresh Agnolotti with Ricotta, Spinach & Parmesan | Unhealthy | Unhealthy |
| Fresh Agnolotti with Chicken, Garlic & Italian Herbs | Unhealthy | Unhealthy |
| Traditional Spinach & Ricotta Cheese Ravioli | Healthy | Healthy |
| Pesto 3 Min Pasta Meal | Unhealthy | Unhealthy |
| Creamy Cheese & Chive Flavoured Pasta & Sauce | Unhealthy | Unhealthy |
| Trottole Tricolour Gourmet Pasta | Healthy | Healthy |
| Tomato, Onion & Herbs 3 Min Pasta Meal | Unhealthy | Unhealthy |
| Tomato, Garlic & Basil 3 Min Pasta Meal | Unhealthy | Unhealthy |
| Spicy Tomato 3 Min Pasta Meal | Unhealthy | Unhealthy |
| Chicken Parmigiana Ravioli | Unhealthy | Healthy |
| Chicken & Porcini Mushroom Ravioli | Unhealthy | Unhealthy |
| Spinach & Handmade Ricotta Ravioli | Unhealthy | Unhealthy |
| Roast Chicken & Garlic Tortellini | Unhealthy | Unhealthy |
| Mung Bean Fettuccine | Healthy | Healthy |
| Black Olive Fussilli | Healthy | Healthy |
| Egg Pasta Spaghettini | Unhealthy | Unhealthy |
| Authentic Fusilli Pasta | Healthy | Healthy |
| Traditional Potato Gnocchi Pasta | Unhealthy | Unhealthy |
| Tomato Mini Penne | Unhealthy | Unhealthy |
| Arrabbiata Spirals | Unhealthy | Unhealthy |
| Tagliatelle Egg Pasta | Healthy | Healthy |
| Rigatoni di Gragnano | Healthy | Healthy |
| Spaghetti | Healthy | Healthy |
| Cheese Filled Tortellini | Unhealthy | Unhealthy |
| Tomato & Mozzarella Filled Tortellini | Unhealthy | Unhealthy |
| Gluten Free Spinach Fettucini | Unhealthy | Healthy |
| Ricotta & Spinach Agnolotti | Unhealthy | Unhealthy |
| Beef Ravioli | Unhealthy | Unhealthy |
| Lasagne Sheets | Healthy | Healthy |
| Ravioli Grandi with Italian Sausage & Fennel | Unhealthy | Healthy |
| Ravioliacci with King Island Beef Ragu | Unhealthy | Unhealthy |
| Potatoes Gnocchi | Unhealthy | Unhealthy |
| Spirals | Healthy | Healthy |
| Spicy Tomato Pasta Snack | Unhealthy | Unhealthy |
| Squid Ink Spaghetti | Healthy | Healthy |
| Fusilli Tris Pasta | Healthy | Healthy |
| Roasted Butternut Pumpkin Chunky Gnocchi | Healthy | Healthy |
| Fresh Egg Fettuccine | Healthy | Healthy |
| Australian Beef Ravioli | Unhealthy | Unhealthy |
| Spinach & Ricotta Agnolotti | Unhealthy | Unhealthy |
| Roast Pumpkin & Aged Grana Padana Agnolotti | Unhealthy | Unhealthy |
| Four Cheese Ravioli | Unhealthy | Unhealthy |
| Ricotta & Spinach Tortellini | Unhealthy | Unhealthy |
| Roast Beef, Caramelised Onion and Red Wine Cappelletti | Unhealthy | Unhealthy |
| Four Cheese Tortellini | Unhealthy | Unhealthy |
| Macaroni Cheese Pasta Snack | Unhealthy | Unhealthy |
| Creamy Bacon Carbonara Pasta Snack | Unhealthy | Unhealthy |
| Classic Macaroni & Cheese | Unhealthy | Unhealthy |
| Lasagne Sheets | Healthy | Healthy |
| Pumpkin & Feta Agnolotti | Unhealthy | Unhealthy |
| Alfredo Pasta Snack | Unhealthy | Unhealthy |
| Gluten Free Original Penne Pasta | Healthy | Healthy |
| Roast Pumpkin & Sage Ravioli Fresh Pasta | Unhealthy | Healthy |
| Vermicelli | Healthy | Healthy |
| Wild Mushroom & Black Truffle Ravioli | Unhealthy | Unhealthy |
| Certified Organic Black Bean Spaghetti | Healthy | Healthy |
| Certified Organic Chickpea Fettuccine | Healthy | Healthy |
| Giant Spirals No. 552 | Healthy | Healthy |
| Fresh Potato Gnocchi with Asiago Cheese and Porcini Mushrooms | Unhealthy | Unhealthy |
| Squid Ink Linguine Pasta | Healthy | Healthy |
| Gnocchi made with Steamed Fresh Potatoes and Pumpkin | Unhealthy | Unhealthy |
| Free Range Egg Mezzalune with Italian Style Pork & Fennel | Unhealthy | Healthy |
| Free Range Egg Triangoli with Porcini Mushroom & Grana Padano | Unhealthy | Healthy |
| Penna Pasta with Mediterranean Vegetables in Napoli Sauce | Unhealthy | Healthy |
| Tortellone Wagyu Beef | Unhealthy | Healthy |
| Spirals Pasta | Healthy | Healthy |
| Sweet Potato & Parmesan Ravioli | Unhealthy | Healthy |
| Pumpkin & Spinach Linguine | Healthy | Healthy |
| Ricotta & Spinach Filled Ravioli | Unhealthy | Healthy |
| Ricotta & Spinach Agnolotti | Unhealthy | Unhealthy |
| Magnifico Tomato & Herb Pasta | Unhealthy | Unhealthy |
| Soul-Full Cajun Pasta | Unhealthy | Unhealthy |
| Cheez 'n Chive Flavour Mac n Cheez | Healthy | Healthy |
| Chilli Cheez Flavour Mac n Cheez | Healthy | Healthy |
| Original Cheez Flavour Mac N Cheez Pasta Meal | Healthy | Healthy |
| Porcini and Cheez Flavour Mac N Cheez Pasta Meal | Healthy | Healthy |
| Beef Ravioli | Unhealthy | Unhealthy |
| Egg Lasagne No.112 Pasta | Healthy | Healthy |
| Lemon & Parsley Linguine | Unhealthy | Healthy |
| Pasta, white wheat flour, plain, dry | Healthy | Healthy |
| Pasta, white wheat flour, plain, boiled from dry, no added salt | Healthy | Healthy |
| Noodle, wheat, Asian style, cooked | Healthy | Healthy |
| Pasta, white wheat flour, plain, fresh, uncooked | Healthy | Healthy |
| Pasta, white wheat flour, plain, fresh, boiled, no added salt | Healthy | Healthy |
| Pasta, white wheat flour & egg, plain, dry | Healthy | Healthy |
| Pasta, white wheat flour & egg, plain, boiled, no added salt | Healthy | Healthy |
| Pasta, white wheat flour & egg, plain, boiled, with added salt | Healthy | Healthy |
| Pasta, white wheat flour & spinach, plain, dry | Healthy | Healthy |
| Pasta, white wheat flour & spinach, plain, boiled, no added salt | Healthy | Healthy |
| Pasta, white wheat flour & spinach, plain, boiled, with added salt | Healthy | Healthy |
| Pasta, wholemeal wheat flour, plain, dry | Healthy | Healthy |
| Pasta, wholemeal wheat flour, plain, boiled from dry, no added salt | Healthy | Healthy |
| Pasta, wholemeal wheat flour, plain, boiled from dry, with added salt | Healthy | Healthy |
| Noodle, wheat, instant, unflavoured, dry, uncooked | Unhealthy | Unhealthy |
| Noodle, wheat, instant, unflavoured, boiled, drained | Unhealthy | Healthy |
| Noodle, wheat, instant, flavoured, dry, uncooked | Unhealthy | Unhealthy |
| Noodle, wheat, instant, flavoured, boiled, drained | Unhealthy | Unhealthy |
| Noodle, wheat, instant, low fat, unflavoured, boiled, drained | Unhealthy | Healthy |
| Noodle, wheat, instant, low fat, flavoured, boiled, drained | Unhealthy | Healthy |
| Noodle, wheat, instant, not further defined | Unhealthy | Unhealthy |
| Noodle, buckwheat or soba, dry | Unhealthy | Unhealthy |
| Noodle, buckwheat or soba, boiled, drained | Healthy | Healthy |
| Noodle, rice stick, boiled, drained | Healthy | Healthy |
| Pasta, gluten free, plain, boiled from dry, no added salt | Healthy | Healthy |
| Pasta, gluten free, plain, boiled from dry, with added salt | Healthy | Healthy |
| Pasta, maize flour (corn) based, plain, dry | Healthy | Healthy |
| Pasta, maize flour (corn) based, plain, cooked | Healthy | Healthy |
| Pasta, filled with meat, fresh, commercial, boiled, without added sauce | Unhealthy | Unhealthy |
| Pasta, filled with vegetables, fresh, commercial, boiled, without added sauce | Unhealthy | Unhealthy |
| Pasta in cream based sauce, dry mix only | Unhealthy | Unhealthy |
| Pasta in tomato based sauce, dry mix | Unhealthy | Unhealthy |
| Gnocchi, potato, commercially prepared, boiled | Unhealthy | Unhealthy |
| **Quinoa and other cereals** | | |
| Quinoa Cups | Unhealthy | Healthy |
| Red Quinoa | Healthy | Healthy |
| Pearl & Black Barley | Healthy | Healthy |
| Raw Buckwheat | Healthy | Healthy |
| Stone Milled Coarse Bulgur | Healthy | Healthy |
| Organic Tricolour Quinoa | Healthy | Healthy |
| Persian Quinoa with Dates and Pistachio | Unhealthy | Healthy |
| White Polenta with Truffle | Unhealthy | Unhealthy |
| Pearl Barley | Healthy | Healthy |
| Thai Coconut & Chilli Quinoa | Unhealthy | Unhealthy |
| Tomato, Garlic & Parsley Quinoa | Unhealthy | Unhealthy |
| Lupin Flakes | Healthy | Healthy |
| Black Truffle Polenta | Healthy | Healthy |
| Amaranth | Healthy | Healthy |
| Thai Flavoured Organic Royal Quinoa | Unhealthy | Unhealthy |
| Australian Polenta | Healthy | Healthy |
| Coarse Semolina | Healthy | Healthy |
| Steamed White Quinoa | Healthy | Healthy |
| Brown Grain Teff | Healthy | Healthy |
| Premium Selected Black Barley | Healthy | Healthy |
| Organic Tri Colour Quinoa | Healthy | Healthy |
| Barley, pearl, uncooked | Healthy | Healthy |
| Barley, pearl, cooked in water, no added fat or salt | Healthy | Healthy |
| BarleyMax, dry, uncooked | Healthy | Healthy |
| Buckwheat groats, uncooked | Healthy | Healthy |
| Bulgur (burghul, burgaul), dry, uncooked | Healthy | Healthy |
| Bulgur (burghul, burgaul), soaked in water, no added fat or salt | Healthy | Healthy |
| Bulgur (burghul, burgaul), cooked in water, no added fat or salt | Healthy | Healthy |
| Cornmeal (polenta), uncooked | Healthy | Healthy |
| Millet, uncooked | Healthy | Healthy |
| Millet, boiled in water, no added fat or salt | Healthy | Healthy |
| Quinoa, uncooked | Healthy | Healthy |
| Rye, uncooked | Healthy | Healthy |
| Sago, dry, uncooked | Healthy | Healthy |
| Spelt, uncooked | Healthy | Healthy |
| Wheat bran, unprocessed, uncooked | Healthy | Healthy |
| Wheat germ | Healthy | Healthy |
| Semolina, uncooked | Healthy | Healthy |
| Tapioca, pearl or seed style, uncooked | Healthy | Healthy |
| Tapioca | Healthy | Healthy |
| **Rice** | | |
| Lemon Risotto with Carnaroli Rice | Healthy | Healthy |
| Mountain Blend Rice and Grains | Healthy | Healthy |
| Steamed Brown Rice Quick Cup | Unhealthy | Healthy |
| Long Grain Steamed Rice | Unhealthy | Healthy |
| Thai Coconut & Lemon Grass Rice | Unhealthy | Unhealthy |
| Special Fried Rice | Unhealthy | Unhealthy |
| Mushroom & Chive Risotto | Unhealthy | Unhealthy |
| Brown Rice & Chia | Unhealthy | Healthy |
| Mexican Flavoured Rice | Unhealthy | Unhealthy |
| Vegetable Fried Rice | Unhealthy | Healthy |
| Tully Grown Rain Fed Tropical North Queensland Brown Wholegrain Rice | Healthy | Healthy |
| Brown Rice & Chia Seed | Unhealthy | Healthy |
| Mexican Style Brown Rice | Unhealthy | Unhealthy |
| Porcini Mushrooms Risotto Mix | Unhealthy | Unhealthy |
| Tomato & Basil Brown Rice & Quinoa | Unhealthy | Unhealthy |
| Brown Medium Grain Steamed Rice | Healthy | Healthy |
| Pumpkin, Bacon & Sour Cream Risotto | Unhealthy | Unhealthy |
| Roast Chicken & Leek Risotto | Unhealthy | Unhealthy |
| Paella with Smoked Paprika Rice | Unhealthy | Unhealthy |
| Chicken Porcini Mushroom & Garlic Rice & Quinoa | Unhealthy | Unhealthy |
| Carnaroli Rice | Healthy | Healthy |
| Carnaroli Rice with Vegetables | Unhealthy | Healthy |
| Edamame, Spring Onion & Wasabi with Wholegrain Basmati Rice | Unhealthy | Healthy |
| Black Bean, Jerk & Coconut with Wholegrain Basmati Rice | Unhealthy | Healthy |
| Split Pea, Green Chilli & Coriander with Wholegrain Basmati Rice | Unhealthy | Healthy |
| Tasmanian Native Pepperberry & Beetroot Risotto Rice | Healthy | Healthy |
| Tasmanian Mushroom and Black Truffle Risotto Rice | Unhealthy | Healthy |
| Tasmanian Goat Fetta and Pumpkin Risotto Rice | Healthy | Healthy |
| Arborio Risotto Rice | Healthy | Healthy |
| Basmati Rice | Unhealthy | Unhealthy |
| Mexican Rice & Beans | Unhealthy | Unhealthy |
| Coconut Rice | Unhealthy | Unhealthy |
| Gluten Free Super Duo | Healthy | Healthy |
| Australian Brown Rice | Healthy | Healthy |
| Organic Quinoa & Brown Rice with Garlic | Unhealthy | Unhealthy |
| Indian Rice, Cous Cous, & Lentil Snack Pot | Unhealthy | Unhealthy |
| Singapore Aromatic Spice Rice | Unhealthy | Unhealthy |
| Jasmine Fragrant Rice | Healthy | Healthy |
| Wild Rice | Healthy | Healthy |
| Fragrant Jasmine Rice | Healthy | Healthy |
| Australian Medium Grain Calrose Rice | Healthy | Healthy |
| Brown Rice | Unhealthy | Healthy |
| Basmati Rice | Unhealthy | Healthy |
| All Natural Wholegrain Five Blend | Healthy | Healthy |
| Medium Grain Rice | Healthy | Healthy |
| Brown & Red Rice with Quinoa & Linseed | Unhealthy | Unhealthy |
| Sweet Potato Rice | Healthy | Healthy |
| Coconut, Chilli & Lemongrass Rice | Unhealthy | Healthy |
| Coconut Basmati Rice | Healthy | Healthy |
| Basmati Rice | Healthy | Healthy |
| Rice, wild, uncooked | Healthy | Healthy |
| Rice, wild, boiled, no added salt | Healthy | Healthy |
| Rice bran, extruded or low processed, uncooked | Healthy | Healthy |
| Rice, white, uncooked | Healthy | Healthy |
| Rice, white, boiled, no added salt | Healthy | Healthy |
| Rice, white, steamed or rice cooker, no added salt | Healthy | Healthy |
| Rice, brown, uncooked | Healthy | Healthy |
| Rice, brown, boiled, no added salt | Healthy | Healthy |
| Rice, flavoured, instant dry mix | Unhealthy | Unhealthy |
| Rice, flavoured, prepared from dry mix | Unhealthy | Unhealthy |
| Rice, purchased as parboiled (gold rice), uncooked | Healthy | Healthy |
| Rice, purchased as parboiled (gold rice), boiled, no added salt | Healthy | Healthy |
| Rice, red, steamed or rice cooker, no added salt | Healthy | Healthy |
| Rice paper wrapper, soaked in water | Unhealthy | Unhealthy |
| Sushi, California roll, commercial | Healthy | Healthy |
| **Confectionery** | | |
| **Chocolates and sweets** | | |
| Barley Sugar Glucose Travel Sweets | Unhealthy | Unhealthy |
| Peppermint Flavoured Sugar Free Mints | Unhealthy | Unhealthy |
| Milk Chocolate Coated Apricots | Unhealthy | Unhealthy |
| Chocolate Nougat Praliné Bunnies | Unhealthy | Unhealthy |
| Milk Chocolate Doctor Bunny | Unhealthy | Unhealthy |
| Mint Pattie | Unhealthy | Unhealthy |
| DC Comics Milk Chocolate Easter Egg | Unhealthy | Unhealthy |
| No Sugar Added Creamy Milk Chocolate | Unhealthy | Unhealthy |
| Turkish Delight Eggs | Unhealthy | Unhealthy |
| Candy Floss Flavoured Round Lollipop | Unhealthy | Unhealthy |
| Citrus Grove Vitamin C Drops | Unhealthy | Unhealthy |
| Milk Flavoured Creamy Candy | Unhealthy | Unhealthy |
| Mint Crisp Flake 55% Cocoa Dark Chocolate | Unhealthy | Unhealthy |
| Choc Coconut & Cherry Snack Bar | Unhealthy | Unhealthy |
| No Added Sugar Rich Dark Chocolate with 70% Cocoa | Unhealthy | Unhealthy |
| Lemon & Lime Flavoured Sugarfree Chewing Gum | Unhealthy | Unhealthy |
| Chocolate Coated Honeycombs | Unhealthy | Unhealthy |
| Strawberry Flavoured Crunchy Mallow Fizz | Unhealthy | Unhealthy |
| Peppermint Mints | Unhealthy | Unhealthy |
| Organic Dark Hazelnut & Sea Salt Chocolate | Unhealthy | Unhealthy |
| Vanilla Mousse Snowman | Unhealthy | Unhealthy |
| Cheeky Chimp Honeycomb Bites | Unhealthy | Unhealthy |
| Orange Infused Milk Chocolate | Unhealthy | Unhealthy |
| Traditional Milk Chocolate | Unhealthy | Unhealthy |
| Sweetness Raspberry Bananas | Unhealthy | Unhealthy |
| Premium Dark Chocolate with a Chocolate Mousse and Orange Filling | Unhealthy | Unhealthy |
| Milk Chocolate Coated Salted Caramel Scorched Almonds | Unhealthy | Unhealthy |
| Banana Flavoured Mints | Unhealthy | Unhealthy |
| Chocolate Bar | Unhealthy | Unhealthy |
| Nut Delight Sea Salt Caramel with Dark Choc Bar | Unhealthy | Unhealthy |
| 55% Dark Chocolate Truffles | Unhealthy | Unhealthy |
| Irresistibly Smooth White Peppermint Chocolate | Unhealthy | Unhealthy |
| Unhealthy Euro Coin Chocolate | Unhealthy | Unhealthy |
| Dream Top Chocolate Xmas Trees | Unhealthy | Unhealthy |
| Extra Dark 85% Chocolate | Unhealthy | Unhealthy |
| Dark Chocolate Melts | Unhealthy | Unhealthy |
| Hazelnut Chocolate | Unhealthy | Unhealthy |
| Peanut & Chocolate Bars | Unhealthy | Unhealthy |
| 80% Cocoa Chocolate | Unhealthy | Unhealthy |
| Sherbet Straws | Unhealthy | Unhealthy |
| Peppermint Place | Unhealthy | Unhealthy |
| Probiotic Chocolate Bars | Unhealthy | Unhealthy |
| Coconut Rough Chocolate Balls | Unhealthy | Unhealthy |
| Chocolate Truffle | Unhealthy | Unhealthy |
| Fruity Burst Chewing Gum | Unhealthy | Unhealthy |
| Liquorice with Dark Chocolate | Unhealthy | Unhealthy |
| Piñata Mix | Unhealthy | Unhealthy |
| Fizzers | Unhealthy | Unhealthy |
| The Best of Cola Fruit Creamy Lollipops | Unhealthy | Unhealthy |
| Eucalyptus Drops | Unhealthy | Unhealthy |
| Milk Chocolate Covered Peanuts in a Thin Crisp Shell | Unhealthy | Unhealthy |
| Marshmallow Eggs | Unhealthy | Unhealthy |
| Milk Chocolate with Cornflakes | Unhealthy | Unhealthy |
| Liquorice Coated in Milk Chocolate Balls | Unhealthy | Unhealthy |
| Hand Rolled Dark Chocolate Egg | Unhealthy | Unhealthy |
| Milk Chocolate Egg with Honeycomb | Unhealthy | Unhealthy |
| Almond, Turmeric, Coconut & Yoghurt Bar | Unhealthy | Unhealthy |
| Milk Chocolate Easter Eggs | Unhealthy | Unhealthy |
| Handmade Coconut Ice | Unhealthy | Unhealthy |
| Chocolate Bites | Unhealthy | Unhealthy |
| Choclettes | Unhealthy | Unhealthy |
| Dairy Milk Milk Chocolate with Vanilla Flavoured Creme, Smooth Flowing Caramel & Peanut Pieces | Unhealthy | Unhealthy |
| Dark Chocolate | Unhealthy | Unhealthy |
| Milk Chocolate Chips | Unhealthy | Unhealthy |
| Raspberry Caramel Popcorn Rocky Road Chocolate | Unhealthy | Unhealthy |
| Toasted Marshmallow | Unhealthy | Unhealthy |
| Java Single Origin Milk Chocolate | Unhealthy | Unhealthy |
| Marshmallow Ice-Creams | Unhealthy | Unhealthy |
| Super Strawberry Flavour Twist Candy | Unhealthy | Unhealthy |
| Strawberry Tiny Tasty Chewy Sweets | Unhealthy | Unhealthy |
| Triple Chocolate Bar | Unhealthy | Unhealthy |
| Soft and Creamy Banana Flavoured and Shaped Treat | Unhealthy | Unhealthy |
| Almond Roasted Nut Bar with Milk Chocolate | Unhealthy | Unhealthy |
| Dark Chocolate Coconut Clusters Cherry + Almond Butter | Unhealthy | Unhealthy |
| Milk Chocolate Truffles | Unhealthy | Unhealthy |
| Mini Bon Bons | Unhealthy | Unhealthy |
| Choc Bullets | Unhealthy | Unhealthy |
| Berry Chocolates | Unhealthy | Unhealthy |
| Milk Chocolate Caramel Pastilles | Unhealthy | Unhealthy |
| Dark Ginger Chocolates | Unhealthy | Unhealthy |
| Assorted Fruit Hard Candies | Unhealthy | Unhealthy |
| No Sugar Added Chocolate Truffle in Milk Chocolate | Unhealthy | Unhealthy |
| Dark Chocolate Mini Easter Eggs with 70% Cocoa | Unhealthy | Unhealthy |
| Rocky Road Bar | Unhealthy | Unhealthy |
| Milk Chocolate Koala | Unhealthy | Unhealthy |
| Hot Cross Bun Chocolates | Unhealthy | Unhealthy |
| Golden Honeycomb Egg | Unhealthy | Unhealthy |
| Berry Mallow Egg | Unhealthy | Unhealthy |
| Milk Chocolate Turkish Delight Mini Eggs | Unhealthy | Unhealthy |
| White, Rose & Cardamom Easter Egg with Mini Eggs | Unhealthy | Unhealthy |
| Grand Crus Chocolate Eggs | Unhealthy | Unhealthy |
| Popping Candy Easter Egg | Unhealthy | Unhealthy |
| Luxury Salted Caramel Truffle Eggs | Unhealthy | Unhealthy |
| Tangerine Candy | Unhealthy | Unhealthy |
| Mixed Wild Berries Candy | Unhealthy | Unhealthy |
| Peppermint Filled Dark Chocolate Mini Eggs | Unhealthy | Unhealthy |
| Giant Easter Egg Tube | Unhealthy | Unhealthy |
| Spearmint Sugar Free Mints | Unhealthy | Unhealthy |
| Rose, Pomegranate & Lemon Mixed Flavour Turkish Delight | Unhealthy | Unhealthy |
| Milk Chocolate Half Egg with Scorched Almonds | Unhealthy | Unhealthy |
| Choc Coated Protein Hazelnuts | Unhealthy | Unhealthy |
| Belgian Hazelnut Chocolate | Unhealthy | Unhealthy |
| Milk Coconut Square | Unhealthy | Unhealthy |
| Organic Salted Caramel Swayzee Chocolate | Unhealthy | Unhealthy |
| Choc Chips Cooking Chocolate | Unhealthy | Unhealthy |
| Native Quartet Box Chocolates | Unhealthy | Unhealthy |
| Salted Caramel White Chocolate | Unhealthy | Unhealthy |
| White Chocolate & Raspberry Bites | Unhealthy | Unhealthy |
| Salted Caramel Fudge Cheesecake Milk Chocolate | Unhealthy | Unhealthy |
| Chocolate Christmas Characters with Hazelnut Filling | Unhealthy | Unhealthy |
| Merry Breakmas Chocolate Santa | Unhealthy | Unhealthy |
| 100s & 100s Belgian Chocolate Tree | Unhealthy | Unhealthy |
| Christmas Pudding Brandy Truffles | Unhealthy | Unhealthy |
| White Chocolate Magic Pudding Bites | Unhealthy | Unhealthy |
| Christmas Hazelnuts | Unhealthy | Unhealthy |
| Fruit and Cola Flavour Chewy Sweets | Unhealthy | Unhealthy |
| Finest Chocolate Ganache Baubles | Unhealthy | Unhealthy |
| Fruit & Nut Milk Chocolate | Unhealthy | Unhealthy |
| Turkish Delight with Pomegranate & Pistachio Flavouring | Unhealthy | Unhealthy |
| Real Milk Chocolate Baking Chips | Unhealthy | Unhealthy |
| Nut Clusters | Unhealthy | Unhealthy |
| Belgian Milk Chocolate Lollipop | Unhealthy | Unhealthy |
| Festive Bauble with Pralines and Chocolates | Unhealthy | Unhealthy |
| Chocolate Sticks with Liquid Kirsch Filling | Unhealthy | Unhealthy |
| Chocolate, compound, cooking | Unhealthy | Unhealthy |
| Chocolate, dark, high cocoa solids, less than 60% cocoa solids | Unhealthy | Unhealthy |
| Chocolate, dark, high cocoa solids, 60% cocoa solids or greater | Unhealthy | Unhealthy |
| Chocolate, milk | Unhealthy | Unhealthy |
| Chocolate, white | Unhealthy | Unhealthy |
| Chocolate, milk & white | Unhealthy | Unhealthy |
| Chocolate, plain, not further defined | Unhealthy | Unhealthy |
| Chocolate, reduced sugar | Unhealthy | Unhealthy |
| Confectionery, truffle | Unhealthy | Unhealthy |
| Bar, cherry & coconut centre, dark chocolate-coated | Unhealthy | Unhealthy |
| Bar, coconut cream centre, milk chocolate-coated | Unhealthy | Unhealthy |
| Bar, nougat, caramel & peanut centre, milk chocolate-coated | Unhealthy | Unhealthy |
| Bar, nougat & nut centre, chocolate-coated | Unhealthy | Unhealthy |
| Bar, wafer & hazelnut, milk chocolate-coated | Unhealthy | Unhealthy |
| Bar, wafer or biscuit, caramel & peanut, milk chocolate-coated | Unhealthy | Unhealthy |
| Chocolate, dark, with dried fruit & nuts | Unhealthy | Unhealthy |
| Chocolate, dark, with nuts | Unhealthy | Unhealthy |
| Chocolate, milk, with coconut | Unhealthy | Unhealthy |
| Chocolate, milk, with dried fruit & nuts | Unhealthy | Unhealthy |
| Chocolate, milk, with hazelnut paste | Unhealthy | Unhealthy |
| Chocolate, milk, with nuts | Unhealthy | Unhealthy |
| Chocolate, white, with macadamias | Unhealthy | Unhealthy |
| Confectionery, almond, chocolate-coated | Unhealthy | Unhealthy |
| Confectionery, chocolate, hazelnut & wafer centre | Unhealthy | Unhealthy |
| Confectionery, chocolate & peanut centre, sugar-coated | Unhealthy | Unhealthy |
| Confectionery, dried fruit & nuts, chocolate-coated | Unhealthy | Unhealthy |
| Confectionery, mixed nuts, chocolate-coated | Unhealthy | Unhealthy |
| Confectionery, peanut brittle | Unhealthy | Unhealthy |
| Confectionery, peanut, chocolate-coated | Unhealthy | Unhealthy |
| Confectionery, rocky road | Unhealthy | Unhealthy |
| Bar, chocolate nougat centre, milk chocolate-coated | Unhealthy | Unhealthy |
| Bar, honeycomb centre, milk chocolate-coated | Unhealthy | Unhealthy |
| Bar, nougat & caramel centre, milk chocolate-coated | Unhealthy | Unhealthy |
| Bar, nougat & caramel centre, milk chocolate coated, battered, deep fried | Unhealthy | Unhealthy |
| Bar, Turkish delight centre, milk chocolate-coated | Unhealthy | Unhealthy |
| Bar, wafer & cream layers, chocolate-coated | Unhealthy | Unhealthy |
| Bar, wafer & fudge, milk chocolate-coated | Unhealthy | Unhealthy |
| Bar, wafer or biscuit & caramel, milk chocolate-coated | Unhealthy | Unhealthy |
| Chocolate, chocolate cream centre | Unhealthy | Unhealthy |
| Chocolate, dark, fondant filled | Unhealthy | Unhealthy |
| Chocolate, dark, peppermint filled | Unhealthy | Unhealthy |
| Chocolate, dark, with dried fruit | Unhealthy | Unhealthy |
| Chocolate, liqueur-filled | Unhealthy | Unhealthy |
| Chocolate, milk, caramel filled | Unhealthy | Unhealthy |
| Chocolate, milk, fondant filled | Unhealthy | Unhealthy |
| Chocolate, milk, with toffee or nougat pieces | Unhealthy | Unhealthy |
| Chocolate, favourites | Unhealthy | Unhealthy |
| Chocolate, boxed assorted, not further defined | Unhealthy | Unhealthy |
| Chocolate, not further defined | Unhealthy | Unhealthy |
| Confectionery, chocolate centre, sugar-coated | Unhealthy | Unhealthy |
| Confectionery, coffee beans, chocolate-coated | Unhealthy | Unhealthy |
| Confectionery, ginger, chocolate-coated | Unhealthy | Unhealthy |
| Confectionery, malt centre, chocolate coated | Unhealthy | Unhealthy |
| Confectionery, marshmallow-filled, chocolate & coconut coated (snowball) | Unhealthy | Unhealthy |
| Confectionery, sultana, chocolate-coated | Unhealthy | Unhealthy |
| Easter egg, milk, fondant filled | Unhealthy | Unhealthy |
| Licorice, chocolate-coated | Unhealthy | Unhealthy |
| Bar, carob | Unhealthy | Unhealthy |
| Fruit, leather | Unhealthy | Unhealthy |
| Fruit bar, apricot, snack or confectionery style | Unhealthy | Unhealthy |
| Fruit bar, 95% fruit, snack or confectionery style, added fibre & vitamin C & folate | Unhealthy | Unhealthy |
| Almond, sugar-coated | Unhealthy | Unhealthy |
| Confectionery, sesame & toffee | Unhealthy | Unhealthy |
| Halvah, plain | Unhealthy | Unhealthy |
| Nougat, honey & nuts, traditional | Unhealthy | Unhealthy |
| Bar, snack style, chocolate flavour, space stick | Unhealthy | Unhealthy |
| Caramels, soft | Unhealthy | Unhealthy |
| Caramels, hard | Unhealthy | Unhealthy |
| Licorice, black | Unhealthy | Unhealthy |
| Licorice, flavoured | Unhealthy | Unhealthy |
| Licorice, allsorts | Unhealthy | Unhealthy |
| Lolly, boiled | Unhealthy | Unhealthy |
| Lolly, boiled, chocolate filled | Unhealthy | Unhealthy |
| Lolly, butterscotch | Unhealthy | Unhealthy |
| Lolly, fruit flavoured, chewy | Unhealthy | Unhealthy |
| Lolly, medicated cough lolly or lozenge | Unhealthy | Unhealthy |
| Lolly, mint flavoured, sugar sweetened | Unhealthy | Unhealthy |
| Lolly, sour, all varieties | Unhealthy | Unhealthy |
| Lollipop or chupa chup | Unhealthy | Unhealthy |
| Marshmallow, plain or flavoured | Unhealthy | Unhealthy |
| Sherbet powder | Unhealthy | Unhealthy |
| Lolly, mint flavoured, intense sweetened | Unhealthy | Unhealthy |
| Lolly, non-mint flavours, intense sweetened | Unhealthy | Unhealthy |
| Chewing gum, regular | Unhealthy | Unhealthy |
| Chewing gum, intense sweetened | Unhealthy | Unhealthy |
| Confectionery, chocolate crackle | Unhealthy | Unhealthy |
| Confectionery, honey joy | Unhealthy | Unhealthy |
| Ginger, crystallised | Unhealthy | Unhealthy |
| Honeycomb, plain | Unhealthy | Unhealthy |
| **Jelly** | | |
| Crazy Plum Babies with Bellies | Unhealthy | Unhealthy |
| My Heart Needs Blood Orange Babies with Bellies | Unhealthy | Unhealthy |
| The Chia Protein Jelly Snack with Peach Flavour | Unhealthy | Unhealthy |
| Cherry Jelly | Unhealthy | Unhealthy |
| Frosty Sour Bears | Unhealthy | Unhealthy |
| Lemon and Lime Jelly | Unhealthy | Unhealthy |
| Sour Neon Dinosaurs | Unhealthy | Unhealthy |
| Gummy Worms | Unhealthy | Unhealthy |
| Strawberries, Apple and Chamomile Wellness Jelly | Unhealthy | Healthy |
| Strawberry Flavour Jelly Crystals with Edible Glitter | Unhealthy | Unhealthy |
| Sour Dummies | Unhealthy | Unhealthy |
| Mango Passionfruit Flavour Low Calorie Jelly Crystals | Unhealthy | Unhealthy |
| Cranberry Raspberry Flavour Low Calorie Jelly Crystals | Unhealthy | Unhealthy |
| Aniseed Jellies | Unhealthy | Unhealthy |
| Peach in Mango Flavoured Jelly | Unhealthy | Unhealthy |
| Orange Flavoured Jelly with Aussie Diced Peaches | Unhealthy | Unhealthy |
| Fruit Mix Jelly Beans | Unhealthy | Unhealthy |
| Lime Flavoured Jelly Crystals | Unhealthy | Unhealthy |
| Soft Jubes | Unhealthy | Unhealthy |
| 3D Gummy Animals | Unhealthy | Unhealthy |
| Sour Worms | Unhealthy | Unhealthy |
| Orange Flavoured Jelly | Unhealthy | Unhealthy |
| Berry Blast Gummies | Unhealthy | Unhealthy |
| Green Power Gummies | Unhealthy | Unhealthy |
| Lime Flavour Jelly Crystals | Unhealthy | Unhealthy |
| Strawberry Flavour Jelly Crystals | Unhealthy | Unhealthy |
| Jelly crystals, sugar sweetened, all flavours | Unhealthy | Unhealthy |
| Jelly crystals, intense sweetened, all flavours | Unhealthy | Unhealthy |
| Lolly, hard varieties | Unhealthy | Unhealthy |
| Lolly, jelly varieties | Unhealthy | Unhealthy |
| Lolly, jelly varieties, natural colours & flavours | Unhealthy | Unhealthy |
| Lolly, jelly varieties, with cocoa | Unhealthy | Unhealthy |
| Orange Flavoured Jelly with Aussie Diced Peaches | Unhealthy | Unhealthy |
| Fruit Mix Jelly Beans | Unhealthy | Unhealthy |
| **Convenience foods** | | |
| **Burgers** | | |
| Breakfast Muffin | Unhealthy | Unhealthy |
| Egg & Bacon Muffin | Unhealthy | Unhealthy |
| Classic Hot Dog | Unhealthy | Unhealthy |
| Cheese Burger | Unhealthy | Unhealthy |
| Chicken burger, white roll, chicken breast, with bacon, cheese, egg & sauce, fast food chain-style | Unhealthy | Unhealthy |
| Chicken burger, white roll, chicken breast, with bacon, cheese, lettuce & mayonnaise, fast food chain | Unhealthy | Unhealthy |
| Chicken burger, white roll, chicken breast, with cheese, lettuce & mayonnaise, fast food chain | Unhealthy | Unhealthy |
| Chicken burger, white roll, chicken breast, with lettuce, tomato & mayonnaise, fast food chain | Unhealthy | Unhealthy |
| Chicken burger, white roll, double chicken breast, with cheese, lettuce, chilli sauce & mayonnaise, fast food chain | Unhealthy | Unhealthy |
| Chicken burger, white roll, crumbed chicken breast, with bacon, cheese, lettuce, mayonnaise & BBQ sauce, fast food chain | Unhealthy | Unhealthy |
| Chicken burger, white roll, crumbed chicken breast, with bacon, cheese, lettuce, pineapple & mayonnaise, fast food | Unhealthy | Unhealthy |
| Chicken burger, white roll, crumbed chicken breast, with bacon, cheese, lettuce, tomato & mayonnaise, fast food chain | Unhealthy | Unhealthy |
| Chicken burger, white roll, crumbed chicken breast, with cheese & mayonnaise, fast food chain | Unhealthy | Unhealthy |
| Chicken burger, white roll, crumbed chicken breast, with cheese, lettuce, hash brown, mayonnaise & sauce, fast food | Unhealthy | Unhealthy |
| Chicken burger, white roll, crumbed chicken breast, with cheese, lettuce, onion, tomato, relish & mayonnaise, fast food chain | Unhealthy | Unhealthy |
| Chicken burger, white roll, crumbed chicken breast, with lettuce & mayonnaise, fast food chain | Unhealthy | Unhealthy |
| Chicken roll, white roll, chicken, with mayonnaise, fast food chain | Unhealthy | Unhealthy |
| Fish burger, with cheese, fast food chain | Unhealthy | Unhealthy |
| Hamburger, white roll, beef patty, with bacon, beetroot, cheese, egg, lettuce, onion & tomato & tomato sauce, fast food chain | Unhealthy | Unhealthy |
| Hamburger, white roll, beef patty, with bacon, cheese, lettuce, onion rings, tomato, pickles, mayonnaise & sauce, fast food chain | Unhealthy | Unhealthy |
| Hamburger, white roll, beef patty, with beetroot, cheese, lettuce, onion, tomato & tomato sauce, fast food chain | Unhealthy | Unhealthy |
| Hamburger, white roll, beef patty, with cheese, lettuce, onion, pickles, tomato, mayonnaise & tomato sauce, fast food chain | Unhealthy | Unhealthy |
| Hamburger, white roll, beef patty, with cheese, lettuce, sauce, fast food chain | Unhealthy | Unhealthy |
| Hamburger, white roll, beef patty, with lettuce, onion, pickles & tomato, mayonnaise & sauce, fast food chain | Unhealthy | Unhealthy |
| Hamburger, white roll, beef patty, with pickles, tomato sauce & mustard, fast food chain | Unhealthy | Unhealthy |
| Hamburger, white roll, 2 beef patties, with lettuce, onion, pickles, tomato, mayonnaise & sauce, fast food chain | Unhealthy | Unhealthy |
| Muffin, English style, with bacon, cheese & egg, fast food chain | Unhealthy | Unhealthy |
| Muffin, English style, with bacon & beef sausage patty, fast food chain | Unhealthy | Unhealthy |
| Muffin, English style, with beef sausage patty, cheese & egg, fast food chain | Unhealthy | Unhealthy |
| Vegetable burger, bread roll, vegetable patty, with cheese, lettuce, onion, sauce & mayonnaise, fast food chain | Unhealthy | Unhealthy |
| Hamburger, white roll, beef patty, with bacon, cheese, onion, pickles & sauce, fast food chain | Unhealthy | Unhealthy |
| Hamburger, white roll, beef patty, with cheese, onion, pickles & sauce, fast food chain | Unhealthy | Unhealthy |
| Hamburger, white roll, 2 beef patties, with bacon, cheese, lettuce, onion, pickles, tomato, mayonnaise & sauce, fast food chain | Unhealthy | Unhealthy |
| Hamburger, white roll, 2 beef patties, with bacon, cheese, lettuce, tomato & mayonnaise, fast food style | Unhealthy | Unhealthy |
| Hamburger, white roll, 2 beef patties, with cheese, onion, pickles & sauce, fast food chain | Unhealthy | Unhealthy |
| Hamburger, white roll, beef patty, with cheese, sauce, fast food chain | Unhealthy | Unhealthy |
| **Canapes** | | |
| Spicy Bean Nacho Balls | Unhealthy | Unhealthy |
| Chickpea Parcels | Unhealthy | Unhealthy |
| Hand Finished Angus Beef & Brown Ale Mini Pies | Unhealthy | Unhealthy |
| Tomato, Feta & Olive Cocktail Quiches | Unhealthy | Unhealthy |
| Beef Cocktail Pies | Unhealthy | Unhealthy |
| Seafood Dumplings | Unhealthy | Unhealthy |
| Pork Dim Sim | Unhealthy | Unhealthy |
| Prawn Dumplings | Unhealthy | Unhealthy |
| Green Curry Rice Balls | Unhealthy | Unhealthy |
| Lean Beef & Sesame Siu Mei | Unhealthy | Unhealthy |
| BBQ Pork & Plum Spring Rolls | Unhealthy | Unhealthy |
| Cabbage & Pork Dumplings | Unhealthy | Unhealthy |
| Petite Quiche Lorraine | Unhealthy | Unhealthy |
| Wild Mushroom Arancini | Healthy | Healthy |
| Shanghai Chives & Pork Dumplings | Unhealthy | Unhealthy |
| Chicken & Mushroom Bun | Unhealthy | Unhealthy |
| Sweet Chilli Chicken Fingers | Unhealthy | Unhealthy |
| Seafood Net Spring Rolls | Unhealthy | Unhealthy |
| Italian Style Tomato Mini Bruschetta | Unhealthy | Unhealthy |
| Garlic Chicken Mini Kiev Balls | Unhealthy | Unhealthy |
| Pork & Water Chestnut Dim Sims | Unhealthy | Unhealthy |
| Gourmet Cocktail Sausage Rolls | Unhealthy | Unhealthy |
| Prawn & Sweetcorn Bites | Unhealthy | Unhealthy |
| Cocktail Hot Dogs | Unhealthy | Unhealthy |
| Jalapeno Cheese & Corn Bites | Unhealthy | Unhealthy |
| Mac 'N Cheese | Unhealthy | Unhealthy |
| Hong Kong Style Pork Dumplings | Unhealthy | Healthy |
| Chicken Mini Skewer with Zesty Citrus & Herbs | Unhealthy | Unhealthy |
| Japanese Style Prawn Gyoza | Healthy | Healthy |
| Soup Dumplings | Unhealthy | Unhealthy |
| Sesame Prawn Toast | Unhealthy | Unhealthy |
| Mac & Cheese Croquettes | Unhealthy | Healthy |
| Free Range Pork & Shallot Gyozo | Healthy | Healthy |
| Wagyu Beef & Chilli Gyoza | Healthy | Healthy |
| Golden Cheese Rings | Unhealthy | Unhealthy |
| Prawn Wonton | Unhealthy | Unhealthy |
| Mozzarella Bites with a Napoli Sauce Filling | Unhealthy | Unhealthy |
| Prawn Hargow | Unhealthy | Unhealthy |
| Gluten Free Pork Dumplings | Unhealthy | Unhealthy |
| Classic Beef Party Pies | Unhealthy | Unhealthy |
| Traditional Party Pasties | Unhealthy | Unhealthy |
| Prawn & Chive Wonton | Unhealthy | Unhealthy |
| Diana Chan's Korean Beef Dumplings | Unhealthy | Unhealthy |
| Diana Chan's Thai Chicken Dumplings | Unhealthy | Unhealthy |
| BBQ Pork Bao | Unhealthy | Unhealthy |
| Quiches Lorraine | Unhealthy | Unhealthy |
| Prawn Dumplings | Unhealthy | Unhealthy |
| Flower Roll | Unhealthy | Unhealthy |
| Spring Rolls | Unhealthy | Unhealthy |
| BBQ Pork Bun | Unhealthy | Unhealthy |
| Prawn Shao Mai | Unhealthy | Unhealthy |
| Free Range Chicken & Shiitake Gyoza | Healthy | Healthy |
| Garlic Chicken Bites | Unhealthy | Unhealthy |
| Seaweed Spring Roll | Unhealthy | Unhealthy |
| Chorizo & Potato Croquette | Unhealthy | Unhealthy |
| Kibbi | Unhealthy | Unhealthy |
| Diana Chan's Vegetable Dumplings | Unhealthy | Unhealthy |
| Fetta Triangles | Unhealthy | Unhealthy |
| Baby Arancini Mushroom | Unhealthy | Unhealthy |
| Extra Tasty Cheese & Crackers | Unhealthy | Unhealthy |
| Vegetable Gyozas | Unhealthy | Unhealthy |
| Beef & Red Wine Tarts | Unhealthy | Unhealthy |
| Siu Mai | Unhealthy | Unhealthy |
| Vegetable Italian Croquettes | Unhealthy | Unhealthy |
| Australian Beef Caramelised Onion & Parsley Party Rolls | Unhealthy | Unhealthy |
| Mac & Cheese Balls | Unhealthy | Healthy |
| Prawn Hargow Dumpling | Unhealthy | Unhealthy |
| Australian Cauliflower in Creamy Camembert Sauce Party Pies | Unhealthy | Unhealthy |
| Pani Puri | Unhealthy | Unhealthy |
| Pork Shao Mai | Unhealthy | Unhealthy |
| Hoisin Pork Spring Rolls | Unhealthy | Unhealthy |
| Beef Koftas | Unhealthy | Unhealthy |
| Chicken Kiev Poppers | Unhealthy | Unhealthy |
| Cocktail Spring Rolls | Unhealthy | Unhealthy |
| Pork & Prawn Dumplings | Unhealthy | Unhealthy |
| Pork & Spring Onion Dumplings | Unhealthy | Unhealthy |
| Steamed BBQ Pork Buns | Unhealthy | Unhealthy |
| Cheeseburger Flavoured Spring Rolls | Unhealthy | Unhealthy |
| Crackling Seekh Kebab Veggie Fingers | Unhealthy | Unhealthy |
| Mild Salami & Cheese Roll-Up | Unhealthy | Unhealthy |
| Dim sim, vegetable &/or meat filling, takeaway style, deep fried | Unhealthy | Unhealthy |
| Dumpling or wonton, savoury, meat & vegetable filled, takeaway style, cooked | Unhealthy | Healthy |
| Samosa, vegetable, deep fried | Unhealthy | Healthy |
| Spring roll, meat &/or vegetable, purchased frozen, baked | Unhealthy | Unhealthy |
| Spring roll, meat &/or vegetable, purchased frozen, deep fried | Unhealthy | Unhealthy |
| Pakora (coated & deep fried vegetables), Indian restaurant-style | Unhealthy | Unhealthy |
| **Meal kits** | | |
| Makhani Meal Kit | Unhealthy | Unhealthy |
| Biryani Meal Kit | Unhealthy | Unhealthy |
| Tomato, Onion and Herbs 3 Min Pasta Meal | Unhealthy | Unhealthy |
| Korean Bibibap Meal Kit | Unhealthy | Unhealthy |
| Chicken Pad Thai & Rice Noodles | Unhealthy | Unhealthy |
| Pesto 3 Min Pasta Meal | Unhealthy | Unhealthy |
| One-Pan Brekky with Beef Chipolata Sausages | Healthy | Healthy |
| Lucknowi Rogan Josh | Unhealthy | Unhealthy |
| Chicken Thai Red Curry Meal for One | Unhealthy | Unhealthy |
| Chinese Chicken Chow Mein Meal for One | Unhealthy | Unhealthy |
| Chicken Pad Thai Meal for One | Unhealthy | Unhealthy |
| Malaysian Peanut Satay | Unhealthy | Unhealthy |
| Korean Bulgogi Beef | Unhealthy | Unhealthy |
| Korma Curry Cooking Kit | Unhealthy | Unhealthy |
| Tarka Dahl Meal Kit | Healthy | Healthy |
| Jamie's Favourite Slaw | Healthy | Healthy |
| Caesar Wrap Kit | Unhealthy | Unhealthy |
| Dill & Pickle Tuna Bruschetta with Rice Crackers | Unhealthy | Unhealthy |
| Yellow Thai Curry | Unhealthy | Unhealthy |
| Lemon Pepper Tuna Bruschetta with Rice Crackers | Unhealthy | Unhealthy |
| All Natural Laksa Soup Kit | Unhealthy | Unhealthy |
| Pad Thai | Unhealthy | Unhealthy |
| Potato & Chorizo | Healthy | Healthy |
| Cacao Superfood Breakfast | Unhealthy | Unhealthy |
| Minestrone Soup Kit | Unhealthy | Unhealthy |
| Classic Tarka Daal | Unhealthy | Unhealthy |
| Lebanese Kofta Meal Kit with Falafel | Unhealthy | Unhealthy |
| Vietnamese Lemongrass & Ginger Chicken Meal Kit | Unhealthy | Unhealthy |
| Taco Kit with Bold Nacho Cheese Flavoured Taco Shells | Unhealthy | Unhealthy |
| Smokey Chipotle Taco Kit | Unhealthy | Unhealthy |
| Chicken and Chorizo Paella with Olive Oil | Unhealthy | Unhealthy |
| Mexican Bean Soup | Unhealthy | Unhealthy |
| Ranch Slaw Kit | Unhealthy | Healthy |
| Kaleslaw Veggie Pot | Unhealthy | Unhealthy |
| Pulled Pork Burrito | Unhealthy | Unhealthy |
| Chicken Noodle Soup | Unhealthy | Unhealthy |
| Macaroni & Cheese Pasta Dinner | Unhealthy | Unhealthy |
| Supreme Macaroni & Cheese | Unhealthy | Unhealthy |
| Chicken with Green Peppercorn & Pearl Barley Recipe Bag | Unhealthy | Healthy |
| Mongolian Beef Noodle Bowls Recipe Bag | Unhealthy | Unhealthy |
| Cheesy Chicken Flavour Pasta & Sauce | Unhealthy | Unhealthy |
| Aromatic Coriander Stir-Fry | Healthy | Healthy |
| Satay Chicken Noodle Kit | Unhealthy | Unhealthy |
| Pad Thai Noodle Kit | Unhealthy | Unhealthy |
| Mee Goreng Noodle Kit | Unhealthy | Unhealthy |
| Chow Mein Noodle Kit | Unhealthy | Unhealthy |
| Korma Curry Kit | Unhealthy | Unhealthy |
| Original Roasted Capsicum & Paprika Fajita Kit | Unhealthy | Unhealthy |
| Sweet Paprika & Tomato Soft Taco Kit | Unhealthy | Unhealthy |
| Smoke Flavour Tuna Pate with Crackers | Unhealthy | Unhealthy |
| Gluten Free Soft Taco Kit | Unhealthy | Unhealthy |
| Delhi Black Makhani Daal | Healthy | Healthy |
| Original Chili con Carne Burrito Kit | Unhealthy | Unhealthy |
| Fiery Goan Curry | Unhealthy | Unhealthy |
| Garlic Aioli Wrap Kit | Unhealthy | Unhealthy |
| Korean Style Duck Bao Buns Share Kit | Unhealthy | Unhealthy |
| Delicate Korma Curry | Unhealthy | Unhealthy |
| Protein Pack | Unhealthy | Unhealthy |
| Royal Festival Briyani Rice Kit | Healthy | Healthy |
| Ham & Egg Protein Bistro Box | Unhealthy | Unhealthy |
| Falafel Protein Bistro Box | Unhealthy | Healthy |
| Chicken Protein Bistro Box | Unhealthy | Unhealthy |
| Original Macaroni with Cheese Sauce | Unhealthy | Unhealthy |
| Vietnamese Rice Paper Rolls Kit with Nuoc Cham | Unhealthy | Unhealthy |
| Vietnamese Rice Paper Rolls Kit with Hoisin Sauce | Unhealthy | Unhealthy |
| Lemon Chicken Complete Bake at Home Meal Kit | Unhealthy | Unhealthy |
| Japanese Ramen Kit | Unhealthy | Healthy |
| Broccolini Roasting Kit | Unhealthy | Unhealthy |
| Vietnamese Style Duck Rice Paper Rolls Share Kit | Unhealthy | Unhealthy |
| Honey Soy Stir Fry Kit | Unhealthy | Unhealthy |
| Bombay Style Cauliflower Roasting Kit | Unhealthy | Unhealthy |
| Brussels Sprouts Roasting Kit | Unhealthy | Unhealthy |
| Asian Style Soup Kit | Unhealthy | Unhealthy |
| Macaroni with Smoky Bacon Flavour Cheese Sauce | Unhealthy | Unhealthy |
| Smokin' Bacon Flavoured Cheesy Mac | Unhealthy | Unhealthy |
| Thai Massaman Curry | Unhealthy | Healthy |
| Thai Red Curry | Unhealthy | Healthy |
| The Oven Baked Cheesy Tomato Enchilada Kit | Unhealthy | Unhealthy |
| Tasty Aged Cheddar Slices and Wholemeal Crackers with Caramelised Onion Relish | Unhealthy | Unhealthy |
| Kaleslaw Salad Kit | Unhealthy | Healthy |
| **Pizza** | | |
| Cheese & Bacon Pockets | Unhealthy | Unhealthy |
| BBQ Angus Pockets | Unhealthy | Unhealthy |
| Hot Dog Pockets | Unhealthy | Unhealthy |
| Hand Crafted Prosciutto and Goats Cheese Pizza with Fresh Rocket to Garnish | Unhealthy | Unhealthy |
| Mexican Chicken Special Edition Pizza | Unhealthy | Unhealthy |
| Stone Baked Spicy Italian Fresh Gourmet Pizza | Unhealthy | Unhealthy |
| Roasted Mediterranean Wood Fired Pizza | Unhealthy | Unhealthy |
| Pepperoni Pizza | Unhealthy | Unhealthy |
| Pizza Mozzarella | Unhealthy | Unhealthy |
| BBQ Chicken & Caramelised Onion Flavour Pizza | Unhealthy | Unhealthy |
| Meat Lovers Mini Pizzas | Unhealthy | Unhealthy |
| Hawaiian Mini Pizzas | Unhealthy | Unhealthy |
| Authentic Italian Stone Baked Pizza Supreme | Unhealthy | Unhealthy |
| Angus Steak & Bacon Pizza | Unhealthy | Unhealthy |
| BBQ Chicken & Bacon Pizza | Unhealthy | Unhealthy |
| Pizza Prosciutto | Unhealthy | Unhealthy |
| Supreme Pizza | Unhealthy | Unhealthy |
| Fresh Margherita Pizza | Unhealthy | Unhealthy |
| Fresh Super Supreme Pizza | Unhealthy | Unhealthy |
| Fresh BBQ Meat Deluxe Pizza | Unhealthy | Unhealthy |
| Fresh BBQ Chicken & Bacon Pizza | Unhealthy | Unhealthy |
| Ultimate Tomato & Mozzarella Pizza | Unhealthy | Healthy |
| Hot Dog Pizza | Unhealthy | Unhealthy |
| Pepperoni Stonebaked Hand Topped Pizza | Unhealthy | Unhealthy |
| Woodfired Spinach & Goat's Cheese Pizza | Unhealthy | Unhealthy |
| Spanish Chorizo Special Edition Pizza | Unhealthy | Unhealthy |
| Four Cheese Stonebaked Pizza | Unhealthy | Unhealthy |
| Mozzarella & Pesto Pizza | Unhealthy | Unhealthy |
| Five Cheese Pizza | Unhealthy | Unhealthy |
| Spinach & Mascarpone Pizza | Unhealthy | Unhealthy |
| Mozzarella & Mushroom Pizza | Unhealthy | Unhealthy |
| Super Thin Stone Baked Bolognese Pizza | Unhealthy | Unhealthy |
| Cheese Burger Pizza | Unhealthy | Unhealthy |
| Four Cheese Pizza | Unhealthy | Unhealthy |
| Margherita Pizza | Unhealthy | Unhealthy |
| Spinach & Mozzarella Pizza | Unhealthy | Unhealthy |
| Fresh Premium Pepperoni Pizza | Unhealthy | Unhealthy |
| Ham & Cheese Pizza | Unhealthy | Unhealthy |
| Double Cheese Pizza | Unhealthy | Unhealthy |
| Ultimate Meatlovers Stone Baked Pizza | Unhealthy | Unhealthy |
| Gourmet Veg Thin-Base Pizza | Unhealthy | Unhealthy |
| Prosciutto Thin Base Pizza | Unhealthy | Unhealthy |
| Pizza Bianca Carne e Patata | Unhealthy | Unhealthy |
| BBQ Pulled Pork Pizza | Unhealthy | Unhealthy |
| BBQ Meat Lovers Pizza Slices | Unhealthy | Unhealthy |
| Gourmet Pizza | Unhealthy | Unhealthy |
| Ultimate BBQ Chicken & Bacon Pizza | Unhealthy | Unhealthy |
| Gourmet Pizza with Leg Ham, Pineapple, Shallots & Mozzarella Cheese | Unhealthy | Unhealthy |
| The Melbourne Pizza | Unhealthy | Unhealthy |
| Mega Meaty Pizza | Unhealthy | Unhealthy |
| Chicken, Portobello Mushrooms & Fetta Stone Baked Pizza | Unhealthy | Unhealthy |
| Hawaiian Calzone | Unhealthy | Unhealthy |
| Gluten-Free Pizza Mozzarella | Unhealthy | Unhealthy |
| Cheese Pizza | Unhealthy | Unhealthy |
| BBQ Chicken & Pineapple Family Pizza | Unhealthy | Unhealthy |
| Pizza with Pepperoni, Red Onion and Capsicum Relish | Unhealthy | Unhealthy |
| BBQ Meatlovers Pizza | Unhealthy | Unhealthy |
| Hawaiian Pizza | Unhealthy | Unhealthy |
| Pepperoni Pizza | Unhealthy | Unhealthy |
| Supreme Pizza | Unhealthy | Unhealthy |
| Pepperoni, Kalamata Olives, Shallots & Mozarella Cheese Pizza | Unhealthy | Unhealthy |
| Happy Hawaiian | Unhealthy | Unhealthy |
| Stone Baked Fresh Hawaiian Pizza | Unhealthy | Unhealthy |
| Happy Pig Pizza | Unhealthy | Unhealthy |
| Chicken & Pancetta Pizza | Unhealthy | Unhealthy |
| Prosciutto & Provolone Pizza | Unhealthy | Unhealthy |
| Quattro Formaggi Pizza | Unhealthy | Unhealthy |
| Mini Pizza Scrolls | Unhealthy | Unhealthy |
| Meatball Pizza Subs | Unhealthy | Unhealthy |
| Tomato & Cheese Pizza Subs | Unhealthy | Unhealthy |
| The Milano Pizza | Unhealthy | Unhealthy |
| Italian Meatball Pizza | Unhealthy | Unhealthy |
| Salami & Basil Pesto Pizza | Unhealthy | Unhealthy |
| Extra Cheese Pepperoni Pizza | Unhealthy | Unhealthy |
| Family Favourite Hawaiian Pizza | Unhealthy | Unhealthy |
| Chicken Parma Pizza | Unhealthy | Unhealthy |
| Garlic Mushroom & Truffle Oil Pizza | Unhealthy | Unhealthy |
| Italian Meats & Kalamata Olive Pizza | Unhealthy | Unhealthy |
| Stuffed Crust Loaded Pepperoni Big Pizza | Unhealthy | Unhealthy |
| Rosemary Pumpkin & Feta Pizza | Unhealthy | Unhealthy |
| Woodfired Beetroot & Goat's Cheese Pizza | Unhealthy | Unhealthy |
| Chicken & Sweet Potato Pizza | Unhealthy | Unhealthy |
| Hawaiian Pizza | Unhealthy | Unhealthy |
| Green Pizza | Unhealthy | Unhealthy |
| Pizza, bacon & egg, thick base, BBQ sauce, fast food chain | Unhealthy | Unhealthy |
| Pizza, bacon & egg, thin base, BBQ sauce, fast food chain | Unhealthy | Unhealthy |
| Pizza, cheese & pesto, purchased frozen, uncooked | Unhealthy | Unhealthy |
| Pizza, chicken & bacon, BBQ sauce, purchased frozen, baked | Unhealthy | Unhealthy |
| Pizza, chicken & bacon, thick base, BBQ sauce, fast food chain | Unhealthy | Unhealthy |
| Pizza, chicken & bacon, thin base, BBQ sauce, fast food chain | Unhealthy | Unhealthy |
| Pizza, ham & cheese, frozen, baked | Unhealthy | Unhealthy |
| Pizza, ham & pineapple, purchased frozen, baked | Unhealthy | Unhealthy |
| Pizza, ham & pineapple, thick base, fast food chain | Unhealthy | Unhealthy |
| Pizza, ham & pineapple, thin base, fast food chain | Unhealthy | Unhealthy |
| Pizza, meat lovers, BBQ sauce, purchased frozen, baked | Unhealthy | Unhealthy |
| Pizza, meat lovers, thick base, BBQ sauce, fast food chain | Unhealthy | Unhealthy |
| Pizza, pepperoni, thick base, fast food chain | Unhealthy | Unhealthy |
| Pizza, seafood, thin base, purchased frozen, baked | Unhealthy | Unhealthy |
| Pizza, seafood, thick base, fast food chain | Unhealthy | Unhealthy |
| Pizza, seafood, thin base, fast food chain | Unhealthy | Unhealthy |
| Pizza, supreme, thick base, fast food chain | Unhealthy | Unhealthy |
| Pizza, supreme, thin base, fast food chain | Unhealthy | Unhealthy |
| Pizza, vegetable, purchased frozen, baked | Unhealthy | Unhealthy |
| Pizza, vegetable, thick base, fast food chain | Unhealthy | Unhealthy |
| Pizza, vegetable, thin base, fast food chain | Unhealthy | Unhealthy |
| Pizza, supreme, purchased frozen, baked | Unhealthy | Unhealthy |
| Pizza, cheese & tomato, purchased frozen, baked | Unhealthy | Unhealthy |
| Pizza, cheese & tomato, thick base, fast food chain | Unhealthy | Unhealthy |
| Pizza, cheese & tomato, thin base, fast food chain | Unhealthy | Unhealthy |
| Pizza, pepperoni, purchased frozen, baked | Unhealthy | Unhealthy |
| Pizza, pepperoni, thin base, fast food chain | Unhealthy | Unhealthy |
| Pizza, meat lovers, thin base, fast food chain | Unhealthy | Unhealthy |
| Pizza, ham & cheese, thin base, fast food chain | Unhealthy | Unhealthy |
| Pizza, chicken & vegetable, thin base, fast food chain | Unhealthy | Unhealthy |
| Pizza, supreme, stuffed crust, fast food chain | Unhealthy | Unhealthy |
| **Pre-prepared salads and sandwiches** | | |
| Beetroot Spinach Feta Salad | Unhealthy | Unhealthy |
| Roast Tomato Cous Cous Salad | Unhealthy | Unhealthy |
| Chicken Basil Penne Salad | Unhealthy | Healthy |
| Ham & Cheese Toasties | Unhealthy | Unhealthy |
| BBQ Beef Toasties | Unhealthy | Unhealthy |
| Cheese & Tomato Toasties | Unhealthy | Unhealthy |
| Middle Eastern Style Cous Cous Premium Prepared Salad | Unhealthy | Unhealthy |
| Tabouleh Premium Prepared Salad | Healthy | Healthy |
| Chicken Tikka Sub | Unhealthy | Unhealthy |
| Chicken Parma Sub | Unhealthy | Unhealthy |
| Chicken Fajitas | Unhealthy | Unhealthy |
| Roast Duck Hoisin Wrap | Unhealthy | Unhealthy |
| Pulled Pork Char Sui Wrap | Unhealthy | Unhealthy |
| French Tuna Salad | Unhealthy | Unhealthy |
| Haloumi & Mushroom Frittata Salad | Healthy | Healthy |
| Vietnamese Chicken Salad | Unhealthy | Healthy |
| Teriyaki Chicken Salad | Unhealthy | Healthy |
| Chicken Burrito | Healthy | Healthy |
| Lentil Salad | Unhealthy | Healthy |
| Red Kidney Bean Salad | Healthy | Healthy |
| Yellowfin Tuna & Rice Italian Style Salad Meal | Unhealthy | Unhealthy |
| Roast Chicken Roll | Unhealthy | Unhealthy |
| Roast Angus Beef Roll | Unhealthy | Unhealthy |
| California Tuna Salad | Healthy | Healthy |
| Chicken Parma Sandwich | Unhealthy | Unhealthy |
| Chick Pea Salad | Healthy | Healthy |
| Kale Caesar Salad Kit | Unhealthy | Unhealthy |
| Sweet Capsicum Salad | Healthy | Healthy |
| Greek Salad | Healthy | Healthy |
| Crunchy Rainbow Salad | Healthy | Healthy |
| Chicken Basil Pesto Pasta Salad | Unhealthy | Unhealthy |
| Peri Peri Chicken Wrap | Unhealthy | Unhealthy |
| Egg, Bacon & Cheese All Day Brekky Burrito | Unhealthy | Unhealthy |
| Ancient Nutty Grain | Healthy | Healthy |
| Mushroom Frittata Garden Salad | Healthy | Healthy |
| Roasted Vegetable Warm Salad | Unhealthy | Unhealthy |
| Pumpkin, Brown Rice, and Feta Salad | Unhealthy | Unhealthy |
| Beetroot & Roasted Butternut Pumpkin Salad with Balsamic Dressing | Unhealthy | Unhealthy |
| Greek-Style Salad Bowl | Unhealthy | Unhealthy |
| Tropical Salad Kit | Unhealthy | Unhealthy |
| Pumpkin & Feta Flatbread | Unhealthy | Unhealthy |
| Lamb & Tzatziki Flatbread | Unhealthy | Unhealthy |
| Pesto Broccoli Rice Pot | Unhealthy | Unhealthy |
| Butter Chicken Naanwich | Unhealthy | Healthy |
| Classic Egg Mayo | Unhealthy | Unhealthy |
| Indian Chicken Rice Salad | Unhealthy | Unhealthy |
| Italian Penne Pasta Salad with Sundried Tomato & Kalamata Olive | Unhealthy | Healthy |
| Potato & Chopped Egg Salad | Unhealthy | Unhealthy |
| Roasted Pumpkin, Red Onion & Feta Pasta Salad with Citrus Dressing | Healthy | Healthy |
| Roast Vegetable Cous Cous Salad with Lemon Vinaigrette | Healthy | Healthy |
| Caesar Salad Kit | Unhealthy | Unhealthy |
| Mix Hawaiian Poke Bowl | Unhealthy | Unhealthy |
| Hawaiian Poke Bowl with Salmon | Unhealthy | Unhealthy |
| Crunchy Tomato & Cheese Panini | Unhealthy | Unhealthy |
| Moroccan Spiced Vegetable Salad | Healthy | Healthy |
| Creamy Pasta Salad | Unhealthy | Unhealthy |
| Tandoori Chicken Flatbread Wrap | Unhealthy | Healthy |
| Caesar Chicken Wrap | Unhealthy | Unhealthy |
| Tuna Salad Sandwich | Unhealthy | Unhealthy |
| Chicken Schnitzel Sandwich | Unhealthy | Unhealthy |
| Pumpkin & Feta Spinach Salad | Healthy | Healthy |
| Chicken & Quinoa Salad with a Hint of Coconut | Unhealthy | Unhealthy |
| Chicken & Kale Slaw in a Turmeric Dressing | Unhealthy | Unhealthy |
| Teriyaki Chicken Salad | Unhealthy | Unhealthy |
| Ancient Grain Salad | Unhealthy | Unhealthy |
| Beef Burrito | Unhealthy | Unhealthy |
| Chicken Parma on White Bread | Unhealthy | Unhealthy |
| Butter Chicken Flatbread | Unhealthy | Unhealthy |
| Triple Chicken & Bacon Caesar on a White Tortilla | Unhealthy | Unhealthy |
| Classic Tuna Pasta Salad | Unhealthy | Unhealthy |
| Quinoa, Baby Broccoli & Fetta Salad | Healthy | Healthy |
| Chicken Cobb Salad | Unhealthy | Unhealthy |
| Hot Smoked Salmon Poke Bowl | Unhealthy | Healthy |
| Bangin' Brekky Wrap | Unhealthy | Unhealthy |
| Roast Chicken & Kale Slaw Salad | Unhealthy | Unhealthy |
| Mexican Beef & Taco Salad | Unhealthy | Unhealthy |
| Chicken Couscous Salad with Chickpeas and Harissa & Mint Dressing | Unhealthy | Unhealthy |
| Quinoa Tabbouleh | Unhealthy | Unhealthy |
| Bean Medley Salad | Unhealthy | Unhealthy |
| Black Rice, Beetroot & Walnut Salad | Unhealthy | Unhealthy |
| Sashimi Salmon Salad | Unhealthy | Unhealthy |
| Classic Triple Chicken Mayo, Egg Mayo and Ham, Cheese & Tomato on White Bread | Unhealthy | Unhealthy |
| Superfood Salad Bowl | Unhealthy | Unhealthy |
| Smashed Avo & Egg | Healthy | Healthy |
| Mexican Style Bean Salsa with Egg | Unhealthy | Unhealthy |
| Lentil Salad | Unhealthy | Unhealthy |
| Miso Chicken & Chickpea Salad | Unhealthy | Unhealthy |
| Thai Beef Salad | Unhealthy | Unhealthy |
| Teriyaki Chicken Flavoured Noodle Salad Bowl | Unhealthy | Healthy |
| Ham, Cheese & Tomato Wrap | Unhealthy | Unhealthy |
| Super Salad Wrap | Unhealthy | Unhealthy |
| Peri Peri Chicken Wrap | Unhealthy | Unhealthy |
| Pesto Chicken, Parmesan & Roasted Capsicum Wrap | Unhealthy | Unhealthy |
| Falafel Hommus Wrap | Unhealthy | Unhealthy |
| Egg & Creamy Mayo Bagel with Rocket | Unhealthy | Unhealthy |
| Roast Beef & Onion Jam Premium Sandwich | Unhealthy | Unhealthy |
| Turkey & Swiss Cheese Premium Sandwich | Unhealthy | Unhealthy |
| Chicken Pesto Premium Sandwich | Unhealthy | Unhealthy |
| Chicken & Bacon Premium Sandwich | Unhealthy | Unhealthy |
| Tasty Cheese & Tomato Chutney Sandwich | Unhealthy | Unhealthy |
| Ham & Egg Sandwich | Unhealthy | Unhealthy |
| Egg Sandwich | Unhealthy | Unhealthy |
| Pesto Egg Mayo & Tasty Cheese Sandwich | Unhealthy | Unhealthy |
| Kale & Quinoa Salad with Tuna | Unhealthy | Unhealthy |
| Asian Style Salad Bowl | Unhealthy | Healthy |
| Kaleslaw Kit | Unhealthy | Healthy |
| Mexican Style Salad Bowl | Unhealthy | Healthy |
| Greek Chicken & Couscous Salad with Tzatziki Dressing | Unhealthy | Unhealthy |
| Traditional Greek Style Salad Kit | Unhealthy | Unhealthy |
| Caesar Salad Bowl | Unhealthy | Unhealthy |
| Pesto Chicken & Semi-Dried Tomatoes Sandwich | Unhealthy | Unhealthy |
| Chicken Mayo Sandwich | Unhealthy | Unhealthy |
| Hummus & Tabouli Falafel Wrap | Unhealthy | Unhealthy |
| Thai Green Chicken & Crunchy Slaw Wrap | Unhealthy | Unhealthy |
| Asian Salad Kit | Unhealthy | Unhealthy |
| Coleslaw Salad | Unhealthy | Unhealthy |
| Creamy & Crunchy Salad Kit | Unhealthy | Unhealthy |
| Hot Smoked Salmon with Creamy Potato & Kale Salad | Unhealthy | Unhealthy |
| Free Range Egg & Wild Rocket Sandwich | Unhealthy | Unhealthy |
| Wood Smoked Ham, Cheese & Tomato with Free Range Egg Mayo on White Bread Sandwich | Unhealthy | Unhealthy |
| Bacon & Free Range Egg with Tomato Relish on Light Rye Bread Sandwich | Unhealthy | Unhealthy |
| Pastrami, Emmental Cheese & Pickles with Free Range Egg, Mustard Mayo on Light Rye Bread Sandwich | Unhealthy | Unhealthy |
| Roast Chicken, Fresh Avocado & Rocket with Free Range Egg Mayo on Multigrain Bread | Unhealthy | Unhealthy |
| Chicken, Wood Smoked Bacon & Cheese Sandwich | Unhealthy | Unhealthy |
| Crispy Bacon, Lettuce & Tomato Sandwich | Unhealthy | Unhealthy |
| Hot Smoked Salmon & Rainbow Salad | Unhealthy | Healthy |
| Rainbow Whole Bowl | Unhealthy | Healthy |
| Crunchy Whole Bowl | Unhealthy | Unhealthy |
| Green Whole Bowl | Unhealthy | Healthy |
| Sunshine Whole Bowl | Unhealthy | Unhealthy |
| Chicken Pesto Pasta Salad | Unhealthy | Unhealthy |
| Pesto Pasta Salad | Unhealthy | Unhealthy |
| Beetroot & Dill Salad with Fragrant Spiced Dressing | Healthy | Healthy |
| Potato Salad | Unhealthy | Unhealthy |
| Green Goddess Salad | Unhealthy | Unhealthy |
| Breakfast wrap, white tortilla, bacon, egg , cheese & sauce, fast food chain | Unhealthy | Unhealthy |
| Chicken wrap, white tortilla, coated chicken breast strips, with salad & mayonnaise, fast food chain | Unhealthy | Unhealthy |
| Chicken wrap, white tortilla, chicken breast strips, with salad & mayonnaise, fast food chain | Unhealthy | Unhealthy |
| **Ready meals** | | |
| Curried Prawns | Unhealthy | Unhealthy |
| Thai Red Curry with Beef, Vegetables & Rice | Unhealthy | Unhealthy |
| Tomato & Bacon Penne with Mushrooms in a Mild Chilli Sauce | Unhealthy | Unhealthy |
| Slow Cooked Beef With Rigatoni Pasta, Mushrooms and Capsicum | Unhealthy | Unhealthy |
| Coconut Chicken with Vegetables & Noodles | Unhealthy | Unhealthy |
| Chicken Kiev | Unhealthy | Unhealthy |
| Mongolian Beef with Wholemeal Noodles | Unhealthy | Unhealthy |
| Chilli Dynamite | Unhealthy | Healthy |
| Chilli Wedges | Unhealthy | Healthy |
| Chilli Prawn Linguine | Unhealthy | Unhealthy |
| Green Chicken Curry | Unhealthy | Unhealthy |
| Brown Rice & Buckwheat Tuna & Superfoods | Unhealthy | Unhealthy |
| Cheese Burger with Tomato Relish | Unhealthy | Unhealthy |
| Shepherd's Pie | Unhealthy | Unhealthy |
| Chili Con Carne | Unhealthy | Unhealthy |
| BBQ Mixed Grill | Unhealthy | Unhealthy |
| Spaghetti and Sausages | Unhealthy | Unhealthy |
| Lasagne Bolognese | Unhealthy | Healthy |
| Beef Cannelloni | Unhealthy | Unhealthy |
| Rice Mac & Cheese | Unhealthy | Healthy |
| Indian Tandoori Chicken with Spinach, Chickpeas, Sunflower Seeds & Black Quinoa | Unhealthy | Unhealthy |
| Pork Belly Buns with Shallot & Hoisin | Unhealthy | Healthy |
| BBQ Pulled Pork with Pickled Cabbage & Sweet Potato Mash | Healthy | Healthy |
| Beef Tortellini | Unhealthy | Unhealthy |
| Mild Chicken & Rice Curry | Unhealthy | Healthy |
| Chunky Pepper Steak Pie | Unhealthy | Healthy |
| Chicken Pesto Gnocchi | Unhealthy | Unhealthy |
| Bhindi Masala Okra Curry | Healthy | Healthy |
| Dhal Tadka | Healthy | Healthy |
| Dhal Makhani | Unhealthy | Unhealthy |
| Grilled Chicken & Bacon Creamy Pasta Bake | Unhealthy | Unhealthy |
| Creamy Mushroom Beef Steak with Creamy Mash Potato, Carrots & a Mushroom Sauce | Unhealthy | Unhealthy |
| Chicken, Porcini Mushroom And Truffle Risotto | Unhealthy | Unhealthy |
| Special Fried Rice | Healthy | Healthy |
| Mild Butter Chicken & Basmati Rice | Unhealthy | Healthy |
| Satay Chicken Rice Paper Rolls | Healthy | Healthy |
| Stacked Brekky Omelette | Unhealthy | Unhealthy |
| Chinese Chicken & Cashews with Rice & Vegetables | Unhealthy | Unhealthy |
| Moroccan Spiced Lamb | Unhealthy | Unhealthy |
| Cracked Pepper Rump Steak | Unhealthy | Unhealthy |
| Lentil & Chickpea Dhal with Roasted Vegetables | Unhealthy | Unhealthy |
| Bangers & Sweet Potato Mash | Healthy | Healthy |
| Grass-Fed Beef Bolognese with Roasted Desirée Potatoes | Unhealthy | Unhealthy |
| Creamy Pumpkin Leek & Spinach Risotto | Healthy | Healthy |
| Creamy Penne Boscaiola | Unhealthy | Unhealthy |
| Beef & Black Bean Sauce with Rice | Unhealthy | Unhealthy |
| Singapore Chicken Noodles | Unhealthy | Unhealthy |
| Penang Curry | Unhealthy | Unhealthy |
| Vegetable Korma with White Rice | Unhealthy | Unhealthy |
| Satay Chicken Curry with Long Grain Rice | Unhealthy | Unhealthy |
| Butter Chicken Curry with Long Grain Rice | Unhealthy | Unhealthy |
| Teriyaki Chicken | Unhealthy | Unhealthy |
| Authentic Beef Ravioli | Healthy | Healthy |
| Massaman Chicken with Rice | Unhealthy | Healthy |
| Lean Beef & Veg | Healthy | Healthy |
| Chicken & Leek Risotto | Unhealthy | Unhealthy |
| Roast Pork | Unhealthy | Unhealthy |
| Sweet Potato, Feta, Spring Onion & Caraway Seeds Frittata | Unhealthy | Unhealthy |
| Zucchini, Roasted Leek & Goat's Cheese Frittata | Unhealthy | Unhealthy |
| Classic Spiced Chicken and Bacon Linguine | Unhealthy | Unhealthy |
| Pancetta Macaroni & Cheese | Unhealthy | Healthy |
| Louisiana Beef Brisket with Mash n' Slaw | Unhealthy | Unhealthy |
| All Natural Supergrain Nasi Goreng | Unhealthy | Healthy |
| All Natural Supergrain Butter Chicken | Healthy | Healthy |
| Vegetable & Lentil | Healthy | Healthy |
| Vegetable Dahl Curry | Unhealthy | Healthy |
| Turmeric Omelette with Chipotle Jam | Unhealthy | Healthy |
| Double Crunchy Tempura Prawn Roll | Unhealthy | Unhealthy |
| Tuscan Bake & Sweet Mash | Unhealthy | Unhealthy |
| Chicken Schnitty & Chips | Unhealthy | Unhealthy |
| Spaghetti Bolognese | Healthy | Healthy |
| Gnocchi Napoletana | Healthy | Healthy |
| Chicken Korma with Rice | Unhealthy | Unhealthy |
| Chicken Tikka Masala with Rice | Unhealthy | Unhealthy |
| Lamb Rogan Josh with Rice | Unhealthy | Healthy |
| Zesty Chicken | Unhealthy | Unhealthy |
| Mexican Style Slow Cooked Beef with Beans | Unhealthy | Unhealthy |
| Ajisai Plate | Unhealthy | Unhealthy |
| Yellow Fish Curry | Healthy | Healthy |
| Veggie Mix Summer Roll | Healthy | Healthy |
| Kangaroo Chilli Con Carne with Green Penne | Unhealthy | Healthy |
| Cottage Pie | Unhealthy | Unhealthy |
| Jamaican Jerk Chicken & Black-Eyed Beans | Unhealthy | Healthy |
| Tulip Chicken and Salmon Sushi Combination | Unhealthy | Unhealthy |
| Pesto Chicken with Broccoli & Pasta | Healthy | Healthy |
| Pumpkin Risotto | Healthy | Healthy |
| Butter Chicken with Long Grain Rice | Unhealthy | Unhealthy |
| Chicken Casserole with Spring Vegetables | Unhealthy | Unhealthy |
| Beef Rice Paper Roll | Unhealthy | Unhealthy |
| Egg White Omelette & Potato Smash | Unhealthy | Healthy |
| Turkey Roast & Potato Bake | Unhealthy | Unhealthy |
| Thai Green Curry with Chik'n and Rice | Unhealthy | Unhealthy |
| Teriyaki Chicken Sushi Rolls | Unhealthy | Unhealthy |
| Char Siu BBQ Pork with Carrots, Broccoli & Rice | Unhealthy | Unhealthy |
| Teriyaki Chicken Lunch Box | Unhealthy | Unhealthy |
| Mexican Lasagne | Unhealthy | Unhealthy |
| Tuna Flavoured Roll-Your-Own Sushi | Unhealthy | Unhealthy |
| Black Pepper Beef Noodles | Unhealthy | Unhealthy |
| Salmon Sushi Pack | Unhealthy | Unhealthy |
| Chicken Tikka Masala | Unhealthy | Healthy |
| Madras Style Coconut Beef | Healthy | Healthy |
| Brown Rice & Quinoa with Tuna | Unhealthy | Unhealthy |
| Three Cheese Macaroni | Unhealthy | Unhealthy |
| Penne Napoletana | Unhealthy | Unhealthy |
| Macaroni & 3 Cheeses | Unhealthy | Unhealthy |
| Meat-Free Chilli Con Carne with Rice | Unhealthy | Unhealthy |
| Meat-Free Thai Green Curry with Rice | Unhealthy | Unhealthy |
| Thai Fish Curry with Coconut Rice | Unhealthy | Unhealthy |
| Yuri Plate | Unhealthy | Unhealthy |
| Satay Cashew Free Range Chicken on Rice with Broccoli and Cauliflower | Unhealthy | Healthy |
| Vegetarian Pack | Unhealthy | Unhealthy |
| Creamy Mushroom Buckwheat | Unhealthy | Unhealthy |
| Aburi Salmon Chef Salmon #1 | Unhealthy | Unhealthy |
| Salmon Sushi Combo | Unhealthy | Unhealthy |
| Fettucine with Chicken and Cream Sauce | Healthy | Healthy |
| Butter Chicken | Unhealthy | Unhealthy |
| Sweet Potato Falafel with Moroccan Spiced Chickpeas & Freekeh | Unhealthy | Unhealthy |
| Chicken Tikka Masala & Rice | Unhealthy | Unhealthy |
| Beef Madras | Unhealthy | Healthy |
| Meat-Free Meatballs & Mash in Mushroom Sauce | Unhealthy | Unhealthy |
| Beef Rendang Curry with Turmeric Rice | Unhealthy | Unhealthy |
| Creamy Tuna Bake | Unhealthy | Unhealthy |
| Moroccan Tagine | Unhealthy | Unhealthy |
| Butter Chicken With Turmeric Basmarti | Unhealthy | Unhealthy |
| Mixed dish, cheeseburger, potato fries & soft drink, fast food style | Unhealthy | Unhealthy |
| Frozen meal, Mexican wrap, beef, rice & vegetables, energy controlled portion | Unhealthy | Unhealthy |
| Frozen meal, beef lasagne, energy controlled portion | Unhealthy | Unhealthy |
| Frozen meal, pasta with bolognese sauce | Unhealthy | Unhealthy |
| Lasagne (Lasagna), beef, commercial, purchased fresh or frozen, cooked | Unhealthy | Unhealthy |
| Spaghetti in meat sauce, canned, regular | Unhealthy | Unhealthy |
| Spaghetti in tomato & cheese sauce, canned, regular | Unhealthy | Unhealthy |
| Spaghetti in tomato & cheese sauce, canned, reduced salt | Unhealthy | Unhealthy |
| Frozen meal, tuna mornay, with pasta & white sauce | Unhealthy | Unhealthy |
| Snack pack, savoury, white wheat flour biscuit with tuna | Unhealthy | Unhealthy |
| Frozen meal, prawn & rice | Unhealthy | Unhealthy |
| Frozen meal, beef & vegetables, energy controlled portion | Unhealthy | Unhealthy |
| Frozen meal, roast beef & vegetables | Unhealthy | Unhealthy |
| Sauce, pasta, beef bolognese, commercial | Unhealthy | Healthy |
| Frozen meal, beef & noodles or pasta, energy controlled portion | Unhealthy | Unhealthy |
| Frozen meal, beef & rice, energy controlled portion | Unhealthy | Unhealthy |
| Frozen meal, veal schnitzel with cheese & potato | Unhealthy | Unhealthy |
| Frozen meal, lamb & vegetables, energy controlled portion | Unhealthy | Unhealthy |
| Frozen meal, pork & vegetables with rice | Unhealthy | Unhealthy |
| Frozen meal, sausage & vegetables | Unhealthy | Unhealthy |
| Saveloy, battered, deep fried, oil not further defined | Unhealthy | Unhealthy |
| Frozen meal, chicken & vegetables, energy controlled portion | Unhealthy | Unhealthy |
| Frozen meal, roast chicken with vegetables | Unhealthy | Unhealthy |
| Frozen meal, chicken, vegetables & noodles or pasta, energy controlled portion | Unhealthy | Unhealthy |
| Frozen meal, curry, chicken & rice, coconut milk sauce | Unhealthy | Unhealthy |
| Frozen meal, curry, chicken & rice, Indian style | Unhealthy | Unhealthy |
| Frozen meal, curry, chicken & rice, energy controlled portion | Unhealthy | Unhealthy |
| Frozen meal, vegetables & pasta or noodles, energy controlled portion | Unhealthy | Healthy |
| Curry, commercial, legume (dhal) | Unhealthy | Healthy |
| **Savoury pies and pastries** | | |
| Chicken & Vegetable Pies | Unhealthy | Unhealthy |
| Traditional Meat Pies | Unhealthy | Unhealthy |
| Beef Sausage Roll | Unhealthy | Unhealthy |
| Leek & Bacon Quiche | Unhealthy | Unhealthy |
| Curry Beef Pie | Unhealthy | Unhealthy |
| Spinach & Ricotta Roll | Unhealthy | Unhealthy |
| Beef & Mushroom Pie | Unhealthy | Unhealthy |
| Vegetable Pastie | Unhealthy | Unhealthy |
| Smoked Leg Ham and Tasty Cheese Croissant | Unhealthy | Unhealthy |
| Corn Jacks | Unhealthy | Unhealthy |
| BBQ Pulled Pork | Unhealthy | Unhealthy |
| Egg & Bacon Roll | Unhealthy | Unhealthy |
| Cheese Burger Pies | Unhealthy | Unhealthy |
| Beef Pie | Unhealthy | Unhealthy |
| Cheese & Leek Plaits | Unhealthy | Healthy |
| Beef & Pepper Pie | Unhealthy | Unhealthy |
| Lorraine Quiche | Unhealthy | Unhealthy |
| Garlic, Herb & Lamb Pie | Unhealthy | Unhealthy |
| Ham & Cheese Quiche | Healthy | Healthy |
| Meat & Vegetable Pastie | Unhealthy | Unhealthy |
| Smoky-Style Beef Pie with Pale Ale | Unhealthy | Unhealthy |
| Premium Chunky Beef Pies with Smoky BBQ Sauce | Unhealthy | Unhealthy |
| Premium Chunky Aussie Angus Beef Pie | Unhealthy | Unhealthy |
| Chicken, Mushroom & Leek Pie | Unhealthy | Unhealthy |
| Slow Cooked King Island Beef Pies in Rich Gravy | Unhealthy | Unhealthy |
| Slow-Cooked Beef with Caramelised Onion & Cabernet Sauvignon Pies | Unhealthy | Unhealthy |
| Slow Cooked Beef with Portobello Mushroom & Merlot | Unhealthy | Unhealthy |
| Beef Pastry Parcels with Red Wine | Unhealthy | Unhealthy |
| Cheddar and Pancetta Quiche | Unhealthy | Unhealthy |
| Beef Pies with a Hickory Bourbon Style Sauce | Unhealthy | Unhealthy |
| Chicken and Leek Pie | Unhealthy | Unhealthy |
| Beef Pies | Unhealthy | Unhealthy |
| Leek, Mushroom, Cheese & Chive Quiche | Unhealthy | Unhealthy |
| Ham & Cheese Croissant Sandwich | Unhealthy | Unhealthy |
| Salmon Parcels with a Leek & Cheese Sauce | Unhealthy | Unhealthy |
| Thai Style Red Chicken Curry Pies | Unhealthy | Unhealthy |
| Spinach and Feta Quiche | Unhealthy | Unhealthy |
| Snack Angus Beef Rolls | Unhealthy | Unhealthy |
| Beef Pie | Unhealthy | Unhealthy |
| Shortcrust & Puff Pastry Encased Chunky Beef & Red Wine Gravy | Unhealthy | Unhealthy |
| Goats Cheese & Cranberry Quiche | Unhealthy | Unhealthy |
| Classic Meat Pies | Unhealthy | Unhealthy |
| Quiche, Lorraine, commercial, baked | Unhealthy | Unhealthy |
| Dim sim, vegetable & meat filling, purchased frozen, microwaved or steamed | Unhealthy | Unhealthy |
| Pastry, filled with spinach, commercial, ready to eat | Unhealthy | Unhealthy |
| Pastry, filled with spinach & cheese, from frozen, baked, no added fat | Unhealthy | Unhealthy |
| Pastry, filled with spinach & cheese, commercial, ready to eat | Unhealthy | Unhealthy |
| Pasty, filled with vegetables, commercial, baked | Unhealthy | Unhealthy |
| Pasty, filled with vegetables & meat, commercial, ready to eat | Unhealthy | Unhealthy |
| Pie, savoury, chicken & vegetable, commercial | Unhealthy | Unhealthy |
| Pie, savoury, meat, commercial | Unhealthy | Unhealthy |
| Pie, savoury, meat, commercial, family size | Unhealthy | Unhealthy |
| Pie, savoury, meat, from frozen, baked or microwaved | Unhealthy | Unhealthy |
| Pie, savoury, meat & cheese, commercial | Unhealthy | Unhealthy |
| Pie, savoury, meat & kidney, commercial | Unhealthy | Unhealthy |
| Pie, savoury, meat & mushroom, from frozen, baked or microwaved | Unhealthy | Unhealthy |
| Pie, savoury, meat filling, topped with potato, commercial | Unhealthy | Unhealthy |
| Pie, savoury, spinach & cheese, commercial | Unhealthy | Unhealthy |
| Pie, savoury, without pastry, meat filling, topped with mashed potato, commercial | Unhealthy | Unhealthy |
| Pie, steak & kidney, canned | Unhealthy | Unhealthy |
| Sausage roll, commercial, ready to eat | Unhealthy | Unhealthy |
| Sausage roll, from frozen, baked or microwaved | Unhealthy | Unhealthy |
| Chiko roll, takeaway style, deep fried | Unhealthy | Unhealthy |
| **Soup** | | |
| Spicy Pumpkin Soup | Unhealthy | Unhealthy |
| Moroccan Pumpkin with Chickpea Soup | Unhealthy | Unhealthy |
| South American Style Chicken & Corn Soup | Unhealthy | Unhealthy |
| Thai Pumpkin Soup | Unhealthy | Unhealthy |
| Chicken and Corn Soup | Unhealthy | Unhealthy |
| Vegetable and Bean Soup | Unhealthy | Unhealthy |
| Creamy Pumpkin Ready to Serve Soup | Unhealthy | Unhealthy |
| Pumpkin with Ginger Organic Soup | Unhealthy | Unhealthy |
| Spicy Vegetable & Red Lentil Soup | Unhealthy | Unhealthy |
| Chunky Beef Soup | Unhealthy | Unhealthy |
| Chunky Roast Chicken & Vegetable Soup | Unhealthy | Unhealthy |
| Minestrone Soup | Unhealthy | Unhealthy |
| Country Chicken Soup | Unhealthy | Unhealthy |
| New England Style Vegetable Chowder Cup Soup | Unhealthy | Unhealthy |
| Tomato & Grilled Pepper Cup Soup | Unhealthy | Unhealthy |
| Creamy Chicken Soup | Unhealthy | Unhealthy |
| Country Pumpkin & Lentil Soup | Unhealthy | Unhealthy |
| Rustic Vegetable & Quinoa Soup | Unhealthy | Unhealthy |
| Dutch Curry with Quinoa Instant Soup | Unhealthy | Unhealthy |
| Kale, Quinoa & Vegetable Soup | Unhealthy | Unhealthy |
| Creamy Cauliflower with Aged Cheddar & Chives Soup | Unhealthy | Unhealthy |
| Lentil Soup | Unhealthy | Unhealthy |
| Tamarind Soup Base | Unhealthy | Unhealthy |
| Lamb & Lentil Soup | Unhealthy | Unhealthy |
| Classic Hearty Beef Flavoured Soup Mix | Unhealthy | Unhealthy |
| Lots-a-Noodles Beef Soup | Unhealthy | Unhealthy |
| Hearty Spanish Tomato Soup | Unhealthy | Unhealthy |
| Creamy Mushroom Soup with Croutons | Unhealthy | Unhealthy |
| Free Range Chicken Bone Broth | Healthy | Healthy |
| Vegetable, Lentil & Chilli Soup with Smoked Paprika | Unhealthy | Unhealthy |
| White Bean, Leek & Chicken Soup with Basil & White Pepper | Unhealthy | Unhealthy |
| Sweet Potato Bacon & Kale Soup with Ginger | Unhealthy | Unhealthy |
| All Natural Hot Korean Soup | Unhealthy | Unhealthy |
| All Natural Tom Yum Soup | Unhealthy | Unhealthy |
| Mushroom Soup | Unhealthy | Unhealthy |
| Vegetable Soup | Unhealthy | Unhealthy |
| Cream of Pumpkin with Croutons Instant Soup | Unhealthy | Unhealthy |
| Spring Vegetable Soup | Unhealthy | Unhealthy |
| Southern Style Pulled Pork Soup | Unhealthy | Unhealthy |
| Spiced Lentil & Tomato Soup | Unhealthy | Unhealthy |
| Creamy Chicken Flavoured Soup with Noodles | Unhealthy | Unhealthy |
| French Onion Soup | Unhealthy | Unhealthy |
| Australian Chunky Chicken & Veg Soup | Unhealthy | Unhealthy |
| Classic French Onion Soup | Unhealthy | Unhealthy |
| Pea & Ham Soup | Unhealthy | Unhealthy |
| Carrot & Coconut Soup | Unhealthy | Unhealthy |
| Chicken & Corn with Croutons Soup in a Cup | Unhealthy | Unhealthy |
| Chicken & Vegetable Soup | Unhealthy | Unhealthy |
| Pea & Ham Flavoured Soup with Croutons | Unhealthy | Unhealthy |
| 100% Australian Organic Chicken, Spelt & Vegetable Soup | Healthy | Healthy |
| Creamy Mushroom Soup with Bacon & Herbs | Unhealthy | Unhealthy |
| Creamy Tomato & Herb Soup | Unhealthy | Unhealthy |
| Vine Ripened Tomato Soup | Unhealthy | Unhealthy |
| Carrot & Ginger Soup | Unhealthy | Unhealthy |
| Roast Beef with Tomato & Thyme Soup Mix | Unhealthy | Unhealthy |
| All Natural Mexican Bean Soup | Unhealthy | Unhealthy |
| Italian Style Tomato & Red Pepper Soup | Unhealthy | Unhealthy |
| French Onion with Croutons Soup Mix | Unhealthy | Unhealthy |
| Chicken Noodle Soup | Unhealthy | Unhealthy |
| Peppered Steak Soup | Unhealthy | Unhealthy |
| Beef Bone Broth | Unhealthy | Healthy |
| Seafood Chowder Soup | Healthy | Healthy |
| Green Onions Instant Miso Soup | Unhealthy | Unhealthy |
| Creamy Chicken Soup | Unhealthy | Unhealthy |
| All Natural Laksa Noodle Soup | Unhealthy | Unhealthy |
| Sweet Potato & Corn with Wild Rice Soup | Healthy | Healthy |
| All Natural Hot & Sour Noodle Soup | Unhealthy | Unhealthy |
| Aussie Lamb Stockpot Soup | Unhealthy | Unhealthy |
| Pumpkin with Sour Cream & Chives Soup Mix | Unhealthy | Unhealthy |
| Nonna's Roast Veggies & Black Barley Soup | Healthy | Healthy |
| Creamy Cauliflower with Aged Cheddar & Chives Soup Mix | Unhealthy | Unhealthy |
| Hearty Homestyle Chicken & Bean Soup | Unhealthy | Unhealthy |
| Cauliflower & Smoked Bacon Soup | Unhealthy | Unhealthy |
| Potato & Leek Soup | Healthy | Healthy |
| Chunky Beef, Vegetable & Barley Soup | Unhealthy | Unhealthy |
| Dutch Curry Soup with Rice | Unhealthy | Unhealthy |
| Asian Laksa Soup | Unhealthy | Unhealthy |
| Classic Pumpkin Soup | Unhealthy | Unhealthy |
| Chicken Noodle Flavoured Soup Mix | Unhealthy | Unhealthy |
| Chicken Flavour Thai Noodle Soup | Unhealthy | Unhealthy |
| Soup, Asian style meat & vegetable, with noodles, cup of soup, instant dry mix | Unhealthy | Unhealthy |
| Soup, Asian style seafood & vegetable, with noodles, cup of soup, instant dry mix | Unhealthy | Unhealthy |
| Soup, broth style with meat, cup of soup, instant dry mix | Unhealthy | Unhealthy |
| Soup, chicken & noodle, cup of soup, instant dry mix | Unhealthy | Unhealthy |
| Soup, chicken, with or without vegetables, cup of soup, instant dry mix | Unhealthy | Unhealthy |
| Soup, chicken, with or without vegetables, cup of soup, instant dry mix, reduced salt | Unhealthy | Unhealthy |
| Soup, chicken & vegetable, with pasta or croutons, cup of soup, instant dry mix | Unhealthy | Unhealthy |
| Soup, pea & ham, cup of soup, instant dry mix | Unhealthy | Unhealthy |
| Soup, cream variety, instant dry mix | Unhealthy | Unhealthy |
| Soup, French onion, instant dry mix | Unhealthy | Unhealthy |
| Soup, French onion, instant dry mix, reduced salt | Unhealthy | Unhealthy |
| Soup, mixed vegetables, reduced energy, cup of soup, instant dry mix | Unhealthy | Unhealthy |
| Soup, tomato, with or without noodles, cup of soup, instant dry mix | Unhealthy | Unhealthy |
| Soup, vegetable, cup of soup, instant dry mix | Unhealthy | Unhealthy |
| Soup, broth style with meat, prepared from instant dry mix with water | Unhealthy | Unhealthy |
| Soup, chicken & noodle, cup of soup, prepared from instant dry mix with water | Unhealthy | Unhealthy |
| Soup, cream variety, prepared from dry mix with water | Unhealthy | Unhealthy |
| Soup, cup of soup style with croutons, prepared from instant dry mix with water | Unhealthy | Unhealthy |
| Soup, mixed vegetables, prepared from instant dry mix with water | Unhealthy | Unhealthy |
| Soup, vegetable, cup of soup, prepared from instant dry mix with water | Unhealthy | Unhealthy |
| Soup, cream of chicken, condensed, canned | Unhealthy | Unhealthy |
| Soup, cream of vegetables, condensed, canned | Unhealthy | Unhealthy |
| Soup, tomato, condensed, canned | Unhealthy | Unhealthy |
| Soup, chicken & noodle, ready to eat, canned | Unhealthy | Unhealthy |
| Soup, chicken & sweetcorn, ready to eat, canned | Unhealthy | Unhealthy |
| Soup, chicken & vegetable, ready to eat, canned | Unhealthy | Unhealthy |
| Soup, cream of chicken, condensed, canned, prepared with regular fat cows milk & water | Unhealthy | Unhealthy |
| Soup, cream of seafood, condensed, canned, prepared with regular fat cows milk & water | Unhealthy | Unhealthy |
| Soup, meat & vegetable, canned, ready-to-eat, heated | Unhealthy | Unhealthy |
| Soup, pea & ham, condensed, canned, prepared with water | Unhealthy | Unhealthy |
| Soup, pea & ham, ready to eat, canned | Unhealthy | Unhealthy |
| Soup, cream of vegetables, condensed, canned, prepared with regular fat cows milk & water | Unhealthy | Unhealthy |
| Soup, cream of vegetable, ready to eat, canned | Unhealthy | Unhealthy |
| Soup, pumpkin, ready to eat, canned | Unhealthy | Unhealthy |
| Soup, tomato, condensed, canned, prepared with water | Unhealthy | Unhealthy |
| Soup, tomato, condensed, canned, prepared with cows milk & water | Unhealthy | Unhealthy |
| Soup, tomato, ready to eat, canned | Unhealthy | Unhealthy |
| Soup, vegetable, ready to eat, canned | Unhealthy | Unhealthy |
| **Dairy** | | |
| **Cheese** | | |
| Cheddar Cheese Spread & Crispbread Crackers | Unhealthy | Unhealthy |
| Vintage Cheese Bites | Healthy | Healthy |
| Organic Creamy Blue | Unhealthy | Unhealthy |
| Parmesan Cheese | Unhealthy | Unhealthy |
| Cholesterol Lowering Cheese | Unhealthy | Unhealthy |
| Aged Parmesan | Unhealthy | Unhealthy |
| Chocolate Cream Cheese Block | Unhealthy | Unhealthy |
| Chive and Onion Cream Cheese | Unhealthy | Unhealthy |
| Organic Goat's Milk Feta Cheese | Unhealthy | Unhealthy |
| Milawa Goat Camembert | Healthy | Healthy |
| 5 Cheese Italian Style Cooking Blend | Unhealthy | Healthy |
| Shaved Italian Grana Padano | Healthy | Healthy |
| Spring Onion Cream Cheese | Healthy | Healthy |
| Grated Cheese | Unhealthy | Unhealthy |
| Finely Sliced Tasty Cheese | Unhealthy | Unhealthy |
| Sweet Chilli Philly Pourover Cream Cheese | Unhealthy | Unhealthy |
| Cheddar & Chive & Onion Cheese | Unhealthy | Unhealthy |
| Vintage Cheddar Cheese | Unhealthy | Unhealthy |
| Maasdam Cheese | Healthy | Healthy |
| Chicken Flavoured Cream Cheese Spread with Breadsticks | Unhealthy | Unhealthy |
| Melon & Mango Cheese | Unhealthy | Unhealthy |
| Sweet Chilli Cheese | Unhealthy | Unhealthy |
| Frozen Buffalo Mozzarella | Unhealthy | Unhealthy |
| Traditional Halloumi Cheese | Unhealthy | Unhealthy |
| Apricot & Almond Cheese | Unhealthy | Unhealthy |
| Black Truffle Australian Fetta | Unhealthy | Healthy |
| Original Spreadable Cheese | Unhealthy | Unhealthy |
| Stockwhip Wild Pepperberries Cheese | Healthy | Healthy |
| Barrel Aged Feta Cheese in Brine | Unhealthy | Unhealthy |
| Italian Buffalo Mozzarella Shredded Cheese | Healthy | Healthy |
| Dairy Free Cheddar Flavoured Bio Cheese Slices | Unhealthy | Unhealthy |
| Tassie Trio | Unhealthy | Unhealthy |
| Cherry Bocconcini | Healthy | Healthy |
| Tasty Shred Cheese | Unhealthy | Unhealthy |
| Three Cheese Blend | Unhealthy | Healthy |
| Cream Cheese with Garlic and Herbs | Unhealthy | Unhealthy |
| Lemon Pepper Marinated Feta | Unhealthy | Unhealthy |
| Tasmanian Atlantic Salmon Light Spreadable Cream Cheese Snack Tubs | Unhealthy | Unhealthy |
| Authentic Greek Feta Cheese | Unhealthy | Unhealthy |
| Halloumi | Unhealthy | Unhealthy |
| Bocconcini | Healthy | Healthy |
| Original Cream Cheese Spread with Breadsticks | Unhealthy | Unhealthy |
| Reduced Fat Wrapped Sandwich Cheese Slices | Unhealthy | Unhealthy |
| Original Cheese Slices | Unhealthy | Unhealthy |
| Australian Tasty Shredded Cheese | Unhealthy | Unhealthy |
| Chef Style Shredded Cheddar | Unhealthy | Healthy |
| Light Cheese Slices | Unhealthy | Unhealthy |
| Mozzarella Cheese Alternative | Unhealthy | Unhealthy |
| Haloumi | Unhealthy | Unhealthy |
| Smoked Buffalino Buffalo Cheese | Unhealthy | Unhealthy |
| Traditional Greek-Style Feta in Oil with Herbs & Garlic | Unhealthy | Unhealthy |
| Traditional Greek-Style Feta in Oil with Black Olives | Unhealthy | Unhealthy |
| Haloumi with Cracked Pepper | Unhealthy | Unhealthy |
| Brie Cheese Spread | Unhealthy | Unhealthy |
| Rubicon Red Cheese | Unhealthy | Healthy |
| Bush Pepper Cheese | Healthy | Healthy |
| Cherrie Bocconcini | Healthy | Healthy |
| Grated Parmesan Cheese | Unhealthy | Unhealthy |
| Marinated Goat Cheese | Unhealthy | Unhealthy |
| Italian Grana Padano | Healthy | Healthy |
| Cheddar Cheese Snackpacks | Unhealthy | Unhealthy |
| Dairy Free Shredded Parmesan Cheese | Unhealthy | Unhealthy |
| Dairy Free Cheddar Shred Bio Cheese | Unhealthy | Unhealthy |
| Natural Australian Mozzarella Shredded Cheese | Unhealthy | Healthy |
| Traditional Buffalo Bocconcini | Unhealthy | Unhealthy |
| Salted Honey Cow | Healthy | Healthy |
| Pocessed Cheese Slices | Unhealthy | Unhealthy |
| Spreadable Light Cream Cheese | Healthy | Healthy |
| Haloumi Cheese | Unhealthy | Unhealthy |
| Bold Blue Cheese | Unhealthy | Unhealthy |
| Greek Feta | Unhealthy | Unhealthy |
| Dairy Free Bio Cheese Pizza Shred | Unhealthy | Unhealthy |
| Extra Light Cheese Slices | Unhealthy | Unhealthy |
| Tasty Cheese & Crackers | Unhealthy | Unhealthy |
| Tasty Cheese & Rice Crackers | Unhealthy | Unhealthy |
| Saganaki Greek Frying Cheese | Unhealthy | Unhealthy |
| Creamy Blue Cheese | Healthy | Healthy |
| Fetta Full Cream | Unhealthy | Unhealthy |
| Pickled Onion & Chive Infused Club Cheddar | Healthy | Healthy |
| Fine Shred Extra Sharp Parmesan Cheese | Unhealthy | Unhealthy |
| P.D.O. Parmigiano Reggiano | Healthy | Healthy |
| Bold Cream Cheese Spread | Unhealthy | Unhealthy |
| Buffalo Mozzarella Cheese | Healthy | Healthy |
| Cream Cheese | Unhealthy | Unhealthy |
| Cyprus Style Haloumi Cheese | Unhealthy | Unhealthy |
| Australian Colby Cheese Slices | Healthy | Healthy |
| Cheese Triangles | Unhealthy | Unhealthy |
| Tasty Cheese & Gourmet Crackers with Rosemary & Rock Salt | Unhealthy | Unhealthy |
| Tasty Cheese Flavoured Cream Cheese & Crackers | Unhealthy | Unhealthy |
| Original Cheese Slices | Unhealthy | Unhealthy |
| Garden Herb & Onion Twist Spreadable Cream Cheese | Unhealthy | Unhealthy |
| Creamy Cheese Dip and Crunchy Breadsticks | Unhealthy | Unhealthy |
| Cheese Pods | Unhealthy | Unhealthy |
| Tasmanian Smoked Salmon with Lemon & Dill Spreadable Cream Cheese | Unhealthy | Unhealthy |
| Tomato & Basil Cream Cheese | Unhealthy | Unhealthy |
| Cranberry & Macadamia Cream Cheese | Unhealthy | Unhealthy |
| Honey & Pistachio Cream Cheese | Unhealthy | Unhealthy |
| Australian Haloumi Cheese | Unhealthy | Unhealthy |
| Classic Cottage Cheese | Unhealthy | Unhealthy |
| Barossa Triple Cream Cheese | Healthy | Healthy |
| Creamy Ash Brie | Healthy | Healthy |
| Creamy Washed Rind Brie | Unhealthy | Unhealthy |
| Mexican Inspired Cheese | Unhealthy | Unhealthy |
| Organic Camembert | Healthy | Healthy |
| Soft Cream Cheese | Unhealthy | Unhealthy |
| Dairy Free Mozzarella Style Slices | Unhealthy | Unhealthy |
| Bio Cheese Original Block | Unhealthy | Unhealthy |
| Manchego Cheese | Healthy | Healthy |
| Vegan Cashew Parmesan Dressing | Healthy | Healthy |
| Processed Cheese Slices | Unhealthy | Unhealthy |
| Fig & Fennel with Wattleseed Cheesboard Pourover | Unhealthy | Unhealthy |
| Light Cheese Slices | Unhealthy | Unhealthy |
| PDO Asiago Cheese | Unhealthy | Unhealthy |
| Dairy Free Mozzarella Style Shreds | Unhealthy | Unhealthy |
| Dairy Free Cheddar Style Shreds | Unhealthy | Unhealthy |
| Black Pepper Cream Cheese | Unhealthy | Unhealthy |
| Chive & Spring Onion Cream Cheese | Unhealthy | Unhealthy |
| Tropical Fruit & Almond Cream Cheese | Unhealthy | Unhealthy |
| Camembert Cheese | Healthy | Healthy |
| Original Natural Cheese | Unhealthy | Unhealthy |
| Bocconcini Dairy Free Cheese | Unhealthy | Healthy |
| Swiss Cheese | Healthy | Healthy |
| Vintage Cheese | Unhealthy | Unhealthy |
| Balsamic Strawberry with Mountain Pepper Cheeseboard Pourover | Unhealthy | Unhealthy |
| Double Cream Camembert | Healthy | Healthy |
| Monterey Jack Cheese Slices | Unhealthy | Unhealthy |
| Swiss Style Cheese Slices | Healthy | Healthy |
| Apricot and Almond with Sweet Papaya Cream Cheese | Unhealthy | Unhealthy |
| Apple Cider Vinegar & Chive Crunchy Cheese Snack | Unhealthy | Unhealthy |
| Crunchy Havarti Cheese Snack | Unhealthy | Unhealthy |
| Sweet Chilli & Sour Cream Flavoured Wholesome Crunchy Cheese Snack | Unhealthy | Unhealthy |
| Cheddar Cheese Crunchy Snack | Unhealthy | Unhealthy |
| Pizza Supreme Wholesome Crunchy Cheese Snack | Unhealthy | Unhealthy |
| Salted Caramel Wholesome Crunchy Cheese Snack | Unhealthy | Unhealthy |
| Original Cheese Slices | Unhealthy | Unhealthy |
| Vintage Cheese & Crackers | Healthy | Healthy |
| Semi Dried Tomato & Fetta | Healthy | Healthy |
| Double Cream Cheese with Chilli | Healthy | Healthy |
| Yorkshire Wensleydale & Apricots Cheese | Unhealthy | Healthy |
| Lactose Free Original All Natural Cream Cheese Block | Unhealthy | Healthy |
| Haloumi Kebab with Sweet Chilli Sauce | Unhealthy | Unhealthy |
| Bulgarian Goat's Cheese | Unhealthy | Unhealthy |
| Lemon & Coconut Cream Cheese | Unhealthy | Unhealthy |
| Peach & Almond Cream Cheese | Unhealthy | Unhealthy |
| Greek Style Fetta Cubes in Brine with Citrus & Herbs | Unhealthy | Unhealthy |
| Cheese, blended for pizza use, regular fat | Unhealthy | Unhealthy |
| Cheese, blue vein | Unhealthy | Unhealthy |
| Cheese, cheddar, natural, flavoured | Unhealthy | Unhealthy |
| Cheese, cheddar, natural, plain, regular fat | Unhealthy | Unhealthy |
| Cheese, cheshire | Healthy | Healthy |
| Cheese, colby style | Healthy | Healthy |
| Cheese, edam | Unhealthy | Unhealthy |
| Cheese, fetta (feta), regular fat | Unhealthy | Unhealthy |
| Cheese, goat, firm | Healthy | Healthy |
| Cheese, goat, soft | Healthy | Healthy |
| Cheese, gloucester style | Healthy | Healthy |
| Cheese, gouda | Unhealthy | Unhealthy |
| Cheese, haloumi | Unhealthy | Unhealthy |
| Cheese, havarti style | Healthy | Healthy |
| Cheese, jarlsberg | Healthy | Healthy |
| Cheese, mozzarella, buffalo | Healthy | Healthy |
| Cheese, mozzarella, regular fat | Healthy | Healthy |
| Cheese, parmesan, dried, finely grated | Unhealthy | Unhealthy |
| Cheese, parmesan, fresh | Unhealthy | Unhealthy |
| Cheese, pecorino style | Unhealthy | Unhealthy |
| Cheese, provolone style | Unhealthy | Unhealthy |
| Cheese, romano style | Unhealthy | Unhealthy |
| Cheese, swiss | Healthy | Healthy |
| Cheese, cheddar, natural, plain, reduced fat (~25%) | Healthy | Healthy |
| Cheese, cheddar, natural, plain, reduced fat (~ 15%) | Healthy | Healthy |
| Cheese, fetta (feta), reduced fat | Unhealthy | Unhealthy |
| Cheese, mozzarella, reduced fat | Healthy | Healthy |
| Cheese, bocconcini | Healthy | Healthy |
| Cheese, cottage, regular fat | Healthy | Healthy |
| Cheese, cream, plain, regular fat (35% fat) | Healthy | Healthy |
| Cheese, cream, fruit flavoured | Unhealthy | Unhealthy |
| Cheese, cream, herb or spice flavoured | Unhealthy | Healthy |
| Cheese, neufchatel | Healthy | Healthy |
| Cheese, ricotta, regular fat | Healthy | Healthy |
| Cheese, cottage, reduced fat | Healthy | Healthy |
| Cheese, cream, plain, reduced fat (25% fat) | Healthy | Healthy |
| Cheese, cream, plain, reduced fat (5% fat) | Healthy | Healthy |
| Cheese, quark | Healthy | Healthy |
| Cheese, ricotta, reduced fat | Healthy | Healthy |
| Cheese, brie | Healthy | Healthy |
| Cheese, camembert | Healthy | Healthy |
| Cheese, soft, white mould coated, not further defined | Unhealthy | Unhealthy |
| Cheese spread, cheddar, regular fat | Unhealthy | Unhealthy |
| Cheese spread, cream cheese, regular fat | Unhealthy | Healthy |
| Cheese, cheddar, processed, regular fat | Unhealthy | Unhealthy |
| Cheese, cheddar, processed, babybel style | Unhealthy | Unhealthy |
| Cheese, cheddar, processed, stick style | Unhealthy | Unhealthy |
| Cheese, processed, stick shape with string texture | Unhealthy | Unhealthy |
| Cheese spread, cream cheese, reduced fat | Unhealthy | Unhealthy |
| Cheese, cheddar, processed, reduced fat (~16%) | Unhealthy | Unhealthy |
| Cheese, cheddar, processed, reduced fat (~8%) | Unhealthy | Unhealthy |
| Cheese, cheddar, processed, reduced fat (3%) | Unhealthy | Unhealthy |
| Cheese, processed, with added phytosterols | Unhealthy | Unhealthy |
| Cheese, for use on sandwiches, not further defined | Unhealthy | Unhealthy |
| Cheese, soy | Unhealthy | Unhealthy |
| **Cream** | | |
| Irish Cream Flavoured Original Cream | Unhealthy | Unhealthy |
| Light Sour Cream | Unhealthy | Unhealthy |
| Double Thick Dollop Cream | Unhealthy | Unhealthy |
| Espresso Cream | Unhealthy | Healthy |
| Vanilla Cream | Unhealthy | Healthy |
| Light Thickened Cream | Unhealthy | Unhealthy |
| Pure Double Cream | Healthy | Healthy |
| Chocolate Heaven Whipped Cream | Unhealthy | Healthy |
| Blue Carnival Vanilla Flavour Whipped Cream | Unhealthy | Unhealthy |
| Lactose Free Light Sour Cream | Unhealthy | Healthy |
| Crème Fraîche | Healthy | Healthy |
| Regular Sour Cream | Healthy | Healthy |
| Creme Fraiche | Healthy | Healthy |
| Whipped Cream | Unhealthy | Unhealthy |
| Catering Whipped Cream | Unhealthy | Unhealthy |
| Pure Cream | Healthy | Healthy |
| Dollop Thick Cream | Unhealthy | Unhealthy |
| Light Thickened Cream | Unhealthy | Unhealthy |
| Thickened Cream | Unhealthy | Healthy |
| Cream, pure, 35% fat | Healthy | Healthy |
| Cream, regular thickened, 35% fat | Healthy | Healthy |
| Cream, regular thickened, 35% fat, ultra high temperature treated | Healthy | Healthy |
| Cream, rich or double thick | Healthy | Healthy |
| Cream, whipped, aerosol, regular fat (~28%) | Healthy | Healthy |
| Cream, reduced fat (~25%), canned | Healthy | Healthy |
| Cream, regular thickened, light (~18% fat) | Healthy | Healthy |
| Cream, sour, regular fat | Healthy | Healthy |
| Cream, sour, light (~18% fat) | Healthy | Healthy |
| Cream, sour, extra light (>12% fat) | Healthy | Healthy |
| Cream, imitation or mock (non-dairy) | Unhealthy | Unhealthy |
| Cream, imitation or mock (non-dairy), reduced fat | Unhealthy | Unhealthy |
| Cream, dairy, sugar sweetened, whipped, commercial | Healthy | Healthy |
| Cream, for use in commercial bakery products, not further defined | Healthy | Healthy |
| **Dairy desserts** | | |
| Crème Brûlée Mix | Unhealthy | Unhealthy |
| Luscious Red Velvet Trifle | Unhealthy | Unhealthy |
| Salted Caramel Semifreddo Mix | Unhealthy | Unhealthy |
| New York Baked Cheesecake | Unhealthy | Unhealthy |
| Crème Brûlée | Unhealthy | Unhealthy |
| French Cream Cheesecake | Unhealthy | Unhealthy |
| Original Baked Cheesecake | Unhealthy | Unhealthy |
| Chocolate Cheesecake | Unhealthy | Unhealthy |
| Gooey Belgian Chocolate & Pear Lava Puddings | Unhealthy | Unhealthy |
| Original Custard | Unhealthy | Unhealthy |
| Belgian Chocolate Mousse | Unhealthy | Unhealthy |
| Creamy Vanilla Flavoured Pouring Custard | Unhealthy | Unhealthy |
| Belgian Chocolate Mousse with Peppermint | Unhealthy | Unhealthy |
| Belgian White Chocolate Mousse with Raspberry Compote | Unhealthy | Unhealthy |
| Peach Mousse with Peach & Passionfruit Compote | Unhealthy | Unhealthy |
| Chocolate Ganache Baked Cheesecake | Unhealthy | Unhealthy |
| Peach in Vanilla Flavoured Custard | Unhealthy | Unhealthy |
| Vanilla Flavour Custard | Unhealthy | Unhealthy |
| Burnt Toffee with Macadamia Pieces Almond Based Frozen Dessert | Unhealthy | Unhealthy |
| Creamy Rice Snack with Vanilla | Unhealthy | Healthy |
| Creamy Rice Snack with Coconut Milk | Healthy | Healthy |
| Panna Cotta | Unhealthy | Unhealthy |
| La Cassata Cake | Unhealthy | Unhealthy |
| Vanilla Custard | Unhealthy | Unhealthy |
| Panna Cotta with Raspberry Coulis | Unhealthy | Unhealthy |
| Chocolate Mousse | Unhealthy | Unhealthy |
| Chocolate Custard | Unhealthy | Healthy |
| Tiramisu | Unhealthy | Unhealthy |
| Sea Salt Caramel Mousse & Dark Chocolate Ganache | Unhealthy | Unhealthy |
| Peanut Butter Bavarian | Unhealthy | Unhealthy |
| Coconut Flavoured Mochi Ice Dessert | Unhealthy | Unhealthy |
| Tiramisu | Unhealthy | Unhealthy |
| Chocolate Stracciatella Cheesecake | Unhealthy | Unhealthy |
| Mango Tapioca Pudding | Unhealthy | Unhealthy |
| Black Rice Pudding | Unhealthy | Unhealthy |
| Mango & Passionfruit Tapioca Pearl Pudding | Unhealthy | Unhealthy |
| Raspberry & Coconut Tapioca Pearl Pudding | Unhealthy | Unhealthy |
| Dark Mint Raw Food Dessert | Unhealthy | Unhealthy |
| Choc-Moose | Unhealthy | Unhealthy |
| Vanilla Bean Rice Pudding | Unhealthy | Unhealthy |
| Chocolate Hazelnut Mousse Bites | Unhealthy | Unhealthy |
| Caramel Choc Delight Frozen Gelato Dessert | Unhealthy | Unhealthy |
| Raspberry & White Chocolate Indulgent Dessert | Unhealthy | Unhealthy |
| Chocolate Salted Caramel Indulgent Dessert | Unhealthy | Unhealthy |
| Strawberry Flavoured Coconut Milk Mousse | Unhealthy | Healthy |
| Chocolate Flavoured Coconut Milk Mousse | Unhealthy | Healthy |
| Mango Flavoured Coconut Milk Mousse | Unhealthy | Healthy |
| Brownie & Cheesecake | Unhealthy | Unhealthy |
| White Chocolate & Raspberry Baked Cheesecake | Unhealthy | Unhealthy |
| Vanilla Flavoured Custard | Unhealthy | Unhealthy |
| Black Sesame Flavoured Mochi Ice Dessert | Unhealthy | Unhealthy |
| Green Tea Flavoured Mochi Ice Dessert | Unhealthy | Unhealthy |
| Triple Choc Chunk Bavarian | Unhealthy | Unhealthy |
| White Choc Chunk and Raspberry Bavarian | Unhealthy | Unhealthy |
| Chocolate & Coconut Dessert Pots | Unhealthy | Unhealthy |
| Chocolate & Burnt Fig Dessert Pots | Unhealthy | Unhealthy |
| Rich Deep Dish Chocolate Ganache Cheesecake | Unhealthy | Unhealthy |
| Chocolate Crémes | Unhealthy | Unhealthy |
| Deep Dish Sticky Butterscotch & Apple Pudding | Unhealthy | Unhealthy |
| Caramel Crèmes | Unhealthy | Unhealthy |
| Chocolate Dessert | Unhealthy | Unhealthy |
| Chocolate Flavoured Custard | Unhealthy | Unhealthy |
| Vanilla and Rum Flavoured Custard | Unhealthy | Unhealthy |
| Italian Style Panna Cotta | Unhealthy | Unhealthy |
| Brandy Flavoured Custard | Unhealthy | Unhealthy |
| Chocolate Trifle | Unhealthy | Unhealthy |
| Matcha Green Tea Crème Brûlée | Unhealthy | Unhealthy |
| Double Chocolate Dessert | Unhealthy | Unhealthy |
| Chocolate Mousse | Unhealthy | Unhealthy |
| Chocolate Mousse | Unhealthy | Unhealthy |
| Creamy Rice Snack with Cacao | Unhealthy | Unhealthy |
| Custard & Cherry Trifle Cake | Unhealthy | Unhealthy |
| Ruby Chocolate Cheesecake | Unhealthy | Unhealthy |
| Custard powder, dry mix, commercial | Unhealthy | Unhealthy |
| Custard, dairy, regular fat, banana, commercial | Unhealthy | Unhealthy |
| Custard, dairy, regular fat, chocolate, commercial | Unhealthy | Unhealthy |
| Custard, dairy, regular fat, vanilla, commercial | Unhealthy | Unhealthy |
| Custard, dairy, reduced fat, vanilla, commercial | Unhealthy | Unhealthy |
| Custard pudding, creme caramel or brulee, regular fat, vanilla, commercial | Unhealthy | Unhealthy |
| Dairy dessert, chocolate, regular fat | Unhealthy | Unhealthy |
| Dairy dessert, chocolate, regular fat, added vitamins A, B1, B2, C, D & folate, Ca & Fe, commercial | Unhealthy | Unhealthy |
| Dairy dessert, flavours other than chocolate, regular fat | Unhealthy | Healthy |
| Fromais frais, berry pieces or flavour, regular fat (5% fat) | Unhealthy | Unhealthy |
| Fromais frais, fruit pieces or flavoured, regular fat (5% fat) | Unhealthy | Unhealthy |
| Fromais frais, vanilla flavoured, regular fat (5% fat) | Unhealthy | Unhealthy |
| Pudding, rice, dairy, vanilla, commercial | Unhealthy | Unhealthy |
| Pudding, rice, dairy, chocolate, commercial | Unhealthy | Unhealthy |
| Cake, cheesecake, biscuit base, chocolate or coffee flavoured cream cheese topping, commercial | Unhealthy | Unhealthy |
| Cake, cheesecake, biscuit base, fruit flavoured cream cheese topping, commercial | Unhealthy | Unhealthy |
| Cake, cheesecake, biscuit base, plain cream cheese topping, commercial | Unhealthy | Unhealthy |
| Pavlova, plain, commercial | Unhealthy | Unhealthy |
| Pavlova, plain, topped with whipped cream | Unhealthy | Unhealthy |
| **Ice cream and edible ices** | | |
| Creamy Ice Bars | Unhealthy | Unhealthy |
| Peanut Butter & Jam Flavoured Ice Cream | Unhealthy | Unhealthy |
| Mango Ice Cream | Unhealthy | Unhealthy |
| Lemon Sorbet | Unhealthy | Unhealthy |
| 3 in 1 Triple Chocolate Ice Cream | Unhealthy | Unhealthy |
| Salted Caramel with Chocolate Coated Hazelnuts Ice Cream | Unhealthy | Unhealthy |
| Cola with Raspberry Goo Ice Cream | Unhealthy | Unhealthy |
| Blood Orange and Tahitian Lime Sorbet | Unhealthy | Unhealthy |
| Pineapple, Coconut & Lime Sorbet | Unhealthy | Unhealthy |
| Choc Coated Ice Confection Sticks | Unhealthy | Unhealthy |
| Tropical Fruit Pops | Unhealthy | Unhealthy |
| Coconut + Mango Fruit Ice | Unhealthy | Unhealthy |
| Raspberry Ripple Ice Cream | Unhealthy | Unhealthy |
| Hazelnut and Chocolate Il Tartufo Ice Cream | Unhealthy | Unhealthy |
| Freeze Pops | Unhealthy | Unhealthy |
| Scooby-Doo! Choc Milk Ice Cream | Unhealthy | Unhealthy |
| Lime Spider Ice Cream | Unhealthy | Unhealthy |
| Coconut Milk Vanilla Bean Ice Dream | Unhealthy | Unhealthy |
| Vanilla Bean Missing You Coconut Milk Ice Cream | Unhealthy | Unhealthy |
| Vanilla Bean Dairy Based Premium Gelato | Unhealthy | Unhealthy |
| Mango Super Premium Sorbet | Unhealthy | Unhealthy |
| Pandan Pecan Party! Coconut Milk Ice Cream | Unhealthy | Unhealthy |
| Strawberries & Cream Ice Cream | Unhealthy | Unhealthy |
| Ice Blocks | Unhealthy | Unhealthy |
| Choc! Choc! Who's There Coconut Milk Ice Cream | Unhealthy | Unhealthy |
| Jammy Custard Donut Ice Cream Cone | Unhealthy | Unhealthy |
| Raspberry Jelly Pops | Unhealthy | Unhealthy |
| Orange you Glad Juice Pops | Healthy | Healthy |
| Blackcurrant Jelly Pops | Unhealthy | Unhealthy |
| Paging Dr. Green Frozen Juice Pops | Unhealthy | Unhealthy |
| Turn Up Beet Frozen Juice Pops with Granny Smith Apple, Beetroot, Carrot & Ginger | Healthy | Healthy |
| Dark Chocolate and Coconut Sorbet | Unhealthy | Unhealthy |
| Golden Gaytime Ice Cream Cones | Unhealthy | Unhealthy |
| Salted Caramel Ice Cream | Unhealthy | Unhealthy |
| Simply Coconut Fruit Ice | Unhealthy | Unhealthy |
| Jelly Splits with Lemonade and Raspberry Jelly Centre | Unhealthy | Unhealthy |
| Cappuccino Buzz Ice Cream | Unhealthy | Unhealthy |
| Salted Coconut Cream with Australian Sea Salt Whole Fruit Ice Pops | Healthy | Healthy |
| Watermelon and Lemonade Whole Fruit Ice Pops | Healthy | Healthy |
| Cloudy Apple and Passionfruit Whole Fruit Ice Pops | Healthy | Healthy |
| French Vanilla Ice Cream | Unhealthy | Unhealthy |
| Rainbow Ice Cream | Unhealthy | Unhealthy |
| Vanilla Blueberry Ice Cream | Unhealthy | Unhealthy |
| Chocolate Hazelnut Fudge Flavoured Non-Dairy Frozen Dessert | Unhealthy | Unhealthy |
| Italian Style Pomegranate Sorbet | Unhealthy | Unhealthy |
| Watermelon Berry Mint Pops | Unhealthy | Unhealthy |
| Dairy-Free Dark Chocolate and Coconut Bars | Unhealthy | Unhealthy |
| I Fell for Caramel Coconut Milk Ice Cream | Unhealthy | Unhealthy |
| Brownie Batter Core Ice Cream | Unhealthy | Unhealthy |
| Berry Passionate Frozen Juice Pops with Packham Pear, Raspberry, Strawberry & Passionfruit | Unhealthy | Unhealthy |
| Summer Sunset Orange & Pink Grapefruit Fruit Ice Sticks | Unhealthy | Unhealthy |
| Keep Calm & Carrot on Juice Pops with Apple, Carrot, Celery & Lemon | Unhealthy | Unhealthy |
| Iced Coconut Cream Real Fruit Ice Pops | Unhealthy | Unhealthy |
| Dairy Free Simply Coconut Dessert | Unhealthy | Unhealthy |
| Classic Frozen Custard | Unhealthy | Unhealthy |
| Blueberry Cheesecake Ice Cream | Unhealthy | Unhealthy |
| Vanilla Flavoured Frozen Custard | Unhealthy | Unhealthy |
| Rich Chocolate Ice Cream Cone | Unhealthy | Unhealthy |
| Italian Style Passionfruit Sorbet | Unhealthy | Unhealthy |
| Lemonade and Raspberry Flavoured Ice Pops | Unhealthy | Unhealthy |
| Chocolate Ice Cream Sticks | Unhealthy | Unhealthy |
| Caramel Almond Brittle Non-Dairy Frozen Dessert | Unhealthy | Unhealthy |
| Coffee Caramel Fudge Non-Dairy Frozen Dessert | Unhealthy | Unhealthy |
| Coconut Seven Layer Bar Non-Dairy Frozen Dessert | Unhealthy | Unhealthy |
| Peanut Butter Organic Coconut Milk Ice Cream | Unhealthy | Unhealthy |
| Dream Team Cookies 'n Cream Coconut Ice Cream | Unhealthy | Unhealthy |
| Caramel Macchiato Dairy Free Frozen Dessert | Unhealthy | Unhealthy |
| Candy Bar Frozen Dessert | Unhealthy | Unhealthy |
| Chocolate Chip Cookie Dough Frozen Dessert | Unhealthy | Unhealthy |
| Pana Chocolate Mint & Pana Choc Chip Organic Ice Cream Alternative | Unhealthy | Unhealthy |
| We're Mint to Be Coconut Ice Cream | Unhealthy | Unhealthy |
| Salted Caramel Organic Coconut Ice Cream | Unhealthy | Unhealthy |
| Choc Peanut Butter Organic Dairy Free Coconut Ice Cream | Unhealthy | Unhealthy |
| Hazelnut Salted Vanilla Ice Cream | Unhealthy | Unhealthy |
| Vanilla Opulence Gourmet Ice Cream | Unhealthy | Unhealthy |
| Limited Edition Josh & Nic's Italian Style Hazelnut Choc Gelato | Unhealthy | Unhealthy |
| Strawberry Daiquiri Frozen Dessert | Unhealthy | Unhealthy |
| Coconut with Raspberry Swirl Non Dairy Premium Ice Cream | Unhealthy | Unhealthy |
| Double Choc Ice Cream | Unhealthy | Unhealthy |
| Salted Caramel Dairy Free Organic Coconut Ice Cream | Unhealthy | Unhealthy |
| Matcha Green Tea Organic Coconut Milk Ice Cream | Unhealthy | Unhealthy |
| Chocolate Italian Gelati | Unhealthy | Unhealthy |
| Dairy Free Boysenberry, Acai and Coconut Frozen Dessert | Unhealthy | Unhealthy |
| Real Fruit Apple, Strawberry and Banana Ice Sticks | Unhealthy | Unhealthy |
| Vanilla Bean Flavour Dairy Free Ice Cream | Unhealthy | Unhealthy |
| Chocolate & Honeycomb Flavoured Ice Cream Cones with Chocolate Coated Crunchie Bits | Unhealthy | Unhealthy |
| Lemongrass Caramel & Macadamia Dairy Free Organic Coconut Ice Cream | Unhealthy | Unhealthy |
| Salted Caramel Dairy Free Ice Cream | Unhealthy | Unhealthy |
| Chocolate Coated Vanilla Bean Frozen Dessert | Unhealthy | Unhealthy |
| Raspberry Frozen Dessert | Unhealthy | Unhealthy |
| Mango Mini Sorbet Sticks | Unhealthy | Unhealthy |
| Double Chocolate Dairy Free Ice Cream | Unhealthy | Unhealthy |
| Aussie Summer Pine-Lime Ice Sticks | Unhealthy | Unhealthy |
| Caramel & Peanut Ice Cream Sticks | Unhealthy | Unhealthy |
| Watermelon Slice | Unhealthy | Unhealthy |
| Choc Peanut Butter Dairy-Free Organic Coconut Ice Cream | Unhealthy | Unhealthy |
| Mint Choc Chip Dairy-Free Coconut Ice Cream | Unhealthy | Unhealthy |
| Dairy Free Classic Ice Cream | Unhealthy | Unhealthy |
| Vanilla Flavoured Soy Ice Cream | Unhealthy | Unhealthy |
| Organic Mango Passion Sorbet Ice Blocks | Unhealthy | Unhealthy |
| Café Grande Gourmet Ice Cream | Unhealthy | Unhealthy |
| Mango Pops | Unhealthy | Unhealthy |
| Lemonade Ice Blocks | Unhealthy | Unhealthy |
| Coconuts for Mango | Unhealthy | Unhealthy |
| Pineapple Milkshake Flavour Ice Cream | Unhealthy | Unhealthy |
| Red Bean Pops | Unhealthy | Unhealthy |
| Hazelnut Chocolate Truffle Dairy Free Ice Cream | Unhealthy | Unhealthy |
| Berry Me in Coconut Ice Cream | Unhealthy | Unhealthy |
| Chocolate Ice Confection with Compound Chocolate and Honeycomb Pieces | Unhealthy | Unhealthy |
| Coffee Avocado Frozen Dessert | Unhealthy | Unhealthy |
| Meringue Semifreddo | Unhealthy | Unhealthy |
| Musang King Durian Snow Ball | Unhealthy | Unhealthy |
| Tasmanian Mint Gourmet Ice Cream Cookies with Chocolate Chip Cookies | Unhealthy | Unhealthy |
| Spiders Ice Creams | Unhealthy | Unhealthy |
| Vanilla Frozen Dessert | Unhealthy | Unhealthy |
| Strawberry Cream and Real Fruit Ice Pops | Unhealthy | Healthy |
| Berry Panna Cotta Ice Cream | Unhealthy | Unhealthy |
| Intense Dark with 70% Cocoa Ice Cream | Unhealthy | Unhealthy |
| Berry Biscuit White Chocolate Ice Cream | Unhealthy | Unhealthy |
| Strawberry Ice Confection | Unhealthy | Unhealthy |
| Cloudy Apple Ice Confection | Unhealthy | Unhealthy |
| Mango & Coconut Ice Confection | Unhealthy | Unhealthy |
| Blueberry, Strawberry & Raspberry Fruity Bars | Unhealthy | Unhealthy |
| Banana & Caramel Dairy Free Ice Cream | Unhealthy | Unhealthy |
| Pineapple, Passionfruit, Lime & Yuzu Fruity Bars | Unhealthy | Unhealthy |
| Vanilla Bean Plant Based Frozen Dessert | Unhealthy | Unhealthy |
| Cookies & Caramel Ice Cream | Unhealthy | Unhealthy |
| Madagascan Vanilla Bean with Salted Caramel Ripple Gelato Cookies | Unhealthy | Unhealthy |
| Ice cream, caramel flavour, regular fat | Unhealthy | Unhealthy |
| Ice cream, caramel, honey & macadamia flavour, premium or rich (~15% fat) | Unhealthy | Unhealthy |
| Ice cream, chocolate or coffee flavour, with or without chocolate chips, regular fat | Unhealthy | Unhealthy |
| Ice cream, chocolate & caramel swirl, regular fat | Unhealthy | Unhealthy |
| Ice cream, chocolate & honey swirl, with chocolate & nougat, premium or rich (~15% fat) | Unhealthy | Unhealthy |
| Ice cream, mint flavour, regular fat | Unhealthy | Unhealthy |
| Ice cream, neopolitan or rainbow flavour, regular fat | Unhealthy | Unhealthy |
| Ice cream, strawberry flavour, regular fat | Unhealthy | Unhealthy |
| Ice cream, vanilla flavour, premium or rich (~15% fat) | Unhealthy | Unhealthy |
| Ice cream, vanilla flavour, regular fat | Unhealthy | Unhealthy |
| Ice cream, vanilla flavour, with chocolate chip, regular fat | Unhealthy | Unhealthy |
| Ice cream, all other non-chocolate or coffee flavours, with chocolate chips, regular fat | Unhealthy | Unhealthy |
| Ice cream, vanilla flavour, with nuts, regular fat | Unhealthy | Unhealthy |
| Ice cream, vanilla flavour, with sweet biscuit, regular fat | Unhealthy | Unhealthy |
| Ice cream, vanilla & caramel swirl, regular fat | Unhealthy | Unhealthy |
| Ice cream, vanilla & chocolate or coffee flavour, regular fat | Unhealthy | Unhealthy |
| Ice cream cake, not further defined | Unhealthy | Unhealthy |
| Ice cream, Cassata-style dessert | Unhealthy | Unhealthy |
| Ice cream, mango flavour, regular fat | Unhealthy | Unhealthy |
| Ice cream, passionfruit flavour, regular fat | Unhealthy | Unhealthy |
| Ice cream, rum & raisin flavour, regular fat | Unhealthy | Unhealthy |
| Ice cream, vanilla flavour, soft serve, fast food style | Unhealthy | Unhealthy |
| Ice cream, vanilla flavour, not further defined | Unhealthy | Unhealthy |
| Ice cream, vanilla flavour, with chocolate chips, reduced fat (6%) | Unhealthy | Unhealthy |
| Ice cream, vanilla flavour, with sweet biscuit, low fat | Unhealthy | Unhealthy |
| Ice cream, not further defined | Unhealthy | Unhealthy |
| Ice cream, caramel flavour, low fat (3% fat) | Unhealthy | Unhealthy |
| Ice cream, chocolate flavour, with or without chocolate chips, low fat (3% fat) | Unhealthy | Unhealthy |
| Ice cream, chocolate & caramel swirl, low fat (3% fat) | Unhealthy | Unhealthy |
| Ice cream, mint flavour, low fat (3% fat) | Unhealthy | Unhealthy |
| Ice cream, neopolitan or rainbow flavour, low fat (3% fat) | Unhealthy | Unhealthy |
| Ice cream, strawberry flavour, low fat (3% fat) | Unhealthy | Unhealthy |
| Ice cream, vanilla flavour, low fat (3% fat) | Unhealthy | Unhealthy |
| Ice cream, bar, Turkish delight flavour, chocolate coated | Unhealthy | Unhealthy |
| Ice cream, sandwich, vanilla flavour, with chocolate flavour biscuit & chocolate coated, regular fat | Unhealthy | Unhealthy |
| Ice cream, stick, chocolate flavoured, chocolate & biscuit crumb coated, regular fat | Unhealthy | Unhealthy |
| Ice cream, stick, flavoured, chocolate & biscuit crumb coated, regular fat | Unhealthy | Unhealthy |
| Ice cream, stick, vanilla flavour, chocolate coated, regular fat | Unhealthy | Unhealthy |
| Ice cream, stick, other non-chocolate flavours, chocolate coated, regular fat | Unhealthy | Unhealthy |
| Ice cream, stick, vanilla flavour, chocolate coated, with almonds, regular fat | Unhealthy | Unhealthy |
| Ice cream, stick, vanilla flavour, with caramel sauce, chocolate coated, regular fat | Unhealthy | Unhealthy |
| Ice cream, chocolate flavour with confectionery & waffle cone, chocolate coated, regular fat | Unhealthy | Unhealthy |
| Ice cream, vanilla flavour with confectionery & waffle cone, chocolate coated, regular fat | Unhealthy | Unhealthy |
| Ice cream, sandwich, vanilla flavour, with chocolate flavour biscuit, regular fat | Unhealthy | Unhealthy |
| Ice cream, stick, vanilla flavour, fruit ice confection-coated, regular fat | Unhealthy | Unhealthy |
| Ice cream, other flavours with confectionery & waffle cone, chocolate coated, regular fat | Unhealthy | Unhealthy |
| Ice confection, stick, milk-based, chocolate flavoured, regular fat | Unhealthy | Unhealthy |
| Ice confection, stick, milk-based, other flavours, regular fat | Unhealthy | Unhealthy |
| Ice cream, with fruit based ice confection, various flavours, regular fat | Unhealthy | Unhealthy |
| Yoghurt, frozen, berry flavoured, regular fat | Unhealthy | Unhealthy |
| Yoghurt, frozen, tropical or fruit salad flavoured, regular fat | Unhealthy | Unhealthy |
| Gelato or sorbet, milk-based, chocolate or coffee flavoured, regular fat | Unhealthy | Unhealthy |
| Gelato or sorbet, milk-based, vanilla & other flavours, regular fat | Unhealthy | Unhealthy |
| Ice cream, chocolate flavour, regular fat, added vitamins A, B1, B2, C, D & folate, Ca & Fe | Unhealthy | Unhealthy |
| Ice cream, soft serve, vanilla, with chocolate cream filled biscuit, fast food style | Unhealthy | Unhealthy |
| Ice cream cone, soft serve vanilla ice cream, with wafer cone, fast food style | Unhealthy | Unhealthy |
| Sundae, vanilla ice cream, caramel topping, fast food style | Unhealthy | Unhealthy |
| Sundae, vanilla ice cream, chocolate topping | Unhealthy | Unhealthy |
| Sundae, vanilla ice cream, chocolate topping, fast food style | Unhealthy | Unhealthy |
| Sundae, vanilla ice cream, non-chocolate topping | Unhealthy | Unhealthy |
| Sundae, vanilla ice cream, strawberry topping, fast food style | Unhealthy | Unhealthy |
| Ice confection, non-dairy, chocolate or coffee flavour, regular fat | Unhealthy | Unhealthy |
| Ice confection, non-dairy, vanilla flavour, regular fat | Unhealthy | Unhealthy |
| Gelato or sorbet, fruit or fruit juice, regular fat | Unhealthy | Unhealthy |
| Ice confection, stick, water-base, various flavours, regular fat | Unhealthy | Unhealthy |
| Ice confection, stick or tub, fruit juice or fruit flavoured, regular fat | Unhealthy | Unhealthy |
| **Milk** | | |
| Unsweetened Almond Milk | Unhealthy | Unhealthy |
| Protein Enriched Rice Milk | Healthy | Healthy |
| Espresso Shot Flavoured Milk | Unhealthy | Healthy |
| Fresh Goat Milk | Healthy | Healthy |
| Permeate Free Light Milk | Healthy | Healthy |
| Almond Milk | Unhealthy | Healthy |
| Unsweetened Soy Milk | Unhealthy | Healthy |
| Affogato Flavoured Milk | Unhealthy | Healthy |
| Arabica Beans Latte Barista Style Iced Coffee | Unhealthy | Unhealthy |
| Hilo Milk | Healthy | Healthy |
| Robusta Beans Double Espresso Barista Style Iced Coffee | Unhealthy | Unhealthy |
| Mocha Barista Style Iced Coffee | Unhealthy | Unhealthy |
| Choc Lamington Flavoured Milk | Unhealthy | Unhealthy |
| High Protein Full Cream Milk | Healthy | Healthy |
| Organic Coconut Milk | Unhealthy | Unhealthy |
| Lite Soy Milk | Unhealthy | Unhealthy |
| No Added Sugar Banana Mylk | Unhealthy | Unhealthy |
| 100% Dairy Free Caffe Latte | Unhealthy | Unhealthy |
| Regular Strength Iced Coffee | Healthy | Healthy |
| Pasteurised Reduced Fat Milk with Coffee | Healthy | Healthy |
| Coconut Mochacinno | Unhealthy | Unhealthy |
| Christmas Egg Nog | Unhealthy | Unhealthy |
| Coconut Double Shot Espresso | Unhealthy | Unhealthy |
| Matcha Green Tea Mylk | Unhealthy | Unhealthy |
| Unsweetened Coconut Milk | Unhealthy | Unhealthy |
| Cold Pressed Latte Arabica Coffee | Healthy | Healthy |
| A Hint of Strawberry Milk | Unhealthy | Unhealthy |
| Black & White Cold Brew Coffee with Almond | Unhealthy | Unhealthy |
| Espresso Flavoured Milk | Unhealthy | Unhealthy |
| Premium Coconut Milk Powder | Unhealthy | Unhealthy |
| Iced Coffee Flavoured High Protein Low Fat Milk | Unhealthy | Unhealthy |
| Iced Mochaccino Flavoured Milk | Unhealthy | Unhealthy |
| Double Espresso Iced Coffee | Unhealthy | Unhealthy |
| Unsweetened Almond Coconut Milk | Unhealthy | Unhealthy |
| Unsweetened Vanilla Almond Milk with Added Calcium | Unhealthy | Unhealthy |
| Vanilla Flavoured Milk Drink | Unhealthy | Unhealthy |
| UHT Chocolate Flavoured Milk | Unhealthy | Unhealthy |
| Coconut Milk Coffee | Unhealthy | Unhealthy |
| Cold Brew Iced Coffee Flavoured with Baileys | Unhealthy | Unhealthy |
| Cold Pressed Strong Latte Arabica Coffee | Healthy | Healthy |
| Lactose Free Milk | Healthy | Healthy |
| Chocolate Milk | Unhealthy | Unhealthy |
| Iced Arabica Coffee Almond Milk | Unhealthy | Unhealthy |
| The Original Sweetened Condensed Milk | Unhealthy | Unhealthy |
| Salted Caramel Flavoured Milk Drink | Unhealthy | Unhealthy |
| Taiwan Banana Milk | Unhealthy | Unhealthy |
| Double Shot Iced Coffee | Unhealthy | Healthy |
| Coffee | Unhealthy | Healthy |
| Beverage with Real Chia, Real Vanilla, Real Milk and Real Oats | Unhealthy | Healthy |
| Ice Coffee Breakfast Smoothie | Unhealthy | Healthy |
| Easy to Digest Chocolate Milk | Unhealthy | Healthy |
| Soy Milk | Healthy | Healthy |
| Unsweetened Soy Milk | Unhealthy | Healthy |
| Malt Soy Drink | Unhealthy | Unhealthy |
| Chocolate Flavoured Milk | Unhealthy | Unhealthy |
| Homogenised Pasteurised Milk | Healthy | Healthy |
| Vanilla Coconut Milk Yoghurt | Unhealthy | Healthy |
| Banana and Mango Coconut Milk Yoghurt | Unhealthy | Healthy |
| Activated Almond Milk | Unhealthy | Healthy |
| Almond & Coconut Milk | Unhealthy | Healthy |
| Choco Refreshing Chocolate Flavoured Coconut Milk Drink with Added Protein | Unhealthy | Unhealthy |
| Semi Skim Milk | Healthy | Healthy |
| Rich Sweetened Condensed Milk | Unhealthy | Unhealthy |
| Chocolate Almond Milk | Unhealthy | Unhealthy |
| Coffee Cashew | Unhealthy | Unhealthy |
| Lactose Free Light Milk | Healthy | Healthy |
| Cold Pressed Coffee | Healthy | Healthy |
| Chocolate Flavoured Coconut and Almond Blend | Unhealthy | Unhealthy |
| Light Milk | Healthy | Healthy |
| Cookies & Cream Flavoured Milk | Unhealthy | Unhealthy |
| Strawberry Flavoured Milk | Unhealthy | Unhealthy |
| Raspberry Pavlova Flavoured Milk | Unhealthy | Unhealthy |
| Murray River Salted Chocolate Flavoured Milk | Unhealthy | Unhealthy |
| High Protein Full Cream Milk | Healthy | Healthy |
| XX Espresso Cold Brew Coffee with Almond Blend | Unhealthy | Unhealthy |
| Sweetened Condensed Milk | Unhealthy | Unhealthy |
| Go Coconuts Coconut & Coconut Water Blend | Unhealthy | Unhealthy |
| Strawberry Milk | Unhealthy | Unhealthy |
| Almond Milk with Organic Cacao | Unhealthy | Unhealthy |
| Skim Sweetened Condensed Milk | Unhealthy | Unhealthy |
| Long Life UHT Full Cream Milk | Healthy | Healthy |
| Colombia La Unión, Nariño Cold Brew Coffee | Healthy | Healthy |
| Banana Honey & Cinnamon Breakfast Smoothie | Unhealthy | Unhealthy |
| Mint Slice Inspired Flavoured Milk | Unhealthy | Unhealthy |
| Vanilla Malt Flavoured Milk | Unhealthy | Healthy |
| Dark Choc Espresso Ice Coffee | Unhealthy | Unhealthy |
| Cold Press Iced Coffee | Healthy | Healthy |
| Full Cream Wholesome Fresh Milk | Healthy | Healthy |
| Almond Milk for Coffee | Unhealthy | Unhealthy |
| Chocolate A2 Protein Dairy Milk | Unhealthy | Unhealthy |
| Soy Milk | Unhealthy | Unhealthy |
| Unsweetened Coconut Milk | Healthy | Healthy |
| Unsweetened Oat Milk | Healthy | Healthy |
| Organic Hazel Quench Hazelnut Coconut Milk | Healthy | Healthy |
| Unsweetened Macadamia Milk | Unhealthy | Unhealthy |
| Original Oat Milk | Healthy | Healthy |
| Espresso Iced Coffee | Unhealthy | Unhealthy |
| Double Espresso Iced Coffee | Unhealthy | Unhealthy |
| Almond Milk | Unhealthy | Unhealthy |
| Barista Edition Oat Milk | Healthy | Healthy |
| Soy Milk | Unhealthy | Unhealthy |
| Oat Milk | Unhealthy | Unhealthy |
| Macadamia Milk for Coffee | Unhealthy | Unhealthy |
| Soy Milk | Healthy | Healthy |
| Soy Milk for Coffee | Unhealthy | Unhealthy |
| Strawberry Flavoured Milk | Unhealthy | Unhealthy |
| Skim Milk | Unhealthy | Healthy |
| Cola Spider Flavoured Milk | Unhealthy | Unhealthy |
| Lactose Free Full Cream Milk | Healthy | Healthy |
| Cold Brew Coffee with Milk | Healthy | Healthy |
| Iced Mocha Cold Brew Coffee | Unhealthy | Healthy |
| Salted Caramel Cold Brew Coffee | Healthy | Healthy |
| Vanilla Flavour Coconut Yoghurt | Unhealthy | Unhealthy |
| Strawberry Coconut Milk Yoghurt | Unhealthy | Unhealthy |
| Rumball Flavoured Milk | Unhealthy | Unhealthy |
| Allen's Red Skins Flavoured Milk | Unhealthy | Unhealthy |
| Nestlé Chokito Flavoured Milk | Unhealthy | Unhealthy |
| Milkshake, bubble tea, non-chocolate or coffee flavour, cows milk, tapioca pearls | Unhealthy | Unhealthy |
| Coffee whitener, dry powder | Unhealthy | Unhealthy |
| Milk, cow, fluid, regular fat (~3.5%) | Healthy | Healthy |
| Milk, cow, fluid, regular fat (~3.5%), A2 | Healthy | Healthy |
| Milk, cow, fluid, regular fat (~3.5%), organic | Healthy | Healthy |
| Milk, cow, fluid, regular fat (~3.5%), raw | Healthy | Healthy |
| Milk, cow, fluid, lactose free, regular fat (~3.5%) | Healthy | Healthy |
| Milk, cow, fluid, regular fat (3.5%), added omega 3 polyunsaturates | Healthy | Healthy |
| Milk, cow, fluid, reduced fat (10%) | Healthy | Healthy |
| Milk, cow, fluid, reduced fat (10%), A2 | Healthy | Healthy |
| Milk, cow, fluid, reduced fat (10%), organic | Healthy | Healthy |
| Milk, cow, fluid, reduced fat (10%), added milk solids | Healthy | Healthy |
| Milk, cow, fluid, reduced fat (~1.5%), increased protein (~4%) | Healthy | Healthy |
| Milk, cow, fluid, lactose free, reduced fat (~10%) | Healthy | Healthy |
| Milk, cow, fluid, reduced fat (10%), added phytosterols | Healthy | Healthy |
| Milk, cow, fluid, reduced fat (10%), increased Ca, added Fe & vitamins C & D | Healthy | Healthy |
| Milk, cow, fluid, reduced fat (1.5%), added Ca, Mg, Zn & vitamin D | Healthy | Healthy |
| Milk, cow, fluid, reduced fat (1.5%), added omega 3 polyunsaturates | Healthy | Healthy |
| Milk, cow, fluid, reduced fat (1.5%), increased Ca, folate & vitamin D | Healthy | Healthy |
| Milk, cow, fluid, skim (~0.15% fat) | Healthy | Healthy |
| Milk, cow, fluid, skim (~0.15% fat), added milk solids | Healthy | Healthy |
| Milk, canned, evaporated, regular | Healthy | Healthy |
| Milk, canned, evaporated, reduced fat (~ 2%) | Healthy | Healthy |
| Milk, canned, evaporated, skim (<0.5% fat) | Healthy | Healthy |
| Milk, canned, sweetened, condensed, regular | Unhealthy | Unhealthy |
| Milk, canned, sweetened, condensed, skim (~0.2% fat) | Unhealthy | Unhealthy |
| Milk, powder, cow, regular fat | Healthy | Healthy |
| Milk, powder, cow, skim | Healthy | Healthy |
| Milk, goat, fluid, regular fat | Healthy | Healthy |
| Milk, powder, goat, regular fat | Healthy | Healthy |
| Sheep's, milk or yoghurt | Healthy | Healthy |
| Milk, cow, fluid, unflavoured, not further defined | Healthy | Healthy |
| Buttermilk, cultured, 2% fat | Unhealthy | Healthy |
| Iced coffee, regular fat cows milk | Unhealthy | Unhealthy |
| Iced coffee, regular fat cows milk, with added sugar | Unhealthy | Unhealthy |
| Iced coffee, regular fat cows milk, with ice cream | Unhealthy | Unhealthy |
| Iced coffee, regular fat cows milk, with ice cream & whipped cream | Unhealthy | Unhealthy |
| Iced coffee, regular fat cows milk, with whipped cream | Unhealthy | Unhealthy |
| Iced chocolate, regular fat cows milk, with ice cream & whipped cream | Unhealthy | Unhealthy |
| Milk, cow, fluid, flavoured, chocolate, regular fat | Unhealthy | Unhealthy |
| Milk, cow, fluid, flavoured, chocolate, not further defined | Unhealthy | Unhealthy |
| Milk, cow, fluid, flavoured, coffee, regular fat | Unhealthy | Unhealthy |
| Milk, cow, fluid, flavoured, coffee, not further defined | Unhealthy | Unhealthy |
| Milkshake, chocolate or coffee flavour, regular fat cows milk, with ice cream | Unhealthy | Unhealthy |
| Milkshake, chocolate or coffee flavour, regular fat cows milk, without ice cream | Unhealthy | Unhealthy |
| Thickshake, chocolate or coffee flavour, regular fat cows milk, with ice cream | Unhealthy | Unhealthy |
| Thickshake, all flavours, with cows milk, crushed ice, ice cream & confectionary, fast food style | Unhealthy | Unhealthy |
| Milk, cow, fluid, flavoured, strawberry, regular fat | Unhealthy | Unhealthy |
| Milk, cow, fluid, flavoured, all other flavours, regular fat | Unhealthy | Unhealthy |
| Milkshake, non-chocolate or coffee flavours, regular fat cows milk, with ice cream | Unhealthy | Unhealthy |
| Milkshake, non-chocolate or coffee flavours, regular fat cows milk, without ice cream | Unhealthy | Unhealthy |
| Thickshake, non-chocolate or coffee flavour, regular fat cows milk, with ice cream | Unhealthy | Unhealthy |
| Iced coffee, reduced fat cows milk | Unhealthy | Unhealthy |
| Iced coffee, reduced fat cows milk, with ice cream | Unhealthy | Unhealthy |
| Milk, cow, fluid, flavoured, chocolate, reduced fat | Unhealthy | Unhealthy |
| Milk, cow, fluid, flavoured, chocolate, reduced fat, added Ca & vitamin D | Unhealthy | Unhealthy |
| Milk, cow, fluid, flavoured, chocolate, reduced fat, added vitamins & minerals | Unhealthy | Unhealthy |
| Milk, cow, fluid, flavoured, coffee, reduced fat | Unhealthy | Unhealthy |
| Milkshake, chocolate or coffee flavour, reduced fat cows milk, with ice cream | Unhealthy | Unhealthy |
| Milkshake, chocolate or coffee flavour, reduced fat cows milk, without ice cream | Unhealthy | Unhealthy |
| Milk, cow, fluid, flavoured, strawberry, reduced fat | Unhealthy | Unhealthy |
| Milk, cow, fluid, flavoured, all other flavours, reduced fat | Unhealthy | Unhealthy |
| Milkshake, non-chocolate or coffee flavours, reduced fat cows milk, with ice cream | Unhealthy | Unhealthy |
| Milkshake, non-chocolate or coffee flavours, reduced fat cows milk, without ice cream | Unhealthy | Unhealthy |
| Thickshake, caramel flavour, fast food style | Unhealthy | Unhealthy |
| Thickshake, chocolate flavour, fast food style | Unhealthy | Unhealthy |
| Thickshake, strawberry flavour, fast food style | Unhealthy | Unhealthy |
| Thickshake, vanilla flavour, fast food style | Unhealthy | Unhealthy |
| Drink, cows milk, added egg & sugar (eggnog or egg nog) | Unhealthy | Unhealthy |
| Smoothie, cows milk, all flavours, added banana | Unhealthy | Unhealthy |
| Smoothie, cows milk, all flavours, added berries | Unhealthy | Unhealthy |
| Smoothie, cows milk, all flavours, added mango | Unhealthy | Unhealthy |
| Smoothie, cows milk, all flavours, added mixed fruit | Unhealthy | Unhealthy |
| Smoothie, cows milk, all flavours, added mixed fruit & egg | Unhealthy | Unhealthy |
| Smoothie, cows milk, all flavours, added mixed fruit, guarana & herbal extracts | Unhealthy | Unhealthy |
| Smoothie, cows milk, all flavours, added mixed fruit & nuts or seeds | Unhealthy | Unhealthy |
| Beverage base, soy, dry powder (not infant food) | Unhealthy | Unhealthy |
| Soy beverage, regular fat (~3%), unfortified | Unhealthy | Unhealthy |
| Soy beverage, regular fat (~3%), homemade from basic ingredients, unfortified | Unhealthy | Unhealthy |
| Soy beverage, regular fat (~3%), added Ca | Unhealthy | Healthy |
| Soy beverage, regular fat (~3%), added Ca & vitamins A, B2, & B12 | Unhealthy | Unhealthy |
| Soy beverage, regular fat (~3%), added Ca & vitamins A, B1, B2 & B12 | Unhealthy | Unhealthy |
| Soy beverage, regular fat (~3%), added Ca & vitamins A, B1, B2, B6, B12, & D | Unhealthy | Healthy |
| Soy beverage, reduced fat (~10% fat), unfortified | Unhealthy | Unhealthy |
| Soy beverage, reduced fat (~1.5% fat), added Ca | Unhealthy | Healthy |
| Soy beverage, reduced fat (~1.5% fat), added vitamins A, B1, B2, B3, B6, B12, C, E, folate & Ca & Fe | Unhealthy | Unhealthy |
| Soy beverage, reduced fat (~1.5% fat), added fibre, Ca & vitamins A, B1, B2, B6 & B12 | Unhealthy | Healthy |
| Soy beverage, reduced fat (~10% fat), added Ca & vitamins A, B2 & B12 | Unhealthy | Healthy |
| Soy beverage, reduced fat (~10% fat), added Ca & vitamins A, B1, B2 & B12 | Unhealthy | Unhealthy |
| Soy beverage, low fat (~ 0.10%), added Ca & vitamins A, B1, B2 & B12 | Unhealthy | Unhealthy |
| Milk, almond, fluid | Unhealthy | Healthy |
| Milk, oat, fluid, unfortified | Unhealthy | Healthy |
| Milk, oat, fluid, added calcium | Unhealthy | Healthy |
| Milk, rice, fluid, added calcium | Unhealthy | Healthy |
| Milk, rice, fluid, protein enriched, added calcium | Unhealthy | Healthy |
| Babyccino, from soy milk | Unhealthy | Unhealthy |
| Smoothie, non-dairy base, all flavours, added mixed fruit | Unhealthy | Unhealthy |
| Soy beverage, regular fat (~3%), unflavoured, not further defined | Unhealthy | Unhealthy |
| Soy beverage, reduced fat (1-2%), unflavoured, not further defined | Unhealthy | Unhealthy |
| Soy beverage, unflavoured, not further defined | Unhealthy | Unhealthy |
| Iced coffee, regular fat soy milk | Unhealthy | Unhealthy |
| Milkshake, non-chocolate or coffee flavours, soy milk, without ice cream | Unhealthy | Unhealthy |
| Soy beverage, chocolate flavoured, regular fat (~3%), added Ca & vitamins A, B1, B2 & B12 | Unhealthy | Unhealthy |
| Soy beverage, chocolate flavoured, reduced fat (~ 1.5%), added Ca & vitamins A, B1, B2 & B12 | Unhealthy | Unhealthy |
| Soy beverage, coffee flavoured, reduced fat (~ 1.5%), added Ca & vitamins A, B1, B2 & B12 | Unhealthy | Unhealthy |
| **Yoghurt and yoghurt drinks** | | |
| Banana Maple Yogurt with Steel Cut Oats | Unhealthy | Unhealthy |
| Super Thick Greek Natural Yoghurt with Real Raspberry | Unhealthy | Unhealthy |
| Raspberry Flavoured Greek Yogurt | Unhealthy | Unhealthy |
| Cranberry Yogurt with Steel Cut Oats | Unhealthy | Unhealthy |
| Rhubarb and Vanilla Yoghurt | Unhealthy | Unhealthy |
| Passion Fruit Flavoured Fat Free Greek Yogurt | Unhealthy | Unhealthy |
| Mango Drinking Yogurt | Unhealthy | Unhealthy |
| Raspberry Flavoured Fat Free Greek Yogurt | Unhealthy | Unhealthy |
| Dahi Indian Style Yoghurt | Unhealthy | Healthy |
| Proactive Drink | Unhealthy | Unhealthy |
| Greek Style Strawberry Yogurt | Unhealthy | Unhealthy |
| Apple and Pear Greek Style Yoghurt | Unhealthy | Unhealthy |
| Blueberry Flavoured Coconut Yogurt | Unhealthy | Unhealthy |
| Berry Flavour Daily Probiotic Drink | Unhealthy | Unhealthy |
| Yogurt with Raspberry Flavoured Mini Jelly Balls | Unhealthy | Unhealthy |
| Low Fat Raspberry & Coconut Gourmet Pot Set Yogurt | Unhealthy | Unhealthy |
| Low Fat Mango & Passionfruit Gourmet Pot Set Yogurt | Unhealthy | Unhealthy |
| Blueberry Yogurt | Unhealthy | Unhealthy |
| Natural Coconut Yogurt | Healthy | Healthy |
| Guava Blood Orange Yogurt | Unhealthy | Unhealthy |
| Fresh Yoghurt with Raspberry | Unhealthy | Unhealthy |
| Blackberry Layered Yogurt | Unhealthy | Unhealthy |
| Sticky Apple & Organic Honey Yoghurt | Unhealthy | Unhealthy |
| Strawberry Greek Yogurt | Unhealthy | Healthy |
| Tropical Greek Yoghurt | Unhealthy | Healthy |
| Mixed Berry Flavoured Almond Milk Yogurt | Unhealthy | Unhealthy |
| Pumpkin Pie Yogurt | Unhealthy | Unhealthy |
| Café Latte Creamy Yoghourt | Unhealthy | Unhealthy |
| Choc Cherry Twist Yogurt | Unhealthy | Unhealthy |
| Gluten Free Coconut Milk Yoghurt | Unhealthy | Unhealthy |
| Natural Unsweetened Goat Yogurt | Unhealthy | Unhealthy |
| Mango and Blood Orange Twist Yogurt | Unhealthy | Unhealthy |
| Peach Mango Greek Yogurt with Steel Cut Oats & Ancient Grains | Unhealthy | Unhealthy |
| My Coco Coconut Milk & Blueberry Premium Yoghurt | Unhealthy | Unhealthy |
| Raspberry Powerpak Yogurt with Protein & Superfood | Unhealthy | Unhealthy |
| Mango Flavoured Probiotic Yogurt | Unhealthy | Unhealthy |
| Lemon Creme Low Fat Probiotic Yogurt | Unhealthy | Unhealthy |
| Mango & Peach Dairy Free Yogurt | Unhealthy | Unhealthy |
| Pro-Biotic Kefir Delicious Blueberry Yogurt | Unhealthy | Unhealthy |
| Passionfruit Flavoured Organic Yoghurt | Unhealthy | Unhealthy |
| Banana Flavoured Yogurt | Unhealthy | Unhealthy |
| Madagascan Vanilla Bean Yogurt | Healthy | Healthy |
| Kesar Mango Yogurt Smoothie | Unhealthy | Healthy |
| Mixed Berry Yoghurt Smoothie | Unhealthy | Unhealthy |
| Pleasantly Tart Blueberry Flavoured Kefir Probiotic Fermented Yogurt Drink | Unhealthy | Healthy |
| Key Lime Crumble Greek Yogurt | Unhealthy | Unhealthy |
| Chocolate Organic Coconut Yoghurt Alternative | Healthy | Healthy |
| Mixed Berry Flavoured Coconut Milk Yoghurt | Unhealthy | Healthy |
| Original Organic Probiotic Kefir | Unhealthy | Healthy |
| Passionfruit Greek Yogurt with Fruit on the Bottom | Unhealthy | Healthy |
| Strawberry Greek Yogurt with Fruit on the Bottom | Unhealthy | Unhealthy |
| Greek Style Yogurt with a Hint of Real Coconut | Unhealthy | Unhealthy |
| Banana Flavour Yogurt Mix | Unhealthy | Unhealthy |
| Natural Probiotic Coconut Yoghurt | Healthy | Healthy |
| Berries Probiotic Coconut Yogurt | Unhealthy | Unhealthy |
| Vanilla Probiotic Coconut Yogurt | Unhealthy | Healthy |
| Mango Probiotic Coconut Yoghurt | Healthy | Healthy |
| Strawberry Probiotic Coconut Yogurt | Unhealthy | Unhealthy |
| Whole Milk Vanilla Yogurt | Unhealthy | Unhealthy |
| Banana, Chia & Cardamom Organic Coconut Yoghurt Alternative | Healthy | Healthy |
| Yogurt Drink | Unhealthy | Unhealthy |
| Vanilla Flavoured Yoghurt | Unhealthy | Unhealthy |
| Plum & Guava Coconut Yoghurt Alternative | Unhealthy | Unhealthy |
| Mango & Passionfruit Coconut Yoghurt Slow Cultured Alternative | Healthy | Healthy |
| Pro-Biotic Fermented Drink | Unhealthy | Unhealthy |
| Blood Orange Flavoured Coconut Milk Yogurt | Unhealthy | Unhealthy |
| Mixed Berry Flavoured Coconut Milk Yogurt | Unhealthy | Healthy |
| Tropical Flavoured Yogurt | Unhealthy | Unhealthy |
| Natural Swedish-Style Quark Yogurt | Healthy | Healthy |
| Strawberry & Watermelon Flavoured Coconut Yogurt | Unhealthy | Unhealthy |
| Greek Style Yogurt with a Hint of Real Honey | Unhealthy | Healthy |
| Coconut Yogurt | Unhealthy | Unhealthy |
| Boyensberry Pot Set Yoghurt | Unhealthy | Unhealthy |
| Cookies & Cream Crunch Greek Yogurt | Unhealthy | Unhealthy |
| Natural Coconut Yogurt | Unhealthy | Healthy |
| All Natural Greek Style Yogurt | Healthy | Healthy |
| Sweetened Organic Coconut Yogurt Alternative | Unhealthy | Healthy |
| Chocolate Mousse Organic Coconut Yogurt Alternative | Unhealthy | Healthy |
| Salted Caramel Organic Coconut Yoghurt Alternative | Unhealthy | Unhealthy |
| Coconut Yoghurt with Raw Cacao | Healthy | Healthy |
| Blueberry Greek Yogurt with Steel Cut Oats | Unhealthy | Unhealthy |
| Natural Organic Probiotic Kefir | Unhealthy | Unhealthy |
| Natural Almond Milk Yogurt | Healthy | Healthy |
| Mango Dairy Free Coconut Yoghurt | Unhealthy | Unhealthy |
| Strawberry Top Down Yoghurt | Unhealthy | Unhealthy |
| Succulent Strawberry Flavoured Yoghurt | Unhealthy | Unhealthy |
| Luscious Blueberry Flavoured Yoghurt | Unhealthy | Unhealthy |
| Mango Flavoured Roasted Almond Milk Yoghurt | Unhealthy | Unhealthy |
| Blueberry Yogurt | Unhealthy | Unhealthy |
| Passionfruit Yogurt | Unhealthy | Unhealthy |
| Dairy Free Vanilla Coconut Yogurt | Unhealthy | Unhealthy |
| Probiotic Drink | Unhealthy | Unhealthy |
| Strawberry Dairy Free Coconut Yoghurt | Unhealthy | Unhealthy |
| Passionfruit Dairy Free Coconut Yoghurt | Unhealthy | Unhealthy |
| Natural Coconut Yoghurt | Healthy | Healthy |
| Sweet Plain Greek Yogurt | Unhealthy | Healthy |
| Organic Blueberry Yogurt | Unhealthy | Unhealthy |
| Watermelon Greek Yogurt | Unhealthy | Unhealthy |
| Extra Creamy Vanilla Flavoured Yoghurt | Unhealthy | Healthy |
| Natural Probiotic Yogurt | Unhealthy | Healthy |
| Apricot, Peach & Mango Flavoured Probiotic Yogurt | Unhealthy | Unhealthy |
| Natural Live Fermented Kefir Probiotic Yogurt | Healthy | Healthy |
| Natural Pot Set Yogurt | Healthy | Healthy |
| Dairy Free Vanilla Flavoured Almond Yoghurt | Unhealthy | Unhealthy |
| Passionfruit & Mango Yogurt Smoothie | Unhealthy | Unhealthy |
| Strawberry Flavour Almond Milk Yogurt | Unhealthy | Unhealthy |
| Vanilla Flavoured Cashew Yoghurt | Unhealthy | Unhealthy |
| Mint Choc Crunch Low Fat Yogurt | Unhealthy | Unhealthy |
| Mixed Berry Flavoured Yoghurt Smoothie | Unhealthy | Unhealthy |
| Coconut Probiotic Kefir | Unhealthy | Unhealthy |
| Probiotic Kefir Yoghurt with Strawberry & Cream | Unhealthy | Unhealthy |
| Vanilla Bean Yogurt | Unhealthy | Healthy |
| Coconut Yogurt | Unhealthy | Unhealthy |
| Mango Super Good Probiotic Yogurt | Unhealthy | Unhealthy |
| Roasted Almond Natural Yogurt | Unhealthy | Healthy |
| Raspberry Greek Yoghurt | Unhealthy | Healthy |
| Brandy Butter Caramel Twist Yogurt | Unhealthy | Unhealthy |
| Drink, probiotic, contains milk solids & sugar | Unhealthy | Unhealthy |
| Drink, probiotic, contains milk solids & sugar, intense sweetened | Unhealthy | Unhealthy |
| Yoghurt, Greek style (~10%), natural | Healthy | Healthy |
| Yoghurt, Greek style (~8%), natural | Healthy | Healthy |
| Yoghurt, Greek style, regular fat (~5%), natural | Healthy | Healthy |
| Yoghurt, natural, regular fat (~4%) | Healthy | Healthy |
| Yoghurt, natural, regular fat (~4%), homemade from basic ingredients | Healthy | Healthy |
| Yoghurt, Greek style, reduced fat (~2%), natural | Healthy | Healthy |
| Yoghurt, natural, reduced fat (~Unhealthy.5%) | Healthy | Healthy |
| Yoghurt, natural, reduced fat (~Unhealthy.5%), homemade from basic ingredients | Healthy | Healthy |
| Yoghurt, natural, low fat (<Healthy.5%) | Healthy | Healthy |
| Yoghurt, honey flavoured, high fat (~5%) | Unhealthy | Healthy |
| Yoghurt, peach, mango & passionfruit pieces or flavoured, high fat (~6%) | Unhealthy | Unhealthy |
| Yoghurt, apricot, peach or nectarine pieces or flavoured, regular fat (~3%) | Unhealthy | Unhealthy |
| Yoghurt, banana pieces or flavoured, regular fat (~3%) | Unhealthy | Unhealthy |
| Yoghurt, berry pieces or flavoured, regular fat (~3%) | Unhealthy | Unhealthy |
| Yoghurt, berry pieces or flavoured, with added fruit juice, reduced fat (~2%) | Unhealthy | Unhealthy |
| Yoghurt, flavoured, regular fat (~4%), homemade from basic ingredients | Unhealthy | Unhealthy |
| Yoghurt, mango pieces or flavoured, regular fat (~3%) | Unhealthy | Unhealthy |
| Yoghurt, passionfruit flavoured, regular fat (~3%) | Unhealthy | Healthy |
| Yoghurt, vanilla flavoured, regular fat (~3%) | Unhealthy | Unhealthy |
| Yoghurt, apricot, peach or nectarine pieces or flavoured, reduced fat (Un0%) | Unhealthy | Unhealthy |
| Yoghurt, berry pieces or flavoured, reduced fat (Un0%) | Unhealthy | Unhealthy |
| Yoghurt, dessert flavoured, reduced fat (~Un0%) | Unhealthy | Unhealthy |
| Yoghurt, flavoured, reduced fat (~Unhealthy.5%), homemade from basic ingredients | Unhealthy | Healthy |
| Yoghurt, honey flavoured, reduced fat (~2%) | Unhealthy | Unhealthy |
| Yoghurt, honey flavoured, reduced fat (~Un0%) | Unhealthy | Unhealthy |
| Yoghurt, mango pieces or flavoured, reduced fat (Un0%) | Unhealthy | Unhealthy |
| Yoghurt, passionfruit flavoured, reduced fat (Un0%) | Unhealthy | Unhealthy |
| Yoghurt, passionfruit flavoured, reduced fat (Un0%), reduced sugar | Unhealthy | Unhealthy |
| Yoghurt, peach & mango pieces or flavoured, reduced fat (2%) | Unhealthy | Unhealthy |
| Yoghurt, tropical fruit or fruit salad pieces or flavoured, with added fruit juice, reduced fat (~2%) | Unhealthy | Unhealthy |
| Yoghurt, vanilla flavoured, reduced fat (~Un0%) | Unhealthy | Unhealthy |
| Yoghurt, apricot, peach or nectarine pieces or flavoured, low fat (<Healthy.5%) | Unhealthy | Unhealthy |
| Yoghurt, berry pieces or flavoured, low fat (<Healthy.5%) | Unhealthy | Unhealthy |
| Yoghurt, vanilla flavoured, low fat (<Healthy.5%) | Unhealthy | Unhealthy |
| Yoghurt, apricot, peach or nectarine pieces or flavoured, low fat (<Healthy.5%), intense sweetened | Unhealthy | Unhealthy |
| Yoghurt, banana & honey flavoured, low fat (<Healthy.5%), intense sweetened | Unhealthy | Unhealthy |
| Yoghurt, berry pieces or flavoured, low fat (<Healthy.5%), intense sweetened | Unhealthy | Unhealthy |
| Yoghurt, dessert flavoured, low fat (<Healthy.5%), intense sweetened | Unhealthy | Unhealthy |
| Yoghurt, mango pieces or flavoured, low fat (<Healthy.5%), intense sweetened | Unhealthy | Unhealthy |
| Yoghurt, passionfruit flavoured, low fat (<Healthy.5%), intense sweetened | Unhealthy | Unhealthy |
| Yoghurt, peach & mango pieces or flavoured, low fat (<Healthy.5%), intense sweetened | Unhealthy | Unhealthy |
| Yoghurt, vanilla flavoured, reduced fat (~Un0%), intense sweetened | Unhealthy | Unhealthy |
| Yoghurt, vanilla flavoured, low fat (<Healthy.5%), intense sweetened | Unhealthy | Unhealthy |
| Yoghurt, drinking style, fruit flavoured, reduced fat (Un0%) | Unhealthy | Unhealthy |
| Yoghurt, drinking style, vanilla flavoured, reduced fat (Un0%) | Unhealthy | Unhealthy |
| Yoghurt, banana pieces or flavoured, regular fat (~3%), added omega-3 polyunsaturates | Unhealthy | Unhealthy |
| Yoghurt, berry pieces or flavoured, regular fat (~3%), added omega-3 polyunsaturates | Unhealthy | Unhealthy |
| Yoghurt, berry pieces or flavoured, reduced fat (2%), added vitamins A, C, E, & omega-3 polyunsaturates | Unhealthy | Unhealthy |
| Yoghurt, berry pieces or flavoured, reduced fat (Un0%), added fibre | Unhealthy | Unhealthy |
| Yoghurt, berry pieces or flavoured, low fat (<Healthy.5%), intense sweetened, added fibre | Unhealthy | Unhealthy |
| Yoghurt, dessert flavoured, low fat (<Healthy.5%), intense sweetened, added fibre | Unhealthy | Unhealthy |
| Yoghurt, fruit pieces or flavoured, reduced fat (~2%), added Ca & vitamin D | Unhealthy | Unhealthy |
| Yoghurt, fruit pieces, reduced fat (2%), added vitamins A, B1, B2, B3, B6, B12, C, D, E, & folate | Unhealthy | Unhealthy |
| Yoghurt, passionfruit flavoured, reduced fat (Un0%), added fibre | Unhealthy | Unhealthy |
| Yoghurt, peach & mango pieces or flavoured, reduced fat (Un0%), added fibre | Unhealthy | Unhealthy |
| Yoghurt, tropical fruit or fruit salad pieces or flavoured, regular fat (~3%), added omega-3 polyunsaturates | Unhealthy | Unhealthy |
| Yoghurt, tropical fruit or fruit salad pieces or flavoured, low fat (<Healthy.5%), intense sweetened, added fibre | Unhealthy | Unhealthy |
| Yoghurt, vanilla flavoured, regular fat (~3%), added omega-3 polyunsaturates | Unhealthy | Unhealthy |
| Yoghurt, vanilla flavoured, reduced fat (~Un0%), added fibre | Unhealthy | Unhealthy |
| Yoghurt, vanilla flavoured, low fat (<Healthy.5%), with added protein & fibre, intense sweetened | Unhealthy | Unhealthy |
| Yoghurt, soy based, berry flavoured, regular fat (approx. 3%) | Unhealthy | Healthy |
| Yoghurt, soy based, apricot or mango flavoured, reduced fat (approx. Un0%) | Unhealthy | Unhealthy |
| Yoghurt, soy based, berry flavoured, reduced fat (Un0%) | Unhealthy | Unhealthy |
| Yoghurt, soy based, vanilla flavoured, reduced fat (approx. Un0%) | Unhealthy | Unhealthy |
| **Edible oils and oil emulsions** | | |
| **Butter, margarine and other fats** | | |
| Cinnamon Flavour Spread | Unhealthy | Unhealthy |
| Chocolate Flavour Spread | Unhealthy | Unhealthy |
| Caramel Flavour Spread | Unhealthy | Unhealthy |
| Traditional Farmhouse Butter | Healthy | Healthy |
| Very Garlic Butter | Healthy | Healthy |
| Dairy Soft Spread | Healthy | Healthy |
| Unsalted Cultured Style Butter | Unhealthy | Healthy |
| Extra Soft Spread | Unhealthy | Unhealthy |
| Pure Irish Butter | Healthy | Healthy |
| Blend of Vegetable Oils and Dairy with a Pinch of Sea Salt | Unhealthy | Unhealthy |
| Blended Spread with Butter and a Pinch of Sea Salt | Unhealthy | Unhealthy |
| Regular Quark | Healthy | Healthy |
| Butter and Chia Oil | Healthy | Healthy |
| A Light Blend of Butter and Avocado Oil | Healthy | Healthy |
| Goose Fat | Healthy | Healthy |
| Salted Pure Spreadable Butter | Healthy | Healthy |
| Butter Blend Spread | Unhealthy | Unhealthy |
| Extra Virgin Coconut Oil | Healthy | Healthy |
| Cultured Style Unsalted Butter with Fresh Australian Cream | Healthy | Healthy |
| Salt Reduced Butter | Healthy | Healthy |
| Reduced Salt Butter | Healthy | Healthy |
| Unsalted Butter | Healthy | Healthy |
| Buttery Spread | Unhealthy | Unhealthy |
| Coconut Oil Blend | Unhealthy | Unhealthy |
| Italian Butter | Healthy | Healthy |
| Original Spread | Unhealthy | Unhealthy |
| Salted Pasteurized Butter | Healthy | Healthy |
| Unsalted Butter | Healthy | Healthy |
| Unsalted Irish Butter Blended with Vegetable Oil | Healthy | Healthy |
| Slightly Salted Irish Butter Blended with Vegetable Oil | Healthy | Healthy |
| Original Soft Spreadable | Unhealthy | Unhealthy |
| Spread the Love Olive Oil Spread | Unhealthy | Unhealthy |
| Spread the Love Canola Spread | Unhealthy | Unhealthy |
| Organic Coconut Butter | Healthy | Healthy |
| Cold Pressed & Unrefined Virgin Coconut Oil | Healthy | Healthy |
| Classic Olive Oil Spread | Unhealthy | Unhealthy |
| Light Olive Oil Spread | Unhealthy | Unhealthy |
| Pure Ghee | Healthy | Healthy |
| Spreadable Butter with Rapeseed Oil | Healthy | Healthy |
| Coconut Oil | Healthy | Healthy |
| Buttery Spread with Real Buttermilk | Unhealthy | Unhealthy |
| Unsalted Bio-Dynamic Fresh Butter | Healthy | Healthy |
| Spread Salted with Calcium | Unhealthy | Unhealthy |
| Buttery Spread with Buttermilk | Unhealthy | Unhealthy |
| Organic Vegan Spreadable | Unhealthy | Unhealthy |
| Salted Plant Based Butter with Buttermilk | Unhealthy | Unhealthy |
| Lightly Salted Plant Based Butter with Buttermilk | Unhealthy | Unhealthy |
| Organic Spreadable Salted Butter Blended with Organic Canola Oil | Healthy | Healthy |
| Buttery Spread with Probiotics | Unhealthy | Unhealthy |
| Thyme & Black Pepper Butter Infusion | Healthy | Healthy |
| Butter, plain, reduced salt (sodium < 350 mg /100 g) | Healthy | Healthy |
| Butter, plain, no added salt | Healthy | Healthy |
| Butter, spreadable, regular (~80% fat) | Healthy | Healthy |
| Butter, spreadable, reduced fat (~60% fat) | Healthy | Healthy |
| Ghee, clarified butter | Healthy | Healthy |
| Dairy blend, butter & edible oil spread (~80% fat), sodium 485 mg/100 g | Unhealthy | Unhealthy |
| Dairy blend, butter & edible oil spread (~80% fat), reduced salt (sodium 290 mg/100 g) | Unhealthy | Unhealthy |
| Dairy blend, butter & edible oil spread (70% fat), sodium 485 mg/100 g | Unhealthy | Unhealthy |
| Dairy blend, butter & edible oil spread (70% fat), reduced salt (sodium 280 mg/100 g) | Unhealthy | Unhealthy |
| Dairy blend, butter & edible oil spread, reduced fat (60% fat), sodium 400 mg/100 g | Unhealthy | Unhealthy |
| Dairy blend, butter & edible oil spread, reduced fat (60% fat) & salt (sodium 200 mg/100 g) | Unhealthy | Unhealthy |
| Dairy blend, butter & edible oil spread, reduced fat (40% fat), sodium 510 mg/100 g | Unhealthy | Unhealthy |
| Dairy blend, butter & edible oil spread, reduced fat (40% fat) & sodium 380 mg/100 g | Unhealthy | Unhealthy |
| Dairy blend, butter & edible oil spread, reduced fat (16% fat) & sodium 390 mg/100 g | Unhealthy | Unhealthy |
| Margarine spread, polyunsaturated (70% fat) | Unhealthy | Unhealthy |
| Margarine spread, polyunsaturated (70% fat), reduced salt (sodium = 280 mg/100g) | Unhealthy | Unhealthy |
| Margarine spread, polyunsaturated (70% fat), reduced salt (sodium = 340 mg/100 g), no added milk, added vitamin E | Unhealthy | Unhealthy |
| Margarine spread, polyunsaturated, reduced fat (60% fat), sodium = 790 mg/100 g | Unhealthy | Unhealthy |
| Margarine spread, polyunsaturated, reduced fat (50% fat) & salt (sodium = 360 mg/100 g) | Unhealthy | Unhealthy |
| Margarine spread, polyunsaturated, reduced fat (50% fat) & salt (sodium = 340 mg/100 g), no added milk, added vitamin E | Unhealthy | Unhealthy |
| Margarine spread, polyunsaturated, reduced fat (40% fat), no added salt or milk | Unhealthy | Unhealthy |
| Margarine spread, polyunsaturated, reduced fat (25% fat) & salt (sodium = 360 mg/100 g) | Unhealthy | Unhealthy |
| Margarine spread, polyunsaturated (60% fat), reduced salt (Na=340 mg/100 g), no added milk, added vitamin E & phytosterols | Unhealthy | Unhealthy |
| Margarine spread, monounsaturated (80% fat), rice bran oil, unfortified | Unhealthy | Unhealthy |
| Margarine spread, monounsaturated (65% fat) | Unhealthy | Unhealthy |
| Margarine spread, monounsaturated (65% fat), reduced salt (sodium = 360mg/100 g) | Unhealthy | Unhealthy |
| Margarine spread, monounsaturated (65% fat), reduced salt (sodium = 300 mg/100 g) | Unhealthy | Unhealthy |
| Margarine spread, olive oil blend (65% fat), reduced salt (sodium = 360mg/100 g) | Unhealthy | Unhealthy |
| Margarine spread, olive oil blend, (65% fat), reduced salt (sodium = 340 mg/100 g), no added milk, added vitamin E | Unhealthy | Unhealthy |
| Margarine spread, monounsaturated, reduced fat (60% fat), no added salt or milk | Unhealthy | Unhealthy |
| Margarine spread, monounsaturated, reduced fat (50% fat) | Unhealthy | Unhealthy |
| Margarine spread, monounsaturated, reduced fat (50% fat) & salt (sodium = 465 mg/100 g) | Unhealthy | Unhealthy |
| Margarine spread, monounsaturated, reduced fat (50% fat) & salt (sodium = 350 mg/100 g) | Unhealthy | Unhealthy |
| Margarine spread, monounsaturated, reduced fat (50% fat) & salt (sodium = 350 mg/100 g), no added milk | Unhealthy | Unhealthy |
| Margarine spread, monounsaturated, reduced fat (30% fat) & salt (sodium = 350 mg/100 g) | Unhealthy | Unhealthy |
| Margarine spread, olive oil blend (55% fat), reduced salt (sodium = 350mg/100 g) | Unhealthy | Unhealthy |
| Margarine, cooking | Unhealthy | Unhealthy |
| Margarine spread, polyunsaturated (65% fat), reduced salt (sodium = 360 mg/100 g), added phytosterols | Unhealthy | Unhealthy |
| Margarine spread, polyunsaturated, reduced fat (~40% fat) & salt (sodium = 360 mg/100 g), added phytosterols | Unhealthy | Unhealthy |
| Margarine spread, polyunsaturated, reduced fat (~25% fat) & salt (sodium = 360 mg/100 g), added phytosterols | Unhealthy | Unhealthy |
| Margarine spread, monounsaturated (70%), reduced salt (sodium = 350 mg/100 g), added phytosterols | Unhealthy | Unhealthy |
| Margarine spread, monounsaturated, reduced fat (45% fat) & salt (sodium = 350 mg/100 g), added phytosterols | Unhealthy | Unhealthy |
| Margarine spread, monounsaturated, reduced fat (25% fat) & salt (sodium = 350 mg/100 g), added vitamin E & phytosterols | Unhealthy | Unhealthy |
| Margarine spread, olive oil blend (50% fat), reduced salt (sodium = 360 mg/100 g), added phytosterols | Unhealthy | Unhealthy |
| Margarine spread, monounsaturated or polyunsaturated, reduced fat (<50% fat), regular salt | Unhealthy | Unhealthy |
| Dripping, beef | Healthy | Healthy |
| Lard | Healthy | Healthy |
| Suet | Healthy | Healthy |
| Shortening, commercial, animal fat (for short pastry, pie bases, tarts and flans) | Unhealthy | Unhealthy |
| Fat, solid, vegetable oil based | Healthy | Healthy |
| Oil, copha | Healthy | Healthy |
| Oil, palm | Healthy | Healthy |
| Shortening, commercial, vegetable fat (for bread, buns & yeast doughs) | Unhealthy | Unhealthy |
| Shortening, commercial, vegetable fat (for flaky pastry, pie tops, sausage rolls & danishes) | Unhealthy | Unhealthy |
| Shortening, commercial, vegetable fat (for coatings, creams, icings, confectionery & fillings) | Unhealthy | Unhealthy |
| Fat, solid, blend of animal & vegetable oils | Healthy | Healthy |
| Shortening, commercial, blend of animal & vegetable fat (for cakes, muffins, shortbreads & biscuits) | Unhealthy | Unhealthy |
| Fat, butter, dairy blend or margarine spread, not further defined | Unhealthy | Unhealthy |
| **Cooking oils** | | |
| Extra Virgin Olive Oil & Tomato Lycopene | Unhealthy | Unhealthy |
| Cold Pressed Extra Virgin Olive Oil Spray | Healthy | Healthy |
| White Truffle Oil Spray | Healthy | Healthy |
| Extra Virgin Olive Oil with Rosemary & Oregano | Healthy | Healthy |
| Cold Pressed Virgin Flaxseed Oil | Healthy | Healthy |
| Certified Organic Sweet Almond Oil | Healthy | Healthy |
| Asian Inspired Cooking Oil | Unhealthy | Unhealthy |
| Juniper Berries & Bay Leaves Infused Extra Virgin Olive Oil | Unhealthy | Unhealthy |
| Pesto Infusion Extra Virgin Olive Oil | Unhealthy | Healthy |
| Non-Stick Australian Canola Spray | Unhealthy | Healthy |
| Canola Oil Spray | Unhealthy | Healthy |
| Coconut Oil Spray | Healthy | Healthy |
| Rice Bran Oil | Healthy | Healthy |
| Vegetable Oil | Healthy | Healthy |
| Flax, Hemp Oil & Honey Omega Boost | Healthy | Healthy |
| Sunflower Oil | Healthy | Healthy |
| 100% Pure Peanut Oil | Healthy | Healthy |
| Cold Pressed Hemp Seed Oil | Healthy | Healthy |
| Olive Oil Spray Light in Taste | Healthy | Healthy |
| Seasoning Oil | Healthy | Healthy |
| Truffle Oil | Healthy | Healthy |
| Organic Avocado Oil | Healthy | Healthy |
| Vegetable Oil | Healthy | Healthy |
| Blended Vegetable Oil | Healthy | Healthy |
| Cold Pressed Macadamia Oil | Healthy | Healthy |
| 100% Pure Grape Seed Oil | Healthy | Healthy |
| Australian Lemon Pressed Extra Virgin Olive Oil | Healthy | Healthy |
| Australian Mandarin Pressed Extra Virgin Olive Oil | Healthy | Healthy |
| Fruity Taste Organic Extra Virgin Olive Oil Spray | Healthy | Healthy |
| Australian Canola Oil | Healthy | Healthy |
| Australian Peanut Oil | Healthy | Healthy |
| Ginger and Lemongrass Infused Australian Extra Virgin Olive Oil | Healthy | Healthy |
| Extra Mild Olive Oil | Healthy | Healthy |
| Sesame Oil | Healthy | Healthy |
| 100% Spanish Mellow Olive Oil | Healthy | Healthy |
| Walnut Oil | Healthy | Healthy |
| Chilli Extra Virgin Olive Oil | Unhealthy | Healthy |
| Oil, blend of polyunsaturated vegetable oils | Healthy | Healthy |
| Oil, cottonseed | Healthy | Healthy |
| Oil, grapeseed | Healthy | Healthy |
| Oil, linseed or flaxseed | Healthy | Healthy |
| Oil, maize | Healthy | Healthy |
| Oil, safflower | Healthy | Healthy |
| Oil, sesame | Healthy | Healthy |
| Oil, soybean | Healthy | Healthy |
| Oil, sunflower | Healthy | Healthy |
| Oil, almond | Healthy | Healthy |
| Oil, blend of monounsaturated vegetable oils | Healthy | Healthy |
| Oil, canola | Healthy | Healthy |
| Oil, macadamia | Healthy | Healthy |
| Oil, mustard seed | Healthy | Healthy |
| Oil, olive | Healthy | Healthy |
| Oil, peanut | Healthy | Healthy |
| Oil, rice bran | Healthy | Healthy |
| Lecithin, soy, granules | Unhealthy | Unhealthy |
| **Eggs** | | |
| Australian Extra Large Free Range Eggs | Healthy | Healthy |
| Simply Egg Whites | Healthy | Healthy |
| Egg, chicken, whole, raw | Healthy | Healthy |
| Egg, chicken, whole, baked, no added fat | Healthy | Healthy |
| Egg, chicken, whole, hard-boiled | Healthy | Healthy |
| Egg, chicken, whole, fried, no fat added | Healthy | Healthy |
| Egg, chicken, whole, poached, no added fat | Healthy | Healthy |
| Egg, chicken, white (albumen) only, raw | Healthy | Healthy |
| Egg, chicken, white (albumen) only, hard-boiled | Healthy | Healthy |
| Egg, chicken, white (albumen) only, fried, no added fat | Healthy | Healthy |
| Egg, chicken, yolk, raw | Healthy | Healthy |
| Egg, chicken, yolk, hard-boiled | Healthy | Healthy |
| Egg, chicken, yolk, fried, no fat added | Healthy | Healthy |
| Egg, chicken, whole, omega-3 polyunsaturate enriched, raw | Healthy | Healthy |
| Egg, chicken, whole, omega-3 polyunsaturate enriched, boiled | Healthy | Healthy |
| Egg, duck, whole, raw | Healthy | Healthy |
| Egg, duck, whole, boiled | Healthy | Healthy |
| Egg, quail, whole, raw | Healthy | Healthy |
| Egg, whole, preserved, cooked | Healthy | Healthy |
| Egg, emu, whole, raw | Healthy | Healthy |
| Omelette, prepared from yolk-free egg mix, fried, butter | Unhealthy | Healthy |
| **Fish and seafood products** | | |
| **Fresh or frozen seafood** | | |
| IQF Pineapple Cut Squids | Healthy | Healthy |
| Salmon with Barbeque Board | Healthy | Healthy |
| South Atlantic Extra Large Raw Prawns Tail On | Healthy | Healthy |
| Barramundi, raw | Healthy | Healthy |
| Barramundi, baked, roasted, grilled, BBQ'd or fried, no added fat | Healthy | Healthy |
| Barramundi, boiled, microwaved, steamed or poached, no added fat | Healthy | Healthy |
| Bassa (basa), raw | Healthy | Healthy |
| Bassa (basa), baked, roasted, grilled, BBQ'd or fried, no added fat | Healthy | Healthy |
| Blue grenadier (hoki), raw | Healthy | Healthy |
| Blue grenadier (hoki), baked, roasted, fried, grilled or BBQ'd, no added fat | Healthy | Healthy |
| Blue-eye trevalla, flesh, raw | Healthy | Healthy |
| Bream, raw | Healthy | Healthy |
| Bream, baked, roasted, fried, grilled or BBQ'd, no added fat | Healthy | Healthy |
| Cod, Atlantic, flesh, raw | Healthy | Healthy |
| Cod, Pacific, flesh, raw | Healthy | Healthy |
| Flathead, raw | Healthy | Healthy |
| Flathead, baked, roasted, fried, grilled or BBQ'd, no added fat | Healthy | Healthy |
| Flounder, raw | Healthy | Healthy |
| Flounder, baked, roasted, fried, grilled or BBQ'd, no added fat | Healthy | Healthy |
| Garfish, raw | Healthy | Healthy |
| Gemfish, raw | Healthy | Healthy |
| Gemfish, boiled, microwaved, steamed or poached, no added fat | Healthy | Healthy |
| Grouper, raw | Healthy | Healthy |
| Grouper, baked, roasted, fried, grilled or BBQ'd, no added fat | Healthy | Healthy |
| John dory, raw | Healthy | Healthy |
| John dory, baked, roasted, fried, grilled or BBQ'd, no added fat | Healthy | Healthy |
| Ling, raw | Healthy | Healthy |
| Ling, baked, roasted, fried, grilled or BBQ'd, no added fat | Healthy | Healthy |
| Mackerel, raw | Healthy | Healthy |
| Mackerel, baked, roasted, fried, grilled or BBQ'd, no added fat | Healthy | Healthy |
| Milkfish, raw | Healthy | Healthy |
| Morwong, raw | Healthy | Healthy |
| Mullet, raw | Healthy | Healthy |
| Mullet, boiled, microwaved, steamed or poached, with or without added fat | Healthy | Healthy |
| Mulloway, raw | Healthy | Healthy |
| Nile perch, baked, roasted, fried, grilled or BBQ'd, no added fat | Healthy | Healthy |
| Orange roughy, raw | Healthy | Healthy |
| Orange roughy, boiled, microwaved, steamed or poached, no added fat | Healthy | Healthy |
| Salmon, Atlantic, raw | Healthy | Healthy |
| Salmon, Pacific king, raw | Healthy | Healthy |
| Salmon, sashimi style, raw | Healthy | Healthy |
| Sardine, raw | Healthy | Healthy |
| Shark (flake), raw | Healthy | Healthy |
| Shark (flake), baked, roasted, fried, grilled or BBQ'd, no added fat | Healthy | Healthy |
| Silver perch, raw | Healthy | Healthy |
| Silver perch, baked, roasted, fried, grilled or BBQ'd, no added fat | Healthy | Healthy |
| Snapper, raw | Healthy | Healthy |
| Snapper, boiled, microwaved, steamed or poached, with or without added fat | Healthy | Healthy |
| Swordfish, raw | Healthy | Healthy |
| Swordfish, baked, roasted, fried, grilled or BBQ'd, no added fat | Healthy | Healthy |
| Tilapia, raw | Healthy | Healthy |
| Tilapia, boiled, microwaved, steamed or poached, no added fat | Healthy | Healthy |
| Trevally or kingfish, raw | Healthy | Healthy |
| Trevally or kingfish, cooked, no fat added | Healthy | Healthy |
| Trout, rainbow, raw | Healthy | Healthy |
| Trout, rainbow, baked, roasted, fried, grilled or BBQ'd, no added fat | Healthy | Healthy |
| Tuna, raw | Healthy | Healthy |
| Tuna, baked, roasted, fried, grilled or BBQ'd, no added fat | Healthy | Healthy |
| Tuna, sashimi style, raw | Healthy | Healthy |
| Whiting, raw | Healthy | Healthy |
| Whiting, baked, roasted, fried, grilled or BBQ'd, no added fat | Healthy | Healthy |
| Cod or hake, smoked, boiled, microwaved, steamed or poached, no added fat | Unhealthy | Unhealthy |
| Moreton bay bug or balmain bug, flesh, raw | Healthy | Healthy |
| Prawn, king or medium, raw (green) | Healthy | Healthy |
| Prawn, king or medium, flesh, baked, roasted, fried, grilled or BBQ'd, no added fat | Healthy | Healthy |
| Prawn, king or medium, flesh, boiled, microwaved, steamed or poached, no added fat | Healthy | Healthy |
| Prawn, school, flesh, boiled, microwaved, steamed or poached, no added fat | Healthy | Healthy |
| Yabby (yabbie), flesh, cooked, no added fat | Healthy | Healthy |
| Mussel, blue, boiled, microwaved, steamed or poached, no added fat | Healthy | Healthy |
| Mussel, green, boiled, microwaved, steamed or poached, no added fat | Healthy | Healthy |
| Octopus, raw | Healthy | Healthy |
| Oyster, raw | Healthy | Healthy |
| Oyster, cooked, with or without added fat | Healthy | Healthy |
| Scallop, with roe, raw | Healthy | Healthy |
| Scallop with or without roe, baked, roasted, grilled, fried or BBQ'd, no added fat | Healthy | Healthy |
| Squid or calamari, raw | Healthy | Healthy |
| Squid or calamari, baked, roasted, fried, stir-fried, grilled or BBQ'd, no added fat | Healthy | Healthy |
| Fish roe (caviar), black | Healthy | Healthy |
| Fish roe (caviar), red | Healthy | Healthy |
| Fish, eel, raw | Healthy | Healthy |
| Fish, eel, baked, roasted, fried, stir-fried, grilled or BBQ'd, no added fat | Healthy | Healthy |
| Stingray, wild caught, flesh, baked, roasted, fried, grilled or BBQ'd, no added fat | Healthy | Healthy |
| Pipi, wild harvested, cooked | Healthy | Healthy |
| **Processed fish and seafood** | | |
| Classic Tuna Bruschetta | Unhealthy | Healthy |
| Tuna Bruschetta with Mayonnaise & Herbs | Unhealthy | Healthy |
| Tuna Bruschetta with Dill & Pickle | Unhealthy | Unhealthy |
| Tuna Bruschetta with Water Chestnut & Ginger | Unhealthy | Unhealthy |
| Orange & Black Pepper Smoked Salmon | Unhealthy | Unhealthy |
| Smoked Tuna | Unhealthy | Healthy |
| Crumbed Fish Fillets | Unhealthy | Unhealthy |
| Whisky Cured Tasmanian Cold Smoked Salmon | Unhealthy | Unhealthy |
| Salmon with Sunblush Tomato Dressing | Healthy | Healthy |
| Soy, Ginger & Garlic Flavoured Salmon | Unhealthy | Unhealthy |
| Mackerel Fillets in Spicy Tomato Sauce | Unhealthy | Unhealthy |
| Beetroot & Dill Cured Salmon with Gin | Unhealthy | Unhealthy |
| Tuna in Springwater | Healthy | Healthy |
| Tempura Battered Fish Fillets | Unhealthy | Unhealthy |
| Pole & Line Caught Yellowfin Tuna in Lemon & Pepper | Unhealthy | Unhealthy |
| Tasmanian Salmon Gravalax | Unhealthy | Unhealthy |
| Responsibly Caught Tuna in Olive Oil | Healthy | Healthy |
| Cantabrico Anchovies in Olive Oil | Unhealthy | Unhealthy |
| Wild Scottish Sardines Deliciously Infused with Rosemary and Sea Salt | Unhealthy | Unhealthy |
| Roe Off Sea Scallops | Healthy | Healthy |
| Basa Fillets | Healthy | Healthy |
| Southern Style Deep Sea Fish Fillets | Unhealthy | Unhealthy |
| Garlic Prawns with Chilli & Parsley | Healthy | Healthy |
| Extra Virgin Olive Oil Blend & Pink Salt Tuna | Unhealthy | Unhealthy |
| Prawns with a Classic Cocktail Sauce | Unhealthy | Unhealthy |
| New Zealand Squid with a Classic Salt & Pepper Coating | Unhealthy | Unhealthy |
| Traditionally Hot Smoked Mediterranean Pepper Atlantic Salmon | Unhealthy | Unhealthy |
| Coconut Prawns | Unhealthy | Unhealthy |
| Hot Smoked Cracked Peppercorn Tassie Salmon | Unhealthy | Unhealthy |
| Japanese BBQ Sauce Marinated Tassie Salmon | Unhealthy | Unhealthy |
| Original Wild Caught Crumbed White Fish | Unhealthy | Unhealthy |
| Tuna & Beans | Unhealthy | Unhealthy |
| Natural Tasmanian Wood Smoked Mussels | Unhealthy | Unhealthy |
| Fresh Tassie Salmon with Lemon & Herb Crumb | Unhealthy | Unhealthy |
| Salmon with Asian Style Marinade | Unhealthy | Unhealthy |
| Salmon with American Style BBQ Marinade | Unhealthy | Unhealthy |
| Responsibly Fished Mexican Style Chipotle Mayonnaise Tuna | Unhealthy | Unhealthy |
| Cheese Flavored Fish Sausage | Unhealthy | Unhealthy |
| Smoked Trout Thinly Sliced | Healthy | Healthy |
| Wood-Smoked Wild Brisling Sardines with Chilli | Unhealthy | Unhealthy |
| Roe Off Scallops | Unhealthy | Healthy |
| Raw Australian Banana Prawns | Healthy | Healthy |
| Bream, boiled, microwaved, steamed or poached, with or without added fat | Healthy | Healthy |
| Flathead, boiled, microwaved, steamed or poached, with or without added fat | Healthy | Healthy |
| Morwong, boiled, microwaved, steamed or poached, with or without added fat | Healthy | Healthy |
| Mulloway, boiled, microwaved, steamed or poached, with or without added fat | Healthy | Healthy |
| Shark (flake), boiled, microwaved, steamed or poached, with or without added fat | Healthy | Healthy |
| Whiting, boiled, microwaved, steamed or poached, with or without added fat | Healthy | Healthy |
| Cod, smoked, raw | Unhealthy | Unhealthy |
| Salmon, smoked, sliced | Unhealthy | Unhealthy |
| Smoked fish (including eel & trout), smoked | Unhealthy | Unhealthy |
| Crab, flesh, purchased steamed, poached or boiled | Healthy | Healthy |
| Lobster or crayfish, flesh, purchased steamed, poached or boiled, no added fat | Healthy | Healthy |
| Moreton bay bug, cooked, with or without fat | Healthy | Healthy |
| Octopus, cooked, with or without added fat | Healthy | Healthy |
| Scallop with or without roe, boiled, microwaved, steamed or poached, with or without added fat | Healthy | Healthy |
| Anchovy, canned | Unhealthy | Unhealthy |
| Herring, Atlantic, pickled | Unhealthy | Unhealthy |
| Mackerel, canned | Unhealthy | Unhealthy |
| Salmon, pink, unflavoured, canned in brine, drained | Healthy | Healthy |
| Salmon, pink, unflavoured, canned in water, drained | Healthy | Healthy |
| Salmon, red, unflavoured, canned in brine, drained | Healthy | Healthy |
| Salmon, red, unflavoured, canned in water, drained | Healthy | Healthy |
| Salmon, unflavoured, canned in brine, drained | Healthy | Healthy |
| Salmon, unflavoured, canned in oil | Healthy | Healthy |
| Salmon, unflavoured, canned in water, drained | Healthy | Healthy |
| Salmon, unflavoured, canned, drained | Healthy | Healthy |
| Salmon, flavoured, canned, drained | Unhealthy | Unhealthy |
| Salmon, canned, drained, not further defined | Healthy | Healthy |
| Sardine, canned in oil, undrained | Healthy | Healthy |
| Sardine, canned in oil, drained | Unhealthy | Unhealthy |
| Sardine, canned in tomato sauce, undrained | Healthy | Healthy |
| Sardine, canned in water, drained | Healthy | Healthy |
| Tuna, unflavoured, canned in brine, drained | Healthy | Healthy |
| Tuna, unflavoured, canned in vegetable oil, drained | Healthy | Healthy |
| Tuna, unflavoured, canned in water, drained | Healthy | Healthy |
| Tuna, flavoured, canned, drained | Healthy | Healthy |
| Tuna, canned, not further defined | Healthy | Healthy |
| Crabmeat, canned in brine, undrained | Unhealthy | Unhealthy |
| Mussel, smoked, canned in oil, drained | Unhealthy | Unhealthy |
| Oyster, smoked, canned in oil, drained | Healthy | Healthy |
| Blue grenadier (hoki), coated, packaged frozen, baked, roasted, fried, grilled or BBQ'd, with or without added fat | Unhealthy | Unhealthy |
| Cod or hake, coated, packaged frozen, baked, roasted, fried, grilled or BBQ'd, with or without added fat | Unhealthy | Unhealthy |
| Shark (flake), coated, packaged frozen, baked, roasted, fried, grilled or BBQ'd, with or without added fat | Unhealthy | Unhealthy |
| Snapper, baked, roasted, fried, grilled or BBQ'd, no added fat | Healthy | Healthy |
| Whiting, coated, packaged frozen, baked, roasted, fried, grilled, or BBQ'd, with or without added fat | Unhealthy | Unhealthy |
| Fish, white flesh, battered, packaged frozen, baked, roasted, fried, grilled or BBQ'd, with or without added fat | Unhealthy | Unhealthy |
| Fish, white flesh, crumbed, packaged frozen, baked, roasted, fried, grilled or BBQ'd, with or without added fat | Unhealthy | Unhealthy |
| Fish finger, crumbed, packaged frozen, raw | Unhealthy | Healthy |
| Fish finger, crumbed, packaged frozen, baked, roasted, fried or grilled, no added fat | Unhealthy | Healthy |
| Fish finger, crumbed, packaged frozen, baked, roasted, fried, grilled or BBQ'd, fat not further defined | Unhealthy | Healthy |
| Prawn, coated, packaged frozen, baked, roasted, fried, grilled or BBQ'd, fat not further defined | Unhealthy | Unhealthy |
| Prawn, coated, takeaway outlet, deep fried | Unhealthy | Unhealthy |
| Squid or calamari, crumbed, takeaway outlet, deep fried | Unhealthy | Unhealthy |
| Squid or calamari, coated, packaged frozen, baked, roasted, fried, grilled or BBQ'd, with or without added fat | Unhealthy | Unhealthy |
| Cod, Atlantic, dried, salted | Unhealthy | Unhealthy |
| Fish ball, Asian style, purchased cooked | Unhealthy | Unhealthy |
| Fish ball, packaged frozen, boiled, steamed, poached, microwaved, no fat added | Unhealthy | Unhealthy |
| Fish patty or cake, packaged frozen, baked, roasted, fried, grilled or BBQ'd, fat not further defined | Unhealthy | Unhealthy |
| Fish patty or cake, takeaway outlet, deep fried | Unhealthy | Unhealthy |
| Fish paste or spread | Unhealthy | Unhealthy |
| Paste, shrimp | Unhealthy | Unhealthy |
| Seafood or fish stick (surimi), packaged frozen, raw | Unhealthy | Unhealthy |
| Seafood or fish stick (surimi), packaged frozen, fried, peanut oil | Unhealthy | Unhealthy |
| Seafood or fish stick (surimi), packaged frozen, boiled, microwaved, steamed or poached, no added fat | Unhealthy | Unhealthy |
| Seafood or fish stick (surimi), coated, takeaway outlet, deep fried | Unhealthy | Unhealthy |
| **Fruit and vegetables** | | |
| **Fruits** | | |
| Blueberries in Syrup | Healthy | Healthy |
| South African Peach Slices in Syrup | Healthy | Healthy |
| Tasmanian Crunchy Dried Apple Wedges Infused with Blackcurrant | Unhealthy | Unhealthy |
| Banana, Apple, Kiwi, Spinach, Broccoli & Oat Breakfast Squeezie | Healthy | Healthy |
| Spanish Stuffed Green Olives | Unhealthy | Unhealthy |
| Mediterranean Chilli Olives | Unhealthy | Unhealthy |
| Organic Mango Coconut Vitality Snack | Unhealthy | Unhealthy |
| Whole Raspberry | Healthy | Healthy |
| Super Booster Smoothie Mix | Healthy | Healthy |
| Cranberries, Flame Raisins & Blueberries | Healthy | Healthy |
| Lychee Whole | Unhealthy | Unhealthy |
| Australian Peaches with Blood Orange Juice | Unhealthy | Unhealthy |
| Mixed Peel | Unhealthy | Unhealthy |
| Apple Fruit Puree | Unhealthy | Healthy |
| Peach Halves in Syrup | Unhealthy | Healthy |
| Diced Paw Paw | Unhealthy | Unhealthy |
| Sliced Pineapple in Light Syrup | Healthy | Healthy |
| Preserved Lemons | Unhealthy | Unhealthy |
| Strawberry | Healthy | Healthy |
| Aussie Diced Pears in Juice | Healthy | Healthy |
| Ligurian Olives | Unhealthy | Unhealthy |
| Apricot Indulgence | Unhealthy | Unhealthy |
| Watermelon | Healthy | Healthy |
| Passionfruit Pulp in Syrup | Unhealthy | Unhealthy |
| Diced Peaches in Pear, Mango & Apricot Puree with Almond, Coconut & Chia Crunch | Unhealthy | Unhealthy |
| Avocado | Healthy | Healthy |
| Refreshing Fruit Platter | Healthy | Healthy |
| Frozen Fruit Raspberries | Healthy | Healthy |
| Fruit Mix | Healthy | Healthy |
| Blueberries | Healthy | Healthy |
| Organic Medjoul Dates | Healthy | Healthy |
| Mixed Fruit | Unhealthy | Unhealthy |
| Cinnamon Apple Chips | Unhealthy | Healthy |
| Mixed Halves with Basil & Extra Virgin Olive Oil | Unhealthy | Unhealthy |
| Smoothie Mix Supercharge | Healthy | Healthy |
| Apple Pouches | Healthy | Healthy |
| Ready-Made Pink Dragon Fruit & Raspberry Bowl | Unhealthy | Unhealthy |
| Pitted Morello Cherries | Healthy | Healthy |
| 100% Natural Tasmanian Freeze-Dried Apple Wedges | Healthy | Healthy |
| Ready-Made Tropical Acai Bowl | Unhealthy | Unhealthy |
| Apple & Berry Puree Enriched with Chia | Unhealthy | Unhealthy |
| Apple & Strawberry Purée Pouches | Unhealthy | Unhealthy |
| Apricot Chia Pot | Unhealthy | Unhealthy |
| Capers in Vinegar | Unhealthy | Unhealthy |
| Pitted Cherries | Healthy | Healthy |
| Mixed Olives Marinated in Orange & Fennel | Unhealthy | Unhealthy |
| Australian Mixed Fruit | Healthy | Healthy |
| Passion Mango Nutritious Frozen Smoothie Pack | Healthy | Healthy |
| Australian Sultanas | Healthy | Healthy |
| Frozen Strawberries | Healthy | Healthy |
| Frozen Raspberries | Healthy | Healthy |
| Peaches in Juice | Unhealthy | Healthy |
| Mixed Fruit with Real Glacé Cherries | Unhealthy | Unhealthy |
| Yellow Peach Roll | Healthy | Healthy |
| Apple and Peach Puree | Unhealthy | Unhealthy |
| Aussie Diced Two Fruits in Juice | Healthy | Healthy |
| Frozen Fresh Lemon Slices | Healthy | Healthy |
| PJ Masks Apple, Strawberry, Raspberry & Blueberry Fruit Flavoured Snacks | Unhealthy | Unhealthy |
| Strawberry Fruit Twists | Unhealthy | Unhealthy |
| Morello Pitted Cherries | Healthy | Healthy |
| Mandarin Whole Segments | Unhealthy | Healthy |
| Goji Berries | Healthy | Healthy |
| Salted Capers | Healthy | Healthy |
| Sun Dried Apricot | Healthy | Healthy |
| Fruit Medley Diced Fruits | Healthy | Healthy |
| Musang King Malaysian Durian Pulp | Healthy | Healthy |
| Freeze-Dried Tasmanian Cherries | Healthy | Healthy |
| Pitted Dates | Healthy | Healthy |
| Pomegranate | Healthy | Healthy |
| Organic Raisins | Healthy | Healthy |
| Tamarind Block | Healthy | Healthy |
| Coconut Chia with Mango & Passionfruit | Unhealthy | Healthy |
| Mango Slices in Syrup | Healthy | Healthy |
| Apple & Banana Dried Fruit Bars | Healthy | Healthy |
| Frozen Seedless Crimson Grapes | Healthy | Healthy |
| 100% Australian Organic Apple & Strawberry Fruit Puree | Healthy | Healthy |
| 100% Australian Organic Apple & Watermelon Fruit Puree | Healthy | Healthy |
| Freeze Dried Strawberries | Unhealthy | Unhealthy |
| Strawberry Fruit Watches | Unhealthy | Unhealthy |
| Frozen Pink Dragonfruit Chunks | Healthy | Healthy |
| Melon Mix | Healthy | Healthy |
| Rosemary & Chili Australian Seasonal Olives | Unhealthy | Unhealthy |
| Apricot, Flame Raisins, Apple, Peach & Carrot Snack Packs | Healthy | Healthy |
| Fruit & Oats with Blueberry and Quinoa Breakfast Pouch | Unhealthy | Healthy |
| Mango | Healthy | Healthy |
| Young Jackfruit | Unhealthy | Unhealthy |
| Freeze-Dried Apple Wedges Infused with Raspberry | Unhealthy | Unhealthy |
| Australian Sultanas | Healthy | Healthy |
| Smokey Grilled Olives | Unhealthy | Unhealthy |
| Mixed Berry Flavoured Snacks | Unhealthy | Unhealthy |
| Green Olive Halves with Garlic & Thyme | Unhealthy | Unhealthy |
| Fruit, wild harvested, raw | Healthy | Healthy |
| Saltbush, ruby, fruit | Healthy | Healthy |
| Apple, bonza, unpeeled, raw | Healthy | Healthy |
| Apple, fuji, unpeeled, raw | Healthy | Healthy |
| Apple, golden delicious, unpeeled, raw | Healthy | Healthy |
| Apple, granny-smith, unpeeled, raw | Healthy | Healthy |
| Apple, granny-smith, unpeeled, baked, no added fat | Healthy | Healthy |
| Apple, jonathon, unpeeled, raw | Healthy | Healthy |
| Apple, pink lady, unpeeled, raw | Healthy | Healthy |
| Apple, red delicious, unpeeled, raw | Healthy | Healthy |
| Apple, royal gala, unpeeled, raw | Healthy | Healthy |
| Apple, green skin, peeled, raw | Healthy | Healthy |
| Apple, green skin, unpeeled, raw | Healthy | Healthy |
| Apple, red skin, peeled, raw | Healthy | Healthy |
| Apple, red skin, unpeeled, raw | Healthy | Healthy |
| Apple, baked, no added fat | Healthy | Healthy |
| Apple, canned or puree | Healthy | Healthy |
| Pear, brown, peeled, raw | Healthy | Healthy |
| Pear, brown, unpeeled, raw | Healthy | Healthy |
| Pear, green, peeled, raw | Healthy | Healthy |
| Pear, green, unpeeled, raw | Healthy | Healthy |
| Pear, nashi, peeled or unpeeled, raw | Healthy | Healthy |
| Pear, packhams triumph, unpeeled, raw | Healthy | Healthy |
| Pear, william bartlett, unpeeled, raw | Healthy | Healthy |
| Pear, canned in pear juice | Healthy | Healthy |
| Pear, canned in pear juice, drained | Healthy | Healthy |
| Pear, canned in pear juice, juice only | Healthy | Healthy |
| Pear, canned in light syrup | Unhealthy | Healthy |
| Pear, canned in light syrup, drained | Unhealthy | Healthy |
| Pear, canned in light syrup, syrup only | Unhealthy | Unhealthy |
| Pear, canned in syrup | Healthy | Healthy |
| Pear, canned in syrup, drained | Healthy | Healthy |
| Pear, canned in sugar syrup, syrup only | Unhealthy | Unhealthy |
| Pear, canned in intense sweetened liquid | Unhealthy | Unhealthy |
| Pear, canned in intense sweetened liquid, drained | Unhealthy | Unhealthy |
| Pear, canned in intense sweetened liquid, liquid only | Unhealthy | Unhealthy |
| Pear, canned, not further defined | Healthy | Healthy |
| Loquat, peeled, raw | Healthy | Healthy |
| Quince, peeled, cooked, sweetened | Healthy | Healthy |
| Blackberry, raw | Healthy | Healthy |
| Blackberry, purchased frozen | Healthy | Healthy |
| Blueberry, raw | Healthy | Healthy |
| Blueberry, purchased frozen | Healthy | Healthy |
| Cranberry, raw | Healthy | Healthy |
| Mulberry, raw | Healthy | Healthy |
| Raspberry, raw | Healthy | Healthy |
| Raspberry, purchased frozen | Healthy | Healthy |
| Strawberry, raw | Healthy | Healthy |
| Strawberry, purchased frozen | Healthy | Healthy |
| Blueberry, canned in syrup | Unhealthy | Unhealthy |
| Blueberry, canned in syrup, drained | Healthy | Healthy |
| Blueberry, canned in syrup, syrup only | Unhealthy | Unhealthy |
| Raspberry, canned in syrup | Unhealthy | Unhealthy |
| Raspberry, canned in syrup, drained | Unhealthy | Unhealthy |
| Raspberry, canned in syrup, syrup only | Unhealthy | Unhealthy |
| Strawberry, canned in syrup | Unhealthy | Unhealthy |
| Strawberry, canned in syrup, drained | Unhealthy | Unhealthy |
| Strawberry, canned in syrup, syrup only | Unhealthy | Unhealthy |
| Orange, navel (all varieties), peeled, raw | Healthy | Healthy |
| Orange, valencia, peeled, raw | Healthy | Healthy |
| Orange, peeled, stewed, no added fat | Healthy | Healthy |
| Lemon, peeled, raw | Healthy | Healthy |
| Lemon peel, raw | Healthy | Healthy |
| Lime, peeled, raw | Healthy | Healthy |
| Lime, native, fruit | Healthy | Healthy |
| Cumquat (kumquat), raw | Healthy | Healthy |
| Grapefruit, peeled, raw | Healthy | Healthy |
| Mandarin, peeled, raw | Healthy | Healthy |
| Tangelo, peeled, raw | Healthy | Healthy |
| Tangerine or tangor, peeled, raw | Healthy | Healthy |
| Mandarin, canned in natural juice | Healthy | Healthy |
| Mandarin, canned in syrup | Healthy | Healthy |
| Mandarin, canned in syrup, drained | Healthy | Healthy |
| Mandarin, canned in syrup, syrup only | Unhealthy | Unhealthy |
| Nectarine, yellow, peeled, raw | Healthy | Healthy |
| Nectarine, yellow, unpeeled, raw | Healthy | Healthy |
| Nectarine, white, peeled or unpeeled, raw | Healthy | Healthy |
| Peach, yellow, peeled, raw | Healthy | Healthy |
| Peach, yellow, unpeeled, raw | Healthy | Healthy |
| Peach, white, peeled, raw | Healthy | Healthy |
| Peach, white, unpeeled, raw | Healthy | Healthy |
| Peach, canned in pear juice | Healthy | Healthy |
| Peach, canned in pear juice, drained | Healthy | Healthy |
| Peach, canned in pear juice, juice only | Healthy | Healthy |
| Peach, canned in light syrup | Unhealthy | Healthy |
| Peach, canned in light syrup, drained | Unhealthy | Healthy |
| Peach, canned in light syrup, syrup only | Unhealthy | Unhealthy |
| Peach, canned in syrup | Healthy | Healthy |
| Peach, canned in syrup, drained | Healthy | Healthy |
| Peach, canned in syrup, syrup only | Unhealthy | Unhealthy |
| Peach, canned in intense sweetened liquid | Unhealthy | Unhealthy |
| Peach, canned in intense sweetened liquid, drained | Unhealthy | Unhealthy |
| Peach, canned in intense sweetened liquid, liquid only | Unhealthy | Unhealthy |
| Peach, canned, not further defined | Healthy | Healthy |
| Apricot, raw | Healthy | Healthy |
| Cherry, raw | Healthy | Healthy |
| Plum, unpeeled, raw | Healthy | Healthy |
| Apricot, canned in pear juice | Healthy | Healthy |
| Apricot, canned in pear juice, drained | Healthy | Healthy |
| Apricot, canned in pear juice, juice only | Healthy | Healthy |
| Apricot, canned in light syrup | Unhealthy | Healthy |
| Apricot, canned in light syrup, drained | Unhealthy | Healthy |
| Apricot, canned in light syrup, syrup only | Unhealthy | Unhealthy |
| Apricot, canned in syrup | Healthy | Healthy |
| Apricot, canned in syrup, drained | Healthy | Healthy |
| Apricot, canned in syrup, syrup only | Unhealthy | Unhealthy |
| Apricot, canned in intense sweetened liquid | Unhealthy | Unhealthy |
| Apricot, canned in intense sweetened liquid, drained | Unhealthy | Unhealthy |
| Apricot, canned in intense sweetened liquid, liquid only | Unhealthy | Unhealthy |
| Cherry, black, canned in syrup | Unhealthy | Unhealthy |
| Cherry, black, canned in syrup, drained | Healthy | Healthy |
| Cherry, black, canned in syrup, syrup only | Unhealthy | Unhealthy |
| Plum, dark, canned in syrup | Unhealthy | Unhealthy |
| Plum, dark, canned in syrup, drained | Unhealthy | Unhealthy |
| Plum, dark, canned in syrup, syrup only | Unhealthy | Unhealthy |
| Banana, cavendish, peeled, raw | Healthy | Healthy |
| Banana, lady finger or sugar, peeled, raw | Healthy | Healthy |
| Pineapple (cayenne), peeled, raw | Healthy | Healthy |
| Babaco, peeled, raw | Healthy | Healthy |
| Cheese fruit, raw | Healthy | Healthy |
| Fig, fresh, peeled or unpeeled, raw | Healthy | Healthy |
| Fig, fresh, peeled or unpeeled, baked | Healthy | Healthy |
| Persimmon, peeled, raw | Healthy | Healthy |
| Tamarillo, peeled, raw | Healthy | Healthy |
| Wax jambu, raw | Healthy | Healthy |
| Custard apple, african pride, peeled, raw | Healthy | Healthy |
| Feijoa, raw | Healthy | Healthy |
| Guava, hawaiian, raw | Healthy | Healthy |
| Jackfruit, peeled, raw | Healthy | Healthy |
| Lychee, peeled, raw | Healthy | Healthy |
| Mango, peeled, raw | Healthy | Healthy |
| Passionfruit, raw | Healthy | Healthy |
| Pawpaw (papaya), orange flesh, peeled, raw | Healthy | Healthy |
| Pomegranate, peeled, raw | Healthy | Healthy |
| Prickly pear, peeled, raw | Healthy | Healthy |
| Rambutan, raw | Healthy | Healthy |
| Tropical fruit, other, raw | Healthy | Healthy |
| Lychee, peeled, canned | Healthy | Healthy |
| Mango, pulped, canned | Healthy | Healthy |
| Passionfruit, pulp, canned | Healthy | Healthy |
| Pineapple, fresh, cooked in light syrup, drained | Unhealthy | Healthy |
| Pineapple, canned in pineapple juice | Healthy | Healthy |
| Pineapple, canned in pineapple juice, drained | Healthy | Healthy |
| Pineapple, canned in pineapple juice, juice only | Unhealthy | Unhealthy |
| Pineapple, canned in syrup | Unhealthy | Unhealthy |
| Pineapple, canned in syrup, drained | Unhealthy | Unhealthy |
| Pineapple, canned in syrup, syrup only | Unhealthy | Unhealthy |
| Grape, black muscatel, raw | Healthy | Healthy |
| Grape, black sultana, raw | Healthy | Healthy |
| Grape, cornichon, raw | Healthy | Healthy |
| Grape, red globe, raw | Healthy | Healthy |
| Grape, thompson seedless or sultana, raw | Healthy | Healthy |
| Grape, waltham cross, raw | Healthy | Healthy |
| Kiwifruit, gold, peeled or unpeeled, raw | Healthy | Healthy |
| Kiwifruit, green (hayward), peeled, raw | Healthy | Healthy |
| Kiwifruit, green (hayward), unpeeled, raw | Healthy | Healthy |
| Melon, honey dew, white skin, peeled, raw | Healthy | Healthy |
| Melon, honey dew, yellow skin, peeled, raw | Healthy | Healthy |
| Melon, rockmelon (cantaloupe), peeled, raw | Healthy | Healthy |
| Melon, watermelon, peeled, raw | Healthy | Healthy |
| Pepino, peeled, raw | Healthy | Healthy |
| Rhubarb, stalk, raw | Healthy | Healthy |
| Plum, davidson (native), flesh | Healthy | Healthy |
| Quandong, fruit, flesh | Healthy | Healthy |
| Fruit salad, tropical, canned, drained or undrained | Healthy | Healthy |
| Fruit salad, canned in pear juice | Healthy | Healthy |
| Fruit salad, canned in pear juice, drained | Healthy | Healthy |
| Fruit salad, canned in pear juice, juice only | Healthy | Healthy |
| Fruit salad, canned in pineapple juice | Healthy | Healthy |
| Fruit salad, canned in pineapple juice, drained | Healthy | Healthy |
| Fruit salad, canned in pineapple juice, juice only | Unhealthy | Unhealthy |
| Fruit salad, canned in heavy syrup | Unhealthy | Unhealthy |
| Fruit salad, canned in heavy syrup, drained | Unhealthy | Unhealthy |
| Fruit salad, canned in heavy syrup, syrup only | Unhealthy | Unhealthy |
| Fruit salad, canned in syrup | Healthy | Healthy |
| Fruit salad, canned in syrup, drained | Healthy | Healthy |
| Fruit salad, canned in syrup, syrup only | Unhealthy | Unhealthy |
| Fruit salad, canned, not further defined | Healthy | Healthy |
| Mixed fruit, pear & peach, canned in pear juice, drained or undrained | Healthy | Healthy |
| Currant, dried | Healthy | Healthy |
| Raisin | Healthy | Healthy |
| Sultana | Healthy | Healthy |
| Apple, dried | Healthy | Healthy |
| Apricot, dried | Healthy | Healthy |
| Banana chip | Unhealthy | Unhealthy |
| Cherry, dried | Healthy | Healthy |
| Cranberry, dried, sweetened | Unhealthy | Unhealthy |
| Date, dried | Healthy | Healthy |
| Fig, dried | Healthy | Healthy |
| Goji berry, dried | Healthy | Healthy |
| Mixed dried fruit | Healthy | Healthy |
| Prune (dried plum) | Healthy | Healthy |
| Cherry, glace or maraschino | Unhealthy | Unhealthy |
| Lemon, preserved | Unhealthy | Unhealthy |
| Plum, salted | Unhealthy | Unhealthy |
| Tamarind, paste, pure, raw | Healthy | Healthy |
| Capers, pickled, canned, drained | Unhealthy | Unhealthy |
| Olive, green or black, drained | Unhealthy | Unhealthy |
| Olive, green, pimento stuffed, drained | Unhealthy | Unhealthy |
| **Herbs and spices** | | |
| Harissa Spice Mix | Unhealthy | Unhealthy |
| Biryani Spice Mix | Unhealthy | Unhealthy |
| Chicken All-Natural Spice Rub | Unhealthy | Unhealthy |
| Chilli & Garlic | Unhealthy | Unhealthy |
| Thai Seven Spice | Unhealthy | Unhealthy |
| Spicy Curry | Unhealthy | Unhealthy |
| Malay Chicken Biryani Recipe & Seasoning Mix | Unhealthy | Unhealthy |
| Moroccan Seasoning | Unhealthy | Unhealthy |
| Spicy Seasoned Salt | Unhealthy | Unhealthy |
| Garlic Salt | Unhealthy | Unhealthy |
| Cold Blended Parsley Paste | Unhealthy | Unhealthy |
| Coriander Cubes | Healthy | Healthy |
| Chilli Blend | Unhealthy | Unhealthy |
| Chipotle Seasoning | Unhealthy | Unhealthy |
| Peri-Peri Salt | Unhealthy | Unhealthy |
| All Purpose Seasoned Salt | Unhealthy | Unhealthy |
| All Purpose Seasoning | Unhealthy | Unhealthy |
| Steak Spice Rub with Garlic, Mustard Seeds and Black Peppercorns | Unhealthy | Unhealthy |
| Butter Chicken Batch-Crafted Spice Mix | Unhealthy | Unhealthy |
| Rogan Josh Batch-Crafted Spice Mix | Unhealthy | Unhealthy |
| Habanero Seasoning Mix | Unhealthy | Unhealthy |
| Organic 24 Herbs & Spices Seasoning Sprinkle | Healthy | Healthy |
| Sriracha Salt Flavour Shaker Fries Seasoning | Unhealthy | Unhealthy |
| Basil, green, raw | Healthy | Healthy |
| Chives, raw | Healthy | Healthy |
| Coriander, fresh, leaves & stems | Healthy | Healthy |
| Dill, raw | Healthy | Healthy |
| Flower, rosella (native) | Healthy | Healthy |
| Mint, raw | Healthy | Healthy |
| Parsley, continental, raw | Healthy | Healthy |
| Parsley, curly, raw | Healthy | Healthy |
| Rosemary, raw | Healthy | Healthy |
| **Jams and marmalades** | | |
| Homestyle Strawberry Conserve | Unhealthy | Unhealthy |
| Breakfast Homestyle Marmalade | Unhealthy | Unhealthy |
| Homestyle Plum Conserve | Unhealthy | Unhealthy |
| Cherry Preserve | Unhealthy | Unhealthy |
| Peach and Mango Preserve | Unhealthy | Unhealthy |
| Premium Black Cherry Fruit Spread | Unhealthy | Unhealthy |
| Red Currant Jelly | Unhealthy | Unhealthy |
| Seville Orange Marmalade | Unhealthy | Unhealthy |
| 70% Fruit Raspberry Fruit Jam | Unhealthy | Unhealthy |
| 70% Fruit Plum Jam | Unhealthy | Unhealthy |
| 70% Fruit Blueberry Jam | Unhealthy | Unhealthy |
| Fruits of the Forest Conserve | Unhealthy | Unhealthy |
| Quandong with Apple Fruit Paste | Unhealthy | Unhealthy |
| Apricot Jam | Unhealthy | Unhealthy |
| Strawberry Fruit Spread | Unhealthy | Unhealthy |
| Breakfast Marmalade | Unhealthy | Unhealthy |
| Original Ginger Marmalade | Unhealthy | Unhealthy |
| Seville Orange Marmalade | Unhealthy | Unhealthy |
| Peach & Raspberry Jam | Unhealthy | Unhealthy |
| Blackcurrant Fruit Paste | Unhealthy | Unhealthy |
| Apple Fruit Paste | Unhealthy | Unhealthy |
| Mountain Berry Jam | Unhealthy | Unhealthy |
| Plum Paste | Unhealthy | Unhealthy |
| Traditional Slow Cooked Raspberry Jam | Unhealthy | Unhealthy |
| Strawberry Preserve Extra Jam | Unhealthy | Unhealthy |
| Apricot, Peach & Vanilla Jam | Unhealthy | Unhealthy |
| Apricot Jam | Unhealthy | Unhealthy |
| Australian Blueberry Jam | Unhealthy | Unhealthy |
| Australian Raspberry Jam | Unhealthy | Unhealthy |
| Strawberry & Cardamom Jam | Unhealthy | Unhealthy |
| Raspberry & Blackberry Conserve | Unhealthy | Unhealthy |
| Raspberry Jam | Unhealthy | Unhealthy |
| Damson Jam | Unhealthy | Unhealthy |
| Sweet Fig Jam | Unhealthy | Unhealthy |
| Jam, apricot, regular | Unhealthy | Unhealthy |
| Jam, blackberry, regular | Unhealthy | Unhealthy |
| Jam, fig, regular | Unhealthy | Unhealthy |
| Jam, mixed berry, regular | Unhealthy | Unhealthy |
| Jam, plum, regular | Unhealthy | Unhealthy |
| Jam, raspberry, regular | Unhealthy | Unhealthy |
| Jam, strawberry, regular | Unhealthy | Unhealthy |
| Jam, other fruit, regular | Unhealthy | Unhealthy |
| Jam, not further defined | Unhealthy | Unhealthy |
| Marmalade, ginger, regular | Unhealthy | Unhealthy |
| Marmalade, cumquat (kumquat), regular | Unhealthy | Unhealthy |
| Marmalade, lime or lemon, regular | Unhealthy | Unhealthy |
| Marmalade, orange, regular | Unhealthy | Unhealthy |
| Marmalade, regular, not further defined | Unhealthy | Unhealthy |
| Paste, quince | Unhealthy | Unhealthy |
| Jam, all flavours, reduced sugar | Unhealthy | Unhealthy |
| Jam, all flavours, no added sugar (100% fruit) | Unhealthy | Unhealthy |
| Jam, all flavours, intense sweetened | Unhealthy | Unhealthy |
| Marmalade, ginger, reduced sugar | Unhealthy | Unhealthy |
| Marmalade, lime or lemon, reduced sugar | Unhealthy | Unhealthy |
| Marmalade, orange, reduced sugar | Unhealthy | Unhealthy |
| Marmalade, orange, no added sugar (100% fruit) | Unhealthy | Unhealthy |
| Sauce, sweet, mixed berry coulis | Unhealthy | Unhealthy |
| **Nuts and seeds** | | |
| Wasabi and Soy Sauce Almonds | Unhealthy | Unhealthy |
| Chia & Berry Bar | Unhealthy | Healthy |
| Chia & Cacao Bar | Unhealthy | Healthy |
| Chia & Almond Bar | Unhealthy | Unhealthy |
| Greek Style Yogurt & Blueberry Roasted Nut Bars | Unhealthy | Unhealthy |
| Walnut & Flax Seed Bar | Unhealthy | Unhealthy |
| Caramel Coated Nuts | Unhealthy | Unhealthy |
| English Toffee Diced Almonds & Cashews | Unhealthy | Unhealthy |
| Bio-Organic Coconut Chips | Healthy | Healthy |
| Cashew Macadamia Munch | Healthy | Healthy |
| Original Coconut Chunks | Unhealthy | Unhealthy |
| Toffee Apple Flavoured Almonds | Unhealthy | Unhealthy |
| Australian Grown Flaked Almonds | Healthy | Healthy |
| Premium Dry Roasted Mixed Nuts | Healthy | Healthy |
| 100% Pure Coconut Flakes | Healthy | Healthy |
| Almond Dukkah | Healthy | Healthy |
| Oven Roasted Almonds & Peanuts | Healthy | Healthy |
| Almonds & Pepitas | Healthy | Healthy |
| Savoury Honey Roasted Peanuts | Unhealthy | Unhealthy |
| Chilli & Lime Peanut & Cashew Nut Mix | Unhealthy | Unhealthy |
| Sesame & Almond Salad Topper | Unhealthy | Healthy |
| Chilli Coconut & Pecan Salad Topper | Unhealthy | Unhealthy |
| Pumpkin Seed & Cranberry Salad Topper | Unhealthy | Healthy |
| Peanuts & Choc Peanuts | Unhealthy | Unhealthy |
| Natural Coconut Chips | Unhealthy | Unhealthy |
| Peanuts & Almonds | Healthy | Healthy |
| Natural Almonds & Cashews | Healthy | Healthy |
| Pistachio and Lemon Myrtle Flavoured Crunchy Egyptian Dukkah | Unhealthy | Unhealthy |
| Crazy Coconut Roasted Coconut Chips | Unhealthy | Unhealthy |
| Red Pepper & Roast Garlic Deli Style Coated Peanuts | Unhealthy | Unhealthy |
| Roma Balsamic & Rosemary Spiced Seeds and Nuts | Unhealthy | Unhealthy |
| Sea Salt Flavour Toasted Coconut Flakes | Unhealthy | Unhealthy |
| Tamari Almonds | Unhealthy | Unhealthy |
| Australian Peeled Chestnuts | Healthy | Healthy |
| Thai Sweet Chilli & Lime Cashews | Unhealthy | Unhealthy |
| Sweet Potato & Turmeric Coated Nuts | Healthy | Healthy |
| Salted Cashews | Healthy | Healthy |
| Chia + Sesame Salad Booster | Healthy | Healthy |
| Activated Organic Pistachios | Unhealthy | Unhealthy |
| Dry Roasted Honey Mixed Nuts | Unhealthy | Unhealthy |
| Maple Cashews | Unhealthy | Unhealthy |
| Super Seed Sprinkles | Unhealthy | Unhealthy |
| BBQ Flavour Coated Peanuts | Unhealthy | Unhealthy |
| Pumpkin Seeds with Pink Himalayan Salt | Healthy | Healthy |
| Australian Natural Almond | Healthy | Healthy |
| Asian Black Sesame Dukkah Salad Sprinkles | Healthy | Healthy |
| Chia + Coconut Breakfast Booster | Healthy | Healthy |
| Matcha & Yoghurt Almonds | Unhealthy | Unhealthy |
| Salted Mixed Nuts | Unhealthy | Unhealthy |
| Organic Toasted Coconut Flakes | Healthy | Healthy |
| Salted Roasted Organic Pumpkin Seed Kernels | Healthy | Healthy |
| Vanilla Maple Flavoured Pecans | Unhealthy | Unhealthy |
| Activated Super Seeds | Unhealthy | Unhealthy |
| Crunchy Roasted & Salted Almonds | Healthy | Healthy |
| Mild & Delicious Pecan Halves | Healthy | Healthy |
| Dry Roasted Cashews | Healthy | Healthy |
| Crunchy Roasted & Salted Pistachio | Unhealthy | Unhealthy |
| Keto Mix | Healthy | Healthy |
| Macadamia & Cashew Salted Mix | Healthy | Healthy |
| Mitey Aussie Vegemite Nuts | Unhealthy | Unhealthy |
| Moist Coconut Flakes | Unhealthy | Unhealthy |
| Salted Roasted Cashews | Healthy | Healthy |
| Spiced Sprouted Sunflower Seeds | Unhealthy | Unhealthy |
| Caramel Toasted Coconut Chips | Unhealthy | Unhealthy |
| Egg Substitute | Healthy | Healthy |
| Almond, Walnut & Cashew Mix | Healthy | Healthy |
| Za'atar Seasoning Blend | Unhealthy | Unhealthy |
| Outdoor Mix | Unhealthy | Unhealthy |
| Caramelized Almonds | Unhealthy | Unhealthy |
| Seed, chia, dried | Healthy | Healthy |
| Seed, linseed or flaxseed | Healthy | Healthy |
| Seed, poppy | Healthy | Healthy |
| Seed, pumpkin, hulled & dried, unsalted | Healthy | Healthy |
| Seed, sesame, unsalted | Healthy | Healthy |
| Seed, sunflower, unsalted | Healthy | Healthy |
| Wattle seed (acacia), ground | Healthy | Healthy |
| Psyllium, dry, uncooked | Healthy | Healthy |
| Tahini, sesame seed pulp | Healthy | Healthy |
| Nut, peanut, with skin, raw or dry roasted, unsalted | Healthy | Healthy |
| Nut, peanut, with skin, roasted, with oil, salted | Healthy | Healthy |
| Nut, peanut, without skin, roasted, with oil, salted | Healthy | Healthy |
| Nut, peanut, without skin, roasted, with oil, unsalted | Healthy | Healthy |
| Nut, peanut, roasted, coated in honey | Unhealthy | Unhealthy |
| Coconut, fresh, mature fruit, flesh | Healthy | Healthy |
| Coconut, fresh, young or immature, flesh | Healthy | Healthy |
| Coconut, fresh, mature, water or juice | Healthy | Healthy |
| Coconut, fresh, young or immature, water or juice | Healthy | Healthy |
| Coconut, grated & desiccated | Healthy | Healthy |
| Nut, almond meal | Healthy | Healthy |
| Nut, almond, with or without skin, roasted, salted | Healthy | Healthy |
| Nut, almond, with or without skin, raw, unsalted | Healthy | Healthy |
| Nut, almond, without skin, blanched, unsalted | Healthy | Healthy |
| Nut, brazil, with or without skin, raw, unsalted | Healthy | Healthy |
| Nut, cashew, raw, unsalted | Healthy | Healthy |
| Nut, cashew, roasted, salted | Healthy | Healthy |
| Nut, cashew, roasted, unsalted | Healthy | Healthy |
| Nut, cashew, roasted, coated in honey | Unhealthy | Unhealthy |
| Nut, chestnut, raw, unsalted | Healthy | Healthy |
| Nut, chestnut, roasted, unsalted | Healthy | Healthy |
| Nut, hazelnut, with or without skin, raw, unsalted | Healthy | Healthy |
| Nut, macadamia, raw, unsalted | Healthy | Healthy |
| Nut, macadamia, roasted, salted | Healthy | Healthy |
| Nut, macadamia, roasted, coated in honey | Unhealthy | Unhealthy |
| Nut, pecan, raw, unsalted | Healthy | Healthy |
| Nut, pine, raw, unsalted | Healthy | Healthy |
| Nut, pistachio, roasted, salted | Unhealthy | Unhealthy |
| Nut, pistachio, raw, unsalted | Healthy | Healthy |
| Nut, walnut, raw, unsalted | Healthy | Healthy |
| Pandanus kernel | Healthy | Healthy |
| Mixed nuts, almond, cashew, peanut, salted | Healthy | Healthy |
| Mixed nuts, cashew & macadamia, salted | Healthy | Healthy |
| Mixed nuts, peanut & 2 or fewer other nuts, salted | Healthy | Healthy |
| Mixed nuts, three or fewer, without peanuts, salted | Healthy | Healthy |
| Mixed nuts, peanut & 3 or more other nuts, salted | Healthy | Healthy |
| **Vegetables** | | |
| Corn Cobbettes | Healthy | Healthy |
| Crinkle Cut Potato Chips | Unhealthy | Healthy |
| European Style Cucumbers | Unhealthy | Unhealthy |
| Grilled Artichokes | Unhealthy | Unhealthy |
| Hot Chips Steakhouse | Unhealthy | Unhealthy |
| Piccalilli Pickle | Unhealthy | Unhealthy |
| Hot Chilli Beans | Unhealthy | Unhealthy |
| Limited Edition Chorizo & Caramelised Onion Wedges | Unhealthy | Unhealthy |
| Roasted Duck Fat Potatoes | Healthy | Healthy |
| Scalloped Potatoes | Unhealthy | Healthy |
| Fiery Mexican Style Beanz | Unhealthy | Unhealthy |
| Spanish Style Beanz Flavoured with Chorizo | Unhealthy | Unhealthy |
| Vintage Cheddar Potato Gratin | Healthy | Healthy |
| Cocktail Onions | Unhealthy | Unhealthy |
| Refried Pinto Beans | Healthy | Healthy |
| Super Juicy Corn Kernels | Healthy | Healthy |
| Yellow Split Peas | Healthy | Healthy |
| Kimchi | Unhealthy | Unhealthy |
| Semi Dried Tomatoes | Healthy | Healthy |
| Black Turtle Beans | Healthy | Healthy |
| Whole Champignons | Healthy | Healthy |
| Baby Roma Tomatoes | Healthy | Healthy |
| Great Northern Beans | Healthy | Healthy |
| Four Bean Mix | Healthy | Healthy |
| 100% Natural Curried Pumpkin Bites | Unhealthy | Unhealthy |
| Potato Crinkles | Unhealthy | Unhealthy |
| Baby Potatoes with Broccoli, Carrots, Peas & Herbs | Unhealthy | Healthy |
| Vegetable Mash | Healthy | Healthy |
| Winter Vegetables | Healthy | Healthy |
| Roasted Vegetables Delights | Unhealthy | Healthy |
| Champignon Pieces & Stems | Healthy | Healthy |
| Raw Sauerkraut | Unhealthy | Unhealthy |
| Bubble 'N Squeak | Unhealthy | Healthy |
| Pickled Jalapeno Beans | Unhealthy | Unhealthy |
| Sriracha Beans | Unhealthy | Unhealthy |
| Green Chilli Jam | Unhealthy | Unhealthy |
| Sweet Potato Straight Cut Chips | Unhealthy | Unhealthy |
| Lightly Seasoned Crisps | Unhealthy | Unhealthy |
| Sweet Potato & Herbs Bites | Unhealthy | Healthy |
| Australian Carrots, Peas & Corn | Healthy | Healthy |
| Potato Mash with Butter | Healthy | Healthy |
| White Cocktail Onions | Unhealthy | Unhealthy |
| Australian Sweet Potato Noodles | Healthy | Healthy |
| Australian Sweet Potato Chips | Healthy | Healthy |
| Waffle Cut Fries | Unhealthy | Unhealthy |
| Shanghai Stir Fry Vegetables | Healthy | Healthy |
| Organic Falafel Mix | Unhealthy | Unhealthy |
| Simply Coat & Bake Italiano Mushrooms with Parmesan and Pepper | Unhealthy | Unhealthy |
| Cold Blended Chunky Garlic Paste | Unhealthy | Unhealthy |
| Seasonal Veg Mash | Healthy | Healthy |
| Edamame Salad | Healthy | Healthy |
| Premium Quality Unmodified Potato Starch | Healthy | Healthy |
| Beetroot Slices | Unhealthy | Healthy |
| Chicken Chips | Unhealthy | Unhealthy |
| Roasted Laver for Sushi | Unhealthy | Unhealthy |
| Creamy Potato Salad | Unhealthy | Unhealthy |
| Mixed Leaf Baby Spinach & Rocket | Healthy | Healthy |
| Country Style Slow Cooked Baked Beans | Unhealthy | Unhealthy |
| Wedges With Gravy & Cheese | Unhealthy | Unhealthy |
| Dark Kidney Beans | Healthy | Healthy |
| Sea Salt and Truffle Flavoured Chips | Unhealthy | Unhealthy |
| Australian Sliced Beetroot | Healthy | Healthy |
| Vegetable Mash | Healthy | Healthy |
| Stir Fry Vegetables with Mushrooms Mix | Unhealthy | Healthy |
| Sweet Potato Wedges | Healthy | Healthy |
| Pontiac Potato, Curry and Thyme | Healthy | Healthy |
| Straight Cut Australian Potato Chips | Unhealthy | Unhealthy |
| Australian Whole Baby Beans | Healthy | Healthy |
| Waffle Cut Fries | Unhealthy | Unhealthy |
| Four Leaf Salad | Healthy | Healthy |
| Carrot, Cauliflower, Broccoli & Baby Corn | Healthy | Healthy |
| Pub Style Hot Bandito | Healthy | Healthy |
| Balsamic Glaze with Kale, Roasted Sweet Potato and Feta | Unhealthy | Unhealthy |
| Garlic Miso with Chinese Broccoli, Choy Sum and Snow Peas | Healthy | Healthy |
| Fried Onion | Healthy | Healthy |
| Tuscan Pesto Beans & Peas with Oven Roasted Tomatoes | Unhealthy | Unhealthy |
| Australian Butter Leaf | Healthy | Healthy |
| Carrot, Cucumber, Capsicum & Tzatziki | Unhealthy | Healthy |
| Baby Potatoes Steamer Tray | Healthy | Healthy |
| Roast Potatoes Glazed with Duck Fat | Unhealthy | Unhealthy |
| Barbecue Potatoes | Unhealthy | Unhealthy |
| Sweet Potato Chips Seasoned with Paprika | Unhealthy | Unhealthy |
| Australian Dill Cucumbers | Unhealthy | Unhealthy |
| Single Leaf Baby Leaf Rocket | Healthy | Healthy |
| Italian Style Soup Mix | Healthy | Healthy |
| Australian Baby Rocket | Healthy | Healthy |
| Australian Chopped Kale | Healthy | Healthy |
| Organic Sweet Potato Rosti | Unhealthy | Unhealthy |
| Organic Wild Fermented Garlic | Unhealthy | Unhealthy |
| Australian Baby Potatoes with Parsley & Butter | Healthy | Healthy |
| Crème Royale Mashed Potato | Healthy | Healthy |
| Grilled Artichokes Slices in Sunflower Oil | Unhealthy | Unhealthy |
| Peri Peri Chips | Unhealthy | Unhealthy |
| Refried Beans | Unhealthy | Healthy |
| Ginger, pickled, drained | Unhealthy | Unhealthy |
| Mixed vegetables, pickled, drained | Unhealthy | Unhealthy |
| Onion, pickled, drained, commercial | Unhealthy | Unhealthy |
| Nutgrass (nut grass), peeled, raw | Healthy | Healthy |
| Potato, wild harvested, cooked | Healthy | Healthy |
| Yam, wild harvested, cooked | Healthy | Healthy |
| Potato, coliban, peeled, raw | Healthy | Healthy |
| Potato, coliban, peeled, baked, roasted, fried, stir-fried, grilled or BBQ'd, no added fat | Healthy | Healthy |
| Potato, coliban, peeled, boiled, microwaved or steamed, drained | Healthy | Healthy |
| Potato, desiree, peeled, raw | Healthy | Healthy |
| Potato, desiree, peeled, baked, roasted, fried, stir-fried, grilled or BBQ'd, no added fat | Healthy | Healthy |
| Potato, desiree, peeled, boiled, microwaved or steamed, drained | Healthy | Healthy |
| Potato, desiree, unpeeled, raw | Healthy | Healthy |
| Potato, desiree, unpeeled, baked, roasted, fried, stir-fried, grilled or BBQ'd, no added fat | Healthy | Healthy |
| Potato, new, peeled or unpeeled, raw | Healthy | Healthy |
| Potato, new, peeled or unpeeled, baked, roasted, fried, stir-fried, grilled or BBQ'd, no added fat | Healthy | Healthy |
| Potato, new, peeled or unpeeled, boiled, microwaved or steamed, drained | Healthy | Healthy |
| Potato, pontiac, peeled, raw | Healthy | Healthy |
| Potato, pontiac, peeled, baked, roasted, fried, stir-fried, grilled or BBQ'd, no added fat | Healthy | Healthy |
| Potato, pontiac, peeled, boiled, microwaved or steamed, drained | Healthy | Healthy |
| Potato, sebago, peeled, raw | Healthy | Healthy |
| Potato, sebago, peeled, baked, roasted, fried, stir-fried, grilled or BBQ'd, animal fat | Healthy | Healthy |
| Potato, sebago, unpeeled, baked, roasted, fried, stir-fried, grilled or BBQ'd, no added fat | Healthy | Healthy |
| Potato, sebago, unpeeled, boiled, microwaved or steamed, drained | Healthy | Healthy |
| Potato, pale skin, peeled, raw | Healthy | Healthy |
| Potato, pale skin, peeled, baked, roasted, fried, stir-fried, grilled or BBQ'd, no added fat | Healthy | Healthy |
| Potato, pale skin, peeled, boiled, microwaved or steamed, drained | Healthy | Healthy |
| Potato, pale skin, unpeeled, raw | Healthy | Healthy |
| Potato, pale skin, unpeeled, baked, roasted, fried, stir-fried, grilled or BBQ'd, no added fat | Healthy | Healthy |
| Potato, pale skin, unpeeled, boiled, microwaved or steamed, drained | Healthy | Healthy |
| Potato, red skin, peeled, raw | Healthy | Healthy |
| Potato, red skin, peeled, baked, roasted, fried, stir-fried, grilled or BBQ'd, no added fat | Healthy | Healthy |
| Potato, red skin, peeled, boiled, microwaved or steamed, drained | Healthy | Healthy |
| Potato, red skin, unpeeled, raw | Healthy | Healthy |
| Potato, red skin, unpeeled, baked, roasted, fried, stir-fried, grilled or BBQ'd, no added fat | Healthy | Healthy |
| Potato, red skin, unpeeled, boiled, microwaved or steamed, drained | Healthy | Healthy |
| Potato, unpeeled, baked, roasted, fried, stir-fried, grilled or BBQ'd, no added fat | Healthy | Healthy |
| Potato, unpeeled, boiled, microwaved or steamed, drained, with or without added fat | Healthy | Healthy |
| Starch, potato | Unhealthy | Unhealthy |
| Potato, chips, regular, fast food outlet, deep fried, blended oil, salted | Unhealthy | Unhealthy |
| Potato, chips, regular, fast food outlet, deep fried, monounsaturated oil, salted | Unhealthy | Unhealthy |
| Potato, chips, regular, independent takeaway outlet, cafe or restaurant, deep fried, blended oil, no added salt | Unhealthy | Unhealthy |
| Potato, chips, regular, independent takeaway outlet, cafe or restaurant, deep fried, blended oil, salted | Unhealthy | Unhealthy |
| Potato, chips, regular, purchased frozen, baked or roasted, no added fat | Unhealthy | Unhealthy |
| Potato, chips, regular, purchased frozen, baked or roasted, fat not further defined | Unhealthy | Unhealthy |
| Potato, chips, regular, purchased frozen, deep fried or fried, fat not further defined | Unhealthy | Unhealthy |
| Potato, chips, reduced fat, purchased frozen, baked or roasted, no added fat | Unhealthy | Unhealthy |
| Potato, fries, fast food outlet, deep fried, blended oil, salted | Unhealthy | Unhealthy |
| Potato, fries, fast food outlet, deep fried, monounsaturated oil, no added salt | Unhealthy | Unhealthy |
| Potato, fries, fast food outlet, deep fried, monounsaturated oil, salted | Unhealthy | Unhealthy |
| Potato, fries, independent takeaway outlet, cafe or restaurant, deep fried, blended oil, no added salt | Unhealthy | Unhealthy |
| Potato, fries, independent takeaway outlet, cafe or restaurant, deep fried, blended oil, salted | Unhealthy | Unhealthy |
| Potato, fries, regular, purchased frozen, par-fried in canola oil, raw | Unhealthy | Unhealthy |
| Potato, fries, regular, purchased frozen, baked or roasted, no added fat | Unhealthy | Unhealthy |
| Potato, fries, regular, purchased frozen, deep fried or fried, fat not further defined | Unhealthy | Unhealthy |
| Potato, gem, nugget or royal, independent takeaway outlet, cafe or restaurant, deep fried, fat not further defined | Unhealthy | Unhealthy |
| Potato, gem, nugget or royal, regular, purchased frozen, par-fried in canola oil, raw | Unhealthy | Healthy |
| Potato, gem, nugget or royal, regular, purchased frozen, baked or roasted, with or without added fat | Unhealthy | Healthy |
| Potato, gem, nugget or royal, regular, purchased frozen, deep fried or fried, fat not further defined | Unhealthy | Healthy |
| Potato, hash brown, McDonalds | Unhealthy | Unhealthy |
| Potato, hash brown, independent takeaway outlet, cafe or restaurant, deep fried, oil not further defined | Unhealthy | Unhealthy |
| Potato, hash brown, purchased frozen, par-fried in canola oil, raw | Unhealthy | Healthy |
| Potato, hash brown, purchased frozen, baked, roasted, grilled or BBQ'd, no added fat | Unhealthy | Healthy |
| Potato, hash brown, purchased frozen, baked or roasted, fat not further defined | Unhealthy | Unhealthy |
| Potato scallop, deep fried, saturated frying fat, salted | Unhealthy | Unhealthy |
| Potato, wedges, independent takeaway outlet, cafe or restaurant, deep fried, blended oil, salted | Unhealthy | Unhealthy |
| Potato, wedges, regular, purchased frozen, par-fried in canola oil, raw | Unhealthy | Healthy |
| Potato, wedges, regular, purchased frozen, baked or roasted, no added fat | Unhealthy | Healthy |
| Potato, wedges, regular, purchased frozen, deep fried or fried, fat not further defined | Unhealthy | Healthy |
| Potato, mashed, dried powder | Unhealthy | Unhealthy |
| Potato, mashed, prepared from dried powder with cows milk or water | Unhealthy | Unhealthy |
| Potato, mashed, with gravy, as purchased from a fast food outlet | Unhealthy | Unhealthy |
| Potato, sebago, peeled, boiled & mashed without added ingredients | Healthy | Healthy |
| Bok choy or choy sum, raw | Healthy | Healthy |
| Bok choy or choy sum, baked, roasted, fried, stir-fried, grilled or BBQ'd, no added fat | Healthy | Healthy |
| Bok choy or choy sum, boiled, microwaved or steamed, drained | Healthy | Healthy |
| Brussels sprout, fresh or frozen, raw | Healthy | Healthy |
| Brussels sprout, fresh, boiled, microwaved or steamed, drained | Healthy | Healthy |
| Brussels sprout, frozen, boiled, microwaved or steamed , drained | Healthy | Healthy |
| Cabbage, Chinese, raw | Healthy | Healthy |
| Cabbage, Chinese, baked, roasted, fried, stir-fried, grilled or BBQ'd, no added fat | Healthy | Healthy |
| Cabbage, Chinese, boiled, microwaved or steamed, drained | Healthy | Healthy |
| Cabbage, Chinese flowering, raw | Healthy | Healthy |
| Cabbage, mustard, raw | Healthy | Healthy |
| Cabbage, mustard, cooked | Healthy | Healthy |
| Cabbage, red, raw | Healthy | Healthy |
| Cabbage, red, boiled, microwaved or steamed, drained, with or without added fat | Healthy | Healthy |
| Cabbage, red, canned, heated, drained | Healthy | Healthy |
| Cabbage, savoy, raw | Healthy | Healthy |
| Cabbage, savoy, baked, roasted, fried, stir-fried, grilled or BBQ'd, with or without added fat | Healthy | Healthy |
| Cabbage, savoy, boiled, microwaved or steamed, drained, with or without added fat | Healthy | Healthy |
| Cabbage, white, raw | Healthy | Healthy |
| Cabbage, white, boiled, microwaved or steamed, drained, with and without added fat | Healthy | Healthy |
| Cabbage, pickled, canned, drained | Healthy | Healthy |
| Kale, raw | Healthy | Healthy |
| Kale, cooked | Healthy | Healthy |
| Kohlrabi, peeled, fresh or frozen, raw | Healthy | Healthy |
| Kohlrabi, peeled, fresh or frozen, boiled, microwaved or steamed, drained | Healthy | Healthy |
| Broccoli, fresh or frozen, raw | Healthy | Healthy |
| Broccoli, fresh or frozen, baked, roasted, fried, stir-fried, grilled or BBQ'd, no added fat | Healthy | Healthy |
| Broccoli, fresh, boiled, microwaved or steamed, drained | Healthy | Healthy |
| Broccoli, frozen, boiled, microwaved or steamed, drained | Healthy | Healthy |
| Broccolini, fresh or frozen, raw | Healthy | Healthy |
| Broccolini, fresh or frozen, boiled, microwaved or steamed, drained | Healthy | Healthy |
| Cauliflower, fresh or frozen, raw | Healthy | Healthy |
| Cauliflower, fresh or frozen, baked, roasted, fried, stir-fried, grilled or BBQ'd, no added fat | Healthy | Healthy |
| Cauliflower, fresh or frozen, boiled, microwaved or steamed, drained | Healthy | Healthy |
| Carrot, baby, peeled or unpeeled, fresh or frozen, raw | Healthy | Healthy |
| Carrot, baby, peeled or unpeeled, fresh or frozen, boiled, microwaved or steamed, drained | Healthy | Healthy |
| Carrot, baby, canned in brine, boiled or microwaved , drained | Healthy | Healthy |
| Carrot, mature, peeled or unpeeled, fresh or frozen, raw | Healthy | Healthy |
| Carrot, mature, peeled or unpeeled, fresh or frozen, baked, roasted, fried, stir-fried, grilled or BBQ'd, no added fat | Healthy | Healthy |
| Carrot, mature, peeled or unpeeled, fresh or frozen, boiled, microwaved or steamed, drained | Healthy | Healthy |
| Artichoke, jerusalem, peeled, raw | Healthy | Healthy |
| Artichoke, jerusalem, peeled, boiled, microwaved or steamed, drained | Healthy | Healthy |
| Beetroot, purple, peeled, fresh or frozen, raw | Healthy | Healthy |
| Beetroot, purple, peeled or unpeeled, fresh or frozen, baked, roasted, fried, stir-fried, grilled or BBQ'd, no added fat | Healthy | Healthy |
| Beetroot, purple, peeled, fresh or frozen, boiled, microwaved or steamed, drained | Healthy | Healthy |
| Beetroot, canned, drained | Healthy | Healthy |
| Cassava, peeled, fresh or frozen, raw | Healthy | Healthy |
| Cassava, peeled, fresh or frozen, boiled, microwaved or steamed, drained | Healthy | Healthy |
| Cassava, white flesh, peeled, fresh or frozen, raw | Healthy | Healthy |
| Cassava, white flesh, peeled, fresh or frozen, boiled, microwaved or steamed, drained | Healthy | Healthy |
| Cassava, yellow flesh, peeled, fresh or frozen, raw | Healthy | Healthy |
| Cassava, yellow flesh, peeled, fresh or frozen, boiled, microwaved or steamed, drained | Healthy | Healthy |
| Celeriac, peeled, raw | Healthy | Healthy |
| Celeriac, peeled, boiled, microwaved or steamed, drained | Healthy | Healthy |
| Chicory, raw | Healthy | Healthy |
| Chicory, boiled, microwaved or steamed, drained | Healthy | Healthy |
| Ginger, peeled, fresh or frozen, raw | Healthy | Healthy |
| Ginger, peeled, fresh or frozen, baked, roasted, fried, stir-fried, grilled or BBQ'd, with or without added fat | Healthy | Healthy |
| Ginger, peeled, fresh or frozen, boiled, microwaved or steamed, with or without added fat | Healthy | Healthy |
| Parsnip, peeled, fresh or frozen, raw | Healthy | Healthy |
| Parsnip, peeled or unpeeled, fresh or frozen, baked, roasted, fried, stir-fried, grilled or BBQ'd, no added fat | Healthy | Healthy |
| Parsnip, peeled or unpeeled, fresh or frozen, boiled, microwaved or steamed, drained, with or without added fat | Healthy | Healthy |
| Radish, red skinned, unpeeled, raw | Healthy | Healthy |
| Radish, red skinned, unpeeled, baked, roasted, fried, stir-fried, grilled or BBQ'd, no added fat | Healthy | Healthy |
| Radish, white skinned, peeled or unpeeled, raw | Healthy | Healthy |
| Radish, white skinned, peeled or unpeeled, baked, roasted, fried, stir-fried, grilled or BBQ'd, no added fat | Healthy | Healthy |
| Swede, peeled, fresh or frozen, raw | Healthy | Healthy |
| Swede, peeled, fresh or frozen, boiled, microwaved or steamed, drained | Healthy | Healthy |
| Sweet potato, orange flesh, peeled or unpeeled, fresh or frozen, raw | Healthy | Healthy |
| Sweet potato, orange flesh, peeled or unpeeled, fresh or frozen, baked, roasted, fried, stir-fried, grilled or BBQ'd, no added fat | Healthy | Healthy |
| Sweet potato, orange flesh, peeled or unpeeled, fresh or frozen, boiled, microwaved or steamed, drained | Healthy | Healthy |
| Sweet potato, white flesh, peeled or unpeeled, fresh or frozen, raw | Healthy | Healthy |
| Sweet potato, white flesh, peeled or unpeeled, fresh or frozen, baked, roasted, fried, stir-fried, grilled or BBQ'd, no added fat | Healthy | Healthy |
| Sweet potato, white flesh, peeled or unpeeled, fresh or frozen, boiled, microwaved or steamed, drained | Healthy | Healthy |
| Sweet potato, chips, regular, purchased frozen, par-fried in canola oil, raw | Healthy | Healthy |
| Sweet potato, chips, regular, purchased frozen, baked or roasted, with or without added fat | Healthy | Healthy |
| Taro, peeled, fresh or frozen, raw | Healthy | Healthy |
| Taro, peeled, fresh or frozen, boiled, microwaved or steamed, drained | Healthy | Healthy |
| Turnip, white, peeled, fresh or frozen, raw | Healthy | Healthy |
| Turnip, white, peeled or unpeeled, fresh or frozen, baked, roasted, fried, stir-fried, grilled or BBQ'd, with or without added fat | Healthy | Healthy |
| Turnip, white, peeled or unpeeled, fresh or frozen, boiled, microwaved or steamed, drained, with or without added fat | Healthy | Healthy |
| Wasabi, root, raw | Healthy | Healthy |
| Endive, raw | Healthy | Healthy |
| Lettuce, cos, raw | Healthy | Healthy |
| Lettuce, iceberg, raw | Healthy | Healthy |
| Lettuce, mignonette, raw | Healthy | Healthy |
| Lettuce, baked, roasted, fried, stir-fried, grilled or BBQ'd, with or without added fat | Healthy | Healthy |
| Lettuce, boiled, casseroled, microwaved, poached, steamed or stewed, with or without added fat | Healthy | Healthy |
| Rocket, raw | Healthy | Healthy |
| Rocket, cooked, with or without added fat | Healthy | Healthy |
| Silverbeet, fresh or frozen, raw | Healthy | Healthy |
| Silverbeet, fresh or frozen, boiled, microwaved or steamed, drained | Healthy | Healthy |
| Spinach, fresh, raw | Healthy | Healthy |
| Spinach, fresh, baked, roasted, fried, stir-fried, grilled or BBQ'd , no added fat | Healthy | Healthy |
| Spinach, fresh, boiled, microwaved or steamed, drained | Healthy | Healthy |
| Spinach, frozen, boiled, microwaved or steamed, drained | Healthy | Healthy |
| Spinach, water, raw | Healthy | Healthy |
| Vine leaf, grape, canned | Unhealthy | Unhealthy |
| Watercress, raw | Healthy | Healthy |
| Watercress, boiled, microwaved or steamed, drained | Healthy | Healthy |
| Artichoke, globe, raw | Healthy | Healthy |
| Artichoke, globe, boiled, microwaved or steamed, drained | Healthy | Healthy |
| Artichoke heart, canned in brine, drained | Healthy | Healthy |
| Asparagus, green, raw | Healthy | Healthy |
| Asparagus, green, fresh or frozen, baked, roasted, stir-fried or fried, grilled or BBQ'd, no added fat | Healthy | Healthy |
| Asparagus, green, fresh or frozen, boiled, microwaved or steamed, drained | Healthy | Healthy |
| Asparagus, canned in brine, drained or undrained | Healthy | Healthy |
| Bamboo shoot, canned in water, heated, drained | Healthy | Healthy |
| Bamboo shoot, fresh, cooked, with or without fat | Healthy | Healthy |
| Celery, fresh or frozen, raw | Healthy | Healthy |
| Celery, fresh or frozen, baked, roasted, fried, stir-fried, grilled or BBQ'd, no added fat | Healthy | Healthy |
| Celery, fresh or frozen, boiled, microwaved or steamed, drained | Healthy | Healthy |
| Seaweed, nori, dried | Healthy | Healthy |
| Pea, green, fresh or frozen, raw | Healthy | Healthy |
| Pea, green, fresh, cooked, no added fat | Healthy | Healthy |
| Pea, green, frozen, cooked, no added fat | Healthy | Healthy |
| Pea, green, canned in brine, cooked | Healthy | Healthy |
| Snow pea, fresh or frozen, raw | Healthy | Healthy |
| Snow pea, fresh or frozen, baked, roasted, fried, stir-fried, grilled or BBQ'd, no added fat | Healthy | Healthy |
| Snow pea, fresh or frozen, boiled, microwaved or steamed, drained | Healthy | Healthy |
| Bean, broad, fresh or frozen, raw | Healthy | Healthy |
| Bean, broad, fresh or frozen, boiled, microwaved or steamed, drained | Healthy | Healthy |
| Bean, butter, fresh, raw | Healthy | Healthy |
| Bean, butter, fresh, boiled, microwaved or steamed, drained | Healthy | Healthy |
| Bean, green, fresh or frozen, raw | Healthy | Healthy |
| Bean, green, fresh or frozen, baked, roasted, fried, stir-fried, grilled or BBQ'd, no added fat | Healthy | Healthy |
| Bean, green, fresh, boiled, microwaved or steamed, drained | Healthy | Healthy |
| Bean, green, frozen, boiled, microwaved or steamed, drained | Healthy | Healthy |
| Bean, green, canned, cooked, no added fat | Healthy | Healthy |
| Bean, red, fresh, raw | Healthy | Healthy |
| Bean, red, fresh, boiled, drained | Healthy | Healthy |
| Sprout, alfalfa, raw | Healthy | Healthy |
| Sprout, bean, raw | Healthy | Healthy |
| Sprout, bean, cooked, no added fat | Healthy | Healthy |
| Tomato, cherry or grape, raw | Healthy | Healthy |
| Tomato, common, raw | Healthy | Healthy |
| Tomato, common, boiled with salt, drained | Healthy | Healthy |
| Tomato, hydroponic, raw | Healthy | Healthy |
| Tomato, roma, raw | Healthy | Healthy |
| Tomato, puree, commercial | Healthy | Healthy |
| Tomato, sundried or semi-sundried | Healthy | Healthy |
| Tomato, whole, canned in tomato juice, undrained | Healthy | Healthy |
| Tomato, whole, canned in tomato juice, drained | Healthy | Healthy |
| Tomato, whole, canned in tomato juice, boiled or microwaved, undrained | Healthy | Healthy |
| Tomato, whole, canned in tomato juice, boiled or microwaved, drained | Healthy | Healthy |
| Pumpkin, butternut, peeled, fresh or frozen, raw | Healthy | Healthy |
| Pumpkin, butternut, peeled, fresh or frozen, baked, roasted, fried, stir-fried, grilled or BBQ'd, no added fat | Healthy | Healthy |
| Pumpkin, butternut, peeled, fresh or frozen, boiled, microwaved or steamed, drained, with or without added fat | Healthy | Healthy |
| Pumpkin, golden nugget, peeled, fresh or frozen, raw | Healthy | Healthy |
| Pumpkin, golden nugget, peeled, fresh or frozen, baked or roasted, no added fat | Healthy | Healthy |
| Pumpkin, jarrahdale, peeled, fresh or frozen, raw | Healthy | Healthy |
| Pumpkin, jarrahdale, peeled, fresh or frozen, baked or roasted, no added fat | Healthy | Healthy |
| Pumpkin, jarrahdale, peeled, fresh or frozen, boiled, microwaved or steamed, drained | Healthy | Healthy |
| Pumpkin, queensland blue, peeled, fresh or frozen, raw | Healthy | Healthy |
| Pumpkin, queensland blue, peeled, fresh or frozen, baked or roasted, no added fat | Healthy | Healthy |
| Pumpkin, queensland blue, peeled, fresh or frozen, boiled, microwaved or steamed, drained | Healthy | Healthy |
| Pumpkin, peeled, fresh or frozen, raw | Healthy | Healthy |
| Pumpkin, peeled, fresh or frozen, baked, roasted, fried, stir-fried, grilled or BBQ'd, no added fat | Healthy | Healthy |
| Pumpkin, peeled, fresh or frozen, boiled, microwaved or steamed, drained | Healthy | Healthy |
| Pumpkin, unpeeled, fresh or frozen, raw | Healthy | Healthy |
| Pumpkin, unpeeled, fresh or frozen, baked, roasted, fried, stir-fried, grilled or BBQ'd, no added fat | Healthy | Healthy |
| Pumpkin, unpeeled, fresh or frozen, boiled, microwaved or steamed, drained, with or without added fat | Healthy | Healthy |
| Squash, button, fresh or frozen, raw | Healthy | Healthy |
| Squash, button, fresh or frozen, boiled, microwaved or steamed, drained | Healthy | Healthy |
| Squash, scallopini, fresh or frozen, raw | Healthy | Healthy |
| Squash, scallopini, fresh or frozen, boiled, microwaved or steamed, drained | Healthy | Healthy |
| Zucchini, golden, fresh or frozen, peeled or unpeeled, raw | Healthy | Healthy |
| Zucchini, golden, fresh or frozen, peeled or unpeeled, boiled, microwaved or steamed, drained | Healthy | Healthy |
| Zucchini, green skin, fresh or frozen, peeled or unpeeled, raw | Healthy | Healthy |
| Zucchini, green skin, fresh or frozen, peeled or unpeeled, baked, roasted, fried, stir-fried, grilled or BBQ'd, no added fat | Healthy | Healthy |
| Zucchini, green skin, fresh or frozen, peeled or unpeeled, boiled, microwaved or steamed, drained | Healthy | Healthy |
| Mushroom, common, fresh or frozen, raw | Healthy | Healthy |
| Mushroom, common, fresh or frozen, baked, roasted, fried, stir-fried, grilled or BBQ'd, no added fat | Healthy | Healthy |
| Mushroom, common, fresh or frozen, boiled, microwaved or steamed, drained | Healthy | Healthy |
| Mushroom, oriental, fresh or frozen, raw, not further defined | Healthy | Healthy |
| Mushroom, oriental, fresh or frozen, boiled, microwaved or steamed, drained | Healthy | Healthy |
| Mushroom, canned in brine, drained | Healthy | Healthy |
| Mushroom, straw, Asian, canned in brine, drained | Healthy | Healthy |
| Sweetcorn, baby, canned in brine, heated, drained | Healthy | Healthy |
| Sweetcorn, creamed, canned, heated | Healthy | Healthy |
| Sweetcorn, fresh or frozen on cob, raw | Healthy | Healthy |
| Sweetcorn, fresh or frozen on cob, baked, roasted, fried, stir-fried, grilled or BBQ'd, no added fat | Healthy | Healthy |
| Sweetcorn, fresh or frozen on cob, boiled, microwaved or steamed, drained | Healthy | Healthy |
| Sweetcorn, fresh or frozen on cob, boiled or microwaved in brine, drained | Healthy | Healthy |
| Sweetcorn, kernels, canned in brine, drained | Healthy | Healthy |
| Sweetcorn, kernels, canned in brine, heated, drained | Healthy | Healthy |
| Sweetcorn, kernels, fresh or frozen, raw | Healthy | Healthy |
| Sweetcorn, kernels, fresh or frozen, boiled, microwaved or steamed, drained | Healthy | Healthy |
| Sweetcorn, kernels, fresh or frozen, boiled or microwaved in brine, drained | Healthy | Healthy |
| Avocado, raw | Healthy | Healthy |
| Avocado, cooked, with or without fat | Healthy | Healthy |
| Capsicum, green, fresh or frozen, raw | Healthy | Healthy |
| Capsicum, green, fresh or frozen, baked, roasted, fried, stir-fried, grilled or BBQ'd, no added fat | Healthy | Healthy |
| Capsicum, green, fresh or frozen, boiled, microwaved or steamed, drained | Healthy | Healthy |
| Capsicum, red, fresh or frozen, raw | Healthy | Healthy |
| Capsicum, red, fresh or frozen, baked, roasted, fried, stir-fried, grilled or BBQ'd, no added fat | Healthy | Healthy |
| Capsicum, red, fresh or frozen, boiled, microwaved or steamed, drained | Healthy | Healthy |
| Capsicum, fresh or frozen, baked, roasted, fried, stir-fried, grilled or BBQ'd, no added fat | Healthy | Healthy |
| Capsicum, fresh or frozen, boiled, microwaved or steamed, drained | Healthy | Healthy |
| Chilli (chili), green, raw | Healthy | Healthy |
| Chilli (chili), green, cooked with or without added fat | Healthy | Healthy |
| Chilli (chili), green, pickled | Healthy | Healthy |
| Chilli (chili), red, raw | Healthy | Healthy |
| Chilli (chili), red, cooked with or without added fat | Healthy | Healthy |
| Chilli (chili), baked, roasted, fried, stir-fried, grilled or BBQ'd, with or without fat | Healthy | Healthy |
| Chilli (chili), boiled, microwaved or steamed, drained, with or without fat | Healthy | Healthy |
| Choko, peeled, fresh or frozen, raw | Healthy | Healthy |
| Choko, peeled, fresh or frozen, boiled, microwaved or steamed, drained | Healthy | Healthy |
| Cucumber, apple crystal, unpeeled, raw | Healthy | Healthy |
| Cucumber, common, peeled, raw | Healthy | Healthy |
| Cucumber, common, unpeeled, raw | Healthy | Healthy |
| Cucumber, Lebanese, unpeeled, raw | Healthy | Healthy |
| Cucumber, telegraph, unpeeled, raw | Healthy | Healthy |
| Cucumber, peeled or unpeeled, cooked, no added fat | Healthy | Healthy |
| Eggplant, peeled or unpeeled, fresh or frozen, raw | Healthy | Healthy |
| Eggplant, peeled or unpeeled, fresh or frozen, baked, roasted, fried, stir-fried, grilled or BBQ'd, no added fat | Healthy | Healthy |
| Eggplant, peeled or unpeeled, fresh or frozen, boiled, microwaved or steamed, drained | Healthy | Healthy |
| Melon, bitter, fresh or frozen, raw | Healthy | Healthy |
| Melon, bitter, fresh or frozen, baked, roasted, fried, stir-fried, grilled or BBQ'd, no added fat | Healthy | Healthy |
| Melon, hairy, fresh or frozen, raw | Healthy | Healthy |
| Okra, raw | Healthy | Healthy |
| Fennel, fresh or frozen, raw | Healthy | Healthy |
| Fennel, fresh or frozen, boiled, microwaved or steamed, drained | Healthy | Healthy |
| Water chestnut, peeled, canned, drained | Healthy | Healthy |
| Garlic, peeled or unpeeled, fresh or frozen, raw | Healthy | Healthy |
| Garlic, peeled or unpeeled, fresh or frozen, baked, roasted, fried, stir-fried, grilled, BBQ'd, deep-fried, with or without added fat | Healthy | Healthy |
| Garlic, peeled or unpeeled, fresh or frozen, boiled, microwaved or steamed, drained, with or without added fat | Healthy | Healthy |
| Leek, raw | Healthy | Healthy |
| Leek, baked, roasted, fried, stir-fried, grilled or BBQ'd, no added fat | Healthy | Healthy |
| Leek, boiled, microwaved or steamed, drained | Healthy | Healthy |
| Onion, mature, brown skinned, peeled, raw | Healthy | Healthy |
| Onion, mature, red skinned, peeled, fresh or frozen, raw | Healthy | Healthy |
| Onion, mature, white skinned, peeled, fresh or frozen, raw | Healthy | Healthy |
| Onion, spring, raw | Healthy | Healthy |
| Onion, spring, baked, roasted, fried, stir-fried, grilled or BBQ'd, no added fat | Healthy | Healthy |
| Onion, spring, boiled, microwaved or steamed, drained, with or without added fat | Healthy | Healthy |
| Shallot, peeled, raw | Healthy | Healthy |
| Shallot, peeled, baked, roasted, fried, stir-fried, grilled or BBQ'd, no added fat | Healthy | Healthy |
| Vegetable and/or fruit blend, dry powder | Unhealthy | Unhealthy |
| Mixed vegetables, carrot, corn & pea/bean, canned, cooked, no added fat | Healthy | Healthy |
| Mixed vegetables, purchased frozen, carrot, corn & pea/bean, cooked, with or without fat | Healthy | Healthy |
| Bean, black, dried, boiled, microwaved or steamed, drained | Healthy | Healthy |
| Bean, haricot, dried | Healthy | Healthy |
| Bean, haricot, dried, boiled, microwaved or steamed, drained | Healthy | Healthy |
| Bean, lima, dried | Healthy | Healthy |
| Bean, lima, dried, boiled, microwaved or steamed, drained | Healthy | Healthy |
| Bean, lupin, raw | Healthy | Healthy |
| Bean, red kidney, dried | Healthy | Healthy |
| Bean, red kidney, dried, boiled, microwaved or steamed, drained | Healthy | Healthy |
| Bean, soya, dried | Healthy | Healthy |
| Bean, soya, dried, boiled, microwaved or steamed, drained | Healthy | Healthy |
| Chickpea, dried, boiled, microwaved or steamed, drained | Healthy | Healthy |
| Lentil, dried, cooked | Healthy | Healthy |
| Lentil, green or brown, dried | Healthy | Healthy |
| Lentil, green or brown, cooked | Healthy | Healthy |
| Lentil, red, dried | Healthy | Healthy |
| Lentil, red, cooked | Healthy | Healthy |
| Pea, split, dried | Healthy | Healthy |
| Pea, split, dried, boiled, microwaved or steamed, drained | Healthy | Healthy |
| Bean, broad, canned, drained | Healthy | Healthy |
| Bean, cannellini, canned, drained | Healthy | Healthy |
| Bean, lupin, canned, drained | Healthy | Healthy |
| Bean, mixed, canned, drained | Healthy | Healthy |
| Bean, red, kidney, canned, drained | Healthy | Healthy |
| Bean, refried, canned | Healthy | Healthy |
| Bean, soya, canned, drained | Healthy | Healthy |
| Chickpea, canned, drained | Healthy | Healthy |
| Baked beans, canned in BBQ sauce, regular | Unhealthy | Unhealthy |
| Baked beans, canned in tomato sauce, regular | Unhealthy | Unhealthy |
| Baked beans, canned in tomato sauce, reduced salt | Unhealthy | Unhealthy |
| Baked beans, canned in tomato & cheese sauce, regular | Unhealthy | Unhealthy |
| Baked beans, canned in tomato sauce, with sausages, regular | Unhealthy | Unhealthy |
| Baked beans, all flavours (except tomato sauce), reduced salt | Unhealthy | Unhealthy |
| Baked beans, canned, not further defined | Unhealthy | Unhealthy |
| Bean paste | Unhealthy | Unhealthy |
| Falafel, chickpea patty, deep fried, fat not further defined | Unhealthy | Unhealthy |
| **Meat and meat products** | | |
| **Fresh of frozen meat** | | |
| Turkey Breast Steaks | Healthy | Healthy |
| Lean Beef Mince | Healthy | Healthy |
| Kangaroo Steak | Healthy | Healthy |
| Echidna, wild caught, flesh, raw | Healthy | Healthy |
| Echidna, wild caught, flesh, cooked | Healthy | Healthy |
| Dugong, wild caught, flesh, raw | Healthy | Healthy |
| Dugong, wild caught, flesh, cooked | Healthy | Healthy |
| Goose, wild caught, flesh, raw | Healthy | Healthy |
| Goose, wild caught, flesh, cooked | Healthy | Healthy |
| Kangaroo, wild caught, tail, cooked | Healthy | Healthy |
| Possum, wild caught, flesh, cooked | Healthy | Healthy |
| Beef, all cuts, separable fat, raw | Healthy | Healthy |
| Beef, all cuts, separable fat, grilled or roasted without fat | Healthy | Healthy |
| Beef, blade steak, separable fat, raw | Healthy | Healthy |
| Beef, blade steak, separable fat, grilled or BBQ'd, no added fat | Healthy | Healthy |
| Beef, blade steak, separable lean, raw | Healthy | Healthy |
| Beef, blade steak, separable lean, grilled or BBQ'd, no added fat | Healthy | Healthy |
| Beef, chuck steak, separable fat, raw | Healthy | Healthy |
| Beef, chuck steak, separable fat, boiled, casseroled, microwaved, poached, steamed, or stewed, no added fat | Healthy | Healthy |
| Beef, chuck steak, separable lean, raw | Healthy | Healthy |
| Beef, chuck steak, separable lean, boiled, casseroled, microwaved, poached, steamed, or stewed, no added fat | Healthy | Healthy |
| Beef, diced, separable lean, raw | Healthy | Healthy |
| Beef, diced, separable lean, fried or stir-fried, no added fat | Healthy | Healthy |
| Beef, diced, fully-trimmed, baked, roasted, fried or stir-fried, grilled or BBQ'd, no added fat | Healthy | Healthy |
| Beef, eye fillet, separable lean, raw | Healthy | Healthy |
| Beef, eye fillet, separable lean, baked, roasted, fried, grilled or BBQ'd, no added fat | Healthy | Healthy |
| Beef, fillet steak, separable lean, raw | Healthy | Healthy |
| Beef, fillet steak, separable lean, grilled or BBQ'd, no added fat | Healthy | Healthy |
| Beef, fillet steak, fully-trimmed, baked, roasted, fried, grilled or BBQ'd, no added fat | Healthy | Healthy |
| Beef, loin (fillet, sirloin, scotch fillet, T-bone), separable fat, raw | Healthy | Healthy |
| Beef, loin (fillet, sirloin, scotch fillet, T-bone), separable fat, grilled or BBQ'd, no added fat | Healthy | Healthy |
| Beef, mince, <5% fat, raw | Healthy | Healthy |
| Beef, mince, <5% fat, baked, roasted, fried or stir-fried, grilled or BBQ'd, no added fat | Healthy | Healthy |
| Beef, mince, <5% fat, boiled, casseroled, microwaved, poached, steamed, or stewed, no added fat | Healthy | Healthy |
| Beef, mince, ~5-10% fat, raw | Healthy | Healthy |
| Beef, mince, ~5-10% fat, baked, roasted, fried or stir-fried, grilled or BBQ'd, no added fat | Healthy | Healthy |
| Beef, mince, ~5-10% fat, boiled, casseroled, microwaved, poached, steamed, or stewed, no added fat | Healthy | Healthy |
| Beef, mince, >10% fat, raw | Healthy | Healthy |
| Beef, mince, >10% fat, baked, roasted, fried or stir-fried, grilled or BBQ'd, no added fat | Healthy | Healthy |
| Beef, mince, baked, roasted, fried or stir-fried, grilled or BBQ'd, no added fat | Healthy | Healthy |
| Beef, rib cutlet or roast, fully-trimmed, baked, roasted, fried, grilled or BBQ'd, no added fat | Healthy | Healthy |
| Beef, roasting cuts, untrimmed, baked or roasted, no added fat | Healthy | Healthy |
| Beef, round medallion, separable lean, raw | Healthy | Healthy |
| Beef, round medallion, separable lean, grilled or BBQ'd, no added fat | Healthy | Healthy |
| Beef, round steak, separable fat, raw | Healthy | Healthy |
| Beef, round steak, separable fat, grilled or BBQ'd, no added fat | Healthy | Healthy |
| Beef, round steak, separable lean, raw | Healthy | Healthy |
| Beef, round steak, separable lean, grilled or BBQ'd, no added fat | Healthy | Healthy |
| Beef, rump medallion, separable lean, raw | Healthy | Healthy |
| Beef, rump medallion, separable lean, grilled or BBQ'd, no added fat | Healthy | Healthy |
| Beef, rump steak, separable fat, raw | Healthy | Healthy |
| Beef, rump steak, separable fat, grilled or BBQ'd, no added fat | Healthy | Healthy |
| Beef, rump steak, separable lean, raw | Healthy | Healthy |
| Beef, rump steak, separable lean, grilled or BBQ'd, no added fat | Healthy | Healthy |
| Beef, scotch fillet, separable lean, raw | Healthy | Healthy |
| Beef, scotch fillet, separable lean, grilled or BBQ'd, no added fat | Healthy | Healthy |
| Beef, silverside minute steak, separable lean, raw | Healthy | Healthy |
| Beef, silverside minute steak, separable lean, baked or roasted, no added fat | Healthy | Healthy |
| Beef, silverside roast, separable lean, raw | Healthy | Healthy |
| Beef, silverside roast, separable lean, baked or roasted, no added fat | Healthy | Healthy |
| Beef, silverside roast, fully-trimmed, boiled, casseroled, microwaved, poached, steamed, or stewed, no added fat | Healthy | Healthy |
| Beef, silverside roast, untrimmed, boiled, casseroled, microwaved, poached, steamed or stewed, no added fat | Healthy | Healthy |
| Beef, sirloin steak, separable lean, raw | Healthy | Healthy |
| Beef, sirloin steak, separable lean, grilled or BBQ'd, no added fat | Healthy | Healthy |
| Beef, stir-fry strips or diced, separable fat, raw | Healthy | Healthy |
| Beef, stir-fry strips or diced, separable fat, fried or stir-fried, no added fat | Healthy | Healthy |
| Beef, stir-fry strips, separable lean, raw | Healthy | Healthy |
| Beef, stir-fry strips, separable lean, fried or stir-fried, no added fat | Healthy | Healthy |
| Beef, stir-fry strips, fully-trimmed, baked, roasted, fried, stir-fried, grilled or BBQ'd, no added fat | Healthy | Healthy |
| Beef, stir-fry strips, fully-trimmed, boiled, casseroled, microwaved, poached, steamed or stewed, no added fat | Healthy | Healthy |
| Beef, T-bone steak, separable lean, raw | Healthy | Healthy |
| Beef, T-bone steak, separable lean, grilled or BBQ'd, no added fat | Healthy | Healthy |
| Beef, topside or silverside, separable fat, raw | Healthy | Healthy |
| Beef, topside roast, separable lean, raw | Healthy | Healthy |
| Beef, topside roast, separable lean, baked or roasted, no added fat | Healthy | Healthy |
| Beef, topside steak, separable lean, raw | Healthy | Healthy |
| Beef, topside/silverside, separable fat, baked or roasted, no added fat | Healthy | Healthy |
| Beef, topside steak, separable lean, baked or roasted, no added fat | Healthy | Healthy |
| Beef, steak, untrimmed, baked, roasted, fried, grilled or BBQ'd, no added fat | Healthy | Healthy |
| Lamb, all cuts, separable fat, raw | Healthy | Healthy |
| Lamb, all cuts, separable fat, cooked | Healthy | Healthy |
| Lamb, butterfly steak, separable lean, raw | Healthy | Healthy |
| Lamb, butterfly steak, fully-trimmed, baked, roasted, fried, grilled or BBQ'd, no added fat | Healthy | Healthy |
| Lamb, butterfly steak, fully-trimmed, boiled, casseroled, microwaved, poached, steamed, or stewed, no added fat | Healthy | Healthy |
| Lamb, chop, untrimmed, baked, roasted, fried, grilled or BBQ'd, no added fat | Healthy | Healthy |
| Lamb, chump chop, separable lean, raw | Healthy | Healthy |
| Lamb, chump chop, separable lean, grilled or BBQ'd, no added fat | Healthy | Healthy |
| Lamb, diced, separable lean, raw | Healthy | Healthy |
| Lamb, diced, separable lean, fried or stir-fried, no added fat | Healthy | Healthy |
| Lamb, drumstick, separable lean, raw | Healthy | Healthy |
| Lamb, drumstick, separable lean, grilled or BBQ'd, no added fat | Healthy | Healthy |
| Lamb, easy carve leg roast, separable lean, raw | Healthy | Healthy |
| Lamb, easy carve leg roast, separable lean, baked or roasted, no added fat | Healthy | Healthy |
| Lamb, easy carve shoulder, separable lean, raw | Healthy | Healthy |
| Lamb, easy carve shoulder, separable lean, baked or roasted, no added fat | Healthy | Healthy |
| Lamb, eye of loin, separable lean, raw | Healthy | Healthy |
| Lamb, eye of loin, separable lean, grilled or BBQ'd, no added fat | Healthy | Healthy |
| Lamb, fillet or tenderloin, fully-trimmed, raw | Healthy | Healthy |
| Lamb, fillet or tenderloin, fully-trimmed, baked, roasted, fried, stir-fried, grilled or BBQ'd, no added fat | Healthy | Healthy |
| Lamb, forequarter (easy carve shoulder, forequarter chop), separable fat, raw | Healthy | Healthy |
| Lamb, forequarter (easy carve shoulder, forequarter chop), separable fat, grilled or BBQ'd, no added fat | Healthy | Healthy |
| Lamb, forequarter chop, separable lean, raw | Healthy | Healthy |
| Lamb, forequarter chop, separable lean, grilled or BBQ'd, no added fat | Healthy | Healthy |
| Lamb, frenched cutlet/rack, separable lean, raw | Healthy | Healthy |
| Lamb, frenched cutlet/rack, separable lean, grilled or BBQ'd, no added fat | Healthy | Healthy |
| Lamb, leg (leg roast, mini roast, chump chop), separable fat, raw | Healthy | Healthy |
| Lamb, leg (leg roast, mini roast, chump chop), separable fat, baked or roasted, no added fat | Healthy | Healthy |
| Lamb, leg roast, separable lean, raw | Healthy | Healthy |
| Lamb, leg roast, separable lean, baked or roasted, no added fat | Healthy | Healthy |
| Lamb, loin chop, separable lean, raw | Healthy | Healthy |
| Lamb, loin chop, separable lean, grilled or BBQ'd, no added fat | Healthy | Healthy |
| Lamb, loin, separable fat, raw | Healthy | Healthy |
| Lamb, loin, separable fat, grilled or BBQ'd, no added fat | Healthy | Healthy |
| Lamb, mince, raw | Healthy | Healthy |
| Lamb, mince, baked, roasted, fried, stir-fried, grilled or BBQ'd, no added fat | Healthy | Healthy |
| Lamb, mini roast, separable lean, raw | Healthy | Healthy |
| Lamb, mini roast, separable lean, baked or roasted, no added fat | Healthy | Healthy |
| Lamb, roasting cuts, untrimmed, baked or roasted, no added fat | Healthy | Healthy |
| Lamb, roasting cuts, untrimmed, boiled, casseroled, microwaved, poached, steamed or stewed, no added fat | Healthy | Healthy |
| Lamb, rump, separable lean, raw | Healthy | Healthy |
| Lamb, rump, separable lean, grilled or BBQ'd, no added fat | Healthy | Healthy |
| Lamb, steak, separable lean, raw | Healthy | Healthy |
| Lamb, steak, separable lean, baked or roasted, no added fat | Healthy | Healthy |
| Lamb, stir-fry strips, separable lean, raw | Healthy | Healthy |
| Lamb, stir-fry strips, separable lean, fried or stir fried, no added fat | Healthy | Healthy |
| Mutton, all cuts, separable fat, raw | Healthy | Healthy |
| Mutton, all cuts, separable fat, cooked | Healthy | Healthy |
| Mutton, shoulder, separable fat, raw | Healthy | Healthy |
| Mutton, shoulder, separable fat, boiled, casseroled, microwaved, poached, steamed, or stewed, no added fat | Healthy | Healthy |
| Mutton, shoulder, separable lean, raw | Healthy | Healthy |
| Mutton, shoulder, separable lean, boiled, casseroled, microwaved, poached, steamed, or stewed, no added fat | Healthy | Healthy |
| Mutton, leg roast, separable fat, raw | Healthy | Healthy |
| Mutton, leg roast, separable fat, baked or roasted, no added fat | Healthy | Healthy |
| Mutton, leg roast, separable lean, raw | Healthy | Healthy |
| Mutton, leg roast, separable lean, baked or roasted, no added fat | Healthy | Healthy |
| Pork, belly, baked, roasted, fried, grilled or BBQ'd, no added fat | Healthy | Healthy |
| Pork, belly, boiled, casseroled, microwaved, poached, steamed or stewed, no added fat | Healthy | Healthy |
| Pork, butterfly steak, fully-trimmed, raw | Healthy | Healthy |
| Pork, butterfly steak, fully-trimmed, baked, roasted, fried, grilled or BBQ'd, no added fat | Healthy | Healthy |
| Pork, crackling, roasted, salted | Unhealthy | Unhealthy |
| Pork, diced, fully-trimmed, raw | Healthy | Healthy |
| Pork, diced, fully-trimmed, baked, roasted, fried, stir-fried, grilled or BBQ'd, no added fat | Healthy | Healthy |
| Pork, diced, fully-trimmed, boiled, casseroled, microwaved, poached, steamed or stewed, no added fat | Healthy | Healthy |
| Pork, fillet, fully-trimmed, raw | Healthy | Healthy |
| Pork, fillet, fully-trimmed, baked, roasted, fried, grilled or BBQ'd, no added fat | Healthy | Healthy |
| Pork, fillet, fully-trimmed, boiled, casseroled, microwaved, poached, steamed or stewed, no added fat | Healthy | Healthy |
| Pork, forequarter (chop, roast, neck), separable fat, raw | Healthy | Healthy |
| Pork, forequarter, separable fat, grilled or BBQ'd, no added fat | Healthy | Healthy |
| Pork, forequarter chop, fully-trimmed, raw | Healthy | Healthy |
| Pork, forequarter chop, fully-trimmed, baked, roasted, fried, grilled or BBQ'd, no added fat | Healthy | Healthy |
| Pork, forequarter shoulder roast, fully-trimmed, raw | Healthy | Healthy |
| Pork, forequarter shoulder roast, fully-trimmed, baked or roasted, no added fat | Healthy | Healthy |
| Pork, head, cooked, with or without added fat | Healthy | Healthy |
| Pork, leg steak (rump), separable fat, raw | Healthy | Healthy |
| Pork, leg steak (rump), separable fat, fried or stir fried, no added fat | Healthy | Healthy |
| Pork, leg steak (round, rump, topside, silverside), separable lean, raw | Healthy | Healthy |
| Pork, leg steak (round, rump, topside, silverside), separable lean, fried or stir fried, no added fat | Healthy | Healthy |
| Pork, loin chop, separable fat, raw | Healthy | Healthy |
| Pork, loin chop, separable fat, baked, roasted, fried, grilled or BBQ'd, no added fat | Healthy | Healthy |
| Pork, loin chop, fully-trimmed, raw | Healthy | Healthy |
| Pork, loin chop, fully-trimmed, baked, roasted, fried, grilled or BBQ'd, no added fat | Healthy | Healthy |
| Pork, loin chop, fully-trimmed, boiled, casseroled, microwaved, poached, steamed or stewed, no added fat | Healthy | Healthy |
| Pork, loin chop, semi-trimmed, boiled, casseroled, microwaved, poached, steamed or stewed, no added fat | Healthy | Healthy |
| Pork, loin cutlet, untrimmed, baked, roasted, fried, grilled or BBQ'd, no added fat | Healthy | Healthy |
| Pork, loin roast, separable fat, raw | Healthy | Healthy |
| Pork, loin roast, separable fat, baked or roasted, no added fat | Healthy | Healthy |
| Pork, loin roast, fully-trimmed, raw | Healthy | Healthy |
| Pork, loin roast, fully-trimmed, baked or roasted, no added fat | Healthy | Healthy |
| Pork, loin roast, fully-trimmed, boiled, casseroled, microwaved, poached, steamed, or stewed, no added fat | Healthy | Healthy |
| Pork, medallion or loin steak, separable fat, raw | Healthy | Healthy |
| Pork, medallion or loin steak, separable fat, fried, grilled or BBQ'd, no added fat | Healthy | Healthy |
| Pork, medallion or loin steak, fully-trimmed, raw | Healthy | Healthy |
| Pork, medallion or loin steak, fully-trimmed, baked, roasted, fried, grilled or BBQ'd, no added fat | Healthy | Healthy |
| Pork, mince, raw | Healthy | Healthy |
| Pork, mince, baked, roasted, fried or stir-fried, grilled or BBQ'd, no added fat | Healthy | Healthy |
| Pork, mince, boiled, casseroled, microwaved, poached, steamed or stewed, no added fat | Healthy | Healthy |
| Pork, round mini roast, separable fat, raw | Healthy | Healthy |
| Pork, round mini roast, separable fat, baked or roasted, no added fat | Healthy | Healthy |
| Pork, round mini roast, fully-trimmed, raw | Healthy | Healthy |
| Pork, round mini roast, fully-trimmed, baked or roasted, no added fat | Healthy | Healthy |
| Pork, round steak, fully-trimmed, raw | Healthy | Healthy |
| Pork, round steak, fully-trimmed, baked, roasted, fried, grilled or BBQ'd, no added fat | Healthy | Healthy |
| Pork, rump steak, fully-trimmed, raw | Healthy | Healthy |
| Pork, rump steak, fully-trimmed, baked, roasted, fried, grilled or BBQ'd no added fat | Healthy | Healthy |
| Pork, scotch roast, separable fat, raw | Healthy | Healthy |
| Pork, scotch roast, separable fat, baked or roasted, no added fat | Healthy | Healthy |
| Pork, scotch roast, fully-trimmed, raw | Healthy | Healthy |
| Pork, scotch roast, fully-trimmed, baked or roasted, no added fat | Healthy | Healthy |
| Pork, silverside steak, fully-trimmed, raw | Healthy | Healthy |
| Pork, silverside steak, fully-trimmed, baked, roasted, fried, grilled or BBQ'd, no added fat | Healthy | Healthy |
| Pork, spare ribs, semi-trimmed, baked, roasted, fried, grilled or BBQ'd, no added fat | Healthy | Healthy |
| Pork, spare ribs, semi-trimmed, boiled, casseroled, microwaved, poached, steamed, or stewed, no added fat | Healthy | Healthy |
| Pork, spare ribs, untrimmed, baked, roasted, fried, grilled or BBQ'd, no added fat | Healthy | Healthy |
| Pork, spare ribs, untrimmed, boiled, casseroled, microwaved, poached, steamed or stewed, no added fat | Healthy | Healthy |
| Pork, strips, fully-trimmed, raw | Healthy | Healthy |
| Pork, strips, fully-trimmed, baked, roasted, fried, stir-fried, grilled or BBQ'd, no added fat | Healthy | Healthy |
| Pork, topside steak, fully-trimmed, raw | Healthy | Healthy |
| Pork, topside steak, fully-trimmed, baked, roasted, fried, grilled or BBQ'd, no added fat | Healthy | Healthy |
| Veal, all cuts, separable fat, raw | Healthy | Healthy |
| Veal, all cuts, separable fat, cooked | Healthy | Healthy |
| Veal, cutlet, separable lean, raw | Healthy | Healthy |
| Veal, cutlet, separable lean, fried, grilled or BBQ'd, no added fat | Healthy | Healthy |
| Veal, diced, separable lean, raw | Healthy | Healthy |
| Veal, diced, separable lean, fried, stir-fried, grilled or BBQ'd, no added fat | Healthy | Healthy |
| Veal, leg roast, separable lean, raw | Healthy | Healthy |
| Veal, leg roast, separable lean, fried, grilled or BBQ'd, no added fat | Healthy | Healthy |
| Veal, leg steak, separable lean, raw | Healthy | Healthy |
| Veal, leg steak, separable lean, fried, grilled or BBQ'd, no added fat | Healthy | Healthy |
| Veal, loin chop, separable fat, raw | Healthy | Healthy |
| Veal, loin chop, separable fat, fried, grilled or BBQ'd, no added fat | Healthy | Healthy |
| Veal, loin chop, separable lean, raw | Healthy | Healthy |
| Veal, loin chop, separable lean, fried, grilled or BBQ'd, no added fat | Healthy | Healthy |
| Veal, stir-fry strips, separable lean, raw | Healthy | Healthy |
| Veal, stir-fry strips, separable lean, fried, stir-fried, grilled or BBQ'd, no added fat | Healthy | Healthy |
| Kangaroo, loin fillet, raw | Healthy | Healthy |
| Kangaroo, loin fillet, baked, roasted, fried, grilled or BBQ'd, no added fat | Healthy | Healthy |
| Kangaroo, rump, raw | Healthy | Healthy |
| Kangaroo, rump, baked, roasted, fried, grilled or BBQ'd, no added fat | Healthy | Healthy |
| Kangaroo, tail, raw | Healthy | Healthy |
| Kangaroo, tail, cooked | Healthy | Healthy |
| Buffalo, riverine, cube roll, raw | Healthy | Healthy |
| Buffalo, riverine, topside, raw | Healthy | Healthy |
| Buffalo, swamp, cube roll, raw | Healthy | Healthy |
| Buffalo, swamp, topside, raw | Healthy | Healthy |
| Camel, cube roll, raw | Healthy | Healthy |
| Camel, rump, raw | Healthy | Healthy |
| Goat, forequarter, separable lean, raw | Healthy | Healthy |
| Goat, separable fat (composite), raw | Healthy | Healthy |
| Rabbit, farmed, whole, raw | Healthy | Healthy |
| Rabbit, flesh, boiled, casseroled, microwaved, poached, steamed, or stewed, no added fat | Healthy | Healthy |
| Venison, diced, lean, raw | Healthy | Healthy |
| Venison, diced, lean, baked, roasted, fried, stir-fried, grilled or BBQ'd, no added fat | Healthy | Healthy |
| Venison, leg medallion, lean, raw | Healthy | Healthy |
| Venison, leg medallion, lean, baked, roasted, fried, stir-fried, grilled or BBQ'd, no added fat | Healthy | Healthy |
| Venison, mince, premium, raw | Healthy | Healthy |
| Venison, mince, premium, baked, roasted, fried, stir-fried, grilled or BBQ'd, no added fat | Healthy | Healthy |
| Venison, stir fry strips, lean, raw | Healthy | Healthy |
| Venison, stir fry strips, lean, baked, roasted, fried, stir-fried, grilled or BBQ'd, no added fat | Healthy | Healthy |
| Chicken, breast, flesh, raw | Healthy | Healthy |
| Chicken, breast, flesh, baked or roasted, no added fat | Healthy | Healthy |
| Chicken, breast, flesh, grilled or BBQ'd, no added fat | Healthy | Healthy |
| Chicken, breast, flesh, fried or stir-fried, no added fat | Healthy | Healthy |
| Chicken, breast, flesh, boiled, casseroled, microwaved, poached, steamed or stewed, no added fat | Healthy | Healthy |
| Chicken, drumstick, flesh, raw | Healthy | Healthy |
| Chicken, drumstick, flesh, baked, roasted, fried, grilled or BBQ'd, no added fat | Healthy | Healthy |
| Chicken, drumstick, flesh, boiled, casseroled, microwaved, poached, steamed or stewed, no added fat | Healthy | Healthy |
| Chicken, feet, boiled, casseroled, microwaved, poached or steamed, with or without added fat | Healthy | Healthy |
| Chicken, maryland, flesh, baked, roasted, fried, grilled or BBQ'd, no added fat | Healthy | Healthy |
| Chicken, mince, raw | Healthy | Healthy |
| Chicken, mince, baked, roasted, fried, stir-fried, grilled or BBQ'd, no added fat | Healthy | Healthy |
| Chicken, thigh, flesh, raw | Healthy | Healthy |
| Chicken, thigh, flesh, baked, roasted, fried, grilled or BBQ'd, no added fat | Healthy | Healthy |
| Chicken, thigh, flesh, boiled, casseroled, microwaved, poached, steamed or stewed, with or without added fat | Healthy | Healthy |
| Chicken, wing, flesh, raw | Healthy | Healthy |
| Chicken, wing, flesh, baked, roasted, fried, grilled or BBQ'd, no added fat | Healthy | Healthy |
| Chicken, wing, flesh, boiled, casseroled, microwaved, poached, steamed or stewed, with or without added fat | Healthy | Healthy |
| Chicken, whole, flesh, skin & fat, baked, roasted, fried, grilled or BBQ'd, no added fat | Healthy | Healthy |
| Chicken, flesh, baked, roasted, fried, grilled or BBQ'd, no added fat | Healthy | Healthy |
| Chicken, separable fat, composite, raw | Healthy | Healthy |
| Chicken, separable fat, composite, boiled, casseroled, microwaved, poached, steamed or stewed, no added fat | Healthy | Healthy |
| Chicken, separable fat, composite, baked or roasted, no added fat | Healthy | Healthy |
| Chicken, skin, composite, raw | Healthy | Healthy |
| Chicken, skin, composite, baked or roasted, no added fat | Healthy | Healthy |
| Chicken, skin, composite, boiled, casseroled, microwaved, poached, steamed or stewed, no added fat | Healthy | Healthy |
| Chicken, for use as subway sandwich filling | Healthy | Healthy |
| Duck, lean, raw | Healthy | Healthy |
| Duck, lean, baked, roasted, fried, grilled or BBQ'd, no added fat | Healthy | Healthy |
| Duck, lean, boiled, casseroled, microwaved, poached, steamed or stewed, no added fat | Healthy | Healthy |
| Duck, skin & fat, raw | Healthy | Healthy |
| Duck, skin & fat, baked or roasted, fried, grilled or BBQ'd, no added fat | Healthy | Healthy |
| Duck, lean, skin & fat, baked, roasted, fried, grilled or BBQ'd, no added fat | Healthy | Healthy |
| Turkey, breast, lean, raw | Healthy | Healthy |
| Turkey, breast, lean, baked, roasted, fried, grilled or BBQ'd, no added fat | Healthy | Healthy |
| Turkey, breast, lean, skin & fat, raw | Healthy | Healthy |
| Turkey, breast, lean, skin & fat, baked, roasted, fried, grilled or BBQ'd, no added fat | Healthy | Healthy |
| Turkey, hindquarter, lean, raw | Healthy | Healthy |
| Turkey, hindquarter, lean, baked, roasted, fried, grilled or BBQ'd, no added fat | Healthy | Healthy |
| Turkey, hindquarter, lean, skin & fat, raw | Healthy | Healthy |
| Turkey, hindquarter, lean, skin & fat, baked, roasted, fried, grilled or BBQ'd, no added fat | Healthy | Healthy |
| Emu, fan fillet, raw | Healthy | Healthy |
| Emu, steak, raw | Healthy | Healthy |
| Mutton-bird, cooked | Healthy | Healthy |
| Ostrich, fan fillet, raw | Healthy | Healthy |
| Ostrich, moon steak, raw | Healthy | Healthy |
| Pigeon (squab), whole, raw | Healthy | Healthy |
| Quail, flesh & skin, raw | Healthy | Healthy |
| Quail, flesh & skin, baked, roasted, fried, grilled or BBQ'd, no added fat | Healthy | Healthy |
| Beef, liver, raw | Healthy | Healthy |
| Beef, liver, simmered or boiled, no added fat | Healthy | Healthy |
| Chicken, liver, raw | Healthy | Healthy |
| Lamb, liver, raw | Healthy | Healthy |
| Lamb, liver, fried, grilled or BBQ'd, no added fat | Healthy | Healthy |
| Veal, liver, raw | Healthy | Healthy |
| Veal, liver, fried, grilled or BBQ'd, no added fat | Healthy | Healthy |
| Beef, kidney, raw | Healthy | Healthy |
| Beef, kidney, simmered or boiled, no added fat | Healthy | Healthy |
| Lamb, kidney, raw | Healthy | Healthy |
| Lamb, kidney, simmered or boiled, no added fat | Healthy | Healthy |
| Veal, kidney, raw | Healthy | Healthy |
| Veal, kidney, fried, grilled or BBQ'd, no added fat | Healthy | Healthy |
| Beef, heart, raw | Healthy | Healthy |
| Beef, heart, simmered or boiled, no added fat | Healthy | Healthy |
| Beef, tail, raw | Healthy | Healthy |
| Beef, tail, simmered or boiled, no added fat | Healthy | Healthy |
| Beef, tongue, raw | Healthy | Healthy |
| Beef, tongue, simmered or boiled, no added fat | Healthy | Healthy |
| Beef, tripe, raw | Healthy | Healthy |
| Beef, tripe, simmered or boiled, no added fat | Healthy | Healthy |
| Lamb, brain, raw | Healthy | Healthy |
| Lamb, brain, simmered or boiled, no added fat | Healthy | Healthy |
| Lamb, heart, raw | Healthy | Healthy |
| Lamb, heart, baked or roasted, no added fat | Healthy | Healthy |
| Lamb, tongue, raw | Healthy | Healthy |
| Lamb, tongue, simmered or boiled, no added fat | Healthy | Healthy |
| Veal, heart, raw | Healthy | Healthy |
| Veal, heart, baked or roasted, no added fat | Healthy | Healthy |
| Lamb, intestine, simmered or boiled, no added fat | Healthy | Healthy |
| Crocodile, tail fillet, raw | Healthy | Healthy |
| Crocodile, back leg, raw | Healthy | Healthy |
| Crocodile, cooked | Healthy | Healthy |
| Goanna, wild caught, flesh, cooked | Healthy | Healthy |
| Turtle, wild caught, flesh, raw | Healthy | Healthy |
| Turtle, wild caught, flesh, cooked | Healthy | Healthy |
| Insect, wild caught, raw | Healthy | Healthy |
| **Meat alternatives** | | |
| Vegetable Patties with Quinoa | Unhealthy | Unhealthy |
| Vegetarian Sausages | Unhealthy | Unhealthy |
| Nutmeat | Unhealthy | Unhealthy |
| Nutolene | Healthy | Healthy |
| Casserole Mince in Gravy | Unhealthy | Unhealthy |
| Classic Veggie Burger | Healthy | Healthy |
| Cheese & Veggie Burger | Unhealthy | Healthy |
| Chickpea & Lentil Burger | Unhealthy | Healthy |
| Organic Veggie Patties | Unhealthy | Unhealthy |
| Mixed Vegetable Veggie Burgers | Unhealthy | Healthy |
| Tomato, Onion & Basil Vegetable Sausages | Unhealthy | Unhealthy |
| Vegie Roast | Unhealthy | Unhealthy |
| 100% Natural Vegie Boost Burgers | Healthy | Healthy |
| Cheese & Spinach Schnitzels | Unhealthy | Unhealthy |
| 100% Natural Vegie and Lentil Burgers | Healthy | Healthy |
| Quinoa & Brown Rice Protein Burgers | Unhealthy | Unhealthy |
| Chunks Lightly Smoked | Unhealthy | Unhealthy |
| Chunks with Zesty Lime & Chilli | Unhealthy | Unhealthy |
| Chunks with Lemon & Thyme | Unhealthy | Unhealthy |
| Natural Veggie Burgers | Unhealthy | Unhealthy |
| Chickpea & Sunflower Seeds Vegetable Burgers | Healthy | Healthy |
| Japanese Tofu | Unhealthy | Healthy |
| Vegie Sausages | Unhealthy | Unhealthy |
| 6 Vegetarian Red Onion & Rosemary Sausages | Unhealthy | Unhealthy |
| Vegan Fillets | Unhealthy | Unhealthy |
| Tender Crumbed Schnitzel | Unhealthy | Unhealthy |
| Mediterranean Koftas | Unhealthy | Unhealthy |
| Tofu Puffs | Healthy | Healthy |
| Jumbo Hot Dogs | Unhealthy | Unhealthy |
| Original Sausage Beer Brats | Unhealthy | Unhealthy |
| Easy Prep Chicken Free Satay | Unhealthy | Unhealthy |
| Medium Classic Tofu | Healthy | Healthy |
| 100% Plant Based Mince | Unhealthy | Unhealthy |
| Crispy Chicken-Style Burger | Unhealthy | Unhealthy |
| Vegie Pakoras | Unhealthy | Unhealthy |
| Bacon Flavoured Chips | Unhealthy | Unhealthy |
| Plant Based 100% Vegetarian Burgers | Unhealthy | Unhealthy |
| Mexican Plant Based Protein Patty Mix | Unhealthy | Unhealthy |
| Meat Free Coconut Bacon | Healthy | Healthy |
| Smooth Tofu | Healthy | Healthy |
| Asian Inspired Sweetcorn Fritters | Unhealthy | Unhealthy |
| Fishless Fillets | Unhealthy | Unhealthy |
| Falafel | Unhealthy | Unhealthy |
| Chicken Style Meat-Free Tenders | Unhealthy | Unhealthy |
| Original Meat-Free Sausages | Unhealthy | Unhealthy |
| Smokey Grill Meat-Free Burgers | Unhealthy | Unhealthy |
| Mixed Garden Vegetable Koftas | Unhealthy | Unhealthy |
| Organic Silken Tofu | Healthy | Healthy |
| Southern Smoke Infused Tofu | Unhealthy | Unhealthy |
| Mandarin Orange Crispy Chick'n | Unhealthy | Unhealthy |
| Seven Grain Crispy Tenders | Unhealthy | Unhealthy |
| Chicken Free Chicken Wild Meaty Chunks | Unhealthy | Unhealthy |
| Silken Tofu | Healthy | Healthy |
| Lightly Battered Prawns | Unhealthy | Healthy |
| Lightly Crumbed Scallops | Unhealthy | Healthy |
| Chick'n Schnitzel | Unhealthy | Unhealthy |
| Italian Beefy Burger | Unhealthy | Unhealthy |
| Perfectly Firm Tofu | Healthy | Healthy |
| Naked Prawns | Unhealthy | Unhealthy |
| Lightly Seasoned Prawns | Unhealthy | Unhealthy |
| Vegan Smoked Salmon | Unhealthy | Unhealthy |
| Organic Red Jackfruit Meat | Healthy | Healthy |
| Traditional Style Chick'n Nuggets | Unhealthy | Unhealthy |
| Traditional Style Fried Chick'n Pieces | Unhealthy | Unhealthy |
| Southern Spice Chick'n Burger | Unhealthy | Unhealthy |
| Golden Fishless Filets | Unhealthy | Unhealthy |
| Chicken-Free Nuggets | Unhealthy | Unhealthy |
| Plant-Based Roast Duck | Unhealthy | Unhealthy |
| Plant-Based Roast Pork | Unhealthy | Unhealthy |
| Italian Eatballs | Unhealthy | Unhealthy |
| Sticky Teriyaki | Unhealthy | Unhealthy |
| Plant Powered BBQ Gourmet Burgers | Unhealthy | Unhealthy |
| Plant Powered BBQ Gourmet Snags | Unhealthy | Unhealthy |
| Meat alternative, commercial, cooked, no added fat | Unhealthy | Unhealthy |
| Sausage, vegetarian style, raw | Unhealthy | Unhealthy |
| Sausage, vegetarian style, added Fe, Zn and vitamin B12, raw | Unhealthy | Unhealthy |
| Soy bean curd skins, dried, rehydrated in boiling water | Unhealthy | Healthy |
| Tempeh (fermented soy beans), fried | Unhealthy | Unhealthy |
| Tofu (soy bean curd), firm, as purchased | Healthy | Healthy |
| Tofu (soy bean curd), silken or soft, as purchased | Healthy | Healthy |
| Tofu (soy bean curd), smoked, as purchased | Unhealthy | Unhealthy |
| Tofu (soy bean curd), fried, stir-fried, grilled or BBQ'd, no added fat | Healthy | Healthy |
| Tofu (soy bean curd), burger patty, as purchased | Unhealthy | Unhealthy |
| **Processed meat** | | |
| Breast Tenders | Unhealthy | Unhealthy |
| Breast Nuggets | Unhealthy | Unhealthy |
| Honey Mustard Chicken Schnitzel | Unhealthy | Unhealthy |
| Mexican Flavour Chicken Breast Tenders | Unhealthy | Unhealthy |
| Lime & Black Pepper Salami | Unhealthy | Unhealthy |
| Spicy Chicken Breast Chunks | Unhealthy | Unhealthy |
| Sliced Naturally Smoked Ham | Unhealthy | Unhealthy |
| Cocktail Tasty BBQ Chicken Kebabs | Unhealthy | Unhealthy |
| Texas BBQ Wing Nibbles | Unhealthy | Unhealthy |
| Devilled Meat Savoury Spread | Unhealthy | Unhealthy |
| Australian Crocodile Sausages | Unhealthy | Unhealthy |
| Smokey Barbeque Air-Dried Beef Snack | Unhealthy | Unhealthy |
| Traditional Flavour Biltong Beef Jerky | Unhealthy | Unhealthy |
| Chilli Flavour Biltong Beef Jerky | Unhealthy | Unhealthy |
| Chilli Beef Jerky | Unhealthy | Unhealthy |
| Schinkengriller | Unhealthy | Unhealthy |
| Black Truffle Duck Paté | Healthy | Healthy |
| Chicken & Madeira Pâté | Unhealthy | Healthy |
| Chicken & Orange Paté | Unhealthy | Unhealthy |
| Chicken Liver Pâte with Brandy Port & Sage | Unhealthy | Unhealthy |
| Kangaroo Jerky | Unhealthy | Unhealthy |
| Parsley & Onion Chicken Burgers | Unhealthy | Unhealthy |
| Australian Angus Beef Burgers with Ale & Shallot | Unhealthy | Healthy |
| Real Bacon Pieces | Unhealthy | Unhealthy |
| Australian Wild Boar Sausages | Unhealthy | Unhealthy |
| Teriyaki Beef Biltong | Unhealthy | Unhealthy |
| Shredded Chicken | Unhealthy | Unhealthy |
| Free Range Chicken Thyme & Garlic Rillette | Unhealthy | Unhealthy |
| Berkshire Pork Thyme & Garlic Rillette | Unhealthy | Unhealthy |
| Duck & Shiraz Pâté | Healthy | Healthy |
| Kangaroo Game Jerky | Unhealthy | Unhealthy |
| Sticky Toffee Bourbon Wings | Unhealthy | Unhealthy |
| Chicken Sandwich Spread | Unhealthy | Unhealthy |
| Barbecue Chicken Bites | Unhealthy | Unhealthy |
| Asian Style Mushroom & Pork Balls | Unhealthy | Unhealthy |
| Slow Cooked Beef with Onion Gravy | Unhealthy | Unhealthy |
| Smoked Streaky Bacon | Unhealthy | Unhealthy |
| Pork & Leek Sausages | Unhealthy | Unhealthy |
| Country Style Chicken Hot Roast | Unhealthy | Unhealthy |
| Pork Spare Rib | Unhealthy | Unhealthy |
| Premium Unsmoked Sliced Ham | Unhealthy | Unhealthy |
| Honey Soy Chicken Breast Tenders | Unhealthy | Unhealthy |
| Pheasant Farm Pate | Unhealthy | Unhealthy |
| Duck & Orange Pate | Unhealthy | Unhealthy |
| Duck & Green Peppercorn Pate | Unhealthy | Unhealthy |
| Pulled Chicken | Unhealthy | Unhealthy |
| Spicy Beef Jerky | Unhealthy | Unhealthy |
| Fresh Organic Chicken Sausages | Unhealthy | Unhealthy |
| Chicken Breast Burger | Unhealthy | Unhealthy |
| Roast Chicken Bites | Unhealthy | Unhealthy |
| Crumbed Chicken Breast Goujons | Unhealthy | Unhealthy |
| Chicken Spread | Unhealthy | Unhealthy |
| Chicken & Cheese Mega Schnitzel | Unhealthy | Unhealthy |
| Chicken Breast Crumbed Tenders | Unhealthy | Unhealthy |
| Champagne Leg Ham | Unhealthy | Unhealthy |
| Kentucky Style Natural Chicken Thigh Fillet | Unhealthy | Unhealthy |
| Southern Style Chicken Patties | Unhealthy | Unhealthy |
| Teriyaki Beef Jerky Meat Snacks | Unhealthy | Unhealthy |
| Garlic Wurst | Unhealthy | Unhealthy |
| German Bratwurst | Unhealthy | Unhealthy |
| Pork Krackles | Unhealthy | Unhealthy |
| Classic Sea Salt Grass-Fed Beef Jerky | Unhealthy | Unhealthy |
| Smoked Chipotle Grass-Fed Beef Jerky | Unhealthy | Unhealthy |
| Flamin Hot Beef Jerky | Unhealthy | Unhealthy |
| Chicken Liver Páte with Sweet Chilli | Unhealthy | Unhealthy |
| Pork Mince with San Choy Bau Seasoning | Unhealthy | Unhealthy |
| Chilli Biltong Cured & Air Dried Beef Slices | Unhealthy | Unhealthy |
| Old Fashioned Smoked Biltong Cured & Air Dried Beef Slices | Unhealthy | Unhealthy |
| Traditional Biltong Cured & Air Dried Beef Slices | Unhealthy | Unhealthy |
| Crispy Cracker Crumbed Chicken Tenders | Unhealthy | Unhealthy |
| Original Beef Jerky | Unhealthy | Unhealthy |
| Peppered Beef Steakettes | Unhealthy | Unhealthy |
| Pork Meatloaf | Unhealthy | Unhealthy |
| Honey Mustard Chicken Drumsticks | Unhealthy | Unhealthy |
| Duck, Fig & Ginger Pate | Unhealthy | Unhealthy |
| Free Range Chicken Liver Pâté | Unhealthy | Unhealthy |
| Duck & Cherry Pâté | Unhealthy | Unhealthy |
| Teriyaki Flavour Cured & Air Dried Beef Slices | Unhealthy | Unhealthy |
| Sea Salt & Vinegar Flavour Biltong Cured & Air Dried Beef Slices | Unhealthy | Unhealthy |
| Wood Smoked Beef Ribs with Sweet 'n' Smokey BBQ Sauce | Unhealthy | Unhealthy |
| Hot Dogs | Unhealthy | Unhealthy |
| Habanero Chilli Grass-Fed Beef Jerky | Unhealthy | Unhealthy |
| Half Chicken with French Garlic Marinade | Unhealthy | Unhealthy |
| Traditional Leg Ham Home Style Cuts | Unhealthy | Unhealthy |
| Barbecue Flavour Premium Beef Biltong Snack | Unhealthy | Unhealthy |
| Souvlaki Chicken Breast Strips | Unhealthy | Unhealthy |
| Rabbit & Confit Onion Terrine | Unhealthy | Unhealthy |
| Free Range Chicken, Leek & Truffle Terrine | Healthy | Healthy |
| Hand Cooked Pork Crackling Sea Salt | Unhealthy | Unhealthy |
| Original Beef Biltong | Unhealthy | Unhealthy |
| Duck & Orange Paté | Unhealthy | Unhealthy |
| Australian Lamb Slow Cooked Shoulder with Greek Inspired Sauce | Unhealthy | Unhealthy |
| Buffalo Chicken Wing Nibbles | Unhealthy | Unhealthy |
| Boneless Chicken Mini Roast with Garlic Bread Stuffing | Unhealthy | Unhealthy |
| Slow Cooked Pulled BBQ Beef | Unhealthy | Unhealthy |
| Chicken Breast Fillets with Ginger, Chilli & Coriander | Unhealthy | Unhealthy |
| Chicken Breast Nuggets Coated in a Ciabatta Crumb | Unhealthy | Unhealthy |
| Smokey BBQ Gourmet Beef Jerky | Unhealthy | Unhealthy |
| Hot Gourmet Beef Jerky | Unhealthy | Unhealthy |
| Spicy Bratwurst Sausages | Unhealthy | Unhealthy |
| Chicken & Cardonnay Pate | Unhealthy | Unhealthy |
| Pork and Red Wine Pate | Unhealthy | Unhealthy |
| Chicken Pate with Black Pepper | Unhealthy | Unhealthy |
| Chicken & Gin Pate | Unhealthy | Unhealthy |
| All Natural Biltong Beef Jerky | Unhealthy | Unhealthy |
| Original Mini Posh Dogs | Unhealthy | Unhealthy |
| Cheese & Bacon Posh Dogs | Unhealthy | Unhealthy |
| Teriyaki Flavoured Chicken Leg Steaks | Unhealthy | Unhealthy |
| Premium Free Range Chicken | Unhealthy | Unhealthy |
| Cracked Pepper Pate | Unhealthy | Unhealthy |
| Turkey & Cranberry Pate | Unhealthy | Unhealthy |
| Small Fresh Turkey Buffet | Unhealthy | Unhealthy |
| Chicken Paté with Black Peppercorns | Unhealthy | Unhealthy |
| Chicken Paté with Cognac | Unhealthy | Unhealthy |
| Chicken Paté with Champagne | Unhealthy | Unhealthy |
| Chicken Paté with Cointreau | Unhealthy | Unhealthy |
| Cinnamon & Apple Pork Crackle | Unhealthy | Unhealthy |
| Maple Bacon Flavour Pork Crackle | Unhealthy | Unhealthy |
| Smokey Premium Air-Dried Jerky | Unhealthy | Unhealthy |
| Beef Meatballs | Unhealthy | Unhealthy |
| Buffalo Chicken Wings | Unhealthy | Unhealthy |
| Lemon Pepper Smashin' Chicken Fillets | Unhealthy | Healthy |
| Brandy Port & Sage Pâté | Unhealthy | Unhealthy |
| Sweet BBQ Glaze Chicken Drumsticks | Unhealthy | Unhealthy |
| Chicken Parsley Sausages | Unhealthy | Unhealthy |
| Beefy Classics | Unhealthy | Unhealthy |
| All Beef Naturally Wood Smoked | Unhealthy | Unhealthy |
| Free Range Turkey | Unhealthy | Unhealthy |
| Shaved Roast Chicken | Unhealthy | Unhealthy |
| Dry Cured Streaky Bacon | Unhealthy | Unhealthy |
| Peri Peri Lekker Biltong Stokkies | Unhealthy | Unhealthy |
| Traditional Wood Smoked Thick Cut Off the Bone Ham | Unhealthy | Unhealthy |
| Italian Style Beef & Pork Sausages | Unhealthy | Unhealthy |
| Thinly Sliced Hungarian Salami | Unhealthy | Unhealthy |
| Wood Smoked Chorizo Salami | Unhealthy | Unhealthy |
| Wallaby Plum & Pepperberry Jerky | Unhealthy | Unhealthy |
| Cracked Pepper & Garlic Jerky | Unhealthy | Unhealthy |
| BBQ Rib Jerky | Unhealthy | Unhealthy |
| Dragons Tongue Jerky | Unhealthy | Unhealthy |
| Honey Jack Jerky | Unhealthy | Unhealthy |
| Chilli Beef Jerky | Unhealthy | Unhealthy |
| Premium Jerky Wagyu | Unhealthy | Unhealthy |
| Hot Peri Peri Beef Jerky | Unhealthy | Unhealthy |
| Cracked Pepper Free Range Pâté | Unhealthy | Unhealthy |
| Crumbed Chicken Ribs | Unhealthy | Unhealthy |
| Grass Fed Gluten-Free Beef Sausages | Unhealthy | Unhealthy |
| Sweet Chilli Chicken Tenders | Unhealthy | Unhealthy |
| Frozen Whole Turkey | Unhealthy | Healthy |
| Ham Roast | Unhealthy | Unhealthy |
| Chicken & Bacon Pate | Unhealthy | Unhealthy |
| Chicken & Smoky Garlic Pate | Unhealthy | Unhealthy |
| Chicken, barbecued, with skin, commercial | Healthy | Healthy |
| Chicken, liver, fried, baked, grilled or BBQ'd, added fat | Healthy | Healthy |
| Pate de foie (chicken liver pate) | Unhealthy | Unhealthy |
| Pate, liverwurst, commercial | Unhealthy | Unhealthy |
| Black pudding, raw | Unhealthy | Unhealthy |
| Sausage, beef, raw | Unhealthy | Unhealthy |
| Sausage, beef, fried | Unhealthy | Unhealthy |
| Sausage, beef, grilled, BBQ'd or baked | Unhealthy | Unhealthy |
| Sausage, beef, flavoured, fried, grilled, BBQ'd or baked | Unhealthy | Unhealthy |
| Sausage, beef, plain or flavoured, boiled, casseroled, microwaved, poached, steamed, or stewed | Unhealthy | Unhealthy |
| Sausage, lamb, plain, fried, grilled, BBQ'd or baked | Unhealthy | Unhealthy |
| Sausage, lamb, flavoured, fried, grilled, BBQ'd or baked | Unhealthy | Unhealthy |
| Sausage, pork, raw | Unhealthy | Unhealthy |
| Sausage, pork, plain, fried | Unhealthy | Unhealthy |
| Sausage, pork, plain, grilled, BBQ'd or baked | Unhealthy | Unhealthy |
| Sausage, pork, flavoured, fried, grilled, BBQ'd or baked | Unhealthy | Unhealthy |
| Sausage, pork, plain or flavoured, boiled, casseroled, microwaved, poached, steamed or stewed | Unhealthy | Unhealthy |
| Sausage, deep fried, commercial | Unhealthy | Unhealthy |
| Frankfurt, cooked | Unhealthy | Unhealthy |
| Sausage, beef, plain or flavoured, reduced fat, raw | Unhealthy | Unhealthy |
| Sausage, beef, plain or flavoured, reduced fat, fried, grilled, BBQ'd or baked | Unhealthy | Healthy |
| Sausage, beef, plain or flavoured, reduced fat, boiled, casseroled, microwaved, poached, steamed or stewed | Unhealthy | Healthy |
| Sausage, chicken, plain, fried, grilled, BBQ'd or baked, with or without added fat | Unhealthy | Unhealthy |
| Sausage, chicken, flavoured, fried, grilled, BBQ'd or baked, with or without fat | Unhealthy | Unhealthy |
| Sausage, chicken, reduced fat, raw | Unhealthy | Unhealthy |
| Sausage, chicken, reduced fat, fried, grilled, BBQ'd or baked, no added fat | Unhealthy | Unhealthy |
| Sausage, chicken, reduced fat, fried, grilled, BBQ'd or baked, fat not further defined | Unhealthy | Unhealthy |
| Sausage, kangaroo, plain or flavoured, fried, grilled, BBQ'd or baked | Unhealthy | Unhealthy |
| Bacon, 97% fat free, raw | Unhealthy | Unhealthy |
| Bacon, 97% fat free, baked, roasted, fried, grilled or BBQ'd, with or without added fat | Unhealthy | Unhealthy |
| Bacon, breakfast rasher, raw | Unhealthy | Unhealthy |
| Bacon, breakfast rasher, baked, roasted or fried, no added fat | Unhealthy | Unhealthy |
| Bacon, breakfast rasher, grilled or BBQ'd, no added fat | Unhealthy | Unhealthy |
| Bacon, middle rasher or shortcut, fully-trimmed, raw | Unhealthy | Unhealthy |
| Bacon, middle rasher or shortcut, fully-trimmed, fried or stir-fried, no added fat | Unhealthy | Unhealthy |
| Bacon, middle rasher or shortcut, fully-trimmed, baked, roasted, grilled or BBQ'd, no added fat | Unhealthy | Unhealthy |
| Bacon, middle rasher, semi-trimmed, raw | Unhealthy | Unhealthy |
| Bacon, middle rasher, semi-trimmed, fried or stir-fried, no added fat | Unhealthy | Unhealthy |
| Bacon, middle rasher, semi-trimmed, baked, roasted, grilled or BBQ'd, no added fat | Unhealthy | Unhealthy |
| Bacon, middle rasher, semi-trimmed, boiled, casseroled, microwaved, poached, steamed or stewed, no added fat | Unhealthy | Unhealthy |
| Bacon, middle rasher, untrimmed, raw | Unhealthy | Unhealthy |
| Bacon, middle rasher, untrimmed, baked, roasted, fried, grilled or BBQ'd, no added fat | Unhealthy | Unhealthy |
| Bacon, middle rasher, untrimmed, boiled, casseroled, microwaved, poached, steamed or stewed, no added fat | Unhealthy | Unhealthy |
| Bacon, middle rasher, fat only, grilled or BBQ'd | Unhealthy | Unhealthy |
| Ham, leg, lean | Unhealthy | Unhealthy |
| Ham, leg, lean & fat | Unhealthy | Unhealthy |
| Ham, leg, lean & fat, canned | Unhealthy | Unhealthy |
| Ham, shoulder, lean & fat | Unhealthy | Unhealthy |
| Ham, shoulder, lean & fat, canned | Unhealthy | Unhealthy |
| Ham steak, raw | Unhealthy | Unhealthy |
| Ham steak, baked, roasted, fried, grilled or BBQ'd, no added fat | Unhealthy | Unhealthy |
| Prosciutto | Unhealthy | Unhealthy |
| Kabana or cabanossi | Unhealthy | Unhealthy |
| Salami, danish | Unhealthy | Unhealthy |
| Salami, hungarian | Unhealthy | Unhealthy |
| Salami, mettwurst | Unhealthy | Unhealthy |
| Salami, milano | Unhealthy | Unhealthy |
| Salami, pepperoni | Unhealthy | Unhealthy |
| Salami, not further defined | Unhealthy | Unhealthy |
| Sausage, chorizo, uncooked | Unhealthy | Unhealthy |
| Sausage, chorizo, cooked | Unhealthy | Unhealthy |
| Beef, corned, lean, cooked | Unhealthy | Unhealthy |
| Beef, corned, 50% trimmed, cooked | Unhealthy | Unhealthy |
| Beef, corned, 75% trimmed, cooked | Unhealthy | Unhealthy |
| Beef, corned, lean & fat, cooked | Unhealthy | Unhealthy |
| Berliner | Unhealthy | Unhealthy |
| Brawn | Unhealthy | Unhealthy |
| Devon, processed luncheon meat | Unhealthy | Unhealthy |
| Garlic roll | Unhealthy | Unhealthy |
| Ham & chicken roll, processed luncheon meat | Unhealthy | Unhealthy |
| Mortadella, processed meat | Unhealthy | Unhealthy |
| Polish sausage | Unhealthy | Unhealthy |
| Strasburg | Unhealthy | Unhealthy |
| Chicken, processed luncheon meat, regular fat | Unhealthy | Unhealthy |
| Chicken, processed luncheon meat, low or reduced fat | Unhealthy | Unhealthy |
| Turkey, roast, deli-sliced | Unhealthy | Unhealthy |
| Turkey, processed luncheon meat | Unhealthy | Unhealthy |
| Chicken, breast, flesh, canned in water, drained | Unhealthy | Healthy |
| Beef, corned, canned | Unhealthy | Unhealthy |
| Beef, corned, with cereal, canned | Unhealthy | Unhealthy |
| Braised steak & onions, canned, heated | Unhealthy | Unhealthy |
| Camp pie, canned | Unhealthy | Unhealthy |
| Casserole or curry, meat & vegetables, canned, heated | Unhealthy | Healthy |
| Frankfurt, canned, heated, drained | Unhealthy | Unhealthy |
| Meat paste, commercial | Unhealthy | Unhealthy |
| Spam, canned | Unhealthy | Unhealthy |
| Stew, casserole or curry, chicken & vegetable, canned | Unhealthy | Unhealthy |
| Vegetables & sausages, canned | Unhealthy | Unhealthy |
| Vegetables & steak, canned | Unhealthy | Unhealthy |
| Jerky, beef, all flavours | Unhealthy | Unhealthy |
| Hamburger, no roll, beef patty, with cheese, fast food chain | Unhealthy | Unhealthy |
| Rissole or patty, beef mince, grilled or fried, no added fat, fast food style | Unhealthy | Unhealthy |
| Meatloaf, commercial, all meats | Unhealthy | Unhealthy |
| Chicken, breast, flesh, purchased frozen with breadcrumb coating, cooked, no added fat | Unhealthy | Unhealthy |
| Chicken, breast, flesh, purchased frozen with breadcrumb coating, cooked, fat not further defined | Unhealthy | Unhealthy |
| Chicken, bite-size pieces, coated, fast food chain, fried, fat not further defined | Unhealthy | Unhealthy |
| Chicken, breast, flesh, skin & fat, coated, fast food chain, fried, undefined fat | Unhealthy | Unhealthy |
| Chicken, breast strip, coated, fast food chain, fried, fat not further defined | Unhealthy | Unhealthy |
| Chicken, drumstick, flesh, skin & fat, coated, fast food chain, fried, undefined fat | Unhealthy | Unhealthy |
| Chicken, finger or chip, purchased frozen, baked, roasted, fried, grilled or BBQ'd, no added fat | Unhealthy | Unhealthy |
| Chicken, kiev, purchased frozen with breadcrumb coating, baked, roasted, fried, grilled or BBQ'd, cooked with or without added fat | Unhealthy | Unhealthy |
| Chicken, nugget, purchased frozen, baked, roasted, fried, grilled or BBQ'd, with or without added fat | Unhealthy | Unhealthy |
| Chicken, nugget, purchased from takeaway chain, fried, canola oil | Unhealthy | Unhealthy |
| Chicken, nugget, purchased from independent & chain takeaway outlets, fried, undefined oil | Unhealthy | Unhealthy |
| Chicken, nugget, purchased from takeaway chain, grilled | Unhealthy | Unhealthy |
| Chicken piece, flesh, skin & fat, coated, fast food outlet, fried, undefined oil | Unhealthy | Unhealthy |
| Chicken, thigh, flesh, skin & fat, coated, fast food chain, fried, undefined fat | Unhealthy | Unhealthy |
| Chicken, wing, flesh, skin & fat, coated, fast food chain, fried, undefined fat | Unhealthy | Unhealthy |
| Chicken, wing, flesh, skin & fat, purchased frozen, breadcrumb coating, baked, roasted, fried, grilled or BBQ'd, with or without added fat | Unhealthy | Unhealthy |
| Pork rind snack | Unhealthy | Unhealthy |
| **Non-alcoholic beverages** | | |
| **Beverage mixes** | | |
| Marshmallow Flavor Hot Cocoa Mix | Unhealthy | Unhealthy |
| Sugar Free Organic Drinking Chocolate | Unhealthy | Unhealthy |
| White Hot Chocolate Balls | Unhealthy | Unhealthy |
| Strawberry Shake Drink | Unhealthy | Unhealthy |
| Sweet Navel Orange Flavoured Beverage Mix with Vitamin C | Unhealthy | Unhealthy |
| Strawberry Flavour Boost | Unhealthy | Unhealthy |
| Strawberry Flavoured Drink | Unhealthy | Unhealthy |
| Lemon Flavour Squirt Drink Concentrate | Unhealthy | Unhealthy |
| Pineapple Mango Water Mixer | Unhealthy | Unhealthy |
| Banana Flavored Mix | Unhealthy | Unhealthy |
| Minions Hot Chocolate Drink | Unhealthy | Unhealthy |
| Organic Cacao Powder | Healthy | Healthy |
| Signature Blend Drinking Chocolate | Unhealthy | Unhealthy |
| Original Malt Drink Mix | Unhealthy | Unhealthy |
| No Added Sugar Drinking Cocoa | Unhealthy | Unhealthy |
| Sweet Navel Orange Drink Mix | Unhealthy | Unhealthy |
| Jamaican Lime Flavoured Drink Mix | Unhealthy | Unhealthy |
| Choc Malt Energy Food Drink | Unhealthy | Unhealthy |
| Choc-a-Choc Milkshake Mix | Unhealthy | Unhealthy |
| Chocoletto Capsules | Unhealthy | Unhealthy |
| Cocoa and Milk Drink Capsules | Unhealthy | Unhealthy |
| Smoothie Vitaboost Coconut Water + Superfruit Smoothie Booster Sachets | Unhealthy | Unhealthy |
| Drinking Chocolate Powder | Unhealthy | Unhealthy |
| Rich Hot Chocolate Capsules | Unhealthy | Unhealthy |
| Cola Flavoured Sparkling Drink Mix | Unhealthy | Unhealthy |
| Cream Soda Flavoured Sparkling Drink Mix | Unhealthy | Unhealthy |
| Cranberry Raspberry Naturally Flavoured Sparkling Water Mix | Unhealthy | Unhealthy |
| Drinking Chocolate | Unhealthy | Unhealthy |
| Dark Chocolate Mix | Unhealthy | Unhealthy |
| Fairtrade Organic Drinking Chocolate | Unhealthy | Unhealthy |
| Slim Bikini Babe Smoothie | Healthy | Healthy |
| Classic Hot Choc Drink | Unhealthy | Unhealthy |
| Extreme Hot Choc Drink | Unhealthy | Unhealthy |
| Honycomb Hot Choc Drink | Unhealthy | Unhealthy |
| Mint Hot Chocolate Drink | Unhealthy | Unhealthy |
| Raw Organic Creamy Drinking Chocolate | Unhealthy | Unhealthy |
| Beetroot Latte Blend | Unhealthy | Unhealthy |
| Spicy Ginger Latte Blend | Unhealthy | Unhealthy |
| Minty Cacao Latte | Unhealthy | Unhealthy |
| Cacao Latte | Unhealthy | Unhealthy |
| Turmeric Latte | Unhealthy | Unhealthy |
| Golden Turmeric Latte | Healthy | Healthy |
| Super Greens Smoothie | Healthy | Healthy |
| Dark Cacao Smoothie | Healthy | Healthy |
| Turmeric Latte | Healthy | Healthy |
| Beetroot Latte | Healthy | Healthy |
| Hot Chocolate Instant Drink | Unhealthy | Unhealthy |
| Turmeric Latte Blend | Healthy | Healthy |
| Orangey Hot Choc Sachets | Unhealthy | Unhealthy |
| Drink Mix | Unhealthy | Unhealthy |
| 99% Sugar Free Drinking Chocolate | Unhealthy | Unhealthy |
| Organic Drinking Chocolate Ruby Bliss | Unhealthy | Unhealthy |
| Caramelly Hot Choc | Unhealthy | Unhealthy |
| Straight Up Cocoa | Healthy | Healthy |
| West Africa 45% Dark Drinking Chocolate | Unhealthy | Unhealthy |
| Salted Caramel Hot Choc | Unhealthy | Unhealthy |
| Instant Ginger Drink | Unhealthy | Unhealthy |
| Turmeric Matcha Flakes | Unhealthy | Healthy |
| Caramel Choc 'n' Stir | Unhealthy | Unhealthy |
| Rocky Road Choc 'n' Stir | Unhealthy | Unhealthy |
| Bio-Fermented Turmeric with Ginger and Black Pepper | Unhealthy | Unhealthy |
| Tropical Flavour Skinny Greens Superfood Detox | Unhealthy | Unhealthy |
| Classic Hot Chocolate | Unhealthy | Unhealthy |
| Instant Drinking Chocolate Powder | Unhealthy | Unhealthy |
| Bio-Fermented Wheatgrass with Beetroot and Carrot Concentrate | Unhealthy | Unhealthy |
| Strawberry Milk Flavouring | Unhealthy | Unhealthy |
| Berry Digest Smoothie Cubes | Unhealthy | Healthy |
| Beetroot and Turmeric Ayurvedic Chai Velvet Latte | Healthy | Healthy |
| Spiced Cacao | Unhealthy | Unhealthy |
| Coconutty Hot Choc Drink | Unhealthy | Unhealthy |
| White Hot Choc Drink Mix | Unhealthy | Unhealthy |
| Rocky Road Hot Choc Drink | Unhealthy | Unhealthy |
| 30% Less Added Sugar Drink Mix | Unhealthy | Unhealthy |
| Chocolate Drink | Unhealthy | Unhealthy |
| Dutch Cocoa | Healthy | Healthy |
| Forest Berries Smoothie | Unhealthy | Unhealthy |
| Super Greens Smothie Cubes | Unhealthy | Unhealthy |
| Matcha & Cacao Latte | Unhealthy | Unhealthy |
| Drinking Chocolate | Unhealthy | Unhealthy |
| Raspberry Hot Choc Drink | Unhealthy | Unhealthy |
| Reishi Mushroom Cacao Mix | Unhealthy | Unhealthy |
| Original Golden Turmeric Elixir | Unhealthy | Unhealthy |
| Malted Milk Mix | Unhealthy | Unhealthy |
| Supergreen Blend | Healthy | Healthy |
| Dark Chocochino Drinking Chocolate | Unhealthy | Unhealthy |
| Drinking Chocolate | Unhealthy | Unhealthy |
| Raw Cacao Drinking Hot Chocolate | Unhealthy | Unhealthy |
| Drink Mix | Unhealthy | Unhealthy |
| Vegetable &/or fruit blend, prepared from dry powder with water | Unhealthy | Healthy |
| Fruit drink, from dry base, regular, recommended dilution | Unhealthy | Unhealthy |
| Beverage base, chocolate flavour, added vitamins A & B3 & Fe | Unhealthy | Unhealthy |
| Beverage base, chocolate flavour, added vitamins A, B1, B2, C, D & folate, Ca & Fe (Milo) | Unhealthy | Unhealthy |
| Beverage base, chocolate flavour, added vitamins A, B1, B2, B3, C, D & folate & Ca (Aktavite) | Unhealthy | Unhealthy |
| Beverage base, malt chocolate flavour, added vitamins A, B1, B2, C, D & folate, Ca & Fe (Milo malt) | Unhealthy | Unhealthy |
| Beverage base, malted milk powder, added vitamins A, B1, B2, B3, B6, B12, biotin, C, D E & folate, Ca & Zn | Unhealthy | Unhealthy |
| Cocoa powder | Unhealthy | Healthy |
| Beverage base, banana flavour (Nesquik brand) | Unhealthy | Unhealthy |
| Beverage base, chocolate flavour, unfortified (Nesquik brand) | Unhealthy | Unhealthy |
| Beverage base, drinking chocolate, unfortified | Unhealthy | Unhealthy |
| Beverage base, malted milk powder, unfortified | Unhealthy | Unhealthy |
| Beverage base, strawberry flavour, unfortified (Nesquik brand) | Unhealthy | Unhealthy |
| Beverage base, strawberry flavour, from drinking straw, containing added sugar & intense sweetener, unfortified | Unhealthy | Unhealthy |
| **Coffee and tea** | | |
| Caramel Flavoured Tea Latte | Unhealthy | Unhealthy |
| Espresso Macchiato Coffee Capsules | Healthy | Healthy |
| Mocha Coffee | Unhealthy | Unhealthy |
| Classic Chai Latte | Unhealthy | Unhealthy |
| Cappuccino | Unhealthy | Healthy |
| Brazilian Café Latte | Unhealthy | Unhealthy |
| Swiss Mochaccino Coffee Mix | Unhealthy | Unhealthy |
| Mocha | Unhealthy | Unhealthy |
| Coconut Latte | Unhealthy | Unhealthy |
| Choc Toffee Latte | Unhealthy | Unhealthy |
| French Vanilla Latte | Unhealthy | Unhealthy |
| Chai Latte | Unhealthy | Unhealthy |
| Wildberry Flavoured Instant Herbal Infusion | Unhealthy | Unhealthy |
| Chai Flavour Tea Latte | Unhealthy | Unhealthy |
| Carbonated Coffee | Healthy | Healthy |
| Matcha Latte | Unhealthy | Unhealthy |
| Spice Chai | Unhealthy | Unhealthy |
| Almond Latte | Unhealthy | Unhealthy |
| Peppermint Choc Bliss Latte | Unhealthy | Unhealthy |
| Almond Cappuccino | Unhealthy | Unhealthy |
| French Vanilla Heaven Latte | Unhealthy | Unhealthy |
| Spiced Choc-Orange Sensation Latte | Unhealthy | Unhealthy |
| Café Latte Instant Coffee Drink | Unhealthy | Unhealthy |
| Cappuccino Instant Coffee Drink | Unhealthy | Unhealthy |
| Mocha Instant Coffee Drink | Unhealthy | Unhealthy |
| Maple Pecan Crave Latte | Unhealthy | Unhealthy |
| Creamy Latte | Unhealthy | Unhealthy |
| Frothy Cappuccino | Unhealthy | Unhealthy |
| Chai Latte | Unhealthy | Unhealthy |
| Free From Dairy Chai Latte | Unhealthy | Unhealthy |
| Choc Matcha | Healthy | Healthy |
| Masala Honey Blend Sticky Chai | Healthy | Healthy |
| Turmeric Super Blend | Healthy | Healthy |
| Caffeine-Free Montville Coffee Concentrate | Healthy | Healthy |
| Strong Cappuccino Coffee | Unhealthy | Unhealthy |
| Original Sparkling Coffee | Unhealthy | Healthy |
| Ice Brew Single Shot Low Calorie Refreshing Coffee | Healthy | Healthy |
| Pomegranate Chai Tea | Unhealthy | Unhealthy |
| Nitro Cold Brew Coffee | Healthy | Healthy |
| Caramel Flavoured Latte | Unhealthy | Unhealthy |
| Ginger Sparkling Coffee | Unhealthy | Unhealthy |
| Skinny Cappuccino | Unhealthy | Unhealthy |
| Mochaccino Coffee | Unhealthy | Unhealthy |
| One High Performance Coffee | Unhealthy | Unhealthy |
| Caramel Latté | Unhealthy | Unhealthy |
| Vanilla Latté | Unhealthy | Unhealthy |
| Original Cappuccino | Unhealthy | Unhealthy |
| Flat White Coffee | Unhealthy | Unhealthy |
| Double Shot Latte | Unhealthy | Unhealthy |
| Sweet Cappuccino | Unhealthy | Unhealthy |
| Turkish Delight Dream Latte | Unhealthy | Unhealthy |
| Roasted Hazelnut Mocha | Unhealthy | Unhealthy |
| Caramel Latte | Unhealthy | Unhealthy |
| Italian Hazelnut Latte | Unhealthy | Unhealthy |
| Salted Caramel Chai Latte | Unhealthy | Unhealthy |
| Cappuccino Coffee Capsules | Healthy | Healthy |
| Matcha Tulsi Latte Blend | Healthy | Healthy |
| Chicory Chai Latte Blend | Healthy | Healthy |
| Matcha Chai Tea | Unhealthy | Unhealthy |
| 99% Sugar Free Mochaccino | Unhealthy | Unhealthy |
| 99% Sugar Free Caramel Latte | Unhealthy | Unhealthy |
| 99% Sugar Free Cappuccino | Unhealthy | Unhealthy |
| White Choc Mocha | Unhealthy | Unhealthy |
| Iced Long Black Flash Brew | Unhealthy | Unhealthy |
| Tea, regular, black, brewed from leaf or teabags, plain, without milk | Healthy | Healthy |
[truncated: 111,903 more chars]
